# Schizophrenia interactome with 504 novel protein–protein interactions

**DOI:** 10.1038/npjschz.2016.12

**Published:** 2016-04-27

**Authors:** Madhavi K Ganapathiraju, Mohamed Thahir, Adam Handen, Saumendra N Sarkar, Robert A Sweet, Vishwajit L Nimgaonkar, Christine E Loscher, Eileen M Bauer, Srilakshmi Chaparala

**Affiliations:** 1 Department of Biomedical Informatics, School of Medicine, University of Pittsburgh, Pittsburgh, PA, USA; 2 Intelligent Systems Program, School of Arts and Sciences, University of Pittsburgh, Pittsburgh, PA, USA; 3 Department of Microbiology and Molecular Genetics, School of Medicine, University of Pittsburgh, Pittsburgh, PA, USA; 4 Cancer Virology Program, University of Pittsburgh Cancer Institute, Pittsburgh, PA, USA; 5 Department of Psychiatry, School of Medicine, University of Pittsburgh, Pittsburgh, PA, USA; 6 Department of Neurology, School of Medicine, University of Pittsburgh, Pittsburgh, PA, USA; 7 Department of Human Genetics, Graduate School of Public Health, University of Pittsburgh, Pittsburgh, PA, USA; 8 Immunomodulation Research Group, School of Biotechnology, Dublin City University, Dublin, Ireland; 9 Department of Surgery, School of Medicine, University of Pittsburgh, Pittsburgh, PA, USA; 10 Vascular Medicine Institute, University of Pittsburgh, Pittsburgh, PA, USA

## Abstract

Genome-wide association studies of schizophrenia (GWAS) have revealed the role of rare and common genetic variants, but the functional effects of the risk variants remain to be understood. Protein interactome-based studies can facilitate the study of molecular mechanisms by which the risk genes relate to schizophrenia (SZ) genesis, but protein–protein interactions (PPIs) are unknown for many of the liability genes. We developed a computational model to discover PPIs, which is found to be highly accurate according to computational evaluations and experimental validations of selected PPIs. We present here, 365 novel PPIs of liability genes identified by the SZ Working Group of the Psychiatric Genomics Consortium (PGC). Seventeen genes that had no previously known interactions have 57 novel interactions by our method. Among the new interactors are 19 drug targets that are targeted by 130 drugs. In addition, we computed 147 novel PPIs of 25 candidate genes investigated in the pre-GWAS era. While there is little overlap between the GWAS genes and the pre-GWAS genes, the interactomes reveal that they largely belong to the same pathways, thus reconciling the apparent disparities between the GWAS and prior gene association studies. The interactome including 504 novel PPIs overall, could motivate other systems biology studies and trials with repurposed drugs. The PPIs are made available on a webserver, called Schizo-Pi at http://severus.dbmi.pitt.edu/schizo-pi with advanced search capabilities.

## Introduction

Schizophrenia (SZ) is a common, potentially severe psychiatric disorder that afflicts all populations.^1^ Gene mapping studies suggest that SZ is a complex disorder, with a cumulative impact of variable genetic effects coupled with environmental factors.^2^ As many as 38 genome-wide association studies (GWAS) have been reported on SZ out of a total of 1,750 GWAS publications on 1,087 traits or diseases reported in the GWAS catalog maintained by the National Human Genome Research Institute of USA^3^ (as of April 2015), revealing the common variants associated with SZ.^4^ The SZ Working Group of the Psychiatric Genomics Consortium (PGC) identified 108 genetic loci that likely confer risk for SZ.^5^ While the role of genetics has been clearly validated by this study, the functional impact of the risk variants is not well-understood.^6,7^ Several of the genes implicated by the GWAS have unknown functions and could participate in possibly hitherto unknown pathways.^8^ Further, there is little or no overlap between the genes identified through GWAS and ‘candidate genes’ proposed in the pre-GWAS era.^9^

Interactome-based studies can be useful in discovering the functional associations of genes. For example, *disrupted in schizophrenia 1* (DISC1), an SZ related candidate gene originally had no known homolog in humans. Although it had well-characterized protein domains such as coiled-coil domains and leucine-zipper domains, its function was unknown.^10,11^ Once its protein–protein interactions (PPIs) were determined using yeast 2-hybrid technology,^12^ investigators successfully linked DISC1 to cAMP signaling, axon elongation, and neuronal migration, and accelerated the research pertaining to SZ in general, and DISC1 in particular.^13^ Typically such studies are carried out on known protein–protein interaction (PPI) networks, or as in the case of DISC1, when there is a specific gene of interest, its PPIs are determined by methods such as yeast 2-hybrid technology.

Knowledge of human PPI networks is thus valuable for accelerating discovery of protein function, and indeed, biomedical research in general. However, of the hundreds of thousands of biophysical PPIs thought to exist in the human interactome,^14,15^ <100,000 are known today (Human Protein Reference Database, HPRD^16^ and BioGRID^17^ databases). Gold standard experimental methods for the determination of all the PPIs in human interactome are time-consuming, expensive and may not even be feasible, as about 250 million pairs of proteins would need to be tested overall; high-throughput methods such as yeast 2-hybrid have important limitations for whole interactome determination as they have a low recall of 23% (i.e., remaining 77% of true interactions need to be determined by other means), and a low precision (i.e., the screens have to be repeated multiple times to achieve high selectivity).^18,19^ Computational methods are therefore necessary to complete the interactome expeditiously. Algorithms have begun emerging to predict PPIs using statistical machine learning on the characteristics of the proteins, but these algorithms are employed predominantly to study yeast. Two significant computational predictions have been reported for human interactome; although they have had high false positive rates, these methods have laid the foundation for computational prediction of human PPIs.^20,21^

We have created a new PPI prediction model called High-Confidence Protein–Protein Interaction Prediction (HiPPIP) model. Novel interactions predicted with this model are making translational impact. For example, we discovered a PPI between OASL and DDX58, which on validation showed that an increased expression of OASL could boost innate immunity to combat influenza by activating the RIG-I pathway.^22^ Also, the interactome of the genes associated with congenital heart disease showed that the disease morphogenesis has a close connection with the structure and function of cilia.^23^ Here, we describe the HiPPIP model and its application to SZ genes to construct the SZ interactome. After computational evaluations and experimental validations of selected novel PPIs, we present here 504 highly confident novel PPIs in the SZ interactome, shedding new light onto several uncharacterized genes that are associated with SZ.

## Results

**Figure 1:**
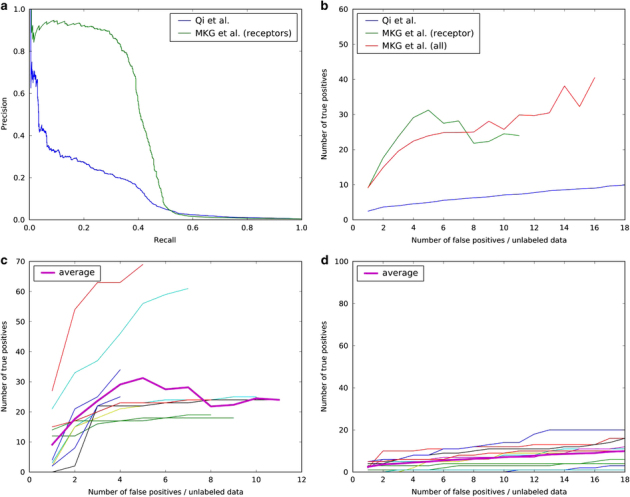
Computational evaluation of predicted protein–protein interactions on hub proteins: (**a**) precision recall curve. (**b**) True positive versus false positives in ranked lists of hub type membrane receptors for our method and that by Qi *et al.* True positives versus false positives are shown for individual membrane receptors by our method in (**c**) and by Qi *et al.* in (**d**). Thick line is the average, which is also the same as shown in (**b**). Note: *x*-axis is recall in (**a**), whereas it is number of false positives in (**b**–**d**). The range of *y*-axis is observed by varying the threshold from 1.0–0 in (**a**), and to 0.5 in (**b**–**d**).

We developed a computational model called HiPPIP to predict PPIs (see Methods and Supplementary File 1). The model has been evaluated by computational methods and experimental validations and is found to be highly accurate. Evaluations on a held-out test data showed a precision of 97.5% and a recall of 5%. 5% recall out of 150,000 to 600,000 estimated number of interactions in the human interactome corresponds to 7,500–30,000 novel PPIs in the whole interactome. Note that, it is likely that the real precision would be higher than 97.5% because in this test data, randomly paired proteins are treated as non-interacting protein pairs, whereas some of them may actually be interacting pairs with a small probability; thus, some of the pairs that are treated as false positives in test set are likely to be true but hitherto unknown interactions. In Figure 1a, we show the precision versus recall of our method on ‘hub proteins’ where we considered all pairs that received a score >0.5 by HiPPIP to be novel interactions. In Figure 1b, we show the number of true positives versus false positives observed in hub proteins. Both these figures also show our method to be superior in comparison to the prediction of membrane-receptor interactome by Qi *et al*’s.^24^ True positives versus false positives are also shown for individual hub proteins by our method in Figure 1c and by Qi *et al*’s.^23^ in Figure 1d. These evaluations showed that our predictions contain mostly true positives. Unlike in other domains where ranked lists are commonly used such as information retrieval, in PPI prediction the ‘false positives’ may actually be unlabeled instances that are indeed true interactions that are not yet discovered. In fact, such unlabeled pairs predicted as interactors of the hub gene HMGB1 (namely, the pairs HMGB1-KL and HMGB1-FLT1) were validated by experimental methods and found to be true PPIs (See the Figures e–g in Supplementary File 3). Thus, we concluded that the protein pairs that received a score of ⩾0.5 are highly confident to be true interactions. The pairs that receive a score less than but close to 0.5 (i.e., in the range of 0.4–0.5) may also contain several true PPIs; however, we cannot confidently say that all in this range are true PPIs. Only the PPIs predicted with a score >0.5 are included in the interactome.

### SZ interactome

**Figure 2:**
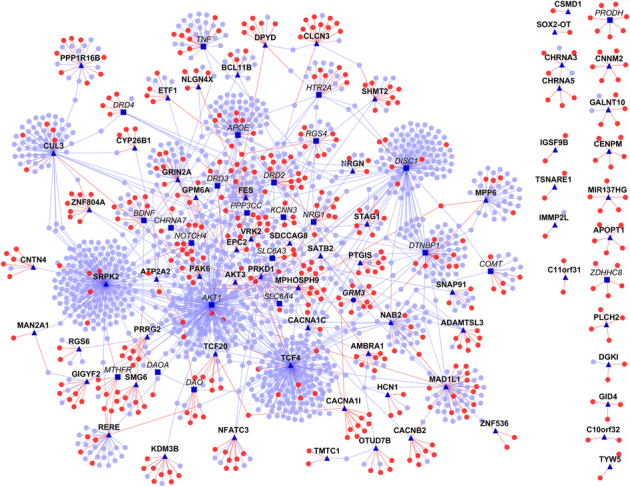
Schizophrenia interactome: network view of the schizophrenia interactome is shown as a graph, where genes are shown as nodes and PPIs as edges connecting the nodes. Schizophrenia-associated genes are shown as dark blue nodes, novel interactors as red color nodes and known interactors as blue color nodes. The source of the schizophrenia genes is indicated by its label font, where Historic genes are shown italicized, GWAS genes are shown in bold, and the one gene that is common to both is shown in italicized and bold. For clarity, the source is also indicated by the shape of the node (triangular for GWAS and square for Historic and hexagonal for both). Symbols are shown only for the schizophrenia-associated genes; actual interactions may be accessed on the web. Red edges are the novel interactions, whereas blue edges are known interactions. GWAS, genome-wide association studies of schizophrenia; PPI, protein–protein interaction.

**Figure 3:**
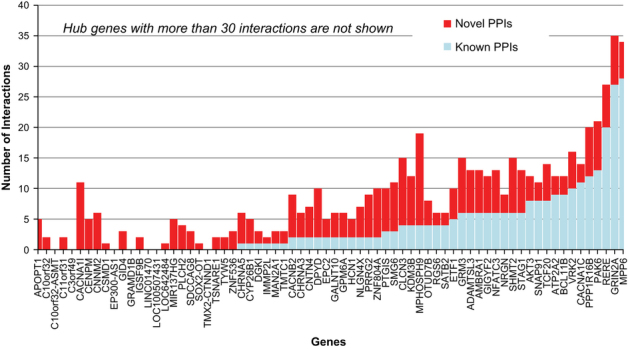
Number of novel and known PPIs of GWAS genes. For each gene, the number of known PPIs is shown in light blue color and novel PPIs is red color. GWAS, genome-wide association studies of schizophrenia; PPI, protein–protein interaction.

By applying HiPPIP to the GWAS genes and Historic (pre-GWAS) genes, we predicted over 500 high confidence new PPIs adding to about 1400 previously known PPIs. Many genes that had no known PPIs previously have several novel PPIs following our analyses. Figure 2 shows the network of protein–protein interactions between SZ genes, where a gene is shown as a square or circular node and a protein–protein interaction between two genes is shown as a line (also called ‘edge’) between the genes. SZ-associated genes are shown as square shaped dark blue nodes, of which those with bold labels are GWAS genes, and those with italicized labels are Historic genes. Genes that are novel interactors are shown as red colored nodes and those that are known interactors as blue colored nodes. Novel PPIs are shown as red edges and known PPIs as blue edges. The interactome figures presented here have been generated with Cytoscape.^25^ There are altogether 101 SZ genes (77 GWAS genes and 25 Historic genes with 1 gene in common). We predicted 504 novel PPIs adding to 1,397 previously known PPIs. The lists of GWAS and Historic genes and the number of interactions for each of them are given in Supplementary File 2. Figure 3 shows the number of known and novel PPIs for each of the GWAS genes; it may be seen that novel PPIs have been predicted for several genes that previously had few known PPIs.

We calculated the average distance (average length of shortest paths) between pairs of SZ genes with and without predicted interactions, and compared them with those of random pairs of genes. The average distance dropped by 4.1 edges when novel PPIs are included (average distance was 5.6 vs 9.7 edges, respectively, with and without novel PPIs). For a set of random genes, the drop in average distance was 3.4 edges (average distance was 9.4 vs 12.8 edges, respectively, with and without predicted PPIs), averaged over 1,000 trials. The drop in average distance between the SZ genes was significantly higher than the drop in average distance between random genes, with a *P* value<0.005 (i.e., the predicted interactions make SZ genes come closer to each other than they do random genes).

### Experimental validations of predicted interactions

We carried out experimental validations of nine predicted PPIs namely STT3A-RPS25, STT3A-MCAM, STT3A-SCN4B, HMGB1-KL, HMGB1-FLT1 and STX3-LPXN with a score of 0.6, STX4-MAPK3 and STT3A-SYCP3 with score 0.5, and DDX58-OASL with score 0.4. Seven of the interactions were studied with co-immunoprecipitation and two by co-localization. Supplementary File 3 shows the validation results of the six interactions, while the remaining three validations along with protocol details for all the experiments are presented in Supplementary File 1. Forward and reciprocal co-immunoprecipitation was performed for each protein pair in question and it provided strong evidence for each of these interactions. All validations tested positive. Validation at 100% hit-rate of all the selected predicted interactions in addition to the computational validations further supports that these predicted interactions are highly confident to be true interactions. While some validated interactions are those of SZ-associated gene STT3A,^26^ some of the other interactions were of proteins that have previously been reported to be associated with SZ: LPXN is involved in adhesion-mediated signaling.^27^ Interestingly, there is evidence for altered focal adhesion dynamics in SZ.^28^ STX3 and STX4 are members of the SNARE family of proteins which are known to be involved in facilitating secretion from a wide variety of cell types including neuronal cells. Furthermore, syntaxin proteins have been implicated in SZ;^29^ and MAPK genes have been shown to have altered activity in SZ.^30^ MCAM, which is predicted to interact with STT3A, has been shown to have strong effects on learning in mice.^31^ Another predicted and validated STT3 interactor, RPS25, has also been found to be downregulated in some mental disorders.^30,32^

### Webserver of SZ interactome

We have made the known and novel interactions of all SZ-associated genes available on a webserver called Schizo-Pi, at the address http://severus.dbmi.pitt.edu/schizo-pi. This webserver is similar to Wiki-Pi^33^ which presents comprehensive annotations of both participating proteins of a PPI side-by-side. The difference between Wiki-Pi which we developed earlier, and Schizo-Pi, is the inclusion of novel predicted interactions of the SZ genes into the latter. Novel PPIs are shown highlighted in yellow in search results. For each protein in a PPI, annotations of pathways, other GWAS associations, Gene Ontology (GO), diseases, drugs, and other such annotations are shown. Schizo-Pi allows users to search for interactions by specifying criteria about both the proteins involved, in ways that were not possible with search engines on other databases. Queries can be constructed to include or exclude any field of annotations such as GO annotations, diseases, drugs, and/or pathways for either gene involved in an interaction. For example, a user may search for ‘interactions between one protein that is associated with SZ and the other protein that is associated with cilium’ to obtain these results: http://severus.dbmi.pitt.edu/schizo-pi/index.php/search/adv?a-all=schizophrenia&b-all=cilium&a-any=&b-any=&a-none=cilium&b-none=.

Schizo-Pi currently includes novel PPIs of the GWAS genes, Historic genes, as well other genes listed in the OMIM database to be SZ-associated genes. The functional role of the associated SNPs in regulating expression of the set of 77 genes is a work in progress. A recent comprehensive analysis indicated that non-coding variants from many GWAS studies function as cis-acting regulators of adjoining coding regions.^34^ Many genetic risk factors for SZ remain to be identified, particularly the rare variants. Our motivation to pick the genes identified in the recent Psychiatric Genomic Consortium’s GWAS genes analysis was to use a set of SNPs and related genes about which there is broad consensus in the field. As additional genes are identified, their novel PPIs will be predicted and presented on Schizo-Pi. To facilitate this, we have added a feature on the website where a user may submit information about other genes that should be included in the webserver. The user would need to provide the gene symbol and a publication that describes its relevance to SZ. Each month, we will verify this information and include the PPIs of these newly submitted genes to the website.

## Discussion

Despite the many advances in biomedical research, identifying the molecular mechanisms underlying the disease is still challenging. Studies based on protein interactions were proven to be valuable in identifying novel gene associations that could shed new light on disease pathology.^35^ The interactome including more than 500 novel PPIs will help to identify pathways and biological processes associated with the disease and also its relation to other complex diseases. It also helps identify potential drugs that could be repurposed to use for SZ treatment.

### Functional and pathway enrichment in SZ interactome

When a gene of interest has little known information, functions of its interacting partners serve as a starting point to hypothesize its own function. We computed statistically significant enrichment of GO biological process terms among the interacting partners of each of the genes using BinGO^36^ (see online at http://severus.dbmi.pitt.edu/schizo-pi).

For example, for the GWAS genes MPHOSPH9 and PRRG2 that have no known GO terms, we predicted several enriched GO terms by including their interacting partners. The terms include regulation of ion channels, sodium ion transport, sodium ion transmembrane transport, negative regulation of voltage-gated calcium channel activity, which could be very relevant as voltage-gated ion channels have important role in neurotransmission and synaptic plasticity in the nervous system and are known to be involved in major neuropsychiatric disorders like SZ and bipolar disorders.^37^ The GO term enrichment analysis of Historic gene *PRODH* revealed that the neuronal terms like synaptic vesicle targeting, regulation of synaptic vesicle transport and exocytosis, and parasympathetic nervous system development are enriched among the interacting partners. Synaptic vesicles are regarded as key organelles in synaptic function and release of neurotransmitters.^38^ These findings further support the role of these novel genes in nervous system development and neurotransmission.

Next, we identified the pathways that have highly significant overlap with SZ interactome using IPA (Ingenuity Systems, www.ingenuity.com). Supplementary File 4 shows the pathways that are associated with the interactomes with their *P* values and pathway-associated genes. Pathways related to neuronal function and development, including synaptic plasticity and neurotransmission, are found significantly enriched in the interactome. Examples are synaptic long-term potentiation, neuropathic pain signaling in dorsal horn neurons, dopamine–DARPP32 feedback in cAMP signaling, neuregulin signaling, reelin signaling, CREB signaling in neurons, calcium signaling, 14-3-3-mediated signaling, and eNOS signaling. There are 15 novel interactors associated with dopamine signaling, 17 with axonal guidance signaling, 8 with synaptic pathways. In addition, other immune system and inflammation-related pathways were also observed with high statistical significance. These processes such as neurotransmission, synaptic plasticity, calcium signaling, immune system and inflammation were previously shown to be dysregulated in SZ through proteomic studies.^39^

We observed that while all the significant pathways are retrieved even when the interactome with only known PPIs is used, inclusion of novel PPIs reveals more connections of the SZ genes with these pathways. For example, *RAR* activation pathway is retrieved even when the interactome with only known PPIs is analyzed; however, including the novel PPIs in the analysis shows that GWAS genes connect to eight more genes of this pathway through novel interactions (*PTEN, CRABP1, GTF2H2, PRKAR1B, RDH5, SRA1, GNAS, PRKAG1*) and Historic genes connect to two additional genes (*ADCY10* and *CRABP2*) through novel PPIs. RAR signaling pathways have been linked to synaptic plasticity, dopamine regulation, and learning and memory deficits.^40,41^ Some of these novel interactors such as PTEN, PRKAR1B in this pathway are shown to be associated with neurodegenerative and neuropsychiatric disorders such as autism.^42,43^ Therefore, the interactors of RAR activation pathway may have a potential role in neuronal function such as neurotransmission. Supplementary File 4 which lists the interactors and the genes involved in each of the pathways, respectively, serves as a valuable resource for prioritizing of genes for future research. Specifically, 17 genes of axonal guidance signaling (*ERAP2, PDGFB, FZD3, PRKAR1B, GNAS, PRKAG1, PDIA3, ARPC3, ADAMTS8, EPHA2, TUBA1C, RTN4R, ADAM23, RND1, SEMA7A, NRP1, MYL9*), and 10 genes of dopamine-related pathways (*KCNJ3, ADCY10, KCNJ4, PRKAG1, GNAS, PDIA3, PPP1R11, CREM, PRKAR1B, SLC18A1*) are discovered to be interactors of SZ genes, in addition to the 73 and 41 genes from these pathways, respectively, that were known to interact previously.

SZ interactome also showed an overlap of pathways related to other diseases, most notably cancer. Cancer-associated pathways such as molecular mechanisms of cancer, colorectal cancer metastasis signaling, breast cancer regulation by stathmin1, ovarian cancer signaling, prostate cancer signaling, small cell lung cancer signaling and thyroid cancer signaling are significantly enriched in both the GWAS and Historic gene interactomes. Cancer pathways often show association to several disease gene networks owing to the fact that these pathways are not only very heavily represented in the databases but also because they consist of genes that carry out basic cellular functions that are central to all biological processes. However, the *P* value computation accounts for the total size of the pathway in the database, while considering its overlap with the gene set. The observed *P* values for these cancer pathways are found to be highly significant in the interactome. It is not only the statistical significance of the pathway that is revealed in this analysis, but also specifically the novel interconnections of SZ genes with cancer pathway-associated genes. These findings through interactome analyses further strengthen the link between cancer and SZ, and highlight specific proteins for future investigation (Supplementary File 4). There are previous studies that suggested that there is a close relationship between some cancers and psychiatric diseases such as depression.^44,45^ It is interesting to note that two more pathways that are associated with sex hormones, estrogen receptor signaling, and androgen signaling, are also associated with the interactomes. These pathways are particularly interesting, as clinical data has long suggested a link between sex and SZ. Increased serum levels of testosterone have been linked to impaired cognitive function in men with SZ.^46^ There is also evidence to suggest that estrogen is protective and/or therapeutic in women with SZ^47^ and there is an interest in leveraging this to develop compounds for men.^48^ Finally, some studies have linked estrogen receptor alleles to risk for diseases.^49^ Our findings provide another link between genetic risk for SZ and protein interactors within these sex hormone signaling pathways, and highlight specific genes and PPIs for future hypothesis testing.

### GWAS versus Historic (pre-GWAS) risk genes of SZ

**Figure 4:**
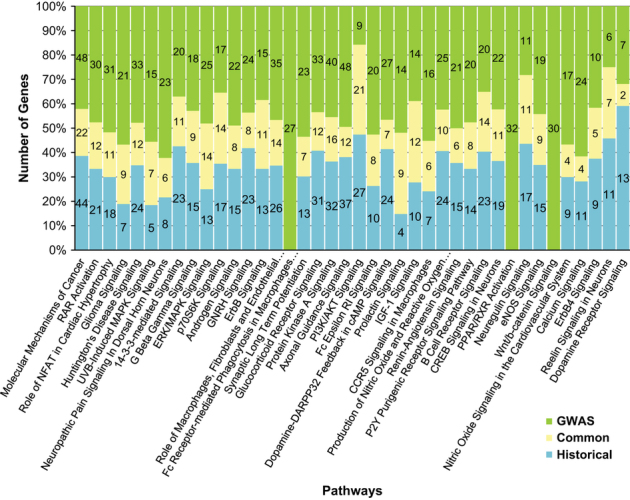
Overlap of significant pathways from GWAS interactome and Historic gene interactome. Pathway associations are computed with Ingenuity Pathway Analysis. Pathways shown are the top 30 pathways in the GWAS interactome, and additionally, some of the pathways that are known to be associated with SZ. Number of genes associated with GWAS interactome is shown in green, with Historic gene interactome in blue and common to both in yellow. GWAS, genome-wide association studies of schizophrenia; PPI, protein–protein interaction.

We used the interactome analyses to study the apparent disconnectedness between the SZ risk genes identified through GWAS studies and those considered as risk genes in pre-GWAS era. We compiled the interactomes of the 77 GWAS genes and the 25 Historic genes separately. HiPPIP predicted 365 novel PPIs of GWAS genes adding to 819 previously known PPIs. Correspondingly, HiPPIP predicted 147 high confidence new PPIs of Historic genes adding to 519 previously known PPIs (see Supplementary Files 5 and 6). Seventeen GWAS genes that had no known PPIs now have altogether 58 novel PPIs. Two Historic genes (*PRODH* and *ZDHHC8*) that had previously no known PPIs now have 12 novel PPIs. Although there is only one gene overlap between GWAS and Historic gene sets, there is a highly significant overlap between their interactomes; 109 genes are common to the 2 interactomes, leading to a statistical significance of *P* value<10^−21^. We compared the shortest path distances from each of the GWAS genes to its closest Historic gene. With the inclusion of novel PPIs, 16 GWAS genes that previously had no connecting paths to the Historic genes now get connected with 2 to 4 edges between them. The histogram of shortest path distances with and without novel PPIs is shown in Supplementary File 7. Could it be that although the gene sets are different, they are related in biological functionality? To study this, we computed the pathways associated with the two interactomes separately and then compared the overlap. We find that the overlap is far richer than the simple overlap of the two interactomes themselves. Figure 4 shows the top 30 pathways of the GWAS interactome and some additional SZ relevant pathways, and the number of genes associated with each of the pathways from GWAS interactome only (green), Historic gene interactome only (blue), and common to both (yellow). It may be seen that almost all of the pathways are associated with both the interactomes; further, the common pathway association arises not only because of the shared interactors but also because of unique genes in each interactome. For example, consider the *RAR* activation pathway; while 12 genes that are common to both interactomes are associated with it, 30 genes that are unique to the GWAS interactome and 21 genes that are unique to the Historic gene interactome are also associated with it. In summary, although GWAS and Historic gene sets appear to be distinct, they share a significant number of interactors, and show a significant overlap of functional pathways. Our analyses thus help to reconcile an ongoing, vigorous debate in the SZ genetics community regarding the pathogenic significance of research in the pre-GWAS era. We repeated this approach with two randomly chosen sets of genes of same sizes, and found that there was no visible relation between them. As IPA analysis is cumbersome and the software license permits computation of only two such sets a day, we did not repeat this large number of times to compute statistical significance.

### Overlap of the interactome with proteomic studies

Some recent reviews of proteomics studies have cataloged the proteins reported as differentially expressed in one or more studies of SZ, explaining the common pathways or functions that were recovered in various proteomic studies.^39,50^ We found that only one Historical gene (*DISC1*) and none of the GWAS genes were found in these proteomic studies. We evaluated whether proteins identified in these reviews were represented in our interactome. Of the differentially expressed proteins in the gray matter of SZ postmortem brains,^50^ 34 were found as known interactors and 7 as novel interactors in the interactome, with statistical significance of *P*-value<10^−8^ by hypergeometric test. Of the genes that were differentially expressed in the white matter of SZ postmortem brains,^50^ 18 were known interactors and 3 were novel interactors in the interactome. Four gray matter proteins (ATP6V1B2, DPYSL2, NEFL and TUBA1C) and six white matter proteins (ATP6V1B2, DPYSL2, NEFL, NEFM, STX1A and TUBB2A) from the interactome were previously implicated at mRNA level in SZ brains.^50^ Of the genes cataloged to be found in the proteome of SZ,^39^ 16 genes associated to neuronal transmission and synaptic function, calcium homeostasis and signaling, energy metabolism, oxidative stress, cytoskeleton, immune system and inflammation were found to be known interactors (statistical significance of the overlap in comparison to all the proteins reviewed with hypergeometric test was *P*-value<0.0003). Thus, we find that a common theme emerges through the interactome which connects the GWAS genes and Historical genes to each other and to a significant number of genes found through proteomic studies. A complete list of genes from the interactome that were found in these proteomics studies are given in Supplementary File 8.

### Novel PPIs connect SZ genes to 20 drug target genes

The possibility and the immense value of drug repurposing are well-understood today. In 2010, it was shown that the drug Comtan (active component: entacapone) that treats Parkinson’s disease can potentially be repurposed to treat multidrug-resistant and extremely-drug resistant forms of tuberculosis.^51^ Comtan is an inhibitor of COMT, which is one of the SZ-associated genes.

**Figure 5:**
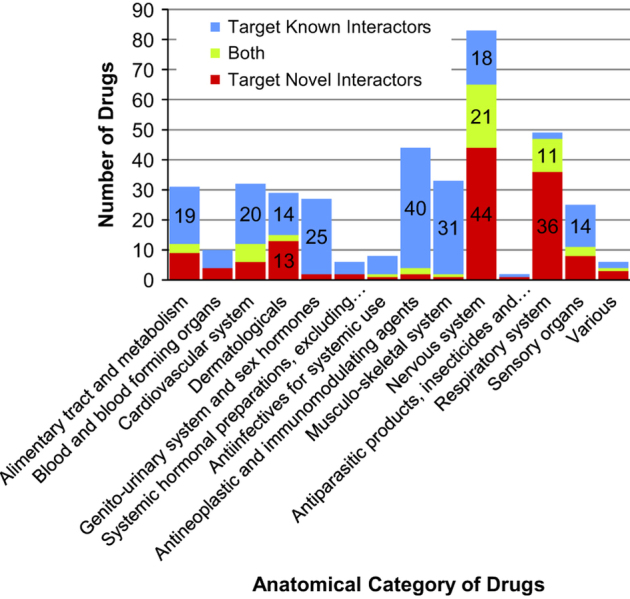
Number of drugs that target the genes in the GWAS interactome: the numbers are shown separated by the anatomic category of the drugs (Anatomic, Therapeutic and Chemical classification) and also separated by whether they target known interactors (blue) or novel interactors (red) or both (green). GWAS, genome-wide association studies of schizophrenia.

We analyzed which of the genes in the interactomes are drug targets, and what diseases these drugs treat. In GWAS interactome, there are 74 genes that are targets of 307 unique drugs (of which 20 are new interactors that are targeted by 131 drugs). Figure 5 shows categorization of these 307 drugs by the Anatomic category of the Anatomic, Therapeutic and Chemical (ATC) classification of the drugs: Nervous system (83 drugs), alimentary tract and metabolism (31 drugs), musculoskeletal system (33 drugs), cardiovascular system (32 drugs), respiratory system (47 drugs), antineoplastic and immunomodulating agents (44 drugs), and genitourinary and sex hormones (27 drugs) (some drugs fall into more than 1 category). Of the nervous system drugs that target novel interactors, 6 drugs are antiepileptics, 34 are calming or stimulating drugs (i.e., psycholeptics and psychoanaleptics), 2 are anesthetics, 2 are anti-Parkinson drugs, and 2 are other nervous system drugs. Corresponding figure for drugs that target Historic gene interactome is shown in Supplementary File 9.

**Figure 6:**
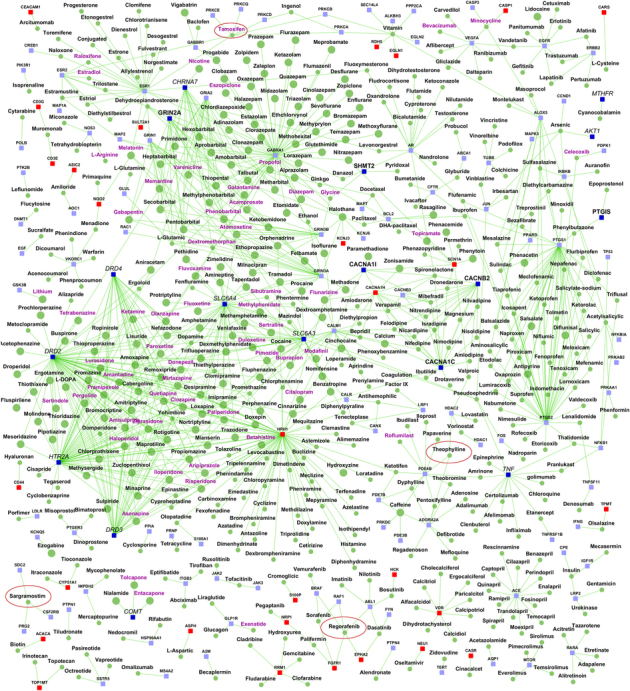
Gene–drug interactome. The network shows the drugs that target genes from the schizophrenia interactome. Drugs are shown as round nodes colored in green and genes as square nodes colored in dark blue (schizophrenia genes), light blue (known interactors), and red (novel interactors). Nervous system drugs (based on Anatomic category of ATC classification) are shown as larger size green colored nodes compared with other drugs. Drugs that are in clinical trials for schizophrenia are labeled purple. ATC, anatomic, therapeutic and chemical classification.

Detailed network of drugs and their targets among SZ interactome genes is shown in Figure 6, in which genes are colored and labeled same as in Figure 2, while drugs are shown as green colored nodes. Nervous system drugs are shown in larger size; thus presenting a birds-eye view of other (non-nervous system) drugs that can be potentially repurposed for SZ. To highlight this, we labeled the drugs that are already in clinical trials for SZ with purple-color labels and it may be seen that a number of them are indeed non-nervous system-related drugs. The figure also visually presents drugs that interact with multiple SZ risk genes as potential candidates for repurposing. Examples are sargramostim, regorafenib, theophylline, which are cancer and respiratory drugs. List of all the drugs that target various genes in the SZ interactome are shown in Supplementary File 10, with detailed characterization of their ATC codes and whether they target GWAS or Historic risk genes or their known or novel interactors. Supplementary File 10 also shows drugs that are found to be significantly connected to SZ genes (see methods in Supplementary File 1).

We further explored the genes in the GWAS interactome that are targeted by nervous system drugs to identify potential drug targets for SZ. The list includes 5 novel interactors HRH1, GRIA2, KCNJ3, SCN1A, and CACNA1H and 6 known interactors GRIN3A, GRIN1, CALM3, BCL2, PDE4B, and PTGS1. We predicted novel interactors of these drug targets (see Supplementary File 11), and found that seven of these drug targets had few known interactions but many novel interactions. HRH1 with 12 novel interactions, SCN1A with 6, PDE4B with 4, PTGS1 with 6, CACNA1H with 9, and KCNJ3 with 8 novel interactors. It is interesting to note that NAB2, a GWAS gene, is predicted to interact with two of these novel genes HRH1 and SCN1A that are targeted by nervous system drugs. SCN1A is known to be associated with neuronal disorder epilepsy, and HRH1 is a histamine receptor that belongs to the family of G protein coupled receptors associated with neurotransmission. Furthermore, HRH1 is targeted by not only nervous system drugs but also many respiratory drugs. Therefore, these genes, NAB2, HRH1 and SCN1A can be tested further to identify their therapeutic value to SZ treatment.

### Conclusions and future investigations

We developed an algorithm to predict protein–protein interactions of genes and presented the novel interactions that are deemed to be highly accurate according to computational evaluations and experimental evaluations on some of the novel PPIs. We then identified novel interactions of SZ risk genes, from which we identified the pathways that are enriched in their interactome, identified drugs that target the interactors, and enriched their GO term associations. Our analysis illustrates that despite the disparity of genes/proteins found in various studies to characterize SZ, a commonality emerges through the interaction network and the pathways associated with the interactome. We developed a search-enabled website which presents the PPIs with comprehensive information about the genes involved in the PPIs. It would be meaningful to validate the novel PPIs of SZ risk genes in postmortem brain tissues, as the experimental evaluations that we carried out were in non-CNS tissues. An important next step will be to validate these predictions at the level of protein network alterations within brain tissue from subjects with SZ.^52^ Protein interactome of SZ genes would be useful in carrying out network-based systems biology studies, while each predicted interaction would itself have a potential to advance SZ research.

## Materials and methods

### Data

The SZ Working Group of the PGC recently combined and re-assessed the data from the various GWAS available for SZ, and identified 108 genomic loci likely to be associated with SZ.^5^ These loci corresponded to single nucleotide polymorphisms in 77 genes. In addition, we considered 25 genes that are believed to be SZ-associated from pre-GWAS era, which were recently evaluated for their continued relevance to the disease.^9^ The two gene sets, which will be referred to as GWAS genes and Historical genes, respectively, had an overlap of only one gene (i.e., GRM3).

Random genes sets used in shortest path comparisons were sampled from about 20,000 human proteins listed in Ensembl database (www.ensembl.org). Drugs, and their ATC codes and drug targets were downloaded from DrugBank (www.drugbank.ca/). Drugs that are currently in clinical trials were downloaded from ClinicalTrials.gov with the following query: condition: SZ and intervention: drug; withdrawn studies were not used here.

For algorithm development of PPI prediction, the training data were composed of 20,000 known interactions collected from HPRD, mixed with 80,000 random pairs, making sure that the random pairs do not contain any of the known PPIs. The fraction of known interactions in the test set was kept low more realistically at 0.3% in a total of 160,000 pairs. We considered all the proteins that had more than 50 known PPIs as hub proteins for evaluations.

### Prediction model—HiPPIP

PPIs were predicted by computing features of protein pairs and developing a random forest model to classify the pairwise features as interacting or non-interacting. Protein annotations that were used in this work are: cellular localization, molecular function and biological process membership, location of the gene on the genome, gene expression in hundreds of microarray experiments, protein domains and tissue membership of proteins. Computation of features of protein pairs is described earlier in the study by Thahir *et al.* ^53^ A random forest with 30 trees was trained using the feature offering maximum information gain out of 4 random features to split each node; minimum number of samples in each leaf node was set to be 10. The random forest outputs a continuous valued score in the range of [0,1]. The threshold to assign a final label was varied over the range of the score for positive class (i.e., 0 to 1) to find the precision and recall combinations that are observed (Figure 1). This prediction model is referred to as HiPPIP model.

### Evaluation of PPI prediction model

Evaluations on a held-out test data showed a precision of 97.5% and a recall of 5% at a threshold of 0.75 on the output score. Next, we created ranked lists for each of the hub genes (i.e., genes that had >50 known PPIs), where we considered all pairs that received a score >0.5 to be novel interactions. The predicted interactions of each of the hub genes are arranged in descending order of the prediction score, and precision versus recall is computed by varying the threshold of predicted score from 1 to 0. Next, by scanning these ranked lists from top to bottom, the number of true positives versus false positives was computed.

### Novel PPIs in the SZ interactome

Each SZ gene, say Z, is paired with each of the other human genes (G_1_, G_2_, …, G_N_), and each pair is evaluated with the HiPPIP model. The predicted interactions of each of the SZ genes (namely, the pairs whose score is greater than the threshold 0.5) were extracted. These PPIs, combined with the previously known PPIs of SZ genes collectively form the SZ interactome.

Note that 0.5 is the threshold chosen not because it is the midpoint between the two classes, but because the evaluations with hub proteins showed that the pairs that received a score >0.5 are highly confident to be interacting pairs. This aspect was further validated by experimentally validating a few novel PPIs above this score.

### Additional details

Additional details of methods are presented in Supplementary File 1.

### Availability of data and materials

The protein–protein interactions of SZ interactome are available on a webserver with free access at http://severus.dbmi.pitt.edu/schizo-pi/. The algorithms used here are implemented with the Weka package (http://sourceforge.net/projects/weka/). Features were computed with programs written in Java. The code for feature processing was implemented with many server configurations that are not portable for distribution. However all novel PPIs are made available freely on the webserver and lists of interactors are also given as Supplementary Data.
