# Supplementary material for: Schizophrenia interactome with 504 novel protein–protein interactions
Source: NPJ Schizophr. 2016 Apr 27;2:16012–. doi: 10.1038/npjschz.2016.12 (PMC4898894; doi:10.1038/npjschz.2016.12)
Supplement: Supplementary File 1 [file npjschz201612-s1.pdf]

Novel Protein-Protein Interactions in the Schizophrenia interactome

Supplementary Material - Methods

Experimental validations of predicted interactions

Table S1-1: Protein pairs that were validated by experiments and found to be true interactions are shown in the table, along with their prediction score.

S. No.	Gene1	Gene2	Score
1	STT3A	RPS25	0.65
2	HMGB1	KL	0.64
3	STT3A	MCAM	0.62
4	STX3	LPXN	0.61
5	HMGB1	FLT1	0.58
6	STT3A	SYCP3	0.51
7	STX4	MAPK3	0.48
8	DDX58	OASL	0.41

Validation of STT3A interactions and HMGB1 interactions (Supplemental File 6 for Figure): All animal procedures were carried out in accordance with the guidelines set by the University of Pittsburgh Institutional Animal Care and Use Committee (IACUC) and with IACUC approval. C57BL/6J mice were purchased from The Jackson Laboratory. Livers and pancreata were dissected and flash frozen. Frozen tissue was homogenized in lysis buffer (50 mm Tris-HCl, pH 7.4, 1% v/v Nonidet P-40, 1mM EDTA, 125 mM NaCl, 0.1% deoxycholic acid, 10 mm NaF, 1 mm vanadate, 10 µg/ml aprotinin, 10 µg/ml leupeptin). Insoluble material was removed by centrifugation and protein concentrations determined using the Bio-Rad DC protein assay. Tissue was precleared with beads (50 µl/sample 15 min) followed by STT3A immunoprecipition from lysates using antibodies (4µg/sample) conjugated to protein G dynabeads (Invitrogen). The optimized precleared lysate was subsequently used for the

immunoprecipitation experiments. All lysates used in this study are precleared prior to the actual IP- experiment.

Control experiments (see Figure 1)

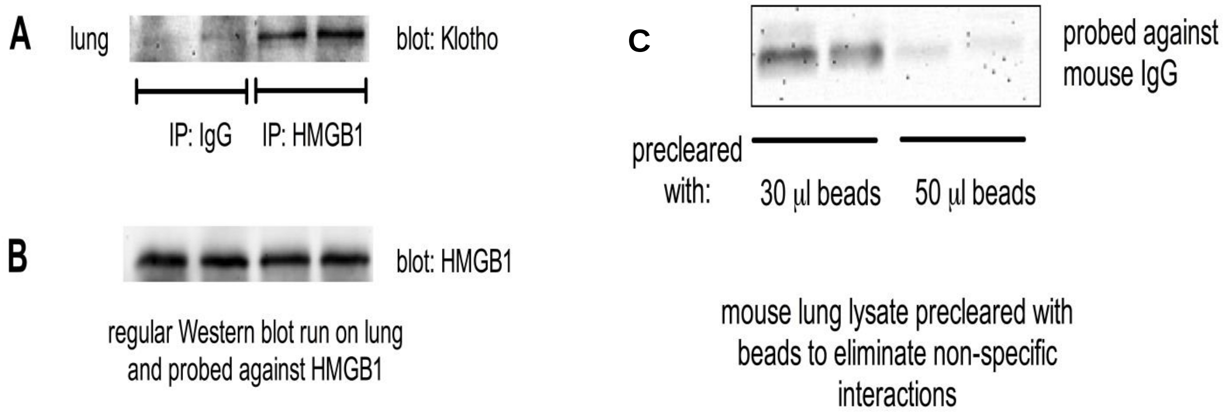

Figure 1: A) Lung lysate was precleared followed by immunoprecipitation using either HMGB1 antibody or non-specific IgG as control. Subsequent Western blot analysis against Klotho resulted in positive interaction with HMGB1 but was negligible for the control. **B)** electrophoresis and western blotting against HMGB1 of the same total lysate (prior to IP) used in A. **C)** Lung lysate was mixed for 15 min at room temperature with 30 or 50 ul of magnetic dynabeads . Beads were then removed from lysate using the magnet and precleared lysate was subjected to electrophoresis and Western blotting against immunoglobulin reactivty resulting in elimination of non-specific binding. The optimized precleared lysate was subsequently used for the immunoprecipitation experiments. All lysates used in this study are precleared prior to the actual IP- experiment.

Reciprocal immunoprecipitations were performed as well (STT3A: sc-168218, sc-390227, Santa Cruz, RPS25, 23599-1-AP, ProteinTech; SCP3 LS-B175 LS Bio, 611230, BD Bioscience, MCAM: bs-1618R Bioss, ab24577 Abcam ; HMGB1, AB18256 Abcam; KL, NBP1-76511; FLT1, SC-316; Santacruz). The antibody-conjugated beads were washed by magnetic separation, and equal amounts of protein samples (200μg/sample) were added to washed beads. After 15 min of incubation, the immune complexes were isolated by magnetic separation, washed, and eluted at 70 °C in 1× SDS-PAGE sample buffer for 10 min. Immunoprecipitated proteins were separated by SDS-PAGE and transferred to nitrocellulose membranes. Membranes were blocked in TBST (Tris-buffered saline, 0.1% Tween 20), 5% nonfat dry milk for 30 min, followed by incubation in primary antibody overnight (antibody list above). Membranes were washed in TBST before incubation for 1 h with horseradish peroxidase-conjugated secondary antibodies (Pierce, NA934V rabbit, NXA931 mouse). Membranes were washed and developed using enhanced chemiluminescence substrate. The experiment was repeated 3 independent times in the lab.

Validation of syntaxin interactions (Figure 2):

STX3-LPXN and STX4-MAPK3 were validated using confocal microscopy in a Human Multiple Myeloma cell line U266. Cell lines were purchased from ATCC and routinely tested and found to be clear from any contamination. Cytospin preparations were prepared by centrifugation of 100 μL of U266 human B cell suspension (1 × 106 cells/ml) on polysine-coated slides at 1000 rpm for 3 min in a Rotofix 32 Hettich-Cyto-System. The slides were air dried and fixed in cold acetone at -20 for 15 mins. After a 5 mins PBS wash, the background staining was blocked for 1 hr at room temperature with 100 mM PBS-Glycine, 1.2 % Cold Fish Gelatin. The slides were then incubated overnight in a humidified chamber with primary antibodies (LPXN,

ab67571, Abcam; STX3, cat.No.110-033 Synaptic Systems; STX4, SC14454 Santacruz; MAPK3, ab884820 Abcam) diluted in the blocking buffer at 1/50.

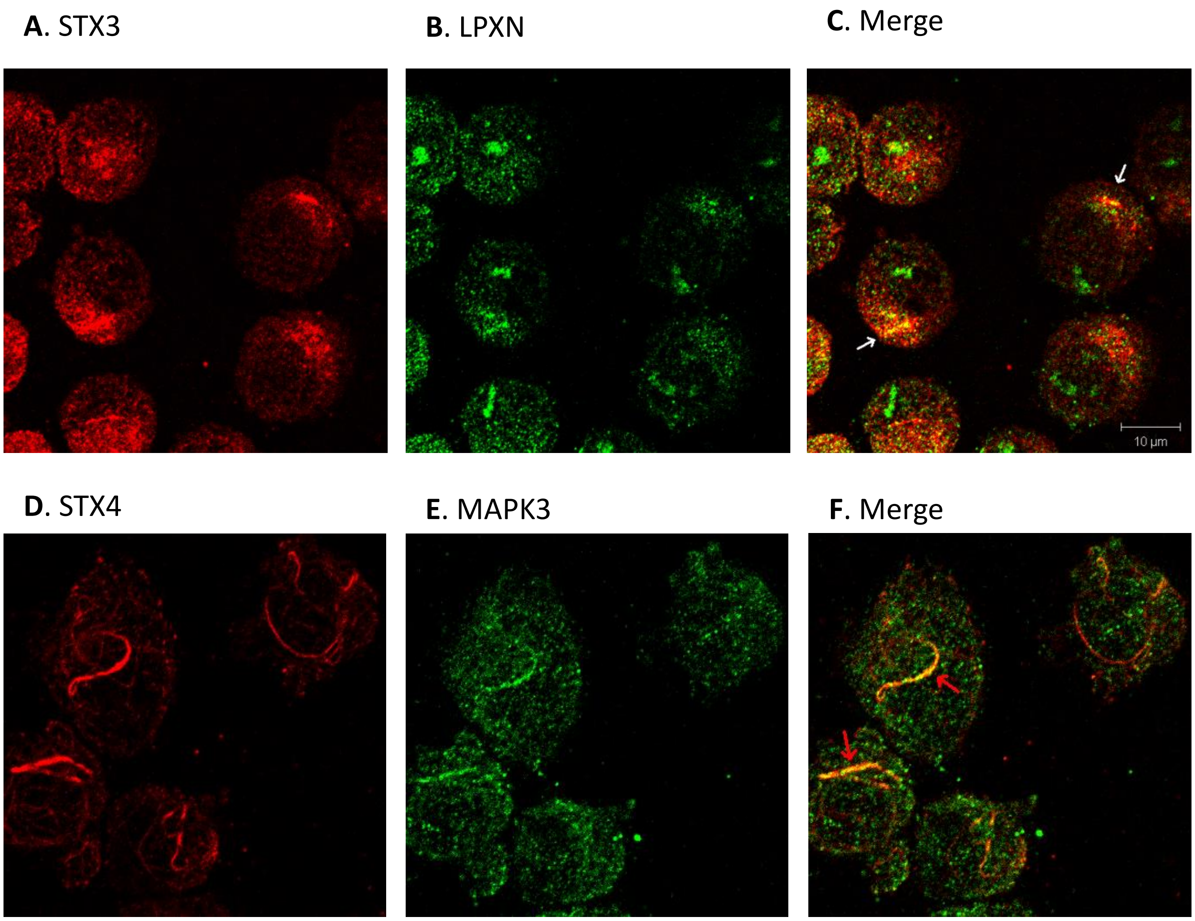

Figure 2: Validation of STX3-LPXN and STX4-MAPK3 interactions.

. The next day, slides were washed 3 × 5 mins in PBS-baths prior to 1 hr incubation at 37 C in the dark with Alexa Fluor 546® donkey anti-rabbit IgG and Alexa Fluor 488® donkey anti-mouse (Molecular Probes, Invitrogen). For double staining the slides were incubated with a mixture of primary antibodies, and the fluorochrome labelled conjugates were also mixed. Following rinsing 3 × 5 min in PBS-baths, the slides were coverslipped with antifade medium

(Dako). For negative controls, the primary antibodies were omitted and Alexa Fluor secondary antibodies were used as primary antibodies. None of these showed staining of any cells. Slide preparations were observed using a Zeiss Axio Observer. Z1 equipped with a Zeiss 710 and ConfoCor 3 laser scanning confocal head (Carl Zeiss, Germany). Colocalization signals were analyzed using ZEN 2008 software. The experiment was repeated two times in the lab.

Pathway analysis

Pathway associations of schizophrenia genes and the interactome were carried out separately by analyzing the corresponding gene sets with Ingenuity Pathway Analysis (IPA) IPA (Ingenuity® Systems, www.ingenuity.com).

Drug Ranking by Significance Values

We ranked drugs that target the genes in the schizophrenia interactome using Fisher's exact test. We compared the number of schizophrenia interactome genes that a drug targets compared to the total number of genes that same drug targets. Fisher's test computes the significance of that overlap as a p-value, which was further filtered using Benjamini-Hochberg correction for multiple testing. The drugs that have p-value < 0.05 are shown at the end of Supplementary File 9.
