# Supplementary material for: Schizophrenia interactome with 504 novel protein–protein interactions
Source: NPJ Schizophr. 2016 Apr 27;2:16012–. doi: 10.1038/npjschz.2016.12 (PMC4898894; doi:10.1038/npjschz.2016.12)
Supplement: Supplementary File 2 [file npjschz201612-s2.pdf]

GWAS

Symbol	Entrez ID	sources	Known PPIs	Novel PPIs
CACNA1I	8911	Nature	0	11
CNNM2	54805	Nature	0	6
APOPT1	84334	Nature	0	5
CENPM	79019	Nature	0	5
MIR137HG	400765	Nature	0	5
PLCH2	9651	Nature	0	4
GID4	79018	Nature	0	3
SDCCAG8	10806	Nature	0	3
ZNF536	9745	Nature	0	3
C10orf32	119032	Nature	0	2
C11orf31	280636	Nature	0	2
IGSF9B	22997	Nature	0	2
TSNARE1	203062	Nature	0	2
TYW5	129450	Nature	0	2
CSMD1	64478	Nature	0	1
LOC642484	642484	Nature	0	1
SOX2-OT	347689	Nature	0	1
C10orf32-ASMT	100528007	Nature	0	0
C3orf49	132200	Nature	0	0
EP300-AS1	101927279	Nature	0	0
GRAMD1B	57476	Nature	0	0
LINC01470	101927134	Nature	0	0
LOC100507431	100507431	Nature	0	0
TMX2-CTNND1	100528016	Nature	0	0
CHRNA5	1138	Nature	1	5
CYP26B1	56603	Nature	1	4
DGKI	9162	Nature	1	2
MAN2A1	4124	Nature	1	2
TMTC1	83857	Nature	1	2
IMMP2L	83943	Nature	1	1
DPYD	1806	Nature	2	8
ZNF804A	91752	Nature	2	8
CACNB2	783	Nature	2	7
PRRG2	5639	Nature	2	7
CNTN4	152330	Nature	2	5
NLGN4X	57502	Nature	2	5
CHRNA3	1136	Nature	2	4
GALNT10	55568	Nature	2	4
GPM6A	2823	Nature	2	4
EPC2	26122	Nature	2	3
HCN1	348980	Nature	2	3
SMG6	23293	Nature	3	8
PTGIS	5740	Nature	3	7
MPHOSPH9	10198	Nature	4	15

CLCN3	1182	Nature	4	11
KDM3B	51780	Nature	4	8
OTUD7B	56957	Nature	4	4
RGS6	9628	Nature	4	2
SATB2	23314	Nature	4	2
ETF1	2107	Nature	5	5
GRM3	2913	Historical, Nature	6	9
SHMT2	6472	Nature	6	9
ADAMTSL3	57188	Nature	6	7
AMBRA1	55626	Nature	6	7
NFATC3	4775	Nature	6	7
STAG1	10274	Nature	6	7
GIGYF2	26058	Nature	6	6
NRGN	4900	Nature	6	3
TCF20	6942	Nature	8	6
AKT3	10000	Nature	8	4
SNAP91	9892	Nature	8	3
ATP2A2	488	Nature	9	3
BCL11B	64919	Nature	9	3
VRK2	7444	Nature	10	6
CACNA1C	775	Nature	11	3
PPP1R16B	26051	Nature	12	8
PAK6	56924	Nature	13	8
RERE	473	Nature	20	7
GRIN2A	2903	Nature	27	8
MPP6	51678	Nature	28	6
FES	2242	Nature	31	10
PRKD1	5587	Nature	31	4
NAB2	4665	Nature	33	7
MAD1L1	8379	Nature	38	15
CUL3	8452	Nature	56	6
TCF4	6925	Nature	174	9
SRPK2	6733	Nature	177	5

Historical

Symbol	Entrez Id	Source	Known PPIs	New PPIs
SCZD10	63944	OMIM	0	10
CHI3L1	1116	OMIM	0	9
SCZD6	8400	OMIM	0	9
SCZD1	6377	OMIM	0	8
SCZD7	8401	OMIM	0	8
PRODH	5625	Historical, OMIM	0	7
SCZD3	6365	OMIM	0	7
ZDHHC8	29801	Historical	0	5
SCZD2	6378	OMIM	0	5
SCZD12	619488	OMIM	0	3
SCZD14	100196913	OMIM	0	2
SCZD8	8806	OMIM	0	2
TAAR6	319100	OMIM	0	1
DISC2	27184	OMIM	0	0
SCZD11	404686	OMIM	0	0
SCZD13	100329170	OMIM	0	0
MTHFR	4524	Historical, OMIM	1	7
APOL2	23780	OMIM	1	4
APOL4	80832	OMIM	1	3
DAOA	267012	Historical, OMIM	1	2
KCNN3	3782	Historical	2	5
VIPR2	7434	OMIM	4	6
CHRNA7	1139	Historical, OMIM	4	2
SLC6A4	6532	Historical	4	1
DAO	1610	Historical, OMIM	5	5
RTN4R	65078	OMIM	6	11
GRM3	2913	Historical, Nature	6	9
SLC1A1	6505	OMIM	6	8
DRD4	1815	Historical	6	4
SYN2	6854	OMIM	6	4
NRG1	3084	Historical, OMIM	8	8
SLC6A3	6531	Historical	8	2
COMT	1312	Historical, OMIM	10	5
PPP3CC	5533	Historical	11	5
NOTCH4	4855	Historical	13	9
RGS4	5999	Historical	13	8
BDNF	627	Historical	14	7
DRD3	1814	Historical, OMIM	15	3
SHANK3	85358	OMIM	15	3
HTR2A	3356	Historical, OMIM	16	6
DRD2	1813	Historical	21	13
TNF	7124	Historical	21	8
NRXN1	9378	OMIM	28	9
DTNBP1	84062	Historical, OMIM	53	8

APOE	348	Historical	54	7
DISC1	27185	Historical, OMIM	113	6
AKT1	207	Historical, OMIM	193	5

GWAS Interactome Genes

Gene	Label
ADAMTSL3	Candidate gene
AKT3	Candidate gene
AMBRA1	Candidate gene
APOPT1	Candidate gene
ATP2A2	Candidate gene
BCL11B	Candidate gene
C10orf32	Candidate gene
C10orf32-ASMT	Candidate gene
C11orf31	Candidate gene
C3orf49	Candidate gene
CACNA1C	Candidate gene
CACNA1I	Candidate gene
CACNB2	Candidate gene
CENPM	Candidate gene
CHRNA3	Candidate gene
CHRNA5	Candidate gene
CLCN3	Candidate gene
CNNM2	Candidate gene
CNTN4	Candidate gene
CSMD1	Candidate gene
CUL3	Candidate gene
CYP26B1	Candidate gene
DGKI	Candidate gene
DPYD	Candidate gene
EP300-AS1	Candidate gene
EPC2	Candidate gene
ETF1	Candidate gene
FES	Candidate gene
FLJ45743	Candidate gene
GALNT10	Candidate gene
GID4	Candidate gene
GIGYF2	Candidate gene
GPM6A	Candidate gene
GRAMD1B	Candidate gene
GRIN2A	Candidate gene
GRM3	Candidate gene
HCN1	Candidate gene
IGSF9B	Candidate gene
IMMP2L	Candidate gene
KDM3B	Candidate gene
LINC0147	Candidate gene
LOC100507431	Candidate gene
MAD1L1	Candidate gene
MAN2A1	Candidate gene

MIR137HG	Candidate gene
MPHOSPH9	Candidate gene
MPP6	Candidate gene
NAB2	Candidate gene
NFATC3	Candidate gene
NLGN4X	Candidate gene
NRGN	Candidate gene
OTUD7B	Candidate gene
PAK6	Candidate gene
PLCH2	Candidate gene
PPP1R16B	Candidate gene
PRKD1	Candidate gene
PRRG2	Candidate gene
PTGIS	Candidate gene
RERE	Candidate gene
RGS6	Candidate gene
SATB2	Candidate gene
SDCCAG8	Candidate gene
SHMT2	Candidate gene
SMG6	Candidate gene
SNAP91	Candidate gene
SOX2-OT	Candidate gene
SRPK2	Candidate gene
STAG1	Candidate gene
TCF20	Candidate gene
TCF4	Candidate gene
TMTC1	Candidate gene
TMX2-CTNND1	Candidate gene
TSNARE1	Candidate gene
TYW5	Candidate gene
VRK2	Candidate gene
ZNF536	Candidate gene
ZNF804A	Candidate gene
AATF	Known Interactor
ABCA1	Known Interactor
ABL1	Known Interactor
ABTB1	Known Interactor
ABTB2	Known Interactor
ACIN1	Known Interactor
ACTN1	Known Interactor
ADAP1	Known Interactor
ADPRH	Known Interactor
AES	Known Interactor
AHCYL1	Known Interactor
AKAP13	Known Interactor
AKT1	Known Interactor
ALG13	Known Interactor

ALKBH3	Known Interactor
ALOX5	Known Interactor
AMMECR1L	Known Interactor
AMOTL2	Known Interactor
AP1M1	Known Interactor
AP2A2	Known Interactor
AP2B1	Known Interactor
AP4M1	Known Interactor
APC	Known Interactor
APEX1	Known Interactor
APOBEC3C	Known Interactor
APP	Known Interactor
AQP1	Known Interactor
AR	Known Interactor
ARGLU1	Known Interactor
ARHGAP12	Known Interactor
ARHGAP18	Known Interactor
ARHGAP32	Known Interactor
ARHGEF1	Known Interactor
ARHGEF12	Known Interactor
ARL6IP1	Known Interactor
ARL6IP4	Known Interactor
ARMC7	Known Interactor
ARPC1B	Known Interactor
ASCL1	Known Interactor
ASCL3	Known Interactor
ASCL4	Known Interactor
ASPSCR1	Known Interactor
ATAT1	Known Interactor
ATN1	Known Interactor
ATP6V1B2	Known Interactor
ATXN1	Known Interactor
AURKA	Known Interactor
BAG5	Known Interactor
BAIAP2	Known Interactor
BARD1	Known Interactor
BCAR1	Known Interactor
BCAS2	Known Interactor
BCL2	Known Interactor
BCL2L2	Known Interactor
BCR	Known Interactor
BECN1	Known Interactor
BRCA1	Known Interactor
BRD3	Known Interactor
BTBD10	Known Interactor
BTK	Known Interactor
BZRAP1	Known Interactor

C16orf78	Known Interactor
C17orf85	Known Interactor
C18orf25	Known Interactor
C19orf66	Known Interactor
C1QBP	Known Interactor
C1orf109	Known Interactor
C1orf35	Known Interactor
C4orf46	Known Interactor
C9orf171	Known Interactor
CABP1	Known Interactor
CABP5	Known Interactor
CACNB3	Known Interactor
CACNG2	Known Interactor
CALM1	Known Interactor
CALM2	Known Interactor
CALM3	Known Interactor
CAMK1D	Known Interactor
CAMK2A	Known Interactor
CAMK2B	Known Interactor
CAND1	Known Interactor
CASK	Known Interactor
CBFA2T2	Known Interactor
CBLN4	Known Interactor
CBX5	Known Interactor
CCDC101	Known Interactor
CCDC28A	Known Interactor
CCDC33	Known Interactor
CCDC94	Known Interactor
CCDC97	Known Interactor
CCHCR1	Known Interactor
CCM2	Known Interactor
CCND1	Known Interactor
CCNE1	Known Interactor
CCT2	Known Interactor
CCT4	Known Interactor
CDC25A	Known Interactor
CDC34	Known Interactor
CDC42	Known Interactor
CDC42EP4	Known Interactor
CDC73	Known Interactor
CDCA7L	Known Interactor
CDK1	Known Interactor
CDK5	Known Interactor
CDK6	Known Interactor
CDK7	Known Interactor
CDKN1A	Known Interactor
CDKN2C	Known Interactor

CEP55	Known Interactor
CEP70	Known Interactor
CFTR	Known Interactor
CHAF1A	Known Interactor
CHCHD2	Known Interactor
CHD2	Known Interactor
CHERP	Known Interactor
CHIC2	Known Interactor
CHRNB4	Known Interactor
CHTOP	Known Interactor
CHUK	Known Interactor
CKS1B	Known Interactor
CLDN19	Known Interactor
CLK1	Known Interactor
CLK2	Known Interactor
CLK3	Known Interactor
CMTM5	Known Interactor
COIL	Known Interactor
COMMD1	Known Interactor
COPS2	Known Interactor
COPS5	Known Interactor
COPS7A	Known Interactor
CREBBP	Known Interactor
CRX	Known Interactor
CSAG1	Known Interactor
CSF2RB	Known Interactor
CSNK1A1	Known Interactor
CSNK2B	Known Interactor
CTBP1	Known Interactor
CTNNB1	Known Interactor
CTNNBL1	Known Interactor
CWF19L2	Known Interactor
DAW1	Known Interactor
DBF4B	Known Interactor
DCUN1D1	Known Interactor
DCUN1D2	Known Interactor
DCUN1D3	Known Interactor
DCUN1D4	Known Interactor
DCUN1D5	Known Interactor
DDX21	Known Interactor
DDX46	Known Interactor
DDX6	Known Interactor
DEF6	Known Interactor
DGCR6	Known Interactor
DHX8	Known Interactor
DIS3	Known Interactor
DLG1	Known Interactor

DLG2	Known Interactor
DLG3	Known Interactor
DLG4	Known Interactor
DLGAP1	Known Interactor
DLGAP2	Known Interactor
DMAP1	Known Interactor
DMRT3	Known Interactor
DNAJC8	Known Interactor
DNM2	Known Interactor
DNMT1	Known Interactor
DOK1	Known Interactor
DPYSL5	Known Interactor
DRAP1	Known Interactor
DRG1	Known Interactor
DYNLL1	Known Interactor
DYNLRB1	Known Interactor
DYRK4	Known Interactor
ECM1	Known Interactor
EED	Known Interactor
EFEMP1	Known Interactor
EFEMP2	Known Interactor
EFHC1	Known Interactor
EFNB1	Known Interactor
EGFR	Known Interactor
EGLN2	Known Interactor
EGR1	Known Interactor
EGR2	Known Interactor
EHMT2	Known Interactor
EIF1AY	Known Interactor
EIF3G	Known Interactor
EIF4E2	Known Interactor
EIF4EBP1	Known Interactor
EIF5	Known Interactor
ENC1	Known Interactor
ENOX2	Known Interactor
EP300	Known Interactor
EPAS1	Known Interactor
EPB41L1	Known Interactor
EPB41L3	Known Interactor
EPB41L4A	Known Interactor
EPHB6	Known Interactor
EPS15	Known Interactor
EPS8L3	Known Interactor
ERCC3	Known Interactor
ERN1	Known Interactor
ESR1	Known Interactor
EVI5L	Known Interactor

EXOSC1	Known Interactor
EXOSC10	Known Interactor
EXOSC2	Known Interactor
EXOSC4	Known Interactor
EXOSC5	Known Interactor
EXOSC6	Known Interactor
EXOSC7	Known Interactor
EXOSC8	Known Interactor
EXOSC9	Known Interactor
EZH2	Known Interactor
FAH	Known Interactor
FAM124B	Known Interactor
FAM131C	Known Interactor
FAM208B	Known Interactor
FAM74A4	Known Interactor
FAM76B	Known Interactor
FAM90A1	Known Interactor
FATE1	Known Interactor
FERD3L	Known Interactor
FGF12	Known Interactor
FKBP4	Known Interactor
FKBP5	Known Interactor
FLAD1	Known Interactor
FLNA	Known Interactor
FOLR1	Known Interactor
FOS	Known Interactor
FOXN3	Known Interactor
FRS3	Known Interactor
FTL	Known Interactor
FXR2	Known Interactor
FYN	Known Interactor
GABARAP	Known Interactor
GABARAPL1	Known Interactor
GAN	Known Interactor
GCC1	Known Interactor
GEM	Known Interactor
GLP1R	Known Interactor
GLRX3	Known Interactor
GMCL1	Known Interactor
GNB1	Known Interactor
GNB2L1	Known Interactor
GNB5	Known Interactor
GNG10	Known Interactor
GOLGA2	Known Interactor
GOLGA8EP	Known Interactor
GOLGA8F	Known Interactor
GOPC	Known Interactor

GORASP2	Known Interactor
GPR114	Known Interactor
GPS1	Known Interactor
GRASP	Known Interactor
GRB10	Known Interactor
GRIN1	Known Interactor
GRIN3A	Known Interactor
GRIN3B	Known Interactor
GRIP1	Known Interactor
GSK3B	Known Interactor
GSPT1	Known Interactor
GSPT2	Known Interactor
GTF2E1	Known Interactor
GTF3C5	Known Interactor
GUCD1	Known Interactor
HAND2	Known Interactor
HAT1	Known Interactor
HAUS1	Known Interactor
HBP1	Known Interactor
HBS1L	Known Interactor
HCN2	Known Interactor
HDAC1	Known Interactor
HDAC2	Known Interactor
HDAC4	Known Interactor
HDAC5	Known Interactor
HDAC7	Known Interactor
HGS	Known Interactor
HIC1	Known Interactor
HIRIP3	Known Interactor
HIST2H2AC	Known Interactor
HIST2H2BE	Known Interactor
HIST2H3C	Known Interactor
HIST3H3	Known Interactor
HLA-DMB	Known Interactor
HMGN3	Known Interactor
HNRNPC	Known Interactor
HNRNPD	Known Interactor
HOMER3	Known Interactor
HSF2	Known Interactor
HSH2D	Known Interactor
HSPA4	Known Interactor
HSPB1	Known Interactor
HSPB7	Known Interactor
IBTK	Known Interactor
ID1	Known Interactor
ID2	Known Interactor
ID3	Known Interactor

ID4	Known Interactor
IFIT5	Known Interactor
IGF1R	Known Interactor
IKZF3	Known Interactor
IL16	Known Interactor
IL4R	Known Interactor
INADL	Known Interactor
INS	Known Interactor
IRS1	Known Interactor
IRS2	Known Interactor
JAK1	Known Interactor
JAK2	Known Interactor
JAK3	Known Interactor
JAKMIP2	Known Interactor
JUN	Known Interactor
JUP	Known Interactor
KAT6A	Known Interactor
KCNN2	Known Interactor
KCTD13	Known Interactor
KCTD14	Known Interactor
KCTD5	Known Interactor
KCTD6	Known Interactor
KCTD9	Known Interactor
KEAP1	Known Interactor
KHDRBS1	Known Interactor
KIDINS220	Known Interactor
KLC3	Known Interactor
KLHL12	Known Interactor
KLHL2	Known Interactor
KLHL20	Known Interactor
KLHL3	Known Interactor
KLHL32	Known Interactor
KLHL41	Known Interactor
KLHL42	Known Interactor
KLHL5	Known Interactor
KNSTRN	Known Interactor
KRT40	Known Interactor
KRTAP10-3	Known Interactor
KRTAP10-8	Known Interactor
KRTAP2-4	Known Interactor
KRTAP4-12	Known Interactor
LASP1	Known Interactor
LCA5	Known Interactor
LCE3D	Known Interactor
LENG1	Known Interactor
LGALS14	Known Interactor
LGALSL	Known Interactor

LIG4	Known Interactor
LIN7A	Known Interactor
LMO1	Known Interactor
LMO4	Known Interactor
LNPEP	Known Interactor
LNX1	Known Interactor
LRRN4CL	Known Interactor
LUC7L	Known Interactor
LUC7L2	Known Interactor
LUC7L3	Known Interactor
LUZP4	Known Interactor
LXN	Known Interactor
LYL1	Known Interactor
LZTR1	Known Interactor
MAB21L2	Known Interactor
MAD2L1	Known Interactor
MAD2L2	Known Interactor
MAGEB1	Known Interactor
MAGOHB	Known Interactor
MAP2K7	Known Interactor
MAP3K7	Known Interactor
MAPK14	Known Interactor
MAPK3	Known Interactor
MAPK8	Known Interactor
MAPK8IP1	Known Interactor
MAPK8IP2	Known Interactor
MAPK9	Known Interactor
MAPKAPK5	Known Interactor
MAPKBP1	Known Interactor
10-Mar	Known Interactor
MATN3	Known Interactor
MAX	Known Interactor
MBD3	Known Interactor
MCM7	Known Interactor
MCOLN3	Known Interactor
MDFI	Known Interactor
MDK	Known Interactor
MDM2	Known Interactor
MEMO1	Known Interactor
MEN1	Known Interactor
MFAP1	Known Interactor
MLLT6	Known Interactor
MLX	Known Interactor
MORF4L1	Known Interactor
MORN4	Known Interactor
MPP3	Known Interactor
MRFAP1L1	Known Interactor

MRPL10	Known Interactor
MRPS6	Known Interactor
MSC	Known Interactor
MSRB3	Known Interactor
MT2A	Known Interactor
MT2P1	Known Interactor
MTA2	Known Interactor
MTOR	Known Interactor
MUC1	Known Interactor
MUC7	Known Interactor
MVP	Known Interactor
MYO5B	Known Interactor
MYOD1	Known Interactor
NCK2	Known Interactor
NDC80	Known Interactor
NDN	Known Interactor
NDOR1	Known Interactor
NDRG1	Known Interactor
NEBL	Known Interactor
NECAP1	Known Interactor
NEDD4	Known Interactor
NEDD4L	Known Interactor
NEDD8	Known Interactor
NEDD9	Known Interactor
NEFL	Known Interactor
NEK2	Known Interactor
NEK6	Known Interactor
NEK8	Known Interactor
NELFE	Known Interactor
NEU4	Known Interactor
NEUROD1	Known Interactor
NEUROG1	Known Interactor
NFKB1	Known Interactor
NGLY1	Known Interactor
NINL	Known Interactor
NME7	Known Interactor
NMUR2	Known Interactor
NOL9	Known Interactor
NONO	Known Interactor
NOP16	Known Interactor
NOTCH2NL	Known Interactor
NPM2	Known Interactor
NR2C2AP	Known Interactor
NR2E1	Known Interactor
NR2F1	Known Interactor
NR2F2	Known Interactor
NR4A1	Known Interactor

NSMCE4A	Known Interactor
NSRP1	Known Interactor
NUDT10	Known Interactor
NUP50	Known Interactor
NXT2	Known Interactor
OCEL1	Known Interactor
OGT	Known Interactor
OLIG2	Known Interactor
ORC4	Known Interactor
OSGIN1	Known Interactor
OTUD4	Known Interactor
PABPC4	Known Interactor
PAK4	Known Interactor
PAM16	Known Interactor
PARK2	Known Interactor
PARN	Known Interactor
PARP1	Known Interactor
PATE1	Known Interactor
PBLD	Known Interactor
PCBD1	Known Interactor
PDE4DIP	Known Interactor
PDE7B	Known Interactor
PDIA2	Known Interactor
PDPK1	Known Interactor
PDZK1	Known Interactor
PELI1	Known Interactor
PHC2	Known Interactor
PHF1	Known Interactor
PHOSPHO2	Known Interactor
PIAS1	Known Interactor
PICK1	Known Interactor
PIK3R1	Known Interactor
PIN1	Known Interactor
PLCG1	Known Interactor
PLCG2	Known Interactor
PLEKHN1	Known Interactor
PLN	Known Interactor
PLSCR1	Known Interactor
PLXNA1	Known Interactor
POLB	Known Interactor
POLR1A	Known Interactor
POLR1C	Known Interactor
POT1	Known Interactor
PPARD	Known Interactor
PPIA	Known Interactor
PPID	Known Interactor
PPIL1	Known Interactor

PPM1A	Known Interactor
PPP1CC	Known Interactor
PPP1R14A	Known Interactor
PPP1R18	Known Interactor
PPP2CA	Known Interactor
PRKAA1	Known Interactor
PRKAA2	Known Interactor
PRKAB2	Known Interactor
PRKACA	Known Interactor
PRKCA	Known Interactor
PRKCB	Known Interactor
PRKCD	Known Interactor
PRKCE	Known Interactor
PRKCG	Known Interactor
PRKCH	Known Interactor
PRKCZ	Known Interactor
PRPF38A	Known Interactor
PRPF40A	Known Interactor
PRRC2A	Known Interactor
PRRC2B	Known Interactor
PSEN1	Known Interactor
PSMA1	Known Interactor
PSMA3	Known Interactor
PSMG2	Known Interactor
PTBP1	Known Interactor
PTF1A	Known Interactor
PTGER3	Known Interactor
PTGES3	Known Interactor
PTGS1	Known Interactor
PTGS2	Known Interactor
PTK2B	Known Interactor
PTPN4	Known Interactor
RAB3IL1	Known Interactor
RAB41	Known Interactor
RABIF	Known Interactor
RAD21	Known Interactor
RASA1	Known Interactor
RASA3	Known Interactor
RBBP4	Known Interactor
RBBP7	Known Interactor
RBFOX2	Known Interactor
RBM12	Known Interactor
RBM15	Known Interactor
RBM23	Known Interactor
RBM39	Known Interactor
RBM8A	Known Interactor
RBPMS	Known Interactor

RBX1	Known Interactor
RCBTB1	Known Interactor
RCBTB2	Known Interactor
RECK	Known Interactor
RELA	Known Interactor
REM1	Known Interactor
REXO1L6P	Known Interactor
RFC5	Known Interactor
RGS3	Known Interactor
RHOBTB3	Known Interactor
RHOJ	Known Interactor
RIMS1	Known Interactor
RIT1	Known Interactor
RIT2	Known Interactor
RNF138	Known Interactor
RNF2	Known Interactor
RNF4	Known Interactor
RNF7	Known Interactor
RNF8	Known Interactor
RNPS1	Known Interactor
RPL22	Known Interactor
RPL39L	Known Interactor
RPL41	Known Interactor
RPS20	Known Interactor
RQCD1	Known Interactor
RSPH14	Known Interactor
RSRC1	Known Interactor
RSRC2	Known Interactor
RSRP1	Known Interactor
RTF1	Known Interactor
RUNX1T1	Known Interactor
RWDD2B	Known Interactor
RYR2	Known Interactor
S100A1	Known Interactor
SCNM1	Known Interactor
SCOC	Known Interactor
SDCBP	Known Interactor
SDR42E1	Known Interactor
SEC14L4	Known Interactor
SEC23B	Known Interactor
SEMA3B	Known Interactor
SF3B4	Known Interactor
SHKBP1	Known Interactor
SIGLEC6	Known Interactor
SIN3A	Known Interactor
SIN3B	Known Interactor
SKIV2L2	Known Interactor

SLAIN2	Known Interactor
SLC39A13	Known Interactor
SLC4A1AP	Known Interactor
SLC9A3R1	Known Interactor
SMAD1	Known Interactor
SMAD2	Known Interactor
SMAD9	Known Interactor
SMARCA4	Known Interactor
SMCP	Known Interactor
SMG5	Known Interactor
SMOC1	Known Interactor
SNIP1	Known Interactor
SNRNP27	Known Interactor
SNRNP35	Known Interactor
SNRNP70	Known Interactor
SNRPB	Known Interactor
SNURF	Known Interactor
SNX2	Known Interactor
SNX9	Known Interactor
SP1	Known Interactor
SPAG8	Known Interactor
SPATA2	Known Interactor
SPATC1L	Known Interactor
SPG21	Known Interactor
SPP1	Known Interactor
SPTAN1	Known Interactor
SRC	Known Interactor
SREK1	Known Interactor
SRI	Known Interactor
SRRM1	Known Interactor
SRSF1	Known Interactor
SRSF12	Known Interactor
SRSF2	Known Interactor
SRSF3	Known Interactor
SRSF5	Known Interactor
SRSF7	Known Interactor
SRSF8	Known Interactor
SRSF9	Known Interactor
STAG2	Known Interactor
STAMBPL1	Known Interactor
STAT3	Known Interactor
STK16	Known Interactor
STMN2	Known Interactor
STON1	Known Interactor
STX11	Known Interactor
SUMO1	Known Interactor
SUMO1P1	Known Interactor

SUMO2	Known Interactor
SUMO3	Known Interactor
SUPT16H	Known Interactor
SUV39H1	Known Interactor
SVIL	Known Interactor
SYK	Known Interactor
SZT2	Known Interactor
TADA2A	Known Interactor
TAF7L	Known Interactor
TAL1	Known Interactor
TAL2	Known Interactor
TAPBPL	Known Interactor
TCEA2	Known Interactor
TCEANC	Known Interactor
TCEB1	Known Interactor
TCF3	Known Interactor
TCHP	Known Interactor
TCL1A	Known Interactor
TCOF1	Known Interactor
TCP1	Known Interactor
TDP2	Known Interactor
TERF1	Known Interactor
TERT	Known Interactor
TEX11	Known Interactor
TGIF2LY	Known Interactor
THOP1	Known Interactor
TLE1	Known Interactor
TLE4	Known Interactor
TMEM213	Known Interactor
TNFAIP1	Known Interactor
TNFRSF10C	Known Interactor
TNIP2	Known Interactor
TP53	Known Interactor
TPD52	Known Interactor
TPD52L1	Known Interactor
TPM1	Known Interactor
TPM3	Known Interactor
TPR	Known Interactor
TRA2A	Known Interactor
TRA2B	Known Interactor
TRAF3IP1	Known Interactor
TRAF6	Known Interactor
TRAPPC2L	Known Interactor
TRIM22	Known Interactor
TRIM28	Known Interactor
TRIM29	Known Interactor
TRIM41	Known Interactor

TRIP6	Known Interactor
TSC2	Known Interactor
TSC22D3	Known Interactor
TSHZ3	Known Interactor
TSSC4	Known Interactor
TSSK3	Known Interactor
TTC19	Known Interactor
TTF1	Known Interactor
TTN	Known Interactor
TUBA1B	Known Interactor
TUBGCP4	Known Interactor
TWIST1	Known Interactor
TWIST2	Known Interactor
TXNL4B	Known Interactor
U2AF1	Known Interactor
U2AF2	Known Interactor
UBC	Known Interactor
UBD	Known Interactor
UBE2D1	Known Interactor
UBE2D2	Known Interactor
UBE2D3	Known Interactor
UBE2E1	Known Interactor
UBE2E2	Known Interactor
UBE2E3	Known Interactor
UBE2M	Known Interactor
UBE2S	Known Interactor
UBQLN1	Known Interactor
UBTF	Known Interactor
USP15	Known Interactor
USP4	Known Interactor
UTP23	Known Interactor
VAV2	Known Interactor
VHL	Known Interactor
VIMP	Known Interactor
VPS28	Known Interactor
VRK1	Known Interactor
WBP11	Known Interactor
WDR5	Known Interactor
WFDC5	Known Interactor
WNT7B	Known Interactor
XRCC5	Known Interactor
XRCC6	Known Interactor
YPEL2	Known Interactor
YPEL3	Known Interactor
YTHDC1	Known Interactor
YWHAB	Known Interactor
YWHAG	Known Interactor

YWHAQ	Known Interactor
YWHAZ	Known Interactor
ZBED8	Known Interactor
ZDHHC17	Known Interactor
ZDHHC24	Known Interactor
ZMAT4	Known Interactor
ZMYND19	Known Interactor
ZMYND8	Known Interactor
ZNF124	Known Interactor
ZNF205-AS1	Known Interactor
ZNF417	Known Interactor
ZNF512B	Known Interactor
ZNF587	Known Interactor
ZNF746	Known Interactor
ZNF775	Known Interactor
ZRANB2	Known Interactor
ZRSR2	Known Interactor
ZSCAN32	Known Interactor
ZSCAN9	Known Interactor
ABI2	Novel Interactor
ACACA	Novel Interactor
ACOT8	Novel Interactor
ACTR1A	Novel Interactor
ACVR1B	Novel Interactor
ADAM23	Novel Interactor
ADAMTS8	Novel Interactor
AGFG1	Novel Interactor
AHNAK2	Novel Interactor
AIMP2	Novel Interactor
ALDH1B1	Novel Interactor
ALDH6A1	Novel Interactor
ALG14	Novel Interactor
ALPK3	Novel Interactor
ALPP	Novel Interactor
ALX4	Novel Interactor
ANXA10	Novel Interactor
ANXA11	Novel Interactor
ANXA2R	Novel Interactor
ANXA5	Novel Interactor
APH1B	Novel Interactor
APOA5	Novel Interactor
APOBEC1	Novel Interactor
APOL1	Novel Interactor
AREL1	Novel Interactor
ARF5	Novel Interactor
ARFGEF2	Novel Interactor
ARL4C	Novel Interactor

ARPC3	Novel Interactor
AS3MT	Novel Interactor
ASB3	Novel Interactor
ASIC2	Novel Interactor
ASPH	Novel Interactor
ATG16L1	Novel Interactor
ATP6V0A2	Novel Interactor
BEGAIN	Novel Interactor
BET1	Novel Interactor
BLM	Novel Interactor
BNC1	Novel Interactor
BRE	Novel Interactor
BTF3	Novel Interactor
BYSL	Novel Interactor
C10orf67	Novel Interactor
C10orf76	Novel Interactor
C10orf95	Novel Interactor
C14orf132	Novel Interactor
C15orf41	Novel Interactor
C19orf12	Novel Interactor
C1orf54	Novel Interactor
C22orf46	Novel Interactor
C4orf27	Novel Interactor
C5orf34	Novel Interactor
CA3	Novel Interactor
CAB39	Novel Interactor
CACNA1H	Novel Interactor
CALB2	Novel Interactor
CAMTA1	Novel Interactor
CARHSP1	Novel Interactor
CASC5	Novel Interactor
CASP3	Novel Interactor
CASR	Novel Interactor
CCDC134	Novel Interactor
CCDC91	Novel Interactor
CCL16	Novel Interactor
CCL22	Novel Interactor
CCL4	Novel Interactor
CCND2	Novel Interactor
CCNT2	Novel Interactor
CD9	Novel Interactor
CDC42SE1	Novel Interactor
CDK2AP1	Novel Interactor
CDK5RAP1	Novel Interactor
CDKL3	Novel Interactor
CDON	Novel Interactor
CEBPG	Novel Interactor

CELSR2	Novel Interactor
CEP104	Novel Interactor
CEP170	Novel Interactor
CEP44	Novel Interactor
CEP63	Novel Interactor
CHCHD3	Novel Interactor
CHST12	Novel Interactor
CLDN18	Novel Interactor
CLUH	Novel Interactor
CNTN5	Novel Interactor
CNTN6	Novel Interactor
COMMD6	Novel Interactor
CPB2	Novel Interactor
CRABP1	Novel Interactor
CREM	Novel Interactor
CSDE1	Novel Interactor
CSF2	Novel Interactor
CTIF	Novel Interactor
CTTNBP2	Novel Interactor
CXADR	Novel Interactor
CYP51A1	Novel Interactor
CYTH2	Novel Interactor
DECR1	Novel Interactor
DESI1	Novel Interactor
DGKH	Novel Interactor
DHDDS	Novel Interactor
DLL4	Novel Interactor
DNAJC10	Novel Interactor
DNAL4	Novel Interactor
DPCD	Novel Interactor
DUSP19	Novel Interactor
DYNAP	Novel Interactor
EFTUD1	Novel Interactor
EI24	Novel Interactor
ELAC2	Novel Interactor
EML1	Novel Interactor
EPB41L4A-AS1	Novel Interactor
EPHA2	Novel Interactor
ERAP2	Novel Interactor
ETFA	Novel Interactor
ETFDH	Novel Interactor
FAM103A1	Novel Interactor
FAM106A	Novel Interactor
FAM13B	Novel Interactor
FAM171B	Novel Interactor
FAM53C	Novel Interactor
FAM63A	Novel Interactor

FAM83D	Novel Interactor
FAP	Novel Interactor
FBLN1	Novel Interactor
FBXL15	Novel Interactor
FBXO42	Novel Interactor
FBXO8	Novel Interactor
FGL2	Novel Interactor
FSCN1	Novel Interactor
FURIN	Novel Interactor
FZD3	Novel Interactor
GABARAPL2	Novel Interactor
GEMIN5	Novel Interactor
GHRL	Novel Interactor
GJD2	Novel Interactor
GMFG	Novel Interactor
GNAS	Novel Interactor
GOLGA2P10	Novel Interactor
GPR123	Novel Interactor
GPR137B	Novel Interactor
GPR85	Novel Interactor
GPR88	Novel Interactor
GPRC5C	Novel Interactor
GRIA2	Novel Interactor
GSK3A	Novel Interactor
GTF2H2	Novel Interactor
GTPBP1	Novel Interactor
GUK1	Novel Interactor
GYPA	Novel Interactor
HCAR3	Novel Interactor
HDGFRP3	Novel Interactor
HMGXB4	Novel Interactor
HOXA10	Novel Interactor
HPCA	Novel Interactor
HRH1	Novel Interactor
HS3ST1	Novel Interactor
HSD17B12	Novel Interactor
IDH3A	Novel Interactor
IGHV5-78	Novel Interactor
INF2	Novel Interactor
INPP5K	Novel Interactor
JOSD1	Novel Interactor
KBTBD4	Novel Interactor
KCNJ3	Novel Interactor
KCNJ4	Novel Interactor
KIAA1715	Novel Interactor
KMT2D	Novel Interactor
KNTC1	Novel Interactor

KXD1	Novel Interactor
LAMP3	Novel Interactor
LCP1	Novel Interactor
LCP2	Novel Interactor
LIPG	Novel Interactor
LOC153684	Novel Interactor
LPPR5	Novel Interactor
LRP1	Novel Interactor
LRP2BP	Novel Interactor
LRRC48	Novel Interactor
LTB4R2	Novel Interactor
MAN2A2	Novel Interactor
MARS2	Novel Interactor
MCEE	Novel Interactor
MED23	Novel Interactor
METTL21A	Novel Interactor
MITF	Novel Interactor
MKRN2	Novel Interactor
MLEC	Novel Interactor
MMADHC	Novel Interactor
MRPL12	Novel Interactor
MRPL19	Novel Interactor
MSL2	Novel Interactor
MSRB2	Novel Interactor
MTCH2	Novel Interactor
MYL9	Novel Interactor
MYO1A	Novel Interactor
MYOCD	Novel Interactor
MYOZ2	Novel Interactor
NAT1	Novel Interactor
NCAPD3	Novel Interactor
NDUFA10	Novel Interactor
NEK1	Novel Interactor
NFX1	Novel Interactor
NKD2	Novel Interactor
NKG7	Novel Interactor
NMI	Novel Interactor
NMUR1	Novel Interactor
NRP1	Novel Interactor
NUP35	Novel Interactor
NXPH1	Novel Interactor
OCM2	Novel Interactor
OOSP2	Novel Interactor
OR10J1	Novel Interactor
OSBPL3	Novel Interactor
OVCH1	Novel Interactor
OVGP1	Novel Interactor

PAF1	Novel Interactor
PATL1	Novel Interactor
PCDH12	Novel Interactor
PCDH8	Novel Interactor
PCDHGB7	Novel Interactor
PCDHGC4	Novel Interactor
PCDHGC5	Novel Interactor
PCYOX1	Novel Interactor
PDGFB	Novel Interactor
PDIA3	Novel Interactor
PEX10	Novel Interactor
PEX11A	Novel Interactor
PGM3	Novel Interactor
PHF5A	Novel Interactor
PIAS2	Novel Interactor
PIN4	Novel Interactor
PKHD1	Novel Interactor
PLA2G10	Novel Interactor
PLA2G6	Novel Interactor
PLD3	Novel Interactor
PLEKHB2	Novel Interactor
POLR2I	Novel Interactor
PPP2R1A	Novel Interactor
PRICKLE4	Novel Interactor
PRKAG1	Novel Interactor
PRKAR1B	Novel Interactor
PRM1	Novel Interactor
PRO2964	Novel Interactor
PRPF8	Novel Interactor
PTEN	Novel Interactor
PTER	Novel Interactor
PTTG1	Novel Interactor
RAB35	Novel Interactor
RAB5C	Novel Interactor
RAD51AP1	Novel Interactor
RAP1GAP2	Novel Interactor
RDH5	Novel Interactor
REEP2	Novel Interactor
RGS1	Novel Interactor
RGS7	Novel Interactor
RIPPLY2	Novel Interactor
RND1	Novel Interactor
RNF40	Novel Interactor
RPA3	Novel Interactor
RPL18A	Novel Interactor
RPS6KA3	Novel Interactor
RTN4RL1	Novel Interactor

RWDD2A	Novel Interactor
S100P	Novel Interactor
SAP30L	Novel Interactor
SATB2-AS1	Novel Interactor
SCAF11	Novel Interactor
SCAMP2	Novel Interactor
SCN1A	Novel Interactor
SEC24A	Novel Interactor
SEMA7A	Novel Interactor
3-Sep	Novel Interactor
SETBP1	Novel Interactor
SFXN2	Novel Interactor
SGTA	Novel Interactor
SLC22A1	Novel Interactor
SLC35C1	Novel Interactor
SLC44A3	Novel Interactor
SMC1B	Novel Interactor
SMCO4	Novel Interactor
SNAI2	Novel Interactor
SNX15	Novel Interactor
SNX20	Novel Interactor
SP3	Novel Interactor
SPG11	Novel Interactor
SPINK7	Novel Interactor
SRA1	Novel Interactor
SSR4	Novel Interactor
ST5	Novel Interactor
STAM	Novel Interactor
STARD5	Novel Interactor
SULT2A1	Novel Interactor
SYNGR1	Novel Interactor
TACR3	Novel Interactor
TBC1D22A	Novel Interactor
TBCA	Novel Interactor
TDRD9	Novel Interactor
TMED10	Novel Interactor
TMEM56	Novel Interactor
TMSB4XP6	Novel Interactor
TNFAIP8L2	Novel Interactor
TOP1MT	Novel Interactor
TOR1B	Novel Interactor
TP73-AS1	Novel Interactor
TRAM1	Novel Interactor
TRIM4	Novel Interactor
TRMT61A	Novel Interactor
TST	Novel Interactor
TTI1	Novel Interactor

TTYH3	Novel Interactor
TUBA1C	Novel Interactor
TUFM	Novel Interactor
TULP4	Novel Interactor
UBE4B	Novel Interactor
UGT2B4	Novel Interactor
UNG	Novel Interactor
VANGL1	Novel Interactor
VDR	Novel Interactor
VPS18	Novel Interactor
WBP1L	Novel Interactor
WDR25	Novel Interactor
WFDC1	Novel Interactor
YY1	Novel Interactor
ZAP70	Novel Interactor
ZBTB17	Novel Interactor
ZC3H15	Novel Interactor
ZMYND11	Novel Interactor
ZNF117	Novel Interactor
ZNF219	Novel Interactor
ZNF239	Novel Interactor
ZNF707	Novel Interactor
ZNF804B	Novel Interactor
ZSCAN31	Novel Interactor
ZSWIM2	Novel Interactor

Historical Interactome Genes

Gene	Label
AKT1	Candidate gene
APOE	Candidate gene
BDNF	Candidate gene
CHRNA7	Candidate gene
COMT	Candidate gene
DAO	Candidate gene
DAOA	Candidate gene
DISC1	Candidate gene
DRD2	Candidate gene
DRD3	Candidate gene
DRD4	Candidate gene
DTNBP1	Candidate gene
GRM3	Candidate gene
HTR2A	Candidate gene
KCNN3	Candidate gene
MTHFR	Candidate gene
NOTCH4	Candidate gene
NRG1	Candidate gene
PPP3CC	Candidate gene
PRODH	Candidate gene
RGS4	Candidate gene
SLC6A3	Candidate gene
SLC6A4	Candidate gene
TNF	Candidate gene
ZDHHC8	Candidate gene
A2M	Known Interactor
ABI3	Known Interactor
ACAP1	Known Interactor
ACE	Known Interactor
ACTG1	Known Interactor
ACTN2	Known Interactor
ADAM17	Known Interactor
ADAM19	Known Interactor
ADAM9	Known Interactor
ADCY6	Known Interactor
ADORA2A	Known Interactor
AGO3	Known Interactor
AGTPBP1	Known Interactor
AHNAK	Known Interactor
AKAP6	Known Interactor
AKAP9	Known Interactor
AKT1S1	Known Interactor
AKT2	Known Interactor
AKTIP	Known Interactor

ALB	Known Interactor
ALYREF	Known Interactor
AMPH	Known Interactor
ANKH	Known Interactor
ANKS1B	Known Interactor
AOC1	Known Interactor
APLP2	Known Interactor
APOH	Known Interactor
APP	Known Interactor
APPL1	Known Interactor
AR	Known Interactor
ARFGAP1	Known Interactor
ARFIP2	Known Interactor
ARHGAP29	Known Interactor
ARHGAP32	Known Interactor
ARIH2	Known Interactor
ATF4	Known Interactor
ATF5	Known Interactor
ATF7IP	Known Interactor
ATXN1	Known Interactor
BAD	Known Interactor
BCL10	Known Interactor
BCL2	Known Interactor
BCL2L1	Known Interactor
BCL2L11	Known Interactor
BGN	Known Interactor
BICD1	Known Interactor
BLOC1S5	Known Interactor
BLOC1S6	Known Interactor
BMPR1B	Known Interactor
BPGM	Known Interactor
BRAF	Known Interactor
BRCA1	Known Interactor
BRF1	Known Interactor
BRK1	Known Interactor
BZRAP1	Known Interactor
C14orf166	Known Interactor
C19orf52	Known Interactor
C19orf66	Known Interactor
CABIN1	Known Interactor
CADPS	Known Interactor
CADPS2	Known Interactor
CALM1	Known Interactor
CALR	Known Interactor
CAMKK1	Known Interactor
CANX	Known Interactor
CARHSP1	Known Interactor

CASP3	Known Interactor
CASP9	Known Interactor
CCDC136	Known Interactor
CCDC141	Known Interactor
CCDC146	Known Interactor
CCDC153	Known Interactor
CCDC24	Known Interactor
CCDC53	Known Interactor
CCDC88A	Known Interactor
CCHCR1	Known Interactor
CCNA2	Known Interactor
CCND3	Known Interactor
CDC27	Known Interactor
CDC37	Known Interactor
CDC5L	Known Interactor
CDK5RAP3	Known Interactor
CDKN1A	Known Interactor
CDKN1B	Known Interactor
CDKN1C	Known Interactor
CEP126	Known Interactor
CEP170	Known Interactor
CEP57L1	Known Interactor
CEP63	Known Interactor
CHEK1	Known Interactor
CHN2	Known Interactor
CHUK	Known Interactor
CIT	Known Interactor
CLIC6	Known Interactor
CLIP3	Known Interactor
CLK2	Known Interactor
CLU	Known Interactor
CMYA5	Known Interactor
CNTF	Known Interactor
COL4A1	Known Interactor
COPB1	Known Interactor
COPB2	Known Interactor
CPE	Known Interactor
CREB1	Known Interactor
CREBBP	Known Interactor
CSF1	Known Interactor
CSNK2A1	Known Interactor
CSNK2B	Known Interactor
CTNNB1	Known Interactor
CTSB	Known Interactor
CYLD	Known Interactor
CYP2C18	Known Interactor
CYP2C8	Known Interactor

DAB2IP	Known Interactor
DCN	Known Interactor
DCTN1	Known Interactor
DCTN2	Known Interactor
DGCR6L	Known Interactor
DLC1	Known Interactor
DLG4	Known Interactor
DLL4	Known Interactor
DMD	Known Interactor
DNAJB1	Known Interactor
DNAJC7	Known Interactor
DNMT1	Known Interactor
DPYSL2	Known Interactor
DPYSL3	Known Interactor
DST	Known Interactor
DTNA	Known Interactor
DTNB	Known Interactor
DYNC1H1	Known Interactor
ECSIT	Known Interactor
EEF1B2	Known Interactor
EEF1G	Known Interactor
EEF2	Known Interactor
EGF	Known Interactor
EGFL7	Known Interactor
EGFR	Known Interactor
EIF3F	Known Interactor
EIF3H	Known Interactor
EIF4EBP1	Known Interactor
ELAVL1	Known Interactor
ELOVL7	Known Interactor
EP300	Known Interactor
EPB41	Known Interactor
EPB41L1	Known Interactor
EPB41L2	Known Interactor
EPN2	Known Interactor
ERBB2	Known Interactor
ERBB3	Known Interactor
ERBB4	Known Interactor
ESR1	Known Interactor
ESR2	Known Interactor
EXOC1	Known Interactor
EXOC4	Known Interactor
EXOC7	Known Interactor
EZH2	Known Interactor
F11R	Known Interactor
FANCA	Known Interactor
FARSA	Known Interactor

FBXL12	Known Interactor
FBXO41	Known Interactor
FBXW7	Known Interactor
FEZ1	Known Interactor
FLNA	Known Interactor
FOXG1	Known Interactor
FOXO1	Known Interactor
FOXO3	Known Interactor
FOXO4	Known Interactor
FRYL	Known Interactor
FXYD7	Known Interactor
FYN	Known Interactor
GAB2	Known Interactor
GABBR1	Known Interactor
GABRA1	Known Interactor
GATA1	Known Interactor
GATA2	Known Interactor
GCDH	Known Interactor
GIPC1	Known Interactor
GLTSCR1L	Known Interactor
GLUL	Known Interactor
GNA11	Known Interactor
GNAI1	Known Interactor
GNAI2	Known Interactor
GNAI3	Known Interactor
GNAO1	Known Interactor
GNAQ	Known Interactor
GNAZ	Known Interactor
GNB1	Known Interactor
GNB2L1	Known Interactor
GNPTAB	Known Interactor
GPRASP2	Known Interactor
GRASP	Known Interactor
GRB10	Known Interactor
GRB2	Known Interactor
GRIA2	Known Interactor
GRIP1	Known Interactor
GSK3A	Known Interactor
GSK3B	Known Interactor
HAUS1	Known Interactor
HAX1	Known Interactor
HERC2P2	Known Interactor
HIST2H2BE	Known Interactor
HMOX1	Known Interactor
HSP90AA1	Known Interactor
HSP90AB1	Known Interactor
HSPB1	Known Interactor

HTRA1	Known Interactor
HTT	Known Interactor
IFIT3	Known Interactor
IFIT5	Known Interactor
IFNG	Known Interactor
IFT20	Known Interactor
IKBKB	Known Interactor
IKZF4	Known Interactor
ILK	Known Interactor
IMMT	Known Interactor
IMPDH2	Known Interactor
INPP5K	Known Interactor
IPO5	Known Interactor
IQSEC1	Known Interactor
IRAK1	Known Interactor
IRS1	Known Interactor
ITGB3	Known Interactor
ITPKC	Known Interactor
ITPR1	Known Interactor
ITPR3	Known Interactor
ITSN1	Known Interactor
JADE1	Known Interactor
JAK2	Known Interactor
JUNB	Known Interactor
KALRN	Known Interactor
KANSL1	Known Interactor
KAT2B	Known Interactor
KAT6A	Known Interactor
KCNJ6	Known Interactor
KCNJ9	Known Interactor
KCNQ5	Known Interactor
KIAA0408	Known Interactor
KIF3A	Known Interactor
KIF3C	Known Interactor
KIFAP3	Known Interactor
KIFC3	Known Interactor
KLHL12	Known Interactor
KRIT1	Known Interactor
KRT10	Known Interactor
KRT31	Known Interactor
KRT40	Known Interactor
KRTAP5-9	Known Interactor
KTN1	Known Interactor
LCAT	Known Interactor
LDLR	Known Interactor
LIMK1	Known Interactor
LITAF	Known Interactor

LONP1	Known Interactor
LOXL4	Known Interactor
LRP1	Known Interactor
LRP2	Known Interactor
LRP8	Known Interactor
LRRK2	Known Interactor
LSM8	Known Interactor
LYN	Known Interactor
MACF1	Known Interactor
MAML1	Known Interactor
MAML2	Known Interactor
MAML3	Known Interactor
MAP1A	Known Interactor
MAP2	Known Interactor
MAP2K4	Known Interactor
MAP3K11	Known Interactor
MAP3K5	Known Interactor
MAP3K8	Known Interactor
MAPK14	Known Interactor
MAPK8IP1	Known Interactor
MAPKAPK2	Known Interactor
MAPT	Known Interactor
MAST1	Known Interactor
MATR3	Known Interactor
MBTPS1	Known Interactor
MDM2	Known Interactor
MDM4	Known Interactor
MEMO1	Known Interactor
METTL1	Known Interactor
MID1IP1	Known Interactor
MLLT10	Known Interactor
MMP17	Known Interactor
MPDZ	Known Interactor
MPPED1	Known Interactor
MRPL28	Known Interactor
MS4A2	Known Interactor
MST1R	Known Interactor
MTCP1	Known Interactor
MTOR	Known Interactor
MUL1	Known Interactor
MVP	Known Interactor
MXD1	Known Interactor
MYO1A	Known Interactor
MYT1L	Known Interactor
NBEA	Known Interactor
NCAM1	Known Interactor
NCK1	Known Interactor

NCOA4	Known Interactor
NCOR2	Known Interactor
NCS1	Known Interactor
NDE1	Known Interactor
NDEL1	Known Interactor
NDN	Known Interactor
NDUFB10	Known Interactor
NEDD4	Known Interactor
NEFM	Known Interactor
NF2	Known Interactor
NFKBIA	Known Interactor
NME3	Known Interactor
NOS3	Known Interactor
NOTCH1	Known Interactor
NR4A1	Known Interactor
NSF	Known Interactor
NTF3	Known Interactor
NTRK2	Known Interactor
NTRK3	Known Interactor
NUF2	Known Interactor
NUP160	Known Interactor
OLFM1	Known Interactor
P4HA3	Known Interactor
PAFAH1B1	Known Interactor
PAK1	Known Interactor
PAK6	Known Interactor
PAWR	Known Interactor
PCMT1	Known Interactor
PCNT	Known Interactor
PCNXL4	Known Interactor
PDCD4	Known Interactor
PDE3B	Known Interactor
PDE4B	Known Interactor
PDIK1L	Known Interactor
PDK2	Known Interactor
PDPK1	Known Interactor
PEA15	Known Interactor
PEX5	Known Interactor
PFKFB1	Known Interactor
PFKFB2	Known Interactor
PGK1	Known Interactor
PHB2	Known Interactor
PI4K2B	Known Interactor
PIAS1	Known Interactor
PIAS2	Known Interactor
PICK1	Known Interactor
PIK3R1	Known Interactor

PKN2	Known Interactor
PLCB1	Known Interactor
PLCG1	Known Interactor
PLEKHA6	Known Interactor
PLEKHO1	Known Interactor
PLTP	Known Interactor
PLXNA1	Known Interactor
PON2	Known Interactor
POU2F1	Known Interactor
PPARGC1B	Known Interactor
PPL	Known Interactor
PPM1A	Known Interactor
PPM1E	Known Interactor
PPP1CA	Known Interactor
PPP1R9B	Known Interactor
PPP2CA	Known Interactor
PPP2R1A	Known Interactor
PPP2R4	Known Interactor
PPP4R1	Known Interactor
PPP5C	Known Interactor
PRAM1	Known Interactor
PRDX2	Known Interactor
PRG2	Known Interactor
PRKAB2	Known Interactor
PRKCQ	Known Interactor
PRKCZ	Known Interactor
PRKDC	Known Interactor
PRNP	Known Interactor
PRTN3	Known Interactor
PSEN1	Known Interactor
PSEN2	Known Interactor
PSME3	Known Interactor
PTAFR	Known Interactor
PTEN	Known Interactor
PTPN1	Known Interactor
RAB11A	Known Interactor
RAB3D	Known Interactor
RABGAP1	Known Interactor
RAC1	Known Interactor
RAD21	Known Interactor
RAF1	Known Interactor
RANBP9	Known Interactor
RARA	Known Interactor
RASSF7	Known Interactor
RBPJ	Known Interactor
RBSN	Known Interactor
RC3H1	Known Interactor

RDX	Known Interactor
RGCC	Known Interactor
RGS2	Known Interactor
RHEB	Known Interactor
RHEBL1	Known Interactor
RICTOR	Known Interactor
RIPK1	Known Interactor
RNF11	Known Interactor
RNF115	Known Interactor
RNF32	Known Interactor
ROGDI	Known Interactor
RPL4	Known Interactor
RPS6KA3	Known Interactor
RPS6KB1	Known Interactor
RQCD1	Known Interactor
S1PR1	Known Interactor
SCARB1	Known Interactor
SDC2	Known Interactor
SDCBP	Known Interactor
SETDB1	Known Interactor
SFR1	Known Interactor
SH2B2	Known Interactor
SH3BP5	Known Interactor
SH3RF1	Known Interactor
SIRT1	Known Interactor
SIRT6	Known Interactor
SKI	Known Interactor
SKP2	Known Interactor
SMAD2	Known Interactor
SMAD3	Known Interactor
SMAD4	Known Interactor
SMAD7	Known Interactor
SMARCB1	Known Interactor
SMARCC1	Known Interactor
SMARCE1	Known Interactor
SMC2	Known Interactor
SMC3	Known Interactor
SNAPIN	Known Interactor
SNCA	Known Interactor
SNTA1	Known Interactor
SNX6	Known Interactor
SORBS2	Known Interactor
SORBS3	Known Interactor
SORT1	Known Interactor
SPARCL1	Known Interactor
SPTAN1	Known Interactor
SPTBN1	Known Interactor

SPTBN4	Known Interactor
SRC	Known Interactor
SRGAP2	Known Interactor
SRGAP3	Known Interactor
SSC5D	Known Interactor
SSTR5	Known Interactor
ST13	Known Interactor
STAT1	Known Interactor
STK3	Known Interactor
STK4	Known Interactor
STX18	Known Interactor
STX1A	Known Interactor
SYBU	Known Interactor
SYNE1	Known Interactor
TBC1D4	Known Interactor
TCEB1	Known Interactor
TCL1A	Known Interactor
TCL1B	Known Interactor
TCL6	Known Interactor
TERF2IP	Known Interactor
TERT	Known Interactor
TFIP11	Known Interactor
TGFB1I1	Known Interactor
TGFBR1	Known Interactor
THAP7	Known Interactor
THEM4	Known Interactor
TIAM2	Known Interactor
TNFAIP3	Known Interactor
TNFRSF1A	Known Interactor
TNFRSF1B	Known Interactor
TNFSF11	Known Interactor
TNIK	Known Interactor
TNKS	Known Interactor
TOPBP1	Known Interactor
TRADD	Known Interactor
TRAF2	Known Interactor
TRAF3IP1	Known Interactor
TRIB3	Known Interactor
TRIM13	Known Interactor
TRIM2	Known Interactor
TRIM27	Known Interactor
TRIM32	Known Interactor
TRIM9	Known Interactor
TRIO	Known Interactor
TRIP13	Known Interactor
TSC1	Known Interactor
TSC2	Known Interactor

TTC3	Known Interactor
TUBB	Known Interactor
TUBB2A	Known Interactor
TXLNB	Known Interactor
TYRO3	Known Interactor
USP4	Known Interactor
USP48	Known Interactor
UTRN	Known Interactor
UXS1	Known Interactor
VEGFA	Known Interactor
VIM	Known Interactor
VKORC1	Known Interactor
VLDLR	Known Interactor
WNK1	Known Interactor
WNK4	Known Interactor
XIAP	Known Interactor
XPNPEP1	Known Interactor
XRN2	Known Interactor
YAP1	Known Interactor
YBX1	Known Interactor
YWHAE	Known Interactor
YWHAG	Known Interactor
YWHAQ	Known Interactor
YWHAZ	Known Interactor
ZFP36L1	Known Interactor
ZHX1	Known Interactor
ZNF197	Known Interactor
ZNF365	Known Interactor
ZNF490	Known Interactor
ZNF558	Known Interactor
ADCY10	Novel Interactor
ADGRA2	Novel Interactor
AHRR	Novel Interactor
ALDH3A2	Novel Interactor
APBA3	Novel Interactor
APOA5	Novel Interactor
APOC1	Novel Interactor
APOC2	Novel Interactor
APOC4	Novel Interactor
AQR	Novel Interactor
ARGLU1	Novel Interactor
ARHGAP1	Novel Interactor
ATP2B1	Novel Interactor
BET1	Novel Interactor
BLOC1S3	Novel Interactor
BRS3	Novel Interactor
BYSL	Novel Interactor

C2	Novel Interactor
C22orf29	Novel Interactor
CA3	Novel Interactor
CAP2	Novel Interactor
CARS	Novel Interactor
CASP1	Novel Interactor
CBWD1	Novel Interactor
CCAR1	Novel Interactor
CD1D	Novel Interactor
CD3E	Novel Interactor
CD3G	Novel Interactor
CD44	Novel Interactor
CD59	Novel Interactor
CD79A	Novel Interactor
CDC42	Novel Interactor
CDK2AP1	Novel Interactor
CEACAM1	Novel Interactor
CEBPD	Novel Interactor
CNST	Novel Interactor
CRABP2	Novel Interactor
CSGALNACT1	Novel Interactor
CST1	Novel Interactor
CTSG	Novel Interactor
CYP51A1	Novel Interactor
DACH1	Novel Interactor
DDR2	Novel Interactor
DDX21	Novel Interactor
DEGS1	Novel Interactor
DEK	Novel Interactor
DIRAS3	Novel Interactor
DKK4	Novel Interactor
E2F6	Novel Interactor
EGLN1	Novel Interactor
ENTPD4	Novel Interactor
ETNK1	Novel Interactor
EXTL1	Novel Interactor
FBXO21	Novel Interactor
FGFR1	Novel Interactor
FHL2	Novel Interactor
GFRA2	Novel Interactor
GGT3P	Novel Interactor
GPC6	Novel Interactor
GSC2	Novel Interactor
HCK	Novel Interactor
HDGFL1	Novel Interactor
HOPX	Novel Interactor
IL10RA	Novel Interactor

ILF2	Novel Interactor
INPPL1	Novel Interactor
ISG20	Novel Interactor
ITM2C	Novel Interactor
KCNC4	Novel Interactor
KCNK4	Novel Interactor
KIF2C	Novel Interactor
LEPROTL1	Novel Interactor
LIPF	Novel Interactor
MBTPS2	Novel Interactor
MEIS2	Novel Interactor
MICAL3	Novel Interactor
MIS12	Novel Interactor
MKI67	Novel Interactor
MMP23B	Novel Interactor
MOB1B	Novel Interactor
MPPED2	Novel Interactor
MPZ	Novel Interactor
MRPL36	Novel Interactor
MRPL40	Novel Interactor
MRPS5	Novel Interactor
MSH5	Novel Interactor
MYO16	Novel Interactor
NEU1	Novel Interactor
NFKBIL1	Novel Interactor
NFYA	Novel Interactor
NQO2	Novel Interactor
NUP153	Novel Interactor
OCM2	Novel Interactor
OSMR	Novel Interactor
PBX2	Novel Interactor
PCDH9	Novel Interactor
PFDN2	Novel Interactor
PILRB	Novel Interactor
POLE2	Novel Interactor
PPIL2	Novel Interactor
PPP1R11	Novel Interactor
PRDM4	Novel Interactor
PSG7	Novel Interactor
PSMB8	Novel Interactor
PSMC3	Novel Interactor
RANBP1	Novel Interactor
RBM22	Novel Interactor
RFXAP	Novel Interactor
RNF144B	Novel Interactor
RRM1	Novel Interactor
RTN4R	Novel Interactor

SDHC	Novel Interactor
5-Sep	Novel Interactor
SGSH	Novel Interactor
SKIV2L	Novel Interactor
SLC18A1	Novel Interactor
SLC22A1	Novel Interactor
SLC27A3	Novel Interactor
SLC27A4	Novel Interactor
SPTSSB	Novel Interactor
SUGT1	Novel Interactor
TAPBP	Novel Interactor
TBX1	Novel Interactor
TDP2	Novel Interactor
TNXB	Novel Interactor
TP53BP2	Novel Interactor
TP73	Novel Interactor
TPMT	Novel Interactor
UAP1	Novel Interactor
UBAP2L	Novel Interactor
UNG	Novel Interactor
VWA5A	Novel Interactor
WWP2	Novel Interactor
ZBP1	Novel Interactor
ZBTB17	Novel Interactor
ZNF219	Novel Interactor
ZNF239	Novel Interactor
ZNF43	Novel Interactor
ZNF804B	Novel Interactor
