# Supplementary material for: Schizophrenia interactome with 504 novel protein–protein interactions
Source: NPJ Schizophr. 2016 Apr 27;2:16012–. doi: 10.1038/npjschz.2016.12 (PMC4898894; doi:10.1038/npjschz.2016.12)
Supplement: Supplementary File 3 [file npjschz201612-s3.pdf]

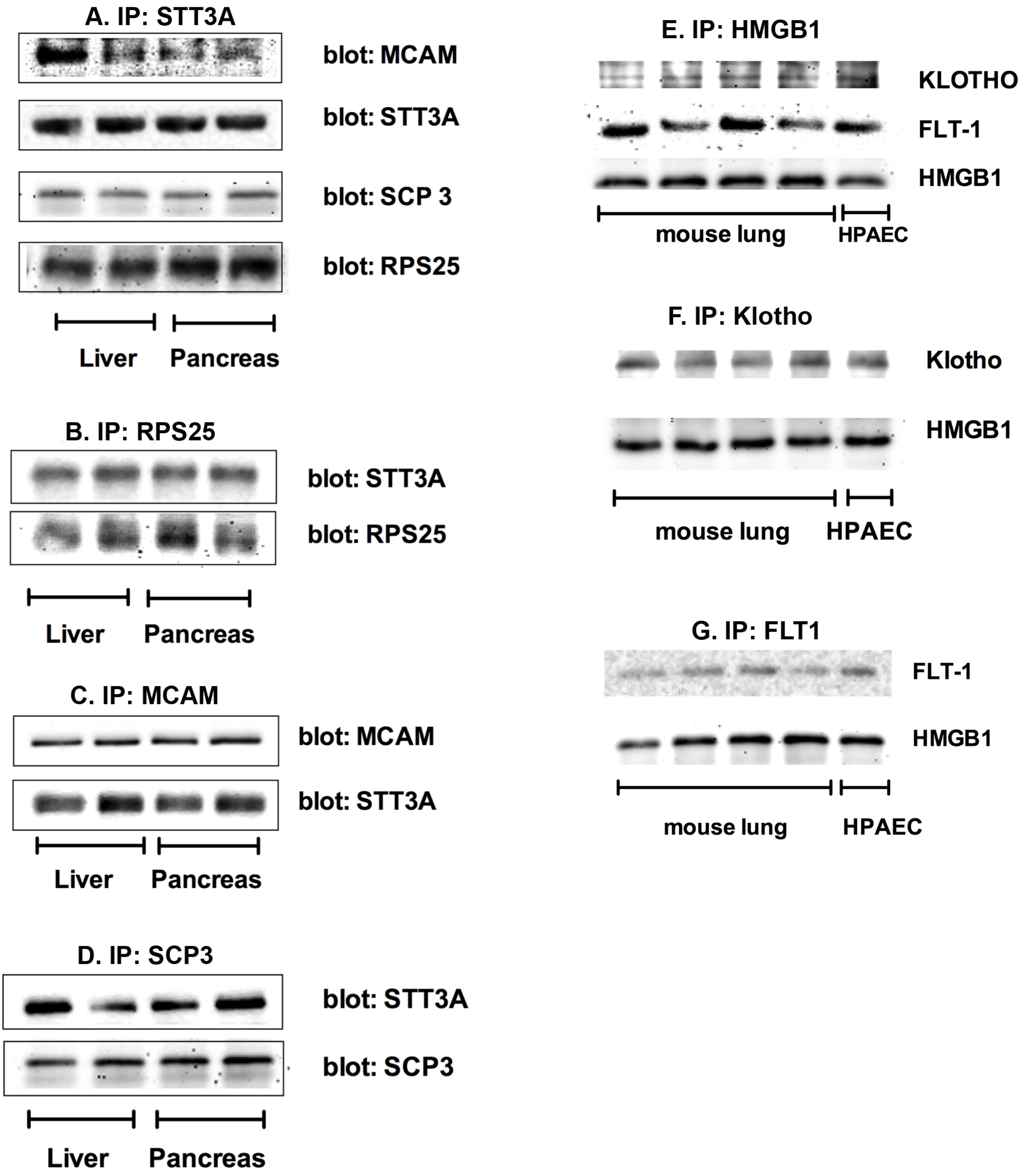
A. IP: STT3A
blot: MCAM
blot: STT3A
blot: SCP 3
blot: RPS25
Liver
Pancreas
B. IP: RPS25
blot: STT3A
blot: RPS25
Liver
Pancreas
C. IP: MCAM
blot: MCAM
blot: STT3A
Liver
Pancreas
D. IP: SCP3
blot: STT3A
blot: SCP3
Liver
Pancreas
E. IP: HMGB1
KLOTHO
FLT-1
HMGB1
mouse lung
HPAEC
F. IP: Klotho
Klotho
HMGB1
mouse lung
HPAEC
G. IP: FLT1
FLT-1
HMGB1
mouse lung
HPAEC
