# Supplementary material for: Schizophrenia interactome with 504 novel protein–protein interactions
Source: NPJ Schizophr. 2016 Apr 27;2:16012–. doi: 10.1038/npjschz.2016.12 (PMC4898894; doi:10.1038/npjschz.2016.12)
Supplement: Supplementary File 4 [file npjschz201612-s4.pdf]

Schizophrenia Interactome Pathways

Pathway	P-value	Schizophrenia Genes	Known Interactors	Novel Interactors
Molecular Mechanisms of Cancer	2.00E-41	TCF4,PAK6,AKT3,PRKD1,AKT1	BCL2L1,AKT2,RBPJ,MAP3K7,ARHGEF1,RABIF,RELA,BRCA1,CDKN2C,ARHGEF12,CAMK2A,CAMK2B,MDM2,SMAD3,ADCY6,CCND1,MAX,CCND3,GSK3A,GSK3B,GRB2,PAK4,APC,PAK1,WNT7B,GNAO1,CDKN1A,NFKBIA,GAB2,RAF1,CCNE1,JUN,SUV39H1,JAK2,JAK3,PIK3R1,JAK1,CHEK1,PLCB1,TCF3,FOS,CDC25A,RHOJ,TP53,GNAQ,HAT1,GNAZ,CREBBP,SMAD9,SMAD4,SMAD7,SMAD1,SMAD2,PRKDC,MAPK14,XIAP,ABL1,MAP3K5,GNAI2,GNAI3,GNAI1,PSEN2,PSEN1,MAPK3,CDKN1B,NFKB1,MAPK8,MAPK9,SRC,TGFBR1,EP300,LRP1,IRS1,BAD,PRKACA,CDK5,CDK6,CDK7,CDC42,CTNNB1,FOXO1,MAP2K4,BCL2L11,FYN,AURKA,SIN3A,PRKCH,BMPR1B,RAC1,PRKCA,PRKCB,PRKCE,PRKCD,PRKCG,PRKCZ,PRKCQ,RASA1,CASP3,NOTCH1,CASP9,BRAF,GNA11,BCL2	APH1B,FZD3,ZBTB17,PRKAR1B,DIRAS3,GNAS,PRKAG1,CCND2,E2F6,ADCY10
PI3K/AKT Signaling	5.01E-31	AKT1,AKT3	BCL2L1,PPP2R1A,PTGS2,CDKN1B,CDKN1A,NFKBIA,GAB2,ILK,IKBKB,BAD,CDC37,RAF1,TSC1,YWHAZ,MAP3K8,THEM4,TSC2,YWHAQ,RPS6KB1,PPP2R4,MAP3K5,HSP90AB1,MAPK3,JAK2,JAK3,PIK3R1,JAK1,YWHAB,NFKB1,RELA,YWHAG,YWHAE,NOS3,CHUK,AKT2,PRKCZ,MTOR,HSP90AA1,MDM2,MAPK8IP1,FOXO3,CTNNB1,TP53,CCND1,PPP2CA,INPP5K,GSK3A,FOXO1,GSK3B,PTEN,GRB2,EIF4EBP1,PDPK1,RHEB,BCL2	INPPL1

14-3-3-mediated Signaling	2.00E-28	PLCH2,AKT1,AKT3,PRKD1,TNF,SRPK2	PLCB1,CDKN1B,TUBB,PLCG2,BAD,MAP2K4,GSK3B,TSC2,TSC1,YWHAZ,TUBA1B,YWHAQ,JUN,MAP3K5,MAPK3,AKT1S1,PIK3R1,YWHAB,MAPK8,MAPK9,YWHAG,YWHAE,PRKCH,TRAF2,FOS,PRKCA,PRKCB,PRKCE,PRKCD,SRC,AKT2,PRKCZ,PRKCG,PRKCQ,TUBB2A,RAF1,YAP1,TNFRSF1A,GSK3A,FOXO1,PLCG1,VIM,GRB2,MAPT,SNCA	TUBA1C,PDIA3,TP73
GNRH Signaling	1.00E-26	PAK6,PRKD1	PLCB1,MAP2K4,EGFR,MAPK14,MAP2K7,DNM2,GNAI2,GNAI3,GNAI1,MAP3K8,ITPR1,RAF1,EGR1,JUN,MAP3K7,MAP3K5,MAPK3,NFKB1,RELA,MAPK9,EGF,PRKCH,FOS,PRKCA,PRKCB,PRKCE,PRKCD,PRKCG,CAMK2A,PRKCZ,CREB1,PRKCQ,ITPR3,EP300,MAPK8,ADCY6,GNAQ,SRC,CAMK2B,MAP3K11,GRB2,PRKACA,RAC1,GNA11,PAK4,ATF4,PAK1,CREBBP,CDC42	PRKAG1,PRKAR1B,ADCY10,GNAS
Huntington's Disease Signaling	3.98E-24	AKT1,BDNF,AKT3,PRKD1	STX1A,BCL2L1,ZDHHC17,HDAC2,HDAC5,HDAC4,HDAC7,HSPA4,GNG10,MAP2K4,EGFR,ARFIP2,PRKCA,AKT2,MAP2K7,DNM2,DCTN1,EGF,ITPR1,DLG4,DNAJB1,JUN,NSF,NCOR2,GNB2L1,MAPK3,HTT,PIK3R1,GNA11,MAPK8,MAPK9,SIN3A,ATF4,PRKCH,IGF1R,GNB5,SP1,PLCB1,PRKCB,GNB1,PRKCD,CREB1,PSME3,PRKCZ,MTOR,PRKCG,AP2A2,PRKCQ,EP300,RASA1,HDAC1,CASP3,NEUROD1,TP53,GNAQ,PRKCE,CASP9,UBD,GRB2,CDK5,UBC,PDPK1,UBE2S,SNCA,CREBBP	POLR2I,CASP1

RAR Activation	3.98E-24	AKT1,AKT3,PRKD1	CSF2RB,SMARCC1,SMAD9,PRKCH,SMAD4,JUN,ERCC3,SMAD1,SMAD2,SMAD3,MAPK14,AKT2,SMARCE1,MAP2K4,SORBS3,MAP3K5,SMARCA4,NCOR2,RARA,RAC1,NR2F1,NR2F2,JAK2,PIK3R1,NFKB1,RELA,MAPK9,SRC,SMAD7,SMARCB1,FOS,PRKCA,PRKCB,PRKCE,PARP1,PRKCG,KAT2B,PRKCZ,VEGFA,PRKCQ,EP300,MAPK8,CSNK2A1,MAPKAPK2,ADCY6,PRKCD,PTEN,PRKACA,CDK7,PDPK1,CSNK2B,CREBBP	PRKAR1B,CRABP1,CRABP2,GNAS,PRKAG1,GTF2H2,RDH5,SRA1,ADCY10
G Beta Gamma Signaling	3.98E-23	AKT1,AKT3,PRKD1	SRC,GNAO1,EGFR,PLCG2,AKT2,DNM2,GNAI2,GNAI3,GNAI1,RAF1,GNG10,GNB2L1,MAPK3,PRKCH,GNB5,GNB1,PRKCB,PRKCE,PRKCD,PRKCG,PRKCZ,PRKCA,PRKCQ,GNAQ,KCNJ6,KCNJ9,PLCG1,GNAZ,GRB2,PRKACA,GNA11,PDPK1,PAK1,BTK,CDC42	PRKAG1,KCNJ3,PRKAR1B,GNAS
Glucocorticoid Receptor Signaling	6.31E-23	AKT1,AKT3,NFATC3,TNF,PPP3CC	BCL2L1,PTGS2,SMARCC1,HSPA4,CDKN1C,CDKN1A,ERCC3,KAT2B,PRKAA1,SMAD3,FOXO3,FKBP5,AKT2,MAP2K7,SMARCE1,MAP2K4,PRKAB2,RAF1,NCOR2,GTF2E1,STAT3,STAT1,SMARCA4,RAC1,JUN,MAP3K7,A2M,HSP90AB1,MAPK3,JAK2,JAK3,PIK3R1,JAK1,NFKB1,RELA,MAPK9,FKBP4,TRAF2,NFKBIA,SMARCB1,TRAF6,FOS,IFNG,TSC22D3,CHUK,CREB1,PRKAA2,AR,SMAD2,EP300,TGFBR1,MAPK8,MAPK14,TAF7L,ESR1,PTGES3,IKBKB,GRB2,PRKACA,CREBBP,CDK7,HSP90AA1,POU2F1,SMAD4,BCL2	PRKAG1,GTF2H2,CSF2,SRA1,POLR2I,CD3E,CD3G

Androgen Signaling	6.31E-23	PRKD1	HSPA4,GNG10,ERCC3,KAT2B,SMAD3,GNAI2,GNAI3,GNAI1,GTF2E1,NCOA4,MAPK3,DNAJB1,JUN,GNB2L1,PRKCA,GNA11,GNAO1,TGFB1I1,RELA,PRKCH,GNB5,PRKCE,PRKCB,GNB1,PRKCD,SRC,PRKCZ,PRKCG,NFKB1,PRKCQ,EP300,GNAQ,CCND1,CALR,CALM1 (includes others),GNAZ,AR,PRKACA,CDK7,HSP90AA1,CREBBP	PRKAG1,GTF2H2,POLR2I,PRKAR1B,GNAS
Dopamine-DARPP32 Feedback in cAMP S	7.94E-23	ATP2A2,DRD4,CACNA1C,DRD2,DRD3,PLCH2,GRIN2A,PPP3CC,PRKD1	PPP2R1A,PLCB1,PPP1CC,PLCG2,PLCG1,GNAI2,GNAI3,GNAI1,EP300,CALM1 (includes others),PPP2R4,GRIN3A,GRIN3B,GRIN1,PPP1R14A,PRKCH,PRKCA,PRKCB,PRKCE,PRKCD,CREB1,PRKCZ,PRKCG,PRKCQ,ITPR3,PAWR,ITPR1,CSNK1A1,ADCY6,GNAQ,PPP1CA,PPP2CA,KCNJ9,KCNJ6,CAMKK1,PRKACA,CDK5,ATF4,CREBBP	KCNJ3,ADCY10,KCNJ4,PRKAG1,GNAS,PDIA3,PPP1R11,CREM,PRKAR1B
Role of NFAT in Cardiac Hypertrophy	1.58E-22	PLCH2,AKT1,AKT3,PRKD1,PPP3CC	HDAC1,HDAC2,HDAC5,CAMK1D,HDAC7,PLCB1,GNG10,TGFBR1,MAPK14,PLCG2,AKT2,MAP2K7,MAP2K4,GNAI2,GNAI3,HDAC4,ITPR1,CABIN1,RAF1,CALM1 (includes others),MAP3K7,GNB2L1,MAPK3,PIK3R1,MAPK8,GNAI1,PRKCH,IGF1R,GNB5,GNB1,PRKCB,PRKCE,CAMK2A,CAMK2B,PRKCD,PRKCZ,PRKCA,PRKCG,PRKCQ,ITPR3,EP300,CSNK1A1,ADCY6,MAPK9,GNAQ,SRC,PLCG1,GSK3B,GRB2,PRKACA	PRKAG1,PDIA3,PRKAR1B,ADCY10,GNAS

Production of Nitric Oxide and Reactive O[illegible]	2.00E-22	AKT1,AKT3,APOE,TNF,PRKD1	PPP2R1A,NFKBIA,IKBKB,PLCG2,AKT2,MAP2K7,MAP2K4,MAP3K8,STAT1,RAC1,JUN,MAP3K7,PPP2R4,MAP3K5,MAPK3,JAK2,JAK3,PIK3R1,JAK1,NFKB1,RELA,MAPK9,PPP1R14A,PRKCH,FOS,IFNG,PRKCB,PRKCE,PRKCD,PRKCG,CHUK,RHOJ,PRKCZ,PRKCA,PRKCQ,CLU,MAPK8,MAPK14,TNFRSF1B,PPP1CC,PPP1CA,PPP2CA,TNFRSF1A,ALB,PLCG1,MAP3K11,CREBBP	DIRAS3,PCYOX1,HOXA10,PPP1R11,APOC4,APOC2,APOC1,APOL1
p70S6K Signaling	3.16E-22	PLCH2,AKT1,AKT3,PRKD1	PPP2R1A,SRC,EGFR,PLCG2,BAD,GNAI2,GNAI3,GNAI1,YWHAZ,RAF1,YWHAQ,RPS6KB1,PPP2R4,MAPK3,PIK3R1,JAK1,YWHAB,LYN,YWHAG,YWHAE,PRKCH,IL4R,PRKCA,PLCB1,PRKCB,PRKCE,PRKCD,PRKCG,AKT2,PRKCZ,MTOR,PRKCQ,GNAQ,PPP2CA,IRS1,PLCG1,SYK,GRB2,EEF2,MAPT,PDPK1,BTK	PDIA3,CD79A
Pancreatic Adenocarcinoma Signaling	5.01E-22	AKT1,AKT3	BCL2L1,PTGS2,SMAD4,CDKN1B,CDKN1A,EGFR,SMAD2,SMAD3,BAD,MAP2K4,ABL1,RAF1,EGF,STAT3,STAT1,CCNE1,MAPK3,JAK2,JAK3,PIK3R1,JAK1,NFKB1,RELA,MAPK9,SIN3A,ERBB2,TGFBR1,RAC1,SUV39H1,AKT2,VEGFA,MDM2,MAPK8,TP53,CCND1,HMOX1,NOTCH1,CASP9,GRB2,BCL2,CDC42	E2F6,PLD3

Axonal Guidance Signaling	7.94E-22	BDNF,NFATC3,PLCH2,AKT1,AKT3,FES,PAK6,PPP3CC,PRKD1	EFNB1,ACE,PLCB1,KALRN,LIMK1,SDCBP,SRGAP3,SRGAP2,PLCG2,AKT2,DPYSL5,ADAM17,ABL1,DPYSL2,GSK3B,GNAI2,EGF,GNAI1,TUBB2A,GNAI3,ITSN1,RAF1,GNG10,NTRK2,NTRK3,GNB2L1,MAPK3,GNA11,PIK3R1,RGS3,GNAO1,TUBB,ERBB2,PRKCH,ARHGEF12,NTF3,EPHB6,RAC1,GNB5,GNB1,FYN,PRKCE,PRKCD,PRKCG,LNPEP,PRKCZ,PRKCA,PRKCQ,COPS5,BCAR1,RASA1,NCK1,PAK1,SEMA3B,VEGFA,GNAQ,NCK2,ARPC1B,SDC2,PLCG1,GNAZ,ADAM9,ADAM19,GRB2,PRKACA,CDK5,TUBA1B,PAK4,BAIAP2,WNT7B,PRKCB,PLXNA1,CDC42	ERAP2,PDGFB,FZD3,PRKAR1B,GNAS,PRKAG1,PDIA3,ARPC3,ADAMTS8,EPHA2,TUBA1C,RTN4R,ADAM23,RND1,SEMA7A,NRP1,MYL9
B Cell Receptor Signaling	1.00E-21	AKT1,AKT3,NFATC3,PPP3CC	BCL2L1,INPP5K,RPS6KB1,NFKBIA,GAB2,IKBKB,PLCG2,PTK2B,MAP2K7,CALM1 (includes others),MAP2K4,ABL1,RAF1,MAP3K8,PTEN,EGR1,JUN,MAP3K7,MAP3K5,MAPK3,PIK3R1,FOXO1,NFKB1,RELA,MAPK9,MAPK8,TCF3,RAC1,PRKCB,CAMK2A,CREB1,CHUK,AKT2,MTOR,CAMK2B,ATF4,PRKCQ,EP300,LYN,MAPK14,SYK,BCL10,VAV2,GSK3A,BAD,GSK3B,MAP3K11,GRB2,CREBBP,PDPK1,BTK,CDC42	INPPL1,CD79A
HER-2 Signaling in Breast Cancer	6.31E-21	NRG1,AKT1,AKT3,PRKD1	ERBB3,CDKN1B,CDKN1A,EGFR,FOXO1,GSK3B,TSC2,EGF,TSC1,CCNE1,MAP3K5,PIK3R1,ERBB2,PRKCH,ITGB3,PRKCA,PRKCB,PRKCE,PRKCD,PRKCG,PRKCZ,PRKCQ,MDM2,TP53,CCND1,GSK3A,AKT2,PLCG1,CASP9,GRB2,BAD,CDK6,CDC42	

ErbB Signaling	1.58E-20	NRG1,AKT1,PAK6,PRKD1	ERBB3,JUN,EGFR,MAPK14,PLCG2,PLCG1,MAP2K4,RAF1,EGF,RPS6KB1,MAPK3,PIK3R1,MAPK8,MAPK9,ERBB2,PRKCH,ERBB4,FOS,PRKCA,PRKCB,PRKCE,PRKCD,PRKCG,PRKCZ,MTOR,PRKCQ,NCK1,NCK2,FOXO1,GSK3B,GRB2,PAK4,PDPK1,PAK1,CDC42	
IL-8 Signaling	2.51E-20	AKT1,AKT3,PRKD1	BCL2L1,PTGS2,PRKCH,GNG10,LIMK1,EGFR,PRKCA,IKBKB,LASP1,AKT2,MAP2K4,GNAI2,GNAI3,GNAI1,RAF1,RAC1,RPS6KB1,EGF,GNB2L1,MAPK3,PIK3R1,NFKB1,RELA,IRAK1,SRC,ITGB3,TRAF6,FOS,GNB5,GNB1,PRKCB,PRKCE,PRKCD,PRKCG,CHUK,RHOJ,JUN,MTOR,PRKCQ,MAPK8,MAPK9,CCND1,CCND3,HMOX1,PTK2B,PRKCZ,EIF4EBP1,BRAF,VEGFA,BCL2	CCND2,GNAS,PLD3,DIRAS3,MYL9
Neuregulin Signaling	3.98E-20	NRG1,AKT1,AKT3,PRKD1	ERBB3,CDKN1B,EGFR,PLCG2,BAD,ADAM17,RAF1,EGF,DCN,DLG4,PSEN1,RPS6KB1,HSP90AB1,MAPK3,PIK3R1,ERBB2,PRKCH,ERBB4,PRKCA,PRKCB,PRKCE,PRKCD,SRC,AKT2,PRKCZ,MTOR,PRKCG,PDPK1,PRKCQ,PICK1,PLCG1,PTEN,GRB2,CDK5,HSP90AA1	
PTEN Signaling	7.94E-20	AKT1,AKT3	BCL2L1,IGF1R,CDKN1B,FOXG1,EGFR,ILK,FOXO3,BMPR1B,FOXO4,CASP9,RAF1,BCL2L11,RPS6KB1,NTRK2,NTRK3,MAPK3,PIK3R1,NFKB1,RELA,CDKN1A,TGFBR1,RAC1,CHUK,AKT2,PRKCZ,BCAR1,CSNK2A1,IKBKB,CASP3,CCND1,INPP5K,GSK3A,FOXO1,BAD,GSK3B,PTEN,GRB2,PDPK1,BCL2,CSNK2B,CDC42	INPPL1,FGFR1

Role of Macrophages, Fibroblasts and End	1.26E-19	TCF4,NFATC3,PLCH2,AKT1,AKT3,PPP3CC,PRKD1,TNF	TNFSF11,SRC,GNAO1,NFKBIA,CSF1,RIPK1,PLCG2,AKT2,MAP2K7,MAP2K4,RAF1,MAPKAPK2,MAPK14,STAT3,TCF3,RAC1,CALM1 (includes others),MAP3K7,TRADD,MAPK3,JAK2,PIK3R1,NFKB1,RELA,IRAK1,PRKCH,TRAF2,TRAF6,FOS,PRKCA,PLCB1,PRKCB,PRKCE,CAMK2A,PRKCG,PRKCD,CHUK,JUN,VEGFA,CREB1,ATF4,PRKCQ,EP300,CSNK1A1,CTNNB1,MAPK9,TNFRSF1B,GNAQ,TNFRSF1A,CCND1,IKBKB,CAMK2B,PLCG1,IL16,GSK3B,PRKCZ,APC,LRP1,CREBBP,WNT7B	PDGFB,FZD3,CEBPG,CEBPD,DKK4,PDIA3,CSF2
Protein Kinase A Signaling	1.58E-19	PLCH2,TCF4,NFATC3,PRKD1,PPP3CC	PTGS2,YWHAQ,CREB1,PLCB1,SMAD4,CALM1 (includes others),NFKBIA,SMAD3,CTNNB1,PLCG2,BAD,AKAP13,GNAI2,GNAI3,GNAI1,YWHAZ,GNB2L1,ITPR1,ADCY6,PPP1CC,RAF1,RYR2,GNG10,AKAP9,PDE3B,APEX1,MAPK3,PDE7B,TTN,YWHAB,NFKB1,RELA,YWHAG,YWHAE,PRKCH,TGFBR1,NOS3,TCF3,GNB5,GNB1,CDC25A,PRKCB,PRKCE,CAMK2A,CAMK2B,PRKCD,CHUK,PRKCZ,PRKCA,PRKCG,FLNA,PRKCQ,ITPR3,PTPN1,EP300,TDP2,PTPN4,AKAP6,HIST3H3,HIST2H3C,GNAQ,PPP1CA,CDC27,GSK3A,PTK2B,PLCG1,GSK3B,PTEN,ATF4,PRKACA,BRAF,PLN,PDE4B,PPP1R14A,CREBBP	PRKAR1B,GNAS,PRKAG1,PDIA3,PPP1R11,CREM,ADCY10,MYL9

AMPK Signaling	1.58E-19	AKT1,AKT3,CHRNA5,CHRNA3	PPP2R1A,SMARCC1,ELAVL1,FOXG1,KAT2B,PRKAA1,MAP3K7,FOXO3,AKT2,SMARCE1,FOXO4,PRKAB2,SMARCA4,TSC1,CCNA2,PPM1A,IRS1,TSC2,RPS6KB1,CHRNB4,PPP2R4,AKT1S1,PIK3R1,PRKACA,CDKN1A,SRC,SIRT1,NOS3,SMARCB1,CREB1,PRKAA2,MTOR,ATF4,EP300,MAPK14,PFKFB1,PPP2CA,PFKFB2,FOXO1,IRS2,INS,CCND1,EIF4EBP1,EEF2,PDPK1,CFTR,CREBBP	PRKAG1,CAB39,ACACA,PRKAR1B,GNAS
Ephrin Receptor Signaling	2.00E-19	AKT1,PAK6,AKT3,GRIN2A	EFNB1,GNAO1,LIMK1,SDCBP,AKT2,ABL1,GNAI2,GNAI3,GNAI1,GNB2L1,STAT3,ITSN1,RAF1,GNG10,EGF,GRIN3A,MAPK3,DOK1,JAK2,KALRN,GRIN1,SRC,EPHB6,RAC1,GNB5,RGS3,GNB1,GRIN3B,CREB1,VEGFA,EP300,BCAR1,RASA1,NCK1,FYN,GNAQ,NCK2,ARPC1B,SDC2,GNAZ,GRB2,GNA11,PAK4,ATF4,PAK1,CREBBP,CDC42	ARPC3,PDGFB,EPHA2,GNAS
FcÎ³ Receptor-mediated Phagocytosis in M	5.01E-19	AKT1,AKT3,PRKD1	ACTG1,SRC,FYN,GAB2,AKT2,RPS6KB1,RAB11A,MAPK3,PIK3R1,LYN,PRKCH,RAC1,PRKCA,PRKCB,PRKCE,PRKCD,PRKCG,PRKCZ,PRKCQ,NCK1,NCK2,SYK,ARPC1B,VAV2,HMOX1,PTK2B,PLCG1,PTEN,PAK1,CDC42	PLD3,ARPC3,CSF2,PLA2G6,HCK,LCP2
PI3K Signaling in B Lymphocytes	6.31E-19	PLCH2,AKT1,AKT3,NFATC3,PPP3CC	FYN,NFKBIA,IRS1,IKBKB,PLCG2,AKT2,ABL1,RAF1,RAC1,CALM1 (includes others),BCL10,MAPK3,PIK3R1,NFKB1,RELA,PLCB1,IL4R,FOS,PRKCB,CHUK,CREB1,CAMK2A,JUN,SH2B2,CAMK2B,PDPK1,ITPR3,ITPR1,LYN,FOXO3,SYK,VAV2,PLCG1,IRS2,PTEN,PRKCZ,ATF5,ATF4,BTK	PDIA3,CD79A

Thrombin Signaling	1.00E-18	PLCH2,AKT1,AKT3,PRKD1	CAMK1D,PLCB1,GNG10,EGFR,IKBKB,PLCG2,AKT2,GNAI2,GNAI3,GNAI1,RAF1,RPS6KB1,ARHGEF1,GNB2L1,MAPK3,PIK3R1,GNAO1,NFKB1,RELA,EGF,PRKCH,ARHGEF12,GNB5,GNB1,PRKCB,PRKCE,CAMK2A,PRKCG,PRKCD,RHOJ,PRKCZ,PRKCA,CREB1,PRKCQ,ITPR3,ITPR1,GATA2,GATA1,MAPK14,ADCY6,GNAQ,SRC,CAMK2B,PLCG1,GNAZ,GRB2,GNA11,PDPK1	GNAS,PDIA3,ADCY10,DIRAS3,MYL9
Renin-Angiotensin Signaling	1.00E-18	PAK6,TNF,PRKD1	ACE,MAPK14,PLCG2,PTK2B,MAP2K4,RAF1,STAT3,STAT1,RAC1,JUN,MAPK3,JAK2,PIK3R1,NFKB1,MAPK8,MAPK9,PRKCH,FOS,PRKCA,PRKCB,PRKCE,PRKCD,PRKCG,PRKCZ,PRKCQ,ITPR3,ITPR1,RELA,ADCY6,GNAQ,PLCG1,GRB2,PRKACA,PAK4,PAK1	PRKAG1,PRKAR1B,ADCY10,GNAS
Breast Cancer Regulation by Stathmin1	1.00E-18	PRKD1	PPP2R1A,CAMK1D,GNAI2,PLCB1,CALM1 (includes others),CDKN1A,LIMK1,RAF1,GNAI3,GNAI1,TUBA1B,CCNE1,GNG10,PPP2R4,ARHGEF1,GNB2L1,MAPK3,PIK3R1,CDKN1B,PPP1R14A,PRKCH,ARHGEF12,RAC1,GNB5,GNB1,PRKCB,PRKCE,CAMK2A,CAMK2B,PRKCD,PRKCZ,PRKCA,PRKCG,PRKCQ,ITPR3,ITPR1,TUBB2A,PPP1CC,ADCY6,TP53,GNAQ,PPP1CA,PPP2CA,TUBB,CDK1,GRB2,PRKACA,PAK1,CDC42	E2F6,ADCY10,GNAS,PRKAG1,PPP1R11,TUBA1C,PRKAR1B
Prostate Cancer Signaling	1.58E-18	AKT1,AKT3	CDKN1B,CDKN1A,NFKBIA,CTNNB1,FOXO1,ABL1,CASP9,RAF1,CCNE1,HSP90AB1,MAPK3,PIK3R1,NFKB1,RELA,SIN3A,ATF4,SUV39H1,CHUK,CREB1,AR,MTOR,PDPK1,MDM2,EP300,TP53,CCND1,AKT2,BAD,GSK3B,PTEN,GRB2,HSP90AA1,CREBBP,BCL2	

Tec Kinase Signaling	6.31E-18	PAK6,TNF,PRKD1	ACTG1,SRC,GNG10,MAPK8,PLCG2,PTK2B,MAP2K4,MS4A2,GNAI3,GNAI1,STAT3,STAT1,GNAI2,FYN,GNB2L1,GNB5,JAK2,JAK3,PIK3R1,GNAO1,NFKB1,RELA,MAPK9,PRKCH,PRKCB,FOS,PRKCA,PRKCE,JAK1,GNB1,PRKCD,PRKCG,RHOJ,PRKCZ,PRKCQ,LYN,GNAQ,VAV2,PLCG1,GNAZ,GNA11,PAK4,PAK1,BTK	HCK,DIRAS3,GNAS
P2Y Purigenic Receptor Signaling Pathwa	6.31E-18	PLCH2,AKT1,AKT3,PRKD1	PLCB1,JUN,PLCG2,AKT2,GNAI2,GNAI3,GNAI1,RAF1,GNG10,GNB2L1,MAPK3,PIK3R1,NFKB1,RELA,PRKCH,ITGB3,FOS,GNB5,PRKCE,PRKCB,GNB1,PRKCD,CREB1,PRKCZ,PRKCA,PRKCG,PRKCQ,EP300,ADCY6,GNAQ,PLCG1,PRKACA,ATF4,CREBBP	PRKAG1,PDIA3,PRKAR1B,ADCY10,GNAS
Glioma Signaling	1.00E-17	AKT1,AKT3,PRKD1	CAMK1D,CDKN2C,CDKN1A,EGFR,PLCG2,AKT2,ABL1,RAF1,EGF,CALM1 (includes others),MAPK3,PIK3R1,SIN3A,PRKCH,IGF1R,SUV39H1,PRKCB,PRKCE,PRKCD,CAMK2B,CAMK2A,PRKCZ,PRKCA,PRKCG,PRKCQ,MDM2,TP53,CCND1,PLCG1,PTEN,GRB2,CDK6,MTOR	E2F6,PDGFB
CREB Signaling in Neurons	1.26E-17	PLCH2,AKT1,AKT3,GRM3,PRKD1,GRIN2A	PLCB1,GRIA2,GNAO1,PLCG2,AKT2,GNAI2,GNAI3,GNAI1,EP300,RAF1,GNG10,GNB2L1,MAPK3,PIK3R1,GRIN1,PRKCH,GNB5,GNB1,PRKCB,PRKCE,CAMK2A,PRKCG,PRKCD,PRKCZ,PRKCA,CREB1,PRKCQ,ITPR3,ITPR1,ADCY6,GNAQ,CALM1 (includes others),CAMK2B,PLCG1,GNAZ,GRB2,PRKACA,GNA11,ATF4,CREBBP	ADCY10,GNAS,PRKAG1,PDIA3,PRKAR1B,POLR2I
Type II Diabetes Mellitus Signaling	2.00E-17	AKT1,AKT3,PRKD1,TNF	NFKBIA,PRKAA2,PRKAA1,IKBKB,AKT2,MAP2K7,MAP2K4,MAP3K7,TRADD,MAP3K5,MAPK3,PIK3R1,NFKB1,RELA,MAPK9,PRKCH,TRAF2,PRKAB2,PRKCA,PRKCB,PRKCE,CHUK,PRKCG,PRKCD,PRKCZ,MTOR,PDPK1,PRKCQ,MAPK8,TNFRSF1B,TNFRSF1A,IRS1,IRS2,INS,SH2B2	PRKAG1,SLC27A4,SLC27A3

PPARÎ±/RXRÎ± Activation	2.51E-17	PLCH2	SMAD4,NR2F1,NFKBIA,PRKAA2,PRKAA1,SMAD3,MAPK14,PLCG2,PLCG1,MAP2K7,MAP2K4,RAF1,NCOR2,CAND1,JUN,MAP3K7,HSP90AB1,MAPK3,JAK2,IKBKB,NFKB1,RELA,PLCB1,TGFBR1,ABCA1,PRKAB2,TRAF6,PRKCA,PRKCB,CHUK,SMAD2,AP2A2,EP300,MAPK8,CYP2C8,ADCY6,GNAQ,IRS1,INS,GRB2,PRKACA,CYP2C18,GNA11,HSP90AA1,CREBBP	ADCY10,ACVR1B,PRKAG1,MED23,GNAS,PDIA3,PRKAR1B
Colorectal Cancer Metastasis Signaling	3.16E-17	TCF4,AKT3,TNF,AKT1	BCL2L1,PTGS2,MMP17,GNB5,SMAD4,GNG10,EGFR,SMAD2,SMAD3,CTNNB1,AKT2,MAP2K4,GSK3B,EGF,STAT3,STAT1,RAC1,JUN,PTGER3,GNB2L1,MAPK3,JAK2,JAK3,PIK3R1,JAK1,NFKB1,RELA,MAPK9,SRC,TGFBR1,TCF3,FOS,IFNG,GNB1,RHOJ,VEGFA,MAPK8,ADCY6,LRP1,TP53,CASP3,APPL1,CCND1,TNFRSF1A,BAD,CASP9,GRB2,PRKACA,BRAF,APC,WNT7B	FZD3,PRKAR1B,DIRAS3,GNAS,PRKAG1,MMP23B,ADCY10
CXCR4 Signaling	3.98E-17	AKT1,PAK6,AKT3,PRKD1	PLCB1,JUN,AKT2,MAP2K4,GNAI2,GNAI3,GNAI1,RAF1,EGR1,GNG10,GNB2L1,MAPK3,PIK3R1,GNAO1,MAPK8,MAPK9,PRKCH,FOS,GNB5,PRKCE,PRKCB,GNB1,PRKCD,SRC,RHOJ,PRKCZ,PRKCA,PRKCG,PRKCQ,ITPR3,ITPR1,BCAR1,LYN,ADCY6,GNAQ,GNAZ,RAC1,GNA11,PAK4,PAK1	GNAS,ADCY10,DIRAS3,MYL9
Synaptic Long Term Potentiation	3.98E-17	CACNA1C,PLCH2,GRIN2A,PPP3CC,PRKD1,GRM3	PLCB1,GRIA2,PLCG2,PLCG1,RAF1,EP300,CALM1 (includes others),GRIN3A,MAPK3,GRIN3B,GRIN1,PPP1R14A,PRKCH,PRKCA,PRKCB,PRKCE,PRKCD,PRKCG,CAMK2A,PRKCZ,CREB1,PRKCQ,ITPR3,ITPR1,PPP1CC,GNAQ,PPP1CA,CAMK2B,PRKACA,GNA11,ATF4,CREBBP	PRKAG1,PPP1R11,PDIA3,PRKAR1B

Phospholipase C Signaling	3.98E-17	NFATC3,PRKD1,PPP3CC	HDAC1,HDAC2,HDAC5,HDAC4,HDAC7,SRC,FYN,PLCG2,PLCG1,RAF1,ITPR1,AHNAK,GNG10,ARHGEF1,GNB2L1,MAPK3,NFKB1,RELA,PPP1R14A,PRKCH,ARHGEF12,RAC1,GNB5,GNB1,PLCB1,PRKCB,PRKCE,PRKCD,CREB1,RHOJ,PRKCZ,PRKCA,PRKCG,PRKCQ,ITPR3,EP300,LYN,ADCY6,RPS6KA3,GNAQ,SYK,CALM1 (includes others),HMOX1,GRB2,CREBBP,ATF4,BTK	PLA2G10,DIRAS3,PLD3,GNAS,PLA2G6,CD3G,ZAP70,ADCY10,LCP2,MYL9,CD3E,CD79A
GÎ±q Signaling	5.01E-17	NFATC3,RGS4,AKT1,AKT3,HTR2A,PPP3CC,PRKD1	PLCB1,GNG10,NFKBIA,IKBKB,PLCG2,PTK2B,RAF1,CALM1 (includes others),GNB2L1,MAPK3,RGS2,NFKB1,RELA,PRKCH,PIK3R1,GNB5,GNB1,PRKCB,PRKCE,CHUK,PRKCG,PRKCD,RHOJ,PRKCZ,PRKCA,PRKCQ,ITPR3,ITPR1,GNAQ,HMOX1,AKT2,PLCG1,GSK3B,GNA11,BTK	GNAS,RGS7,PLD3,DIRAS3,HRH1
Corticotropin Releasing Hormone Signalin	1.00E-16	BDNF,PRKD1	PTGS2,JUN,MAPK14,PLCG2,PLCG1,GNAI2,GNAI3,GNAI1,ITPR1,RAF1,CALM1 (includes others),MAPK3,GNAO1,PRKCH,NOS3,FOS,PRKCA,PRKCB,PRKCE,PRKCD,PRKCG,PRKCZ,VEGFA,CREB1,PRKCQ,ITPR3,EP300,ADCY6,GNAQ,NR4A1,PRKACA,BRAF,ATF4,CREBBP	PRKAG1,PRKAR1B,ADCY10,GNAS
Gap Junction Signaling	1.00E-16	DRD2,PLCH2,AKT1,AKT3,HTR2A,PPP3CC,PRKD1	ACTG1,GNAI2,SRC,EGFR,CTNNB1,PLCG2,AKT2,RAF1,GNAI3,GNAI1,TUBA1B,EGF,MAPK3,PIK3R1,PRKCH,PRKCA,SP1,PLCB1,PRKCB,PRKCE,PRKCD,PRKCG,PRKCZ,PRKCQ,ITPR3,ITPR1,TUBB2A,CSNK1A1,ADCY6,GNAQ,TUBB,PLCG1,GRB2,PRKACA	ADCY10,GNAS,PRKAG1,SP3,PDIA3,TUBA1C,PRKAR1B
EGF Signaling	1.58E-16	AKT1,AKT3	JUN,EGFR,MAPK14,AKT2,MAP2K7,MAP2K4,RAF1,EGF,STAT3,STAT1,RPS6KB1,MAPK3,PIK3R1,JAK1,MAPK8,SRC,FOS,PRKCA,MTOR,ITPR3,ITPR1,RASA1,CSNK2A1,PLCG1,GRB2,CSNK2B	

UVB-Induced MAPK Signaling	3.16E-16	AKT1,PRKD1	JUN,EGFR,MAPK14,BAD,MAP2K4,RPS6KB1,MAPK3,PIK3R1,MAPK8,MAPK9,PRKCH,FOS,PRKCA,PRKCB,PRKCE,PRKCD,PRKCG,PRKCZ,HIST2H3C,PRKCQ,RPS6KA3,TP53,EIF4EBP1,MTOR,HIST3H3	
Role of NFAT in Regulation of the Immune	3.16E-16	AKT1,AKT3,NFATC3,PPP3CC	GNG10,NFKBIA,IKBKB,PLCG2,AKT2,GNAI2,GNAI3,GNAI1,CABIN1,RAF1,FYN,GNB2L1,MAPK3,PIK3R1,GNAO1,NFKB1,RELA,MS4A2,PLCB1,FOS,GNB5,GNB1,CHUK,JUN,PRKCQ,ITPR3,ITPR1,LYN,CSNK1A1,GNAQ,SYK,CALM1 (includes others),GSK3A,PLCG1,GNAZ,GSK3B,GRB2,GNA11,HLA-DMB,BTK	GNAS,ZAP70,LCP2,CD79A,CD3E,CD3G
NF-Î°B Signaling	5.01E-16	AKT1,AKT3,TNF	HDAC1,TNFSF11,HDAC2,NFKBIA,EGFR,TRAF2,IKBKB,PLCG2,BMPR1B,MAP2K7,NTRK2,INS,RAF1,EGF,MAP3K8,PELI1,MAP3K7,TRADD,NTRK3,TDP2,PIK3R1,NFKB1,RELA,IRAK1,IGF1R,TRAF6,PRKCB,CHUK,PRKCZ,PRKCQ,EP300,TGFBR1,MAPK8,CSNK2A1,RIPK1,TNFRSF1B,TNFRSF1A,BCL10,AKT2,TNFAIP3,GSK3B,PRKACA,BRAF,CSNK2B,CREBBP	ZAP70,FGFR1
G-Protein Coupled Receptor Signaling	5.01E-16	DRD4,DRD2,DRD3,RGS4,AKT1,AKT3,HTR2A,GRM3	CREB1,PLCB1,GNAO1,NFKBIA,IKBKB,PTK2B,GABBR1,PTGER3,GNAI2,GNAI3,GNAI1,ADORA2A,MAP3K8,STAT3,RAF1,S1PR1,FYN,PDE3B,APEX1,MAPK3,PDE7B,PIK3R1,GLP1R,NFKB1,RELA,SRC,RGS2,PRKCA,PRKCB,PRKCE,CAMK2A,PRKCG,CHUK,CAMK2B,ATF4,EP300,RASA1,TDP2,ADCY6,GNAQ,AKT2,GRB2,PRKACA,BRAF,GNA11,PDPK1,PDE4B,CREBBP	ADCY10,GNAS,PRKAG1,HCAR3,RGS7,PRKAR1B,HRH1

Neuropathic Pain Signaling In Dorsal Horn	5.01E-16	PLCH2,GRIN2A,KCNN3,BDNF,PRKD1,GRM3	CAMK1D,PLCB1,GRIA2,PLCG2,PLCG1,NTRK2,GRIN3A,MAPK3,GRIN3B,PIK3R1,GRIN1,PRKCH,FOS,PRKCA,PRKCB,PRKCE,CAMK2A,CREB1,PRKCD,PRKCZ,PRKCG,PRKCQ,ITPR3,ITPR1,SRC,CAMK2B,PRKACA,KCNN2	PRKAG1,PDIA3,PRKAR1B
ERK/MAPK Signaling	7.94E-16	PAK6,VRK2	PPP2R1A,SRC,PLCG2,BAD,MAPKAPK5,RAF1,YWHAZ,STAT3,STAT1,YWHAQ,FYN,PPP2R4,MAPK3,PIK3R1,YWHAB,EIF4EBP1,YWHAG,FOS,PPP1R14A,HSPB7,HSPB1,PRKCA,PRKCB,PRKCE,PRKCD,CREB1,HIST2H3C,PRKCG,EP300,BCAR1,PPP1CC,PPP1CA,PPP2CA,ESR1,PTK2B,PLCG1,GRB2,PRKACA,BRAF,RAC1,PAK4,ATF4,HIST3H3,PAK1,CREBBP	PRKAG1,PLA2G6,PPP1R11,PLA2G10,PRKAR1B
Glioblastoma Multiforme Signaling	1.00E-15	PLCH2,AKT1,AKT3	SRC,CDKN1B,CDKN1A,EGFR,CTNNB1,PLCG2,AKT2,RAF1,EGF,TSC1,TSC2,CCNE1,RPS6KB1,MAPK3,NF2,PIK3R1,PLCB1,IGF1R,TCF3,RAC1,PRKCD,RHOJ,MTOR,ITPR3,MDM2,ITPR1,TP53,CCND1,FOXO1,PLCG1,GSK3B,PTEN,GRB2,CDK6,APC,WNT7B,CDC42	E2F6,PDGFB,FZD3,PDIA3,DIRAS3
Insulin Receptor Signaling	1.00E-15	AKT1,AKT3	RPS6KB1,FOXO3,BAD,FOXO4,INS,RAF1,TSC1,TSC2,FYN,PDE3B,MAPK3,JAK2,PIK3R1,JAK1,EIF4EBP1,MAPK8,PPP1R14A,FOXO1,AKT2,PRKCZ,MTOR,PDPK1,PTPN1,NCK1,PPP1CC,PPP1CA,GRB10,INPP5K,GSK3A,IRS1,IRS2,GSK3B,PTEN,GRB2,PRKACA,SH2B2	PRKAG1,PPP1R11,PRKAR1B,INPPL1,ASIC2
IGF-1 Signaling	1.26E-15	AKT1,AKT3	RPS6KB1,FOXO3,BAD,RAF1,YWHAZ,STAT3,YWHAQ,NEDD4,MAPK3,GRB2,JAK2,PIK3R1,JAK1,YWHAB,MAPK8,YWHAG,YWHAE,IGF1R,FOS,FOXO1,AKT2,JUN,RASA1,CSNK2A1,GRB10,IRS1,IRS2,CASP9,PRKCZ,PRKACA,PDPK1,CSNK2B	PRKAG1,PRKAR1B

Fc Epsilon RI Signaling	1.58E-15	AKT1,AKT3,PRKD1,TNF	VAV2,MAPK14,PLCG2,PLCG1,MAP2K7,MAP2K4,MS4A2,RAF1,FYN,MAPK3,PIK3R1,MAPK8,MAPK9,PRKCH,RAC1,PRKCA,PRKCB,PRKCE,PRKCD,PRKCG,PRKCZ,PRKCQ,LYN,SYK,INPP5K,AKT2,GRB2,PDPK1,BTK	PLA2G6,LCP2,PLA2G10,CSF2,INPPL1
CCR5 Signaling in Macrophages	1.58E-15	PRKD1	JUN,MAPK14,PLCG2,PLCG1,MAP2K4,GNAI2,GNAI3,GNAI1,GNG10,GNB2L1,PRKCA,MAPK8,MAPK9,PRKCH,FOS,GNB5,GNB1,PRKCB,PRKCE,PRKCD,PRKCG,PRKCZ,PRKCQ,CALM1 (includes others),PTK2B	CD3G,CD3E,CCL4,GNAS
Sertoli Cell-Sertoli Cell Junction Signaling	2.00E-15	AKT1,AKT3,MPP6,TNF	ACTG1,SPTAN1,CLDN19,ILK,EPB41,CTNNB1,AKT2,MAP3K11,MAP2K7,MAP2K4,RAF1,A2M,SPTBN1,DLG1,MAP3K8,ACTN2,BCAR1,KEAP1,TUBA1B,JUN,MAP3K7,MAP3K5,MAPK3,JUP,MAPK8,MAPK9,SRC,NOS3,RAC1,EPN2,TUBB2A,F11R,MAPK14,ACTN1,TNFRSF1A,GSK3A,TUBB,GSK3B,PTEN,PRKACA,CDC42	PRKAG1,TUBA1C,CLDN18,PRKAR1B,ADCY10
eNOS Signaling	2.00E-15	AKT1,AKT3,PRKD1,CHRNA5,CHRNA3	HSPA4,PRKAA2,PRKAA1,PLCG2,AKT2,DNM2,CCNA2,CALM1 (includes others),CHRNB4,HSP90AB1,PIK3R1,PRKCH,NOS3,PRKAB2,AQP1,PRKCA,PRKCB,PRKCE,PRKCD,PRKCG,PRKCZ,VEGFA,PDPK1,PRKCQ,ITPR3,ITPR1,CASP3,ADCY6,ESR2,GNAQ,ESR1,PLCG1,CASP9,PRKACA,HSP90AA1	PRKAG1,PRKAR1B,ADCY10,GNAS
Melatonin Signaling	2.51E-15	PLCH2,PRKD1	PLCB1,GNAO1,PLCG2,PLCG1,MAP2K7,MAP2K4,RAF1,GNAI3,GNAI1,GNAI2,CALM1 (includes others),MAPK3,PRKCH,PRKCA,PRKCB,PRKCE,CAMK2A,PRKCG,PRKCD,PRKCZ,CAMK2B,PRKCQ,GNAQ,PRKACA,BRAF	PRKAG1,PDIA3,PRKAR1B

Endothelin-1 Signaling	1.00E-14	PLCH2,PRKD1	PTGS2,PTGS1,PLCB1,GNAO1,LCAT,MAPK14,PLCG2,PLCG1,GNAI2,GNAI3,GNAI1,RAF1,JUN,MAPK3,PIK3R1,MAPK8,MAPK9,PRKCH,NOS3,FOS,PRKCA,PRKCB,PRKCE,PRKCD,SRC,PRKCZ,PRKCG,PRKCQ,ITPR3,ITPR1,CASP3,ADCY6,GNAQ,HMOX1,GNAZ,CASP9,GRB2,BRAF,GNA11	PLA2G10,PLD3,CASP1,PDIA3,PLA2G6,ADCY10,GNAS
ILK Signaling	1.26E-14	AKT1,AKT3,TNF	PPP2R1A,ACTG1,PTGS2,ILK,CTNNB1,AKT2,MAP2K4,RICTOR,JUN,PPP2R4,MAPK3,PDPK1,PIK3R1,TGFB1I1,RELA,MAPK9,ITGB3,FOS,MUC1,CREB1,RHOJ,MTOR,GSK3B,NFKB1,EP300,MAPK8,ACTN2,ACTN1,VEGFA,CASP3,NCK2,TNFRSF1A,CCND1,PPP2CA,GSK3A,IRS1,IRS2,VIM,PTEN,ATF4,SH2B2,FLNA,CREBBP,CDC42	SNAI2,DIRAS3,MYL9
Role of Osteoblasts, Osteoclasts and Cho	1.58E-14	TCF4,NFATC3,AKT1,AKT3,PPP3CC,TNF	TNFSF11,SMAD9,SMAD4,CALM1 (includes others),ADAM17,SMAD1,IKBKB,PTK2B,MAP2K7,MAP2K4,CASP9,TCF3,JUN,MAP3K7,TRADD,MAP3K5,MAPK3,CTNNB1,PIK3R1,NFKB1,RELA,MAPK9,SRC,TRAF2,NFKBIA,TRAF6,FOS,IFNG,CHUK,FOXO1,AKT2,MAPK8,ITGB3,MAPK14,LRP1,TNFRSF1B,CSNK1A1,TNFRSF1A,XIAP,CSF1,BAD,GSK3B,SPP1,APC,WNT7B,BCL2	DKK4,FZD3,CSF2
Cardiac Hypertrophy Signaling	3.16E-14	PLCH2,AKT1,CACNA1C,PPP3CC	RPS6KB1,TGFBR1,MAPK14,PLCG2,PLCG1,MAP2K7,HAND2,MAP2K4,GNAI2,GNAI3,GNAI1,MAP3K8,RAF1,GNG10,MAP3K7,MAP3K5,GNB2L1,MAPK3,PIK3R1,GNAO1,MAPK8,MAPK9,PLCB1,IGF1R,HSPB1,GNB5,GNB1,CREB1,RHOJ,JUN,MTOR,EP300,MAPKAPK2,ADCY6,GNAQ,CALM1 (includes others),IRS1,GNAZ,GSK3B,MAP3K11,GRB2,PRKACA,GNA11,CREBBP	ADCY10,DIRAS3,GNAS,PRKAG1,PDIA3,PRKAR1B,MYL9

IL-3 Signaling	3.98E-14	AKT1,AKT3,PRKD1,PPP3CC	CSF2RB,GAB2,AKT2,RAF1,STAT3,STAT1,RAC1,JUN,MAPK3,JAK2,PIK3R1,JAK1,PRKCH,FOS,PRKCA,PRKCB,PRKCE,PRKCD,PRKCG,PRKCZ,PRKCQ,FOXO1,BAD,GRB2,PAK1	
IL-1 Signaling	3.98E-14	NONE	JUN,NFKBIA,IKBKB,MAP2K7,MAP2K4,GNAI2,GNAI3,GNAI1,GNG10,MAP3K7,GNB2L1,GNAO1,NFKB1,MAPK8,MAPK9,TRAF6,FOS,GNB5,GNB1,CHUK,ECSIT,RELA,MAPK14,ADCY6,IRAK1,GNAQ,GNAZ,PRKACA,GNA11	PRKAG1,PRKAR1B,ADCY10,GNAS
LPS-stimulated MAPK Signaling	7.94E-14	PRKD1	NFKBIA,IKBKB,MAP2K4,RAF1,FOS,JUN,MAP3K7,MAP3K5,MAPK3,PIK3R1,NFKB1,MAPK8,MAPK9,PRKCH,RAC1,PRKCA,PRKCB,PRKCE,PRKCD,CREB1,CHUK,PRKCZ,PRKCG,PRKCQ,RELA,MAPK14,PAK1,CDC42	
Chronic Myeloid Leukemia Signaling	7.94E-14	AKT1,AKT3	HDAC1,BCL2L1,HDAC2,HDAC5,HDAC4,HDAC7,SMAD4,CDKN1B,CDKN1A,GAB2,SMAD3,IKBKB,BAD,ABL1,RAF1,MAPK3,PIK3R1,NFKB1,RELA,SIN3A,TGFBR1,SUV39H1,CHUK,MDM2,TP53,CCND1,AKT2,GRB2,CDK6,CTBP1	E2F6
ATM Signaling	7.94E-14	NONE	CDKN1A,NFKBIA,MAPK14,MAP2K4,ABL1,CBX5,SMC3,SMC2,JUN,MAPK8,MAPK9,CHEK1,BRCA1,TRIM28,CDC25A,CREB1,MDM2,EP300,MDM4,TP53,CDK1,ATF4,CREBBP	BLM,SMC1B,TP73
Cell Cycle: G1/S Checkpoint Regulation	1.00E-13	NRG1	HDAC1,HDAC2,HDAC5,HDAC4,HDAC7,SMAD4,CDKN1B,CDKN1A,SMAD3,FOXO1,ABL1,SKP2,CCNE1,SIN3A,CDKN2C,SUV39H1,CDC25A,MDM2,TP53,CCND1,MAX,CCND3,GSK3B,CDK6	E2F6,CCND2

HGF Signaling	1.26E-13	AKT1,AKT3,PRKD1	PTGS2,CDKN1A,PLCG2,AKT2,MAP2K7,MAP2K4,RAF1,MAP3K8,STAT3,RAC1,JUN,MAP3K7,MAP3K5,MAPK3,PIK3R1,MAPK8,MAPK9,PRKCH,FOS,PRKCA,PRKCB,PRKCE,PRKCD,PRKCG,PRKCZ,PRKCQ,CCND1,PLCG1,MAP3K11,GRB2,PAK1,CDC42	
Regulation of IL-2 Expression in Activated	1.26E-13	NFATC3,PPP3CC	SMAD4,FYN,NFKBIA,SMAD2,SMAD3,IKBKB,PLCG2,PLCG1,MAP2K7,MAP2K4,RAF1,RAC1,CALM1 (includes others),MAPK3,NFKB1,MAPK8,MAPK9,TGFBR1,FOS,CHUK,JUN,BCL10,RELA,VAV2,GRB2	CD3G,ZAP70,CD3E
Erythropoietin Signaling	3.98E-13	AKT1,AKT3,PRKD1	SRC,RPS6KB1,NFKBIA,PLCG2,PLCG1,RAF1,JUN,MAPK3,JAK2,PIK3R1,NFKB1,RELA,PRKCH,FOS,PRKCA,PRKCB,PRKCE,PRKCD,PRKCG,PRKCZ,PRKCQ,AKT2,GRB2,PDPK1	
Acute Phase Response Signaling	3.98E-13	TCF4,AKT3,TNF,AKT1	HMOX1,NFKBIA,RIPK1,AKT2,MAP2K7,TRADD,MAP2K4,RAF1,A2M,STAT3,TCF3,JUN,MAP3K7,APOH,MAP3K5,MAPK3,JAK2,PIK3R1,NFKB1,RELA,IRAK1,TRAF2,TRAF6,FOS,MAPK8,CHUK,MTOR,ECSIT,FTL,MAPK14,MAPK9,TNFRSF1B,TNFRSF1A,IKBKB,ALB,GRB2,PDPK1	C2,OSMR,CRABP1,CRABP2
p53 Signaling	3.98E-13	AKT1,AKT3,STAG1	HDAC1,BCL2L1,CDKN1A,KAT2B,PRKDC,CTNNB1,AKT2,ST13,JUN,PIK3R1,MAPK8,CHEK1,BRCA1,SIRT1,TRIM29,MDM2,EP300,MDM4,MAPK14,TP53,TOPBP1,CCND1,GSK3B,PTEN,PIAS1,BCL2	SNAI2,CCND2,TP73,TP53BP2
RANK Signaling in Osteoclasts	6.31E-13	AKT1,AKT3,PPP3CC	TNFSF11,CALM1 (includes others),NFKBIA,IKBKB,PTK2B,MAP2K7,MAP2K4,RAF1,MAP3K8,JUN,MAP3K7,MAP3K5,MAPK3,PIK3R1,NFKB1,MAPK8,MAPK9,SRC,TRAF2,TRAF6,FOS,CHUK,AKT2,RELA,MAPK14,XIAP,MAP3K11	MITF

IL-12 Signaling and Production in Macroph	6.31E-13	AKT1,AKT3,APOE,TNF,PRKD1	ALB,IKBKB,AKT2,MAP2K4,MAP3K8,STAT1,JUN,MAPK3,PIK3R1,NFKB1,MAPK8,MAPK9,PRKCH,TRAF6,FOS,IFNG,PRKCB,PRKCE,CHUK,PRKCG,PRKCD,PRKCZ,PRKCA,PRKCQ,CLU,EP300,RELA,MAPK14,MST1R	PCYOX1,APOC4,APOC2,APOL1,APOC1
Aryl Hydrocarbon Receptor Signaling	6.31E-13	TNF	CCNE1,HSPB7,NR2F1,CDKN1A,SMARCA4,NCOR2,RARA,CCNA2,FOS,JUN,HSP90AB1,MAPK3,CDKN1B,NFKB1,RELA,CHEK1,SRC,HSPB1,SP1,NEDD8,MCM7,TP53,MDM2,EP300,MAPK8,ESR2,ESR1,CCND1,CCND3,PTGES3,CDK6,HSP90AA1	ALDH3A2,CCND2,NQO2,TP73,AHRR,ALDH6A1,ALDH1B1
Apoptosis Signaling	7.94E-13	TNF	BCL2L1,SPTAN1,NFKBIA,IKBKB,PLCG2,BAD,MAP2K7,MAP2K4,RAF1,BCL2L11,MAP3K5,MAPK3,NFKB1,MAPK8,PRKCA,ACIN1,PRKCE,CHUK,PARP1,PRKCQ,TNFRSF1B,RELA,TP53,CASP3,TNFRSF1A,XIAP,PLCG1,CASP9,CDK1,BCL2	
UVC-Induced MAPK Signaling	1.00E-12	PRKD1	SRC,PRKCH,TP53,FOS,PRKCA,JUN,PRKCB,PRKCE,PRKCD,PRKCG,MAPK14,MAPK3,PRKCZ,BRAF,MAP2K4,PRKCQ,MAPK8,MAPK9,RAF1,EGFR	
CD28 Signaling in T Helper Cells	1.00E-12	AKT1,AKT3,NFATC3,PPP3CC	JUN,NFKBIA,IKBKB,PLCG1,MAP2K4,RAC1,FYN,BCL10,PIK3R1,NFKB1,RELA,MAPK9,FOS,CHUK,PRKCQ,ITPR3,ITPR1,MAPK8,SYK,ARPC1B,CALM1 (includes others),AKT2,GRB2,PDPK1,HLA-DMB,PAK1,CDC42	ARPC3,LCP2,CD3G,ZAP70,CD3E
NGF Signaling	1.26E-12	AKT1,AKT3	IKBKB,PLCG2,AKT2,MAP2K4,RAF1,MAP3K8,RPS6KB1,MAP3K7,MAP3K5,MAPK3,PIK3R1,NFKB1,RELA,MAPK9,TRIO,TRAF6,RAC1,PRKCD,CREB1,CHUK,PRKCZ,ATF4,EP300,MAPK8,RPS6KA3,TP53,PLCG1,MAP3K11,GRB2,PDPK1,CREBBP,CDC42	

Small Cell Lung Cancer Signaling	2.00E-12	AKT1,AKT3	BCL2L1,PTGS2,CDKN1B,NFKBIA,IKBKB,AKT2,ABL1,SKP2,CCNE1,CKS1B,PIK3R1,NFKB1,RELA,SIN3A,TRAF2,TRAF6,SUV39H1,CHUK,TP53,CCND1,MAX,CASP9,PTEN,CDK6,BCL2	
Î±-Adrenergic Signaling	2.51E-12	PRKD1	CALM1 (includes others),PLCG2,PLCG1,RAF1,GNAI3,GNAI1,GNAI2,GNG10,GNB2L1,MAPK3,PRKCH,GNB5,GNB1,PRKCB,PRKCE,PRKCD,PRKCG,PRKCZ,PRKCA,PRKCQ,ITPR3,ITPR1,ADCY6,GNAQ,PRKACA	PRKAG1,PRKAR1B,ADCY10,GNAS
Role of Tissue Factor in Cancer	3.16E-12	AKT1,AKT3	BCL2L1,PLCB1,RPS6KB1,LIMK1,EGFR,MAPK14,PTK2B,EGR1,FYN,MAPK3,JAK2,PIK3R1,LYN,SRC,ITGB3,RAC1,PRKCA,PDIA2,AKT2,MTOR,CASP3,RPS6KA3,TP53,GNAQ,CSF1,PTEN,GNA11,VEGFA,PAK1,CDC42	HCK,CSF2
TNFR1 Signaling	3.98E-12	PAK6,TNF	IKBKB,TRAF2,PAK1,CASP3,FOS,NFKBIA,JUN,CHUK,TRADD,CASP9,RIPK1,XIAP,TNFAIP3,MAP2K4,PAK4,NFKB1,MAPK8,CDC42,RELA,TNFRSF1A	
Cyclins and Cell Cycle Regulation	3.98E-12	NONE	HDAC1,PPP2R1A,HDAC2,HDAC5,HDAC4,HDAC7,CDKN1B,CDKN1A,ABL1,RAF1,SKP2,CCNE1,PPP2R4,SIN3A,CDKN2C,SUV39H1,CDC25A,CCNA2,TP53,PPP2CA,CCND3,GSK3B,CDK1,CDK6,CDK7,CCND1	E2F6,CCND2
VDR/RXR Activation	3.98E-12	PRKD1	PRKCH,TNFSF11,PPARD,SEMA3B,PRKCA,CDKN1B,CDKN1A,PRKCB,PRKCE,PRKCD,PRKCG,IFNG,FOXO1,PRKCZ,SPP1,CSNK2A1,SP1,PRKCQ,EP300,NCOR2,MXD1	VDR,YY1,CSF2,HOXA10,CASR,SULT2A1
Prolactin Signaling	5.01E-12	PRKD1	FYN,PLCG2,PLCG1,RAF1,STAT3,STAT1,JUN,MAPK3,JAK2,PIK3R1,PRKCH,FOS,PRKCA,SP1,PRKCB,PRKCE,PRKCD,PRKCG,PRKCZ,PRKCQ,EP300,IRS1,GRB2,PDPK1,CREBBP	NMI

GPCR-Mediated Nutrient Sensing in Enter	5.01E-12	PLCH2,PRKD1	PRKCH,ADCY6,GNAQ,PLCB1,PRKCA,PRKCE,PRKCB,GNG10,PRKCD,PRKCG,PLCG2,PLCG1,PRKCZ,PRKACA,GNA11,PRKCQ,ITPR3,ITPR1,GNAI2,GNAI3,GNAI1	ADCY10,GNAS,PRKAG1,PDIA3,PRKAR1B,CASR
Hypoxia Signaling in the Cardiovascular S	1.00E-11	AKT1	JUN,NFKBIA,UBE2D2,UBE2D3,UBE2D1,VHL,CDC34,HSP90AB1,NOS3,UBE2E1,CREB1,UBE2E2,VEGFA,UBE2E3,ATF4,TP53,MDM2,EP300,COPS5,UBE2M,PTEN,HSP90AA1,UBE2S,CREBBP	
Non-Small Cell Lung Cancer Signaling	1.00E-11	AKT1,AKT3	EGFR,FOXO3,BAD,ABL1,RAF1,EGF,STK4,MAPK3,PIK3R1,SIN3A,ERBB2,SUV39H1,AKT2,PRKCA,ITPR3,ITPR1,TP53,CCND1,PLCG1,CASP9,GRB2,CDK6,PDPK1	
fMLP Signaling in Neutrophils	1.00E-11	NFATC3,PRKD1,PPP3CC	PLCB1,CALM1 (includes others),NFKBIA,GNAI2,GNAI3,GNAI1,RAF1,GNG10,GNB2L1,MAPK3,PIK3R1,NFKB1,RELA,PRKCH,RAC1,GNB5,GNB1,PRKCB,PRKCE,PRKCD,PRKCG,PRKCZ,PRKCA,PRKCQ,ITPR3,ITPR1,ARPC1B,CDC42	ARPC3,GNAS
ErbB4 Signaling	1.00E-11	NRG1,AKT1,PRKD1	PRKCH,ERBB4,MAPK3,PRKCA,PSEN2,PSEN1,PRKCB,PRKCE,PRKCD,PRKCG,GRB2,PLCG2,PLCG1,PRKCZ,PIK3R1,ADAM17,PRKCQ,PDPK1,RAF1,YAP1	APH1B
HIPPO signaling	1.00E-11	NONE	PPP2R1A,SMAD4,SMAD1,SMAD2,SMAD3,PRKCZ,DLG1,DLG3,DLG2,SKP2,DLG4,YWHAQ,PPP2R4,NF2,YWHAB,YWHAG,PPP1R14A,YWHAZ,STK3,STK4,YAP1,PPP1CC,PPP1CA,PPP2CA,INADL,YWHAE	PPP1R11,CD44,TP53BP2
IL-6 Signaling	1.58E-11	AKT1,AKT3,TNF	NFKBIA,IKBKB,AKT2,MAP2K7,MAP2K4,RAF1,A2M,STAT3,FOS,JUN,MAP3K7,MAPKAPK2,MAPK3,JAK2,PIK3R1,NFKB1,RELA,MAPK9,HSPB7,TRAF2,TRAF6,HSPB1,CHUK,VEGFA,MAPK8,CSNK2A1,MAPK14,TNFRSF1B,TNFRSF1A,GRB2,CSNK2B	

Type I Diabetes Mellitus Signaling	1.58E-11	TNF	NFKBIA,CPE,IKBKB,MAP2K7,MAP2K4,CASP9,RIPK1,STAT1,MAP3K7,TRADD,MAP3K5,JAK2,JAK1,NFKB1,MAPK8,IRAK1,TRAF2,TRAF6,IFNG,CHUK,RELA,MAPK14,MAPK9,TNFRSF1B,CASP3,TNFRSF1A,INS,PIAS1,HLA-DMB,BCL2	CD3E,CD3G
Relaxin Signaling	2.00E-11	AKT1,AKT3	GNG10,NFKBIA,AKT2,GNAI2,GNAI3,GNAI1,GNB2L1,JUN,PDE3B,APEX1,MAPK3,PDE7B,PIK3R1,GNAO1,NFKB1,RELA,NOS3,FOS,GNB5,GNB1,CREB1,PRKCZ,VEGFA,TDP2,ADCY6,GNAQ,GNAZ,PRKACA,BRAF,GNA11,PDE4B	PRKAG1,PRKAR1B,ADCY10,GNAS
Wnt/Î²-catenin Signaling	2.51E-11	TCF4,AKT3,AKT1	HDAC1,PPP2R1A,GNAO1,TLE1,ILK,TLE4,CTNNB1,AKT2,PIN1,RARA,JUN,MAP3K7,PPP2R4,SRC,TGFBR1,TCF3,APPL1,PPARD,MDM2,EP300,CSNK2A1,CSNK1A1,LRP1,TP53,GNAQ,PPP2CA,GSK3A,GSK3B,UBD,CCND1,UBC,APC,CSNK2B,CREBBP,WNT7B	CD44,DKK4,FZD3,ACVR1B
Synaptic Long Term Depression	2.51E-11	PLCH2,PRKD1,GRM3	PPP2R1A,PLCB1,GRIA2,GNAO1,LCAT,PLCG2,PLCG1,GNAI2,GNAI3,GNAI1,RAF1,RYR2,PPP2R4,MAPK3,LYN,PRKCH,IGF1R,NOS3,PRKCA,PRKCB,PRKCE,PRKCD,PRKCG,PRKCZ,PRKCQ,ITPR3,ITPR1,GNAQ,PPP2CA,GNAZ,GNA11	PLA2G6,PLA2G10,PDIA3,GNAS
Cholecystokinin/Gastrin-mediated Signalin	3.16E-11	TNF,PRKD1	PTGS2,SRC,EGFR,MAPK14,PTK2B,MAP2K4,RAF1,JUN,MAPK3,MAPK8,MAPK9,PRKCH,FOS,PRKCA,PLCB1,PRKCB,PRKCE,PRKCD,PRKCG,RHOJ,PRKCZ,PRKCQ,ITPR3,ITPR1,BCAR1,GNAQ,GRB2	CREM,DIRAS3
Cell Cycle: G2/M DNA Damage Checkpoir	3.98E-11	NONE	YWHAZ,BRCA1,TP53,YWHAE,CDKN1A,YWHAQ,YWHAB,CKS1B,PRKDC,KAT2B,CDK1,PRKCZ,AURKA,CDK7,ABL1,SKP2,MDM2,EP300,MDM4,YWHAG,CHEK1	

Regulation of the Epithelial-Mesenchymal	3.98E-11	NOTCH4,TCF4,AKT3,AKT1	EGR1,SMAD4,ADAM17,EGFR,SMAD2,SMAD3,CTNNB1,AKT2,MAP2K7,MAP2K4,RAF1,EGF,STAT3,MAML1,TWIST1,PSEN2,RBPJ,TWIST2,MAPK3,JAK2,JAK3,PIK3R1,JAK1,FGF12,RELA,TGFBR1,TCF3,NFKB1,ID2,NOTCH1,GSK3B,GRB2,BRAF,APC,PSEN1,WNT7B	SNAI2,APH1B,FZD3,FGFR1
Xenobiotic Metabolism Signaling	3.98E-11	TNF,PRKD1,CUL3	PPP2R1A,HDAC5,CAMK1D,MAPK8,MAPK14,MAP2K7,MAP2K4,RAF1,NCOR2,HDAC4,MAP3K8,KEAP1,MAP3K7,PPP2R4,MAP3K5,HSP90AB1,MAPK3,PIK3R1,NFKB1,RELA,MAPK9,PRKCH,PRKCA,PRKCB,PRKCE,CAMK2A,CAMK2B,PRKCD,PRKCZ,PRKCG,RBX1,PRKCQ,EP300,FTL,CYP2C8,PTGES3,PPP2CA,DNAJC7,HMOX1,MAP3K11,GRIP1,HSP90AA1,CREBBP	UGT2B4,CHST12,HS3ST1,ALDH3A2,NQO2,AHRR,ALDH6A1,SRA1,ALDH1B1,SULT2A1
Calcium-induced T Lymphocyte Apoptosis	5.01E-11	PPP3CC,PRKD1,ATP2A2	HDAC1,PRKCH,HDAC2,CABIN1,PRKCA,CALM1 (includes others),NR4A1,PRKCE,PRKCD,PRKCG,ITPR1,PRKCB,PLCG1,PRKCZ,PRKCQ,ITPR3,EP300,HLA-DMB	CD3G,ZAP70,CD3E
Thrombopoietin Signaling	7.94E-11	PRKD1	PRKCH,PRKCE,PRKCB,STAT3,STAT1,MAPK3,PRKCA,JUN,FOS,GAB2,PRKCD,PRKCG,GRB2,PLCG2,PLCG1,PRKCZ,JAK2,IRS2,PIK3R1,PRKCQ,RAF1	
PKCÎ¸ Signaling in T Lymphocytes	1.29E-10	NFATC3,PPP3CC	FYN,NFKBIA,IKBKB,PLCG2,PLCG1,MAP2K4,MAP3K8,RAC1,JUN,MAP3K7,MAP3K5,MAPK3,PIK3R1,NFKB1,RELA,FOS,MAPK8,CHUK,CAMK2B,CAMK2A,PRKCQ,BCL10,VAV2,MAP3K11,GRB2,HLA-DMB,POU2F1	LCP2,CD3G,ZAP70,CD3E
Nitric Oxide Signaling in the Cardiovascula	1.35E-10	AKT1,AKT3,PRKD1,ATP2A2,CACNA1C	PRKAA1,AKT2,RYR2,CALM1 (includes others),PDE3B,HSP90AB1,MAPK3,PIK3R1,PRKCH,NOS3,PRKCA,PRKCB,PRKCE,PRKCD,PRKCG,PRKCZ,VEGFA,PLN,PRKCQ,ITPR3,ITPR1,PRKACA,HSP90AA1	PRKAG1,PRKAR1B

Mouse Embryonic Stem Cell Pluripotency	1.70E-10	TCF4,AKT3,AKT1	SMAD9,SMAD4,SMAD1,MAPK14,XIAP,ID1,RAF1,STAT3,MAP3K7,MAPK3,JAK2,JAK3,PIK3R1,JAK1,TCF3,AKT2,CTNNB1,TP53,ID4,ID2,ID3,GSK3B,GRB2,APC,CREBBP	FZD3
Ephrin B Signaling	1.95E-10	NONE	EFNB1,KALRN,LIMK1,CTNNB1,GNAI2,GNAI3,GNAI1,ITSN1,GNG10,GNB2L1,MAPK3,RGS3,GNAO1,EPHB6,RAC1,GNB5,GNB1,GNAQ,NCK2,VAV2,GNAZ,GNA11,PAK1,CDC42	GNAS
NRF2-mediated Oxidative Stress Respons	2.34E-10	AKT1,PRKD1,CUL3	ACTG1,CDC34,JUNB,MAPK14,FKBP5,MAP2K7,MAP2K4,RAF1,DNAJB1,JUN,MAP3K7,MAP3K5,MAPK3,PIK3R1,MAPK8,MAPK9,PRKCH,FOS,PRKCA,PRKCB,PRKCE,PRKCD,UBE2E3,PRKCZ,PRKCG,ATF4,PRKCQ,EP300,FTL,DNAJC8,SCARB1,DNAJC7,HMOX1,GSK3B,KEAP1,RBX1,CREBBP	NQO2,DNAJC10
Adipogenesis pathway	2.51E-10	AKT1,TNF	HDAC1,HDAC2,HDAC5,HDAC4,HDAC7,ERCC3,SMAD1,SMAD3,FOXO1,EZH2,RUNX1T1,SMAD9,EGR2,RPS6KB1,NR2F2,SIN3B,SETDB1,SIN3A,SIRT1,TP53,TNFRSF1A,RBBP7,RBBP4,CDK5,CDK7,CTBP1,FBXW7	GTF2H2,FGFR1,FZD3,SAP30L,CEBPD
Germ Cell-Sertoli Cell Junction Signaling	2.63E-10	AKT1,PAK6,TNF	ACTG1,LIMK1,ILK,MAPK14,MAP2K7,MAP2K4,A2M,MAP3K8,ACTN2,BCAR1,MAP3K11,TUBA1B,MAP3K7,MAP3K5,MAPK3,JUP,PIK3R1,MAPK8,MAPK9,SRC,TGFBR1,RAC1,EPN2,RHOJ,TUBB2A,CTNNB1,ACTN1,TNFRSF1A,TUBB,KEAP1,PAK4,PDPK1,PAK1,CDC42	TUBA1C,DIRAS3

mTOR Signaling	2.82E-10	AKT1,AKT3,PRKD1	PPP2R1A,PRKAA2,PRKAA1,AKT2,EIF3F,RICTOR,TSC2,TSC1,EIF3H,RPS6KB1,PPP2R4,EIF3G,MAPK3,AKT1S1,PIK3R1,PRKCH,PRKAB2,RPS20,RAC1,PRKCA,PRKCB,PRKCE,PRKCD,PRKCG,RHOJ,PRKCZ,MTOR,PRKCQ,RPS6KA3,VEGFA,PPP2CA,HMOX1,IRS1,INS,EIF4EBP1,PDPK1,RHEB	PRKAG1,DIRAS3,PLD3
Signaling by Rho Family GTPases	4.47E-10	PAK6	ACTG1,JUN,LIMK1,ARFIP2,PTK2B,MAP2K7,MAP2K4,GNAI2,GNAI3,GNAI1,RAF1,RAC1,NEDD4,ARHGEF1,GNB2L1,MAPK3,CIT,PIK3R1,GNAO1,NFKB1,RELA,MAPK9,GNG10,ARHGEF12,FOS,GNB5,GNB1,RDX,CDC42EP4,RHOJ,PRKCZ,MAPK8,GNAQ,ARPC1B,GNAZ,VIM,MAP3K11,GNA11,PAK4,BAIAP2,PAK1,CDC42	DIRAS3,GNAS,ARPC3,SEPT5,SEPT3,MYL9
CCR3 Signaling in Eosinophils	4.57E-10	PAK6,PRKD1	PLCB1,CALM1 (includes others),LIMK1,MAPK14,RAF1,GNAI3,GNAI1,GNAI2,GNG10,GNB2L1,MAPK3,PIK3R1,PRKCH,RAC1,GNB5,GNB1,PRKCB,PRKCE,PRKCD,PRKCG,PRKCZ,PRKCA,PRKCQ,ITPR3,ITPR1,PAK4,PAK1	PLA2G6,PLA2G10,GNAS
TGF-Î² Signaling	4.79E-10	NONE	HDAC1,SMAD9,SMAD4,SMAD7,SMAD1,SMAD2,SMAD3,MAPK14,BMPR1B,MAP2K4,SKI,RAF1,JUN,MAP3K7,MAPK3,MAPK8,MAPK9,TGFBR1,TRAF6,FOS,EP300,GRB2,BCL2,CREBBP,CDC42	VDR,ACVR1B
CD40 Signaling	4.90E-10	NONE	PTGS2,PTGS1,NFKBIA,IKBKB,MAP2K7,MAP2K4,MAPKAPK2,STAT3,JUN,MAP3K7,MAPK3,JAK3,PIK3R1,NFKB1,MAPK8,MAPK9,TRAF2,TRAF6,FOS,CHUK,RELA,MAPK14,TNFAIP3	
Telomerase Signaling	5.13E-10	AKT1,AKT3	HDAC1,PPP2R1A,HDAC2,HDAC5,HDAC4,HDAC7,CDKN1A,EGFR,POT1,AKT2,ABL1,RAF1,EGF,PPP2R4,HSP90AB1,MAPK3,TERF1,PIK3R1,TERT,SP1,TERF2IP,PDPK1,TP53,PPP2CA,PTGES3,GRB2,HSP90AA1	

Induction of Apoptosis by HIV1	5.37E-10	TNF	BCL2L1,IKBKB,TRAF2,TNFRSF1B,TP53,TNFRSF1A,CASP9,NFKBIA,CHUK,TRADD,MAP3K5,RIPK1,XIAP,MAP2K7,BCL2,MAP2K4,NFKB1,RELA,MAPK9,MAPK8,CASP3	
Ovarian Cancer Signaling	6.17E-10	TCF4,AKT3,AKT1	PTGS2,PTGS1,VEGFA,EGFR,CTNNB1,AKT2,ABL1,RAF1,EGF,RPS6KB1,MAPK3,PIK3R1,SIN3A,BRCA1,SRC,TCF3,SUV39H1,MTOR,TP53,CCND1,GSK3B,PTEN,PRKACA,BRAF,APC,WNT7B,BCL2	PRKAG1,CD44,FZD3,PRKAR1B
PDGF Signaling	7.24E-10	NONE	PLCG2,PLCG1,MAP2K4,ABL1,RAF1,STAT3,STAT1,JUN,MAPK3,JAK2,JAK3,PIK3R1,JAK1,MAPK8,SRC,FOS,PRKCA,PRKCB,RASA1,CSNK2A1,INPP5K,GRB2,CSNK2B	PDGFB,INPPL1
Reelin Signaling in Neurons	1.32E-09	AKT1,APOE	VLDLR,ARHGEF12,MAP2K7,MAP2K4,FYN,ARHGEF1,PIK3R1,LYN,MAPK9,SRC,ITGB3,MAPK8,NDEL1,PAFAH1B1,MAPK8IP1,MAPK8IP2,APP,GSK3B,MAP3K11,CDK5,MAPT,LRP8	HCK
T Cell Receptor Signaling	1.45E-09	NFATC3,PPP3CC	FYN,NFKBIA,IKBKB,PLCG1,MAP2K4,RAF1,RAC1,CALM1 (includes others),RASA1,MAPK3,PIK3R1,NFKB1,MAPK8,FOS,CHUK,JUN,PRKCQ,BCL10,RELA,VAV2,GRB2,BTK	LCP2,CD3G,ZAP70,CD3E
ERK5 Signaling	1.58E-09	AKT1	YWHAZ,SRC,MAP3K8,WNK1,GNAQ,RPS6KA3,FOS,RPS6KB1,YWHAQ,EGFR,CREB1,YWHAG,FOXO3,BAD,PRKCZ,YWHAB,ATF4,EP300,EGF,CREBBP,YWHAE	
Natural Killer Cell Signaling	1.74E-09	AKT1,PAK6,AKT3,PRKD1	VAV2,PLCG2,AKT2,RAF1,FYN,MAPK3,PIK3R1,PRKCH,RAC1,PRKCA,PRKCB,PRKCE,PRKCD,PRKCG,PRKCZ,PRKCQ,NCK1,SYK,INPP5K,PLCG1,GRB2,PAK4,PAK1	LCP2,ZAP70,INPPL1

Semaphorin Signaling in Neurons	1.82E-09	PAK6,FES	ARHGEF12,DPYSL5,DPYSL2,DPYSL3,RAC1,PLXNA1,LIMK1,FYN,PAK1,RHOJ,CDK5,PAK4,MAPK3	RND1,SEMA7A,ARHGAP1,DIRAS3,NRP1
Death Receptor Signaling	1.91E-09	TNF	TNKS,ACTG1,SPTAN1,LIMK1,RIPK1,XIAP,MAP2K7,MAP2K4,TRADD,MAP3K5,NFKB1,RELA,HSPB7,TRAF2,NFKBIA,HSPB1,ACIN1,CHUK,PARP1,MAPK8,IKBKB,TNFRSF1B,CASP3,TNFRSF1A,CASP9,BCL2	
Growth Hormone Signaling	1.91E-09	PRKD1	PLCG2,PLCG1,A2M,STAT3,STAT1,RPS6KB1,MAPK3,JAK2,PIK3R1,PRKCH,IGF1R,FOS,PRKCA,PRKCB,PRKCE,PRKCD,PRKCG,PRKCZ,PRKCQ,RPS6KA3,IRS1,PDPK1	
Calcium Signaling	2.04E-09	NFATC3,ATP2A2,CHRNA5,CHRNA7,CHRNA3,PPP3CC,GRIN2A	HDAC1,HDAC2,HDAC5,CAMK1D,HDAC7,GRIA2,CALM1 (includes others),HDAC4,EP300,CABIN1,RYR2,TPM3,CHRNB4,TPM1,GRIN3A,MAPK3,GRIN3B,GRIN1,CAMK2A,CAMK2B,CREB1,ITPR3,ITPR1,CALR,CAMKK1,PRKACA,ATF4,CREBBP	PRKAG1,ATP2B1,ASPH,PRKAR1B,MYL9
IL-17A Signaling in Airway Cells	2.19E-09	AKT1,AKT3	IKBKB,RELA,STAT3,TRAF6,PTEN,NFKBIA,GSK3B,MAP3K7,CHUK,MAPK14,MAPK3,AKT2,JAK2,JAK3,PIK3R1,MAP2K4,NFKB1,MAPK8,MAPK9,JAK1	
Pyridoxal 5'-phosphate Salvage Pathway	2.19E-09	NONE	LIMK1,PRKAA2,PRKAA1,AKT2,MAP2K4,MAP3K8,NEK2,MAPK3,MAPK8,MAPK9,BRAF,PRKCH,PRKCE,PRKCD,PRKCQ,IRAK1,CSNK1A1,CDK1,CDK5,CDK6,CDK7,PAK1	
VEGF Family Ligand-Receptor Interaction	2.95E-09	AKT1,AKT3,PRKD1	PRKCH,NOS3,FOS,PRKCA,PRKCB,PRKCE,PRKCD,PRKCG,AKT2,PLCG2,PLCG1,GRB2,VEGFA,PIK3R1,PRKCQ,RAF1,MAPK3,PRKCZ	PLA2G6,PLA2G10,NRP1

PPAR Signaling	3.24E-09	TNF	PTGS2,NR2F1,NFKBIA,IKBKB,RAF1,NCOR2,JUN,MAP3K7,HSP90AB1,MAPK3,NFKB1,RELA,TRAF2,TRAF6,FOS,CHUK,PPARD,EP300,TNFRSF1B,TNFRSF1A,INS,GRB2,HSP90AA1,CREBBP	PDGFB,SRA1
Role of PKR in Interferon Induction and Ar	3.47E-09	AKT1,TNF	IKBKB,TRAF2,STAT1,TP53,CASP3,TRAF6,TNFRSF1A,IFNG,NFKBIA,MAP3K7,CASP9,MAPK14,CHUK,NFKB1,RELA	
Chemokine Signaling	3.63E-09	NONE	CAMK1D,SRC,CALM1 (includes others),LIMK1,MAPK14,PLCG2,PTK2B,RAF1,GNAI3,GNAI1,GNAI2,JUN,MAPK3,MAPK8,PLCB1,FOS,PRKCA,PRKCB,CAMK2A,CAMK2B,GNAQ,PLCG1	CCL4
Renal Cell Carcinoma Signaling	3.63E-09	AKT1,PAK6,AKT3	FOS,TCEB1,RAC1,JUN,PAK1,UBD,VHL,AKT2,GRB2,VEGFA,PIK3R1,UBC,PAK4,RBX1,EP300,RAF1,MAPK3,CREBBP,CDC42	PDGFB
Virus Entry via Endocytic Pathways	4.27E-09	PRKD1	ACTG1,SRC,PLCG2,PLCG1,DNM2,ABL1,AP2B1,ITSN1,FYN,PIK3R1,PRKCH,ITGB3,RAC1,PRKCA,PRKCB,PRKCE,PRKCD,PRKCG,PRKCZ,AP2A2,PRKCQ,FLNA,FOLR1,CDC42	CXADR
cAMP-mediated signaling	4.27E-09	DRD4,DRD2,DRD3,RGS4,PPP3CC,GRM3	CAMK1D,GNAO1,AKAP13,GABBR1,PTGER3,GNAI2,GNAI3,GNAI1,ADORA2A,STAT3,ADCY6,RAF1,S1PR1,CALM1 (includes others),PDE3B,APEX1,MAPK3,PDE7B,RGS2,GLP1R,SRC,CAMK2A,CREB1,CAMK2B,EP300,TDP2,AKAP6,AKAP9,PRKACA,BRAF,ATF4,PDE4B,CREBBP	ADCY10,GNAS,HCAR3,RGS7,CREM,PRKAR1B
iCOS-iCOSL Signaling in T Helper Cells	4.79E-09	AKT1,AKT3,NFATC3,PPP3CC	RAC1,GAB2,NFKBIA,CALM1 (includes others),CAMK2A,CAMK2B,CHUK,IKBKB,PTEN,PLCG1,GRB2,BAD,PIK3R1,ITPR3,PRKCQ,NFKB1,RELA,ITPR1,HLA-DMB,PDPK1,AKT2	LCP2,CD3G,ZAP70,CD3E
JAK/Stat Signaling	4.90E-09	AKT1,AKT3	BCL2L1,STAT3,GNAQ,STAT1,FOS,GRB2,CDKN1A,JUN,PIAS2,MAPK3,AKT2,PIAS1,JAK2,JAK3,PIK3R1,JAK1,MTOR,NFKB1,RELA,RAF1,PTPN1	

Leukocyte Extravasation Signaling	5.13E-09	PRKD1	MMP17,ACTG1,SRC,CLDN19,CTNNB1,PLCG2,PTK2B,MAP2K4,ABL1,GNAI2,GNAI3,GNAI1,ACTN2,PIK3R1,MAPK8,MAPK9,DLC1,PRKCH,ITGB3,RAC1,PRKCA,PRKCB,PRKCE,RDX,PRKCG,PRKCD,PRKCZ,PRKCQ,BCAR1,F11R,MAPK14,ACTN1,VAV2,PLCG1,ARHGAP12,BTK,CDC42	CD44,CLDN18,MMP23B,ARHGAP1
Hepatic Cholestasis	5.13E-09	TNF,PRKD1	CNTF,NFKBIA,IKBKB,MAP2K4,RARA,JUN,MAP3K7,PRKCA,NFKB1,RELA,IRAK1,PRKCH,TRAF2,TRAF6,IFNG,PRKCB,PRKCE,PRKCD,PRKCG,CHUK,PRKCZ,PRKCQ,MAPK8,ADCY6,MAPK9,TNFRSF1B,TNFRSF1A,ESR1,INS,PRKACA	PRKAG1,CSF2,PRKAR1B,ADCY10,GNAS
Neurotrophin/TRK Signaling	5.89E-09	AKT1,BDNF	NTF3,FOS,ATF4,EP300,JUN,NTRK2,NTRK3,MAPK3,PLCG1,GRB2,MAP2K7,CREB1,PIK3R1,MAP2K4,PDPK1,MAPK8,MAP3K5,RAF1,CREBBP,CDC42	
Amyloid Processing	5.89E-09	AKT1,AKT3	CSNK1A1,PSEN2,APP,PRKCE,GSK3B,MAPK14,MAPK3,AKT2,PRKACA,CDK5,MAPT,CSNK2B,PSEN1,CSNK2A1	PRKAG1,APH1B,PRKAR1B
NF-ÎºB Activation by Viruses	6.61E-09	AKT1,AKT3,PRKD1	PRKCH,TRAF2,PRKCB,PRKCA,NFKBIA,PRKCE,ITGB3,PRKCG,PRKCD,IKBKB,MAPK3,AKT2,PRKCZ,CHUK,PIK3R1,PRKCQ,NFKB1,RELA,RAF1,RIPK1	
Clathrin-mediated Endocytosis Signaling	6.61E-09	SNAP91,APOE,PPP3CC	ACTG1,SNX9,DNM2,EGF,EPS15,AP2B1,RAB11A,PIK3R1,FGF12,SRC,ITGB3,RAC1,HGS,LDLR,VEGFA,AP2A2,CLU,MDM2,CSNK2A1,AMPH,ARPC1B,ALB,INS,UBD,GRB2,UBC,CSNK2B,CDC42	PDGFB,RAB5C,PCYOX1,ARPC3,APOC4,APOC2,APOC1,STAM,APOL1
GM-CSF Signaling	6.92E-09	AKT1,AKT3,PPP3CC	CSF2RB,BCL2L1,STAT3,STAT1,CCND1,PRKCB,CAMK2A,CAMK2B,GNB2L1,MAPK3,AKT2,GRB2,JAK2,PIK3R1,LYN,RAF1	HCK,CSF2
ErbB2-ErbB3 Signaling	7.94E-09	NRG1,AKT1	ERBB2,ERBB3,STAT3,MAPK3,CCND1,JUN,GSK3A,BAD,GSK3B,PTEN,FOXO1,GRB2,JAK3,PIK3R1,SP1,CDKN1B,PDPK1,RAF1	

GDNF Family Ligand-Receptor Interaction	8.13E-09	NONE	MAPK3,MAPK9,NCK1,FOS,DOK1,RAC1,JUN,IRS1,CREB1,PLCG2,PLCG1,GRB2,IRS2,PIK3R1,MAP2K4,ITPR3,MAPK8,ITPR1,RAF1,RASA1,CDC42	GFRA2
p38 MAPK Signaling	8.32E-09	TNF	TGFBR1,MAPK14,MAP2K4,MAPKAPK5,MAPKAPK2,STAT1,RPS6KB1,MAP3K7,TRADD,MAP3K5,IRAK1,HSPB7,TRAF2,TRAF6,HSPB1,CREB1,HIST2H3C,TNFRSF1B,EP300,RPS6KA3,TP53,TNFRSF1A,MAX,MAPT,ATF4,HIST3H3,CREBBP	PLA2G6,PLA2G10
CD27 Signaling in Lymphocytes	8.51E-09	NONE	BCL2L1,TRAF2,MAPK9,CASP3,FOS,NFKBIA,JUN,MAP3K8,CASP9,IKBKB,MAP3K11,CHUK,MAP2K7,MAP2K4,MAPK8,NFKB1,RELA,MAP3K5,MAP3K7	
nNOS Signaling in Neurons	8.91E-09	GRIN2A,PRKD1,PPP3CC	PRKCH,DLG2,DLG4,PRKCA,CALM1 (includes others),PRKCB,PRKCE,CAMK2A,PRKCG,PRKCD,GRIN3A,GRIN3B,PRKCQ,GRIN1,PRKCZ	
Leptin Signaling in Obesity	8.91E-09	PLCH2,AKT1,AKT3	PLCB1,ADCY6,STAT3,MAPK3,FOXO1,PDE3B,AKT2,PLCG2,PLCG1,GRB2,PRKACA,PIK3R1,JAK2,INS	ADCY10,GNAS,PRKAG1,GHRL,PDIA3,PRKAR1B
VEGF Signaling	9.12E-09	AKT1,AKT3	BCL2L1,ELAVL1,FOXO3,PLCG2,PLCG1,RAF1,ACTG1,EIF1AY,MAPK3,PTK2B,PIK3R1,YWHAE,SRC,NOS3,PRKCA,PRKCB,FOXO1,AKT2,VEGFA,ACTN2,ACTN1,BAD,GRB2,BCL2	
Factors Promoting Cardiogenesis in Verte	9.12E-09	TCF4,PRKD1	SMAD9,SMAD4,SMAD1,SMAD2,CTNNB1,BMPR1B,CCNE1,MAP3K7,PRKCH,TGFBR1,TCF3,PRKCA,PRKCB,PRKCE,PRKCD,PRKCG,PRKCZ,PRKCQ,MAPK14,LRP1,GSK3B,APC	FZD3,ACVR1B
Estrogen-Dependent Breast Cancer Signa	9.55E-09	AKT1,AKT3	SRC,IGF1R,TERT,FOS,CCND1,SP1,ESR1,JUN,CREB1,MAPK3,AKT2,PIK3R1,NFKB1,ATF4,RELA,EP300,CREBBP,EGFR	HSD17B12

RhoGDI Signaling	1.00E-08	PAK6	ACTG1,GNAO1,LIMK1,GNAI2,GNAI3,GNAI1,GNG10,ARHGEF1,GNB2L1,GNB5,DLC1,SRC,ARHGEF12,RAC1,PRKCA,GNB1,RDX,RHOJ,EP300,GRIP1,ESR2,GNAQ,ESR1,ARPC1B,GNAZ,ARHGAP12,GNA11,PAK4,PAK1,CREBBP,CDC42	DIRAS3,GNAS,ARPC3,CD44,ARHGAP1,MYL9
Myc Mediated Apoptosis Signaling	1.12E-08	AKT1,AKT3	YWHAZ,IGF1R,TP53,CASP3,YWHAE,YWHAQ,AKT2,CASP9,BAD,GRB2,PIK3R1,MAP2K4,YWHAB,MAPK8,MAPK9,YWHAG,PRKCZ,BCL2	
TNFR2 Signaling	1.12E-08	TNF	TRAF2,TNFRSF1B,FOS,NFKBIA,JUN,CHUK,TNFAIP3,IKBKB,XIAP,MAP2K4,NFKB1,RELA,MAPK8	
Epithelial Adherens Junction Signaling	1.26E-08	NOTCH4,TCF4,AKT3,AKT1	ACTG1,EGFR,TUBB,CTNNB1,AKT2,EGF,KEAP1,TUBA1B,JUP,SRC,TGFBR1,TCF3,RAC1,EPN2,BAIAP2,TUBB2A,ACTN2,ACTN1,ARPC1B,VAV2,NOTCH1,PTEN,APC,CDC42	ACVR1B,TUBA1C,ARPC3,SNAI2,MYL9,FGFR1
UVA-Induced MAPK Signaling	1.55E-08	PLCH2	BCL2L1,TNKS,JUN,EGFR,MAPK14,PLCG2,PLCG1,MAP2K4,STAT1,RPS6KB1,MAPK3,PIK3R1,MAPK8,MAPK9,PLCB1,FOS,PRKCA,PARP1,MTOR,RPS6KA3,TP53,CASP3,CASP9	PDIA3
Inhibition of Angiogenesis by TSP1	1.55E-08	AKT1,AKT3	TGFBR1,NOS3,TP53,CASP3,FYN,SDC2,JUN,MAPK14,AKT2,VEGFA,MAP2K4,MAPK8,MAPK9	
iNOS Signaling	1.95E-08	NONE	IKBKB,STAT1,TRAF6,FOS,IFNG,CALM1 (includes others),NFKBIA,JUN,CHUK,MAPK14,JAK2,JAK3,JAK1,NFKB1,RELA,IRAK1,CREBBP	
Sperm Motility	1.95E-08	PLCH2,PRKD1	PTK2B,PLCB1,PRKCE,PRKCH,PRKCA,LCAT,PRKCB,CALM1 (includes others),PRKCD,PRKCG,MST1R,PLCG2,PLCG1,PRKCZ,PRKACA,PRKCQ,ITPR3,MAP3K11,ITPR1,PDE4B	CACNA1H,PLA2G10,ADCY10,GNAS,PRKAG1,PDIA3,PLA2G6,PRKAR1B

Rac Signaling	3.47E-08	PAK6	JUN,LIMK1,BRK1,PTK2B,MAP2K7,MAP2K4,RAF1,RAC1,RPS6KB1,MAPK3,PIK3R1,NFKB1,MAPK8,ARFIP2,SH3RF1,PRKCZ,RELA,ARPC1B,MAP3K11,PAK4,BAIAP2,PAK1,CDC42	ARPC3,CD44,ABI2
Acute Myeloid Leukemia Signaling	3.55E-08	TCF4,AKT3,AKT1	CSF2RB,RARA,STAT3,EIF4EBP1,TCF3,CCND1,RPS6KB1,JUP,AKT2,MAPK3,BAD,GRB2,MAP2K7,BRAF,PIK3R1,MAP2K4,MTOR,NFKB1,RELA,RAF1	
Macropinocytosis Signaling	4.37E-08	PRKD1	SRC,ITGB3,PAK1,RAC1,PRKCA,PRKCB,PRKCE,PRKCD,PRKCG,CSF1,PLCG2,PLCG1,PRKCZ,PRKCH,PIK3R1,PRKCQ,INS,EGF,CDC42	PDGFB
Salvage Pathways of Pyrimidine Ribonucle	5.25E-08	NONE	LIMK1,PRKAA2,PRKAA1,AKT2,MAP2K4,MAP3K8,NEK2,MAPK3,MAPK8,MAPK9,CDK5,PRKCH,PRKCE,PRKCD,PRKCQ,NME7,CSNK1A1,NME3,CDK1,BRAF,CDK6,CDK7,IRAK1,PAK1	APOBEC1
Endometrial Cancer Signaling	5.62E-08	AKT1,AKT3	ERBB2,FOXO3,TP53,MAPK3,CCND1,ILK,GSK3B,CTNNB1,PTEN,BAD,GRB2,PIK3R1,PDPK1,CASP9,RAF1,AKT2	
HMGB1 Signaling	6.17E-08	AKT1,AKT3,TNF	KAT2B,MAPK14,AKT2,MAP2K7,MAP2K4,RAC1,JUN,MAPK3,PIK3R1,NFKB1,RELA,MAPK9,CNTF,FOS,IFNG,SP1,RHOJ,MAPK8,TNFRSF1B,TNFRSF1A,HAT1,RBBP7,KAT6A,CDC42	CSF2,DIRAS3
Integrin Signaling	7.59E-08	AKT1,PAK6,AKT3	ACTG1,ILK,PLCG2,AKT2,MAP2K4,ABL1,RAF1,NEDD9,PTEN,FYN,MAPK3,TTN,PIK3R1,MAPK8,SRC,ITGB3,RAC1,RHOJ,BCAR1,ACTN2,ACTN1,NCK1,NCK2,ARPC1B,PLCG1,GSK3B,MAP3K11,GRB2,BRAF,PAK4,PAK1,CDC42	ARPC3,PDGFB,ARF5,DIRAS3,MYL9
BMP signaling pathway	7.76E-08	NONE	SMAD9,BMPR1B,SMAD4,SMAD7,JUN,SMAD1,CREB1,MAP3K7,MAPK14,MAPK3,XIAP,GRB2,PRKACA,MAP2K4,NFKB1,RELA,MAPK9,RAF1,MAPK8,CREBBP	PRKAG1,PRKAR1B

Hereditary Breast Cancer Signaling	9.12E-08	AKT1,AKT3	HDAC1,HDAC2,HDAC5,HDAC4,HDAC7,RFC5,CDKN1A,AKT2,SMARCC1,SMARCE1,SMARCA4,CDK1,PIK3R1,CHEK1,BRCA1,SMARCB1,FANCA,BARD1,EP300,TP53,CCND1,UBD,PTEN,CDK6,UBC,CREBBP	BLM,POLR2I
April Mediated Signaling	9.55E-08	NFATC3	IKBKB,TRAF2,TRAF6,FOS,NFKBIA,JUN,CHUK,MAPK14,MAP2K7,MAP2K4,NFKB1,RELA,MAPK9,MAPK8	
Lymphotoxin Î² Receptor Signaling	1.10E-07	AKT1,AKT3	BCL2L1,TRAF2,CASP3,TRAF6,NFKBIA,CHUK,CASP9,IKBKB,MAPK3,AKT2,PIK3R1,PDPK1,NFKB1,RELA,EP300,CREBBP	
IL-15 Signaling	1.32E-07	AKT1,AKT3,TNF	BCL2L1,TRAF2,STAT3,SYK,PLCG1,MAPK14,MAPK3,AKT2,JAK2,JAK3,PIK3R1,JAK1,NFKB1,RELA,RAF1,BCL2	CSF2
STAT3 Pathway	1.74E-07	NONE	SRC,TGFBR1,STAT3,MAPK3,RAC1,CDC25A,EGFR,IGF1R,NTRK2,NTRK3,MAPK14,MAP3K11,BMPR1B,JAK2,MAP2K4,MAPK8,MAPK9,RAF1,CDKN1A,BCL2	FGFR1
Ceramide Signaling	2.14E-07	AKT1,AKT3,TNF	PPP2R1A,TNFRSF1B,FOS,PPP2CA,S1PR1,TNFRSF1A,JUN,AKT2,PPP2R4,MAPK3,BAD,PRKCZ,PIK3R1,MAP2K4,NFKB1,RELA,RAF1,MAPK8,BCL2	
B Cell Activating Factor Signaling	2.14E-07	NFATC3	IKBKB,TRAF2,TRAF6,FOS,NFKBIA,JUN,CHUK,MAPK14,MAP2K7,MAP2K4,NFKB1,RELA,MAPK9,MAPK8	
FLT3 Signaling in Hematopoietic Progenito	2.24E-07	AKT1,AKT3	RPS6KA3,STAT3,STAT1,RPS6KB1,GAB2,AKT2,CREB1,MAPK14,MAPK3,BAD,GRB2,EIF4EBP1,PIK3R1,PDPK1,MTOR,ATF4,EP300,RAF1,CREBBP	
4-1BB Signaling in T Lymphocytes	2.95E-07	NONE	IKBKB,TRAF2,NFKBIA,JUN,CHUK,MAP3K5,MAPK14,MAPK3,MAP2K4,NFKB1,RELA,MAPK9,MAPK8	
Role of MAPK Signaling in the Pathogenes	2.95E-07	AKT1,AKT3,TNF	PTGS2,CASP3,IFNG,LCAT,MAP3K5,MAPK14,MAPK3,AKT2,PRKCA,MAP2K4,MAP2K7,MAPK8,MAPK9,RAF1,BCL2	PLA2G6,PLA2G10

HIF1Î± Signaling	3.63E-07	AKT1,AKT3	EP300,APEX1,MMP17,TP53,EGLN2,TCEB1,JUN,COPS5,MAPK14,MAPK3,AKT2,NOS3,VEGFA,PIK3R1,HSP90AA1,RBX1,MAPK8,MAPK9,MDM2,CREBBP,VHL	EGLN1,MMP23B
Sphingosine-1-phosphate Signaling	3.80E-07	PLCH2,AKT1,AKT3	GNAI1,PLCB1,ADCY6,GNAQ,RAC1,S1PR1,PLCG1,CASP9,AKT2,PLCG2,PTK2B,PIK3R1,RHOJ,MAPK3,GNAI2,GNAI3,CASP3	PDGFB,DIRAS3,GNAS,CASP1,PDIA3,ADCY10
GPCR-Mediated Integration of Enteroendo	4.90E-07	PLCH2	PLCB1,ADCY6,GNAQ,ITPR3,GNA11,PLCG2,PLCG1,SSTR5,GLP1R,PRKACA,ITPR1,GNAI2,GNAI3,GNAI1	PRKAG1,PDIA3,PRKAR1B,ADCY10,GNAS
Melanocyte Development and Pigmentatio	5.37E-07	NONE	SRC,ADCY6,RPS6KA3,MAPK3,RPS6KB1,CREB1,PLCG2,PLCG1,GRB2,SH2B2,PIK3R1,BCL2,ATF4,EP300,RAF1,CREBBP,PRKACA	PRKAG1,MITF,PRKAR1B,ADCY10,GNAS
Dopamine Receptor Signaling	5.89E-07	SLC6A3,DRD4,DRD2,COMT,DRD3	PPP2R1A,ADCY6,PPP1CC,PPP1CA,PPP2CA,PCBD1,NCS1,PPP2R4,PRKACA,PPP1R14A	PRKAR1B,GNAS,PRKAG1,PPP1R11,ADCY10,SLC18A1
Cardiac Î²-adrenergic Signaling	6.31E-07	CACNA1C,ATP2A2	PPP2R1A,AKAP13,GNB2L1,RYR2,GNG10,PPP2R4,PDE3B,APEX1,PDE7B,PPP1R14A,GNB5,GNB1,ADCY6,TDP2,AKAP6,PPP1CC,PPP1CA,PPP2CA,AKAP9,PRKACA,PLN,PDE4B	PRKAG1,PPP1R11,PRKAR1B,ADCY10,GNAS
Angiopoietin Signaling	6.46E-07	AKT1,PAK6,AKT3	NOS3,NCK1,NFKBIA,CHUK,FOXO1,CASP9,IKBKB,AKT2,BAD,GRB2,PIK3R1,PAK4,NFKB1,RELA,RASA1,PAK1	
FGF Signaling	6.61E-07	AKT1,AKT3	ITPR1,STAT3,RAC1,PRKCA,AKT2,CREB1,MAP3K5,MAPK14,MAPK3,PLCG1,GRB2,PIK3R1,FGF12,ATF4,MAPK8,EP300,RAF1,MAPKAPK2,CREBBP	FGFR1
Antioxidant Action of Vitamin C	7.76E-07	PLCH2,TNF	CSF2RB,PLCB1,MAPK3,HMOX1,NFKBIA,LCAT,CHUK,IKBKB,PLCG2,PLCG1,JAK2,MAP2K4,NFKB1,RELA,MAPK9,MAPK8,MAPK14	PLA2G6,PLA2G10,PDIA3,CSF2,PLD3
CDK5 Signaling	7.76E-07	BDNF	PPP2R1A,ADCY6,PPP1CC,PPP1CA,PPP2CA,EGR1,NTRK2,MAPK14,MAPK3,PRKACA,PPP2R4,MAPT,ABL1,MAPK8,MAPK9,RAF1,PPP1R14A,CDK5	PRKAG1,PPP1R11,PRKAR1B,ADCY10,GNAS

GÎ±i Signaling	7.94E-07	RGS4,DRD4,DRD2,DRD3,GRM3	SRC,ADCY6,GNB1,STAT3,GNB5,RAF1,S1PR1,GNG10,PTGER3,GNB2L1,MAPK3,GRB2,PRKACA,GABBR1,GNAI2,GNAI3,GNAI1	PRKAG1,RGS7,PRKAR1B,ADCY10,GNAS
IL-22 Signaling	8.32E-07	AKT1,AKT3	STAT3,STAT1,JAK1,MAPK14,MAPK3,AKT2,MAP2K4,MAPK8,MAPK9	
Estrogen-mediated S-phase Entry	8.32E-07	NONE	CCNA2,ESR2,ESR1,CCND1,CDKN1B,CDKN1A,CCNE1,CDK1,SKP2,CDC25A	E2F6
Docosahexaenoic Acid (DHA) Signaling	1.00E-06	AKT1,AKT3	BCL2L1,CASP3,APP,GSK3A,AKT2,FOXO1,CASP9,BAD,PIK3R1,PDPK1,GSK3B,BCL2	
IL-10 Signaling	1.07E-06	TNF	IKBKB,STAT3,IL4R,TRAF6,FOS,HMOX1,NFKBIA,JUN,MAP3K7,CHUK,MAPK14,SP1,MAP2K4,NFKB1,RELA,MAPK8,JAK1	IL10RA
Tight Junction Signaling	1.20E-06	AKT1,AKT3,TNF	PPP2R1A,ACTG1,SPTAN1,CLDN19,EPB41,CTNNB1,AKT2,CASK,FOS,JUN,PPP2R4,NFKB1,RELA,MPDZ,TGFBR1,RAC1,PRKCZ,NSF,F11R,TNFRSF1B,TNFRSF1A,PPP2CA,PTEN,INADL,PRKACA,CDC42	PRKAG1,CLDN18,PRKAR1B,MYL9
Aldosterone Signaling in Epithelial Cells	1.23E-06	PLCH2,PRKD1	PRKCH,PLCG2,HSPB7,RAF1,DNAJB1,HSPA4,NEDD4,HSP90AB1,MAPK3,PIK3R1,PLCB1,HSPB1,PRKCA,PRKCB,PRKCE,PRKCD,PRKCG,PRKCZ,PDPK1,PRKCQ,ITPR3,ITPR1,DNAJC8,DNAJC7,PLCG1,HSP90AA1	PDIA3,DNAJC10,ASIC2
Agrin Interactions at Neuromuscular Junct	1.38E-06	NRG1,PAK6	ERBB2,SRC,ITGB3,ACTG1,ERBB3,RAC1,JUN,EGFR,MAPK3,UTRN,MAP2K4,PAK4,MAPK8,MAPK9,ERBB4,PAK1,CDC42	
IL-17A Signaling in Gastric Cells	1.38E-06	TNF	FOS,EGFR,JUN,MAPK14,MAPK3,MAP2K4,NFKB1,RELA,MAPK9,MAPK8	
IL-17A Signaling in Fibroblasts	1.58E-06	NONE	IKBKB,TRAF6,FOS,NFKBIA,JUN,MAP3K7,GSK3B,MAPK14,MAPK3,CHUK,NFKB1,RELA	CEBPD
GÎ±12/13 Signaling	1.62E-06	AKT1,AKT3	NFKBIA,IKBKB,PTK2B,MAP2K7,MAP2K4,ARHGEF1,RAF1,RASA1,JUN,MAP3K5,MAPK3,PIK3R1,NFKB1,RELA,MAPK9,SRC,CHUK,MAPK8,CTNNB1,VAV2,AKT2,BTK,CDC42	MYL9

Endoplasmic Reticulum Stress Pathway	1.74E-06	NONE	TRAF2,CASP3,MBTPS1,CALR,CASP9,ATF4,MAPK8,MAP3K5,ERN1	MBTPS2
Amyotrophic Lateral Sclerosis Signaling	2.40E-06	CACNA1C,AKT3,GRIN2A	BCL2L1,TP53,CASP3,RAC1,NEFM,NEFL,XIAP,CASP9,GRIN3A,GRIA2,GLUL,GRIN3B,VEGFA,PIK3R1,GRIN1,PAK1,BCL2	SSR4,RAB5C,CASP1
Human Embryonic Stem Cell Pluripotency	2.40E-06	TCF4,BDNF,AKT3,AKT1	TGFBR1,NTF3,TCF3,SMAD4,S1PR1,SMAD7,GSK3A,SMAD1,SMAD2,SMAD3,CTNNB1,GSK3B,FOXO1,NTRK2,PIK3R1,APC,PDPK1,NTRK3,WNT7B,AKT2	GNAS,PDGFB,FZD3,FGFR1
IL-2 Signaling	2.45E-06	AKT1,AKT3	FOS,SYK,JUN,AKT2,MAPK3,PTK2B,GRB2,JAK3,PIK3R1,JAK1,MAPK8,RAF1,CSNK2B,CSNK2A1	
IL-17 Signaling	2.75E-06	AKT1,AKT3	MAPK8,PTGS2,TRAF6,JAK1,JUN,MAP3K7,GSK3B,MAPK14,MAPK3,AKT2,JAK2,PIK3R1,MAP2K4,NFKB1,RELA,MAPK9,MAPKAPK2	
Melanoma Signaling	2.82E-06	AKT1,AKT3	TP53,MAPK3,CCND1,CDKN1A,AKT2,PTEN,BAD,BRAF,PIK3R1,MDM2,RAF1	MITF
Protein Ubiquitination Pathway	2.88E-06	NONE	HSPA4,NEDD4,TCEB1,UBE2D2,UBE2D3,UBE2D1,VHL,XIAP,SKP2,USP48,DNAJB1,CDC34,PARK2,HSP90AB1,PSMA3,PSMA1,FBXW7,BRCA1,HSPB7,TRAF6,HSPB1,IFNG,USP4,UBE2E1,UBE2E3,THOP1,USP15,DNAJC8,MDM2,UBE2E2,UBE2M,RBX1,DNAJC7,UBD,NEDD4L,UBC,HSP90AA1,UBE2S	UBE4B,SUGT1,PSMC3,DNAJC10,PSMB8
FAK Signaling	3.98E-06	AKT1,PAK6,AKT3	SRC,MAPK3,ACTG1,RAC1,PTEN,FYN,EGFR,AKT2,PAK1,PLCG2,PLCG1,GRB2,PIK3R1,PAK4,PDPK1,BCAR1,EGF,RAF1	
Dendritic Cell Maturation	4.57E-06	PLCH2,AKT1,AKT3,TNF	NFKBIA,IKBKB,PLCG2,PLCG1,MAP2K4,STAT1,MAPK3,JAK2,PIK3R1,NFKB1,RELA,MAPK9,PLCB1,TRAF6,CHUK,CREB1,EP300,MAPK8,MAPK14,TNFRSF1B,TNFRSF1A,AKT2,ATF4,HLA-DMB,CREBBP	CD1D,PDIA3,CSF2,FSCN1

G Protein Signaling Mediated by Tubby	5.13E-06	NONE	PLCB1,GNAQ,GNB5,GNG10,GNB1,INS,GNB2L1,PLCG2,PLCG1,JAK2,ABL1	GNAS
Wnt/Ca+ pathway	5.50E-06	PLCH2,NFATC3	PLCB1,PRKCA,CAMK2A,CREB1,GSK3B,PLCG2,PLCG1,NFKB1,ATF4,RELA,EP300,CREBBP	FZD3,PDIA3
Estrogen Receptor Signaling	8.13E-06	NONE	PHB2,ERCC3,KAT2B,PRKDC,RAF1,NCOR2,GTF2E1,SMARCA4,MAPK3,HNRNPD,SRC,HIST2H3C,EP300,RBFOX2,ESR2,TAF7L,ESR1,GRB2,CDK7,CTBP1,HIST3H3,CREBBP	GTF2H2,MED23,POLR2I,SRA1
FXR/RXR Activation	8.13E-06	AKT1,AKT3,APOE,TNF	RARA,VLDLR,ALB,SCARB1,LCAT,AKT2,APOH,INS,FOXO1,PLTP,MAP2K4,CLU,MAPK8,MAPK9,CREBBP	UGT2B4,PCYOX1,APOC4,APOC2,APOC1,APOL1,SULT2A1
PEDF Signaling	9.33E-06	TCF4,BDNF,AKT3,AKT1	BCL2L1,MAPK14,TP53,RAC1,NFKBIA,CHUK,IKBKB,MAPK3,AKT2,PIK3R1,NFKB1,RELA,RAF1,BCL2	
FcÎ³RIIB Signaling in B Lymphocytes	1.17E-05	AKT1	SYK,GRB2,PLCG2,DOK1,PIK3R1,MAP2K4,PDPK1,MAPK8,MAPK9,LYN,BTK	CD79A
Role of JAK family kinases in IL-6-type Cy	1.17E-05	NONE	STAT3,STAT1,JAK1,MAPK14,MAPK3,JAK2,MAP2K4,MAPK8,MAPK9	OSMR
MIF Regulation of Innate Immunity	1.17E-05	NONE	PTGS2,TP53,FOS,NFKBIA,JUN,MAPK3,MAP2K4,NFKB1,RELA,MAPK9,MAPK8	PLA2G6,PLA2G10
SAPK/JNK Signaling	1.45E-05	NFATC3	TRAF2,MAP3K5,TP53,RAC1,JUN,GNB1,MAP3K7,TRADD,IRS1,RIPK1,MAP3K11,GRB2,MAP2K7,PIK3R1,MAP2K4,MAPK8,MAPK9,MAPK8IP1,MAPK8IP2,CDC42	
Unfolded protein response	1.55E-05	NONE	TRAF2,MBTPS1,CALR,HSPA4,PDIA2,MAP3K5,MAP2K7,ERN1,ATF4,MAPK8,CANX,BCL2	MBTPS2,CEBPG,CEBPD
GÎ±s Signaling	1.58E-05	NONE	SRC,ADCY6,ADORA2A,GNB5,GNG10,RYR2,GNB1,CREB1,GNB2L1,MAPK3,PRKACA,BRAF,RGS2,GLP1R,ATF4,EP300,CREBBP	ADCY10,GNAS,PRKAG1,HCAR3,PRKAR1B,HCK
Toll-like Receptor Signaling	1.74E-05	TNF	UBD,MAP2K4,TRAF6,FOS,NFKBIA,JUN,MAP3K7,TNFAIP3,CHUK,IKBKB,ECSIT,UBC,NFKB1,RELA,IRAK1,MAPK8,MAPK14	

Antiproliferative Role of TOB in T Cell Sign	1.78E-05	NONE	TGFBR1,CCNA2,SMAD4,CDKN1B,CCNE1,PABPC4,SMAD2,SMAD3,CDC34,SKP2	
Role of Pattern Recognition Receptors in F	2.09E-05	TNF,PRKD1	PRKCH,TRAF6,PLCG2,IFNG,SYK,PRKCB,PRKCE,PRKCD,PRKCG,MAPK3,CNTF,PRKCZ,PRKCA,CREB1,PIK3R1,MAP2K4,PRKCQ,NFKB1,RELA,MAPK9,MAPK8	CSF2,CASP1
PAK Signaling	2.14E-05	PAK6,TNF	CASP3,NCK2,RAC1,LIMK1,MAPK3,PTK2B,GRB2,NCK1,PIK3R1,MAP2K4,PAK4,MAPK8,MAPK9,RAF1,PAK1,CDC42	PDGFB,MYL9
IL-15 Production	2.63E-05	NONE	STAT1,MST1R,MAP3K11,PTK2B,PRKCZ,JAK2,JAK3,JAK1,NFKB1,RELA	
Notch Signaling	2.69E-05	NOTCH4	MAML2,MAML3,MAML1,PSEN2,RBPJ,NOTCH1,DLL4,ADAM17,PSEN1	APH1B,FURIN
Activation of IRF by Cytosolic Pattern Reco	2.82E-05	TNF	IKBKB,STAT1,TRAF6,NFKBIA,JUN,CHUK,RIPK1,CREBBP,MAP2K4,NFKB1,RELA,MAPK9,MAPK8,PIN1	ZBP1
Nur77 Signaling in T Lymphocytes	3.16E-05	PPP3CC	HDAC1,HDAC2,CASP3,CABIN1,SIN3B,NR4A1,CALM1 (includes others),CASP9,EP300,HLA-DMB,SIN3A,BCL2	CD3E,CD3G
Caveolar-mediated Endocytosis Signaling	3.63E-05	NONE	SRC,ITGB3,ACTG1,ITSN1,ALB,FYN,ABL1,INS,PRKCA,DNM2,COPB1,FLNA,PTPN1,COPB2,EGF,EGFR	RAB5C
Role of BRCA1 in DNA Damage Response	3.72E-05	NONE	BRCA1,SMARCC1,TP53,STAT1,TOPBP1,BARD1,IFNG,CDKN1A,RFC5,SMARCE1,FANCA,SMARCB1,CHEK1,SMARCA4,POU2F1	BLM,E2F6,BRE
TWEAK Signaling	4.47E-05	NONE	IKBKB,TRAF2,CASP3,NFKBIA,CHUK,TRADD,CASP9,RIPK1,XIAP,NFKB1,RELA	
Oncostatin M Signaling	4.47E-05	NONE	EPAS1,MT2A,STAT1,MAPK3,GRB2,JAK2,JAK3,JAK1,STAT3,RAF1	OSMR
Paxillin Signaling	4.57E-05	PAK6	SRC,ITGB3,ACTN2,CDC42,ACTG1,NCK2,RAC1,ACTN1,ARFIP2,MAPK14,PTK2B,GRB2,PIK3R1,MAP2K4,PAK4,MAPK8,MAPK9,BCAR1,PAK1,NCK1	

GADD45 Signaling	5.89E-05	NONE	BRCA1,TP53,CCND1,CCND3,CCNE1,CDK1,CDKN1A	CCND2
CTLA4 Signaling in Cytotoxic T Lymphocy	6.03E-05	AKT1,AKT3	PPP2R1A,PPP2CA,AP1M1,FYN,AKT2,PPP2R4,SYK,PLCG1,GRB2,JAK2,PIK3R1,AP2A2,AP2B1	LCP2,CD3G,ZAP70,CD3E
Mechanisms of Viral Exit from Host Cells	6.31E-05	PRKD1	PRKCH,ACTG1,PRKCA,PRKCE,VPS28,PRKCB,NEDD4,PRKCD,PRKCG,PRKCZ,PRKCQ	
Role of NANOG in Mammalian Embryonic	6.61E-05	AKT1,AKT3	TCL1A,STAT3,SMAD9,SMAD4,SMAD1,GSK3B,CTNNB1,MAPK3,BMPR1B,GRB2,JAK2,JAK3,PIK3R1,JAK1,APC,AKT2,WNT7B,RAF1,TP53	FZD3
Cdc42 Signaling	6.61E-05	NONE	LIMK1,MAPK14,MAP2K4,RAF1,JUN,MAPK8,MAPK9,SRC,FOS,PRKCZ,APC,RASA1,EXOC1,EXOC7,EXOC4,ARPC1B,VAV2,GSK3B,MAP3K11,PAK4,BAIAP2,HLA-DMB,PAK1,CDC42	ARPC3,CD3G,CDC42SE1,CD3E,MYL9
Cellular Effects of Sildenafil (Viagra)	8.91E-05	PLCH2,CACNA1C,KCNN3	PLCB1,ADCY6,NOS3,ACTG1,CALM1 (includes others),PABPC4,PDE3B,PLCG2,PLCG1,PRKACA,KCNN2,ITPR3,PDE4B,ITPR1,CACNG2	ADCY10,GNAS,PRKAG1,PDIA3,PRKAR1B,MYL9
LXR/RXR Activation	8.91E-05	APOE,TNF	PTGS2,TNFRSF1B,TNFRSF1A,ALB,LCAT,APOH,LDLR,PLTP,ABCA1,NFKB1,RELA,CLU,NCOR2	ACACA,PCYOX1,APOC1,APOC4,APOC2,CYP51A1,APOA5,APOL1
DNA Methylation and Transcriptional Repr	9.12E-05	NONE	HDAC1,HDAC2,DNMT1,RBBP7,RBBP4,MBD3,MTA2,SIN3A	
Role of JAK1 and JAK3 in Î³c Cytokine Sig	0.00011	FES	STAT3,IL4R,STAT1,SYK,IRS1,IRS2,MAPK3,PTK2B,GRB2,JAK2,JAK3,PIK3R1,JAK1,SH2B2	
Parkinson's Signaling	0.000129	NONE	CASP3,CASP9,MAPK14,SNCA,MAPK8,PARK2	5-Sep
Role of IL-17F in Allergic Inflammatory Air	0.000132	NONE	RPS6KA3,TRAF6,CREB1,MAPK3,NFKB1,ATF4,RELA,EP300,RAF1,CREBBP	CSF2,CCL4
CNTF Signaling	0.000186	AKT1	CNTF,RPS6KA3,STAT3,STAT1,RPS6KB1,MAPK3,GRB2,JAK2,PIK3R1,JAK1,MTOR,RAF1	
Thyroid Cancer Signaling	0.000234	TCF4,BDNF	NTF3,TP53,TCF3,CCND1,NTRK2,NTRK3,CTNNB1,MAPK3,BRAF	
Neuroprotective Role of THOP1 in Alzhein	0.000234	NONE	ACE,IFNG,APP,CREB1,THOP1,PRKACA,MAPT,YWHAE	PRKAG1,NFYA,PRKAR1B
IL-9 Signaling	0.000245	TNF	STAT3,STAT1,IRS1,IRS2,JAK3,PIK3R1,JAK1,NFKB1,RELA	

IL-4 Signaling	0.000263	AKT1,AKT3,NFATC3	IL4R,INPP5K,RPS6KB1,IRS1,AKT2,GRB2,JAK2,JAK3,PIK3R1,JAK1,MTOR,HLA-DMB	INPPL1
Remodeling of Epithelial Adherens Junctio	0.000275	NONE	ACTN2,ACTN1,ACTG1,SRC,TUBA1B,TUBB,HGS,CTNNB1,DNM2,APC,TUBB2A,ARPC1B	ARPC3,TUBA1C,RAB5C
RhoA Signaling	0.000275	NONE	ARHGEF12,ACTG1,KTN1,ARPC1B,PLXNA1,LIMK1,NEDD4,IGF1R,RDX,PTK2B,ARHGAP12,TTN,CIT,BAIAP2,ARHGEF1,CDC42EP4,DLC1	SEPT5,ARPC3,ARHGAP1,SEPT3,MYL9
Actin Cytoskeleton Signaling	0.000302	PAK6	ACTG1,LIMK1,TRIO,BRK1,RAF1,EGF,BAIAP2,ARHGEF1,MAPK3,TIAM2,TTN,PIK3R1,FGF12,ARHGEF12,RAC1,RDX,FLNA,BCAR1,ACTN2,ACTN1,ARPC1B,VAV2,INS,GRB2,PAK4,APC,PAK1,CDC42	ARPC3,ABI2,PDGFB,MYL9
Role of JAK1, JAK2 and TYK2 in Interfero	0.000398	NONE	STAT3,STAT1,IFNG,JAK2,JAK1,NFKB1,RELA,RAF1	
DNA damage-induced 14-3-3Ïƒ Signaling	0.000457	AKT1,AKT3	BRCA1,CDK1,AKT2,CCNE1,TP53	
Role of PI3K/AKT Signaling in the Pathoge	0.000479	AKT1,AKT3	IFNG,NFKBIA,CASP9,MAPK3,AKT2,PIK3R1,NFKB1,RELA,GSK3B,GNAI2,GNAI3,GNAI1	
Glutamate Receptor Signaling	0.00049	GRIN2A,GRM3	DLG4,GRIA2,CALM1 (includes others),GNB1,PICK1,GRIN3A,GLUL,GRIN3B,GRIP1,HOMER3,GRIN1	
Regulation of Cellular Mechanics by Calpa	0.00049	NONE	SRC,ACTN1,CCNA2,ACTN2,CCND1,CDKN1B,CCNE1,EGFR,MAPK3,GRB2,CDK6,EGF,CDK1	
Telomere Extension by Telomerase	0.000724	NONE	TNKS,POT1,TERF1,TERF2IP,XRCC5,XRCC6	
GABA Receptor Signaling	0.000776	KCNN3	ADCY6,UBC,UBQLN1,NSF,GABARAP,UBD,KCNN2,AP2A2,GABBR1,GABRA1,AP2B1	ADCY10,GNAS
PXR/RXR Activation	0.000776	AKT1,AKT3,TNF	CYP2C8,AKT2,INS,FOXO3,FOXO1,PRKACA,RELA	PRKAG1,ALDH3A2,PRKAR1B,SULT2A1
TREM1 Signaling	0.000832	AKT1,AKT3,TNF	STAT3,MAPK3,AKT2,PLCG2,PLCG1,GRB2,JAK2,NFKB1,RELA,IRAK1	CSF2,CASP1
phagosome formation	0.00107	PLCH2,PRKD1	PLCB1,PRKCH,SYK,PRKCB,PRKCE,PRKCD,PRKCG,PLCG2,PLCG1,PRKCZ,PRKCA,PIK3R1,PRKCQ,RHOJ	PDIA3,DIRAS3,INPPL1

Regulation of eIF4 and p70S6K Signaling	0.00145	AKT1,AKT3	EIF3H,PPP2R1A,RPS20,IRS1,PPP2CA,GRB2,RPS6KB1,EIF1AY,PPP2R4,EIF3G,MAPK14,MAPK3,AKT2,PRKCZ,MTOR,EIF3F,PIK3R1,PDPK1,EIF4EBP1,RAF1,AGO3	
Bladder Cancer Signaling	0.00145	NONE	ERBB2,MMP17,TP53,SUV39H1,CDKN1A,EGFR,MAPK3,VEGFA,FGF12,ABL1,CCND1,MDM2,RAF1,EGF,SIN3A	MMP23B
LPS/IL-1 Mediated Inhibition of RXR Funct	0.00151	APOE,TNF	RARA,CYP2C8,TRAF2,MAPK9,TNFRSF1B,TRAF6,TNFRSF1A,SCARB1,MAP2K4,JUN,MAP3K7,PPARGC1B,MAP2K7,ECSIT,ABCA1,PLTP,MAPK8,IRAK1	CHST12,HS3ST1,ALDH3A2,ALDH6A1,APOC4,APOC2,APOC1,ALDH1B1,SLC27A4,SLC27A3,SULT2A1
Interferon Signaling	0.00178	NONE	STAT1,IFNG,PIAS1,JAK2,JAK1,RELA,IFIT3,BCL2	PSMB8
Role of RIG1-like Receptors in Antiviral Inn	0.00219	NONE	IKBKB,TRAF2,TRAF6,NFKBIA,CHUK,RIPK1,NFKB1,RELA,EP300,CREBBP	
TR/RXR Activation	0.00302	AKT1,AKT3,NRGN	NCOA4,SCARB1,LDLR,PDE3B,AKT2,MTOR,PIK3R1,MDM2,EP300,NCOR2	APOA5,ACACA
Netrin Signaling	0.00324	NFATC3,PPP3CC	RYR2,PRKACA,NCK1,NCK2,RAC1	PRKAG1,PRKAR1B
Role of IL-17A in Arthritis	0.00339	NONE	MAPK8,PTGS2,NFKBIA,MAPK14,MAPK3,PIK3R1,MAP2K4,NFKB1,RELA,MAPK9,MAPKAPK2	
DNA Double-Strand Break Repair by Non-	0.00363	NONE	LIG4,PRKDC,XRCC5,PARP1,XRCC6	
Lipid Antigen Presentation by CD1	0.00363	NONE	CALR,AP2B1,CANX,AP2A2	CD1D,PDIA3,CD3E
Prostanoid Biosynthesis	0.0038	PTGIS	PTGES3,PTGS2,PTGS1	
Circadian Rhythm Signaling	0.00389	GRIN2A	CREB1,GRIN3A,GRIN3B,ATF4,GRIN1,EP300,CREBBP	
Role of CHK Proteins in Cell Cycle Checkp	0.00398	NONE	BRCA1,PPP2R1A,TP53,PPP2CA,CDKN1A,PPP2R4,CDK1,CDC25A,RFC5,CHEK1	E2F6
PCP pathway	0.00407	NONE	EFNB1,HSPB1,RAC1,SDC2,JUNB,JUN,MAP2K4,MAPK8,MAPK9,WNT7B	VANGL1,FZD3
Role of JAK2 in Hormone-like Cytokine Sig	0.00575	NONE	STAT3,STAT1,IRS1,IRS2,JAK2,JAK1,SH2B2,PTPN1	
Mitotic Roles of Polo-Like Kinase	0.00603	NONE	PPP2R1A,RAD21,PPP2CA,SMC3,CDC27,PPP2R4,HSP90AB1,STAG2,HSP90AA1,CDC25A,CDK1	PTTG1

Phospholipases	0.00603	PLCH2	PLCB1,LCAT,HMOX1,PLCG1,PLCG2	PLA2G6,PDIA3,PLA2G10,LIPG,PLD3
Hepatic Fibrosis / Hepatic Stellate Cell Act	0.00646	TNF	TGFBR1,TNFRSF1B,IL4R,STAT1,TNFRSF1A,IFNG,SMAD7,EGFR,IGF1R,SMAD2,CSF1,SMAD4,VEGFA,COL4A1,NFKB1,RELA,SMAD3,EGF,A2M,BCL2	PDGFB,FGFR1,IL10RA,MYL9
Spliceosomal Cycle	0.00646	NONE	U2AF1/U2AF1L5,U2AF2	
Polyamine Regulation in Colon Cancer	0.00661	TCF4	CTNNB1,MAX,PSME3,MXD1,APC	
Systemic Lupus Erythematosus Signaling	0.00794	AKT1,AKT3,NFATC3,TNF	SNRNP70,LSM8,PLCG2,SF3B4,FOS,JUN,SNRNP35,PRPF40A,GRB2,AKT2,MAPK3,PLCG1,SNRNP27,MTOR,PIK3R1,PRPF38A,SNRPB,LYN,HNRNPC	PRPF8,CD3E,CD3G,CREM,CD79A
Role of p14/p19ARF in Tumor Suppressio	0.00851	NONE	TP53,RAC1,UBTF,TTF1,PIK3R1,MDM2,NPM2	

GWAS Interactome Pathways

Pathway Name	B-H P-value value of association	GWAS genes	Known Interactors	Novel Interactors
Molecular Mechanisms of Cancer	1.00E-22	AKT3,PAK6,TCF4,PRKD1	CAMK2B,CDC42,JAK3,RABIF,FOS,PRKCB,PRKCH,MAPK9,PRKCE,SRC,JAK2,CDKN2C,AURKA,CTNNB1,SMAD2,CDC25A,JAK1,CDK5,RHOJ,ABL1,PRKCD,HAT1,CCND1,SMAD1,GSK3B,JUN,MAPK14,AKT1,ARHGEF12,IRS1,PIK3R1,MAX,TP53,APC,FYN,SUV39H1,CCNE1,CDKN1A,SIN3A,CREBBP,MAPK8,CDK6,PRKCZ,MAPK3,PSEN1,MAP3K7,BRCA1,NFKB1,TCF3,CDK7,WNT7B,EP300,CAMK2A,SMAD9,RELA,PRKCA,ARHGEF1,BCL2,RASA1,MDM2,PRKACA,PAK4,PRKCG	PRKAR1B,LRP1,APH1B,CCND2,CASP3,PRKAG1,GSK3A,FZD3,ZBTB17,GNAS
Glioma Signaling	3.16E-15	AKT3,PRKD1	CAMK2B,AKT1,CALM1 (includes others),PIK3R1,TP53,SUV39H1,CDKN1A,SIN3A,PRKCB,PRKCH,CDK6,PRKCZ,MAPK3,PRKCE,EGFR,MTOR,CDKN2C,CAMK1D,CAMK2A,PLCG2,ABL1,IGF1R,PRKCD,PRKCA,CCND1,PLCG1,MDM2,PRKCG	PTEN,PDGFB
RAR Activation	5.01E-15	AKT3,PRKD1	JUN,MAPK14,AKT1,SMARCA4,PIK3R1,FOS,PRKCB,CREBBP,MAPK8,PRKCH,PRKCZ,MAPK9,ERCC3,PRKCE,NFKB1,SRC,JAK2,CDK7,CSNK2B,SMAD2,CSF2RB,EP300,NR2F2,RELA,SMAD9,PARP1,PRKCD,PDPK1,PRKCA,SMAD1,NR2F1,PRKACA,PRKCG	PTEN,CRABP1,GTF2H2,PRKAR1B,RDH5,SRA1,GNAS,PRKAG1
Role of NFAT in Cardiac Hypertrophy	7.94E-15	AKT3,PLCH2,PRKD1	GSK3B,CAMK2B,CSNK1A1,MAP2K7,MAPK14,AKT1,CALM1 (includes others),GNG10,GNB1,PIK3R1,PRKCB,MAPK8,PRKCH,HDAC4,PRKCZ,HDAC1,MAPK9,MAPK3,PRKCE,MAP3K7,HDAC5,HDAC2,SRC,GNB2L1,EP300,CAMK1D,CAMK2A,HDAC7,PLCG2,IGF1R,PRKCD,PRKCA,GNB5,PLCG1,PRKACA,PRKCG	PRKAR1B,GNAS,PDIA3,PRKAG1
UVB-Induced MAPK Signaling	6.31E-14	PRKD1	JUN,PRKCE,MAPK14,EGFR,MTOR,AKT1,HIST2H3C,PIK3R1,TP53,FOS,HIST3H3,PRKCD,PRKCA,PRKCB,MAPK8,PRKCH,PRKCZ,EIF4EBP1,MAPK9,PRKCG,MAPK3	RPS6KA3
Androgen Signaling	1.58E-13	PRKD1	JUN,CALM1 (includes others),GNG10,GNB1,PRKCB,CREBBP,GTF2E1,PRKCH,PRKCZ,MAPK3,ERCC3,PRKCE,NFKB1,SRC,CDK7,GNB2L1,EP300,RELA,AR,HSPA4,PRKCD,PRKCA,GNB5,CCND1,PRKACA,PRKCG	GTF2H2,PRKAR1B,POLR2I,GNAS,PRKAG1
Huntington's Disease Signaling	1.58E-13	AKT3,PRKD1	JUN,NEUROD1,MAP2K7,AKT1,GNG10,UBD,GNB1,PIK3R1,TP53,UBE2S,SIN3A,PRKCB,CREBBP,MAPK8,PRKCH,HDAC4,PRKCZ,HDAC1,MAPK9,UBC,MAPK3,PRKCE,HDAC5,EGFR,HDAC2,MTOR,GNB2L1,EP300,CDK5,SP1,ZDHHC17,HDAC7,IGF1R,HSPA4,PRKCD,PDPK1,DLG4,PRKCA,GNB5,DNM2,AP2A2,RASA1,PRKCG	POLR2I,CASP3

PI3K/AKT Signaling	3.98E-13	AKT3	GSK3B,YWHAQ,AKT1,JAK3,PIK3R1,TP53,MAPK8IP1,TSC2,CDKN1A,PRKCZ,EIF4EBP1,YWHAB,PPP2CA,MAPK3,NFKB1,MTOR,CHUK,JAK2,YWHAG,CTNNB1,JAK1,RELA,PDPK1,CCND1,BCL2,PTGS2,MDM2,YWHAZ	PTEN,GSK3A,INPP5K,PPP2R1A
G Beta Gamma Signaling	3.98E-13	AKT3,PRKD1	PRKCE,CDC42,EGFR,SRC,AKT1,GNB2L1,GNG10,BTK,GNB1,PLCG2,PRKCD,PDPK1,PRKCA,GNB5,DNM2,PRKCB,PLCG1,PRKCH,PRKACA,PRKCZ,PRKCG,MAPK3	PRKAR1B,GNAS,KCNJ3,PRKAG1
Neuropathic Pain Signaling In Dorsal Horn Neurons	1.58E-12	GRIN2A,PLCH2,PRKD1,GRM3	CAMK2B,PIK3R1,GRIN1,FOS,GRIN3B,PRKCB,PRKCH,PRKCZ,GRIN3A,MAPK3,PRKCE,SRC,KCNN2,CAMK1D,CAMK2A,PLCG2,PRKCD,PRKCA,PLCG1,PRKACA,PRKCG	GRIA2,PRKAR1B,PDIA3,PRKAG1
GNRH Signaling	1.58E-12	PAK6,PRKD1	JUN,CAMK2B,MAP2K7,MAPK14,CDC42,FOS,PRKCB,CREBBP,MAPK8,PRKCH,PRKCZ,MAPK9,MAPK3,PRKCE,MAP3K7,NFKB1,EGFR,SRC,EP300,EGR1,CAMK2A,RELA,PRKCD,PRKCA,DNM2,PAK4,PRKACA,PRKCG	PRKAR1B,GNAS,PRKAG1
ERK/MAPK Signaling	2.00E-12	VRK2,PAK6	PPP1CC,YWHAQ,PPP1R14A,PIK3R1,FOS,STAT3,FYN,HSPB7,PRKCB,CREBBP,EIF4EBP1,YWHAB,ESR1,PPP2CA,MAPK3,PRKCE,SRC,MAPKAPK5,YWHAG,EP300,HIST2H3C,PLCG2,HSPB1,HIST3H3,PRKCD,PRKCA,PTK2B,BCAR1,PLCG1,PAK4,PRKACA,YWHAZ,PRKCG	PLA2G6,PPP2R1A,PRKAR1B,PLA2G10,PRKAG1
14-3-3-mediated Signaling	2.51E-12	AKT3,PLCH2,PRKD1,SRPK2	JUN,GSK3B,YWHAQ,AKT1,PIK3R1,FOS,TSC2,PRKCB,MAPK8,PRKCH,PRKCZ,YWHAB,MAPK9,MAPK3,PRKCE,SRC,YWHAG,PLCG2,PRKCD,PRKCA,TUBA1B,PLCG1,YWHAZ,PRKCG	GSK3A,TUBA1C,PDIA3
Glucocorticoid Receptor Signaling	2.51E-12	NFATC3,AKT3	JUN,MAP2K7,MAPK14,AKT1,PRKAA1,JAK3,SMARCA4,PIK3R1,TAF7L,SUMO1,FKBP4,FOS,STAT3,FKBP5,TSC22D3,CDKN1A,CREBBP,MAPK8,GTF2E1,ESR1,MAPK9,MAPK3,TRAF6,ERCC3,MAP3K7,NFKB1,CHUK,JAK2,CDK7,SMAD2,EP300,JAK1,RELA,AR,HSPA4,PTGES3,BCL2,PTGS2,PRKAA2,PRKACA,PRKAB2	CSF2,GTF2H2,POLR2I,SRA1,PRKAG1
p70S6K Signaling	3.98E-12	AKT3,PLCH2,PRKD1	YWHAQ,AKT1,IRS1,PIK3R1,SYK,PRKCB,PRKCH,PRKCZ,YWHAB,PPP2CA,MAPK3,PRKCE,EGFR,MTOR,SRC,YWHAG,BTK,JAK1,PLCG2,PRKCD,PDPK1,PRKCA,IL4R,PLCG1,YWHAZ,PRKCG	PPP2R1A,PDIA3
FcÎ³ Receptor-mediated Phagocytosis in Macrophages and Monocytes	7.94E-12	AKT3,PRKD1	PRKCE,CDC42,SRC,AKT1,PIK3R1,ARPC1B,VAV2,PRKCD,SYK,FYN,PRKCA,PTK2B,NCK2,PRKCB,PLCG1,PRKCH,PRKCZ,PRKCG,MAPK3	PTEN,CSF2,PLD3,PLA2G6,LCP2,ARPC3
ErbB Signaling	7.94E-12	PAK6,PRKD1	JUN,GSK3B,MAPK14,CDC42,AKT1,PIK3R1,FOS,PRKCB,MAPK8,PRKCH,PRKCZ,MAPK9,MAPK3,PRKCE,EGFR,MTOR,PLCG2,PRKCD,PDPK1,PRKCA,NCK2,PLCG1,PAK4,PRKCG	
IL-8 Signaling	1.58E-11	AKT3,PRKD1	JUN,AKT1,GNG10,GNB1,PIK3R1,LASP1,FOS,PRKCB,MAPK8,PRKCH,PRKCZ,EIF4EBP1,MAPK9,MAPK3,TRAF6,PRKCE,NFKB1,EGFR,MTOR,CHUK,SRC,GNB2L1,RHOJ,RELA,PRKCD,PRKCA,PTK2B,GNB5,CCND1,BCL2,PTGS2,PRKCG	PLD3,CCND2,MYL9,GNAS

CCR5 Signaling in Macrophages	1.58E-11	PRKD1	JUN,PRKCE,MAPK14,CALM1 (includes others),GNB2L1,GNG10,GNB1,PLCG2,FOS,PRKCD,PRKCA,PTK2B,GNB5,PRKCB,PLCG1,MAPK8,PRKCH,PRKCZ,MAPK9,PRKCG	GNAS,CCL4
Synaptic Long Term Potentiation	2.00E-11	GRIN2A,CACNA1C,PLCH2,PRKD1,GRM3	CAMK2B,PPP1CC,PPP1R14A,CALM1 (includes others),GRIN1,GRIN3B,PRKCB,CREBBP,PRKCH,PRKCZ,GRIN3A,MAPK3,PRKCE,EP300,CAMK2A,PLCG2,PRKCD,PRKCA,PLCG1,PRKACA,PRKCG	GRIA2,PRKAR1B,PDIA3,PRKAG1
P2Y Purigenic Receptor Signaling Pathway	2.00E-11	AKT3,PLCH2,PRKD1	JUN,AKT1,GNG10,GNB1,PIK3R1,FOS,PRKCB,CREBBP,PRKCH,PRKCZ,MAPK3,PRKCE,NFKB1,GNB2L1,EP300,RELA,PLCG2,PRKCD,PRKCA,GNB5,PLCG1,PRKACA,PRKCG	PRKAR1B,GNAS,PDIA3,PRKAG1
Protein Kinase A Signaling	3.16E-11	NFATC3,TCF4,PLCH2,PRKD1	CAMK2B,AKAP13,GNB1,PRKCB,PRKCH,PRKCE,YWHAG,FLNA,CTNNB1,CDC25A,HIST2H3C,HIST3H3,PRKCD,GNB5,PTK2B,YWHAZ,GSK3B,PPP1CC,YWHAQ,TDP2,PPP1R14A,CALM1 (includes others),GNG10,PTPN4,PDE7B,CREBBP,PRKCZ,YWHAB,MAPK3,NFKB1,TCF3,CHUK,GNB2L1,EP300,CAMK2A,PLCG2,RELA,PLN,PRKCA,PTGS2,PLCG1,PRKACA,APEX1,TTN,RYR2,PRKCG	PTEN,PRKAR1B,MYL9,PDIA3,PRKAG1,GSK3A,CREM,GNAS
Prolactin Signaling	5.01E-11	PRKD1	JUN,PRKCE,JAK2,EP300,IRS1,PIK3R1,SP1,PLCG2,FOS,STAT3,PRKCD,FYN,PDPK1,PRKCA,PRKCB,CREBBP,PLCG1,PRKCH,PRKCZ,PRKCG,MAPK3	NMI
Renin-Angiotensin Signaling	5.01E-11	PAK6,PRKD1	JUN,MAPK14,PIK3R1,FOS,STAT3,PRKCB,MAPK8,PRKCH,PRKCZ,MAPK9,MAPK3,PRKCE,NFKB1,JAK2,RELA,PLCG2,PRKCD,PRKCA,PTK2B,PLCG1,PAK4,PRKACA,PRKCG	PRKAR1B,GNAS,PRKAG1
Fc Epsilon RI Signaling	5.01E-11	AKT3,PRKD1	PRKCE,MAP2K7,MAPK14,AKT1,BTK,PIK3R1,VAV2,PLCG2,PRKCD,SYK,FYN,PDPK1,PRKCA,PRKCB,PLCG1,MAPK8,PRKCH,PRKCZ,MAPK9,PRKCG,MAPK3	CSF2,PLA2G6,LCP2,INPP5K,PLA2G10
Role of Macrophages Fibroblasts and Endothelial Cells in Rheumatoid Arthritis	5.01E-11	NFATC3,AKT3,TCF4,PLCH2,PRKD1	JUN,GSK3B,CAMK2B,CSNK1A1,MAP2K7,MAPK14,AKT1,CALM1 (includes others),PIK3R1,IL16,APC,FOS,STAT3,PRKCB,CREBBP,PRKCH,PRKCZ,MAPK9,MAPK3,TRAF6,PRKCE,MAP3K7,NFKB1,CHUK,SRC,TCF3,JAK2,CTNNB1,WNT7B,EP300,CAMK2A,RELA,PLCG2,PRKCD,PRKCA,CCND1,PLCG1,PRKCG	CSF2,LRP1,CEBPG,PDIA3,PDGFB,FZD3
Prostate Cancer Signaling	7.94E-11	AKT3	GSK3B,NFKB1,MTOR,CHUK,AKT1,CTNNB1,EP300,PIK3R1,TP53,RELA,ABL1,AR,PDPK1,SUV39H1,CCND1,BCL2,CCNE1,CDKN1A,SIN3A,CREBBP,MDM2,MAPK3	PTEN
Axonal Guidance Signaling	1.00E-10	NFATC3,AKT3,PAK6,PLCH2,PRKD1,FES	GSK3B,CDC42,AKT1,GNG10,ARHGEF12,GNB1,PIK3R1,DPYSL5,RGS3,ARPC1B,FYN,COPS5,PRKCB,SDCBP,PRKCH,PRKCZ,MAPK3,PRKCE,EFNB1,EPHB6,GNB2L1,WNT7B,SEMA3B,CDK5,PLCG2,ABL1,BAIAP2,PLXNA1,PRKCD,PRKCA,GNB5,NCK2,TUBA1B,BCAR1,PLCG1,RASA1,PAK4,PRKACA,PRKCG	ADAMTS8,TUBA1C,RND1,PRKAR1B,ARPC3,MYL9,NRP1,PDIA3,PRKAG1,PDGFB,EPHA2,SEMA7A,FZD3,ADAM23,GNAS,ERAP2

HER-2 Signaling in Breast Cancer	1.00E-10	AKT3,PRKD1	PRKCE,GSK3B,CDC42,EGFR,AKT1,PIK3R1,TP53,PRKCD,PRKCA,CCND1,CCNE1,TSC2,CDKN1A,PRKCB,PLCG1,MDM2,PRKCH,CDK6,PRKCZ,PRKCG	GSK3A
Dopamine-DARPP32 Feedback in cAMP Signaling	1.00E-10	GRIN2A,CACNA1C,PLCH2,PRKD1,ATP2A2	CSNK1A1,PPP1CC,PPP1R14A,CALM1 (includes others),GRIN1,GRIN3B,PRKCB,CREBBP,PRKCH,PRKCZ,GRIN3A,PPP2CA,PRKCE,EP300,CDK5,PLCG2,PRKCD,PRKCA,PLCG1,PRKACA,PRKCG	CREM,PPP2R1A,PRKAR1B,KCNJ4,GNAS,KCNJ3,PDIA3,PRKAG1
IGF-1 Signaling	1.00E-10	AKT3	GRB10,JUN,YWHAQ,IRS2,YWHAG,JAK2,AKT1,CSNK2B,JAK1,IRS1,PIK3R1,FOS,IGF1R,NEDD4,STAT3,PDPK1,MAPK8,RASA1,PRKACA,YWHAZ,PRKCZ,YWHAB,MAPK3	PRKAR1B,PRKAG1
Production of Nitric Oxide and Reactive Oxygen Species in Macrophages	1.07E-10	AKT3,PRKD1	JUN,PPP1CC,MAP2K7,PPP1R14A,MAPK14,AKT1,JAK3,PIK3R1,FOS,PRKCB,CREBBP,MAPK8,PRKCH,PRKCZ,MAPK9,PPP2CA,MAPK3,PRKCE,MAP3K7,NFKB1,CHUK,JAK2,JAK1,RHOJ,RELA,PLCG2,PRKCD,PRKCA,PLCG1,PRKCG	PPP2R1A,APOL1,PCYOX1,HOXA10
Phospholipase C Signaling	1.66E-10	NFATC3,PRKD1	PPP1R14A,CALM1 (includes others),GNG10,ARHGEF12,GNB1,SYK,FYN,PRKCB,CREBBP,PRKCH,HDAC4,PRKCZ,HDAC1,MAPK3,PRKCE,HDAC5,NFKB1,HDAC2,SRC,GNB2L1,EP300,BTK,RHOJ,HDAC7,RELA,PLCG2,PRKCD,PRKCA,GNB5,ARHGEF1,PLCG1,PRKCG	PLD3,PLA2G6,LCP2,ZAP70,PLA2G10,RPS6KA3,MYL9,GNAS
Tec Kinase Signaling	2.19E-10	PAK6,PRKD1	GNG10,JAK3,GNB1,PIK3R1,FOS,STAT3,FYN,PRKCB,MAPK8,PRKCH,PRKCZ,MAPK9,PRKCE,NFKB1,SRC,JAK2,GNB2L1,BTK,JAK1,RHOJ,RELA,VAV2,PLCG2,PRKCD,PRKCA,PTK2B,GNB5,PLCG1,PAK4,PRKCG	GNAS
Type II Diabetes Mellitus Signaling	2.29E-10	AKT3,PRKD1	MAP2K7,AKT1,PRKAA1,IRS1,PIK3R1,PRKCB,MAPK8,PRKCH,PRKCZ,MAPK9,MAPK3,PRKCE,MAP3K7,NFKB1,MTOR,IRS2,CHUK,INS,RELA,PRKCD,PDPK1,PRKCA,PRKAA2,PRKAB2,PRKCG	PRKAG1
UVC-Induced MAPK Signaling	2.29E-10	PRKD1	JUN,PRKCE,MAPK14,EGFR,SRC,TP53,FOS,PRKCD,PRKCA,PRKCB,MAPK8,PRKCH,PRKCZ,MAPK9,PRKCG,MAPK3	
B Cell Receptor Signaling	2.29E-10	NFATC3,AKT3	JUN,GSK3B,CAMK2B,MAP2K7,MAPK14,CDC42,AKT1,CALM1 (includes others),PIK3R1,SYK,PRKCB,CREBBP,MAPK8,MAPK9,MAPK3,MAP3K7,NFKB1,MTOR,CHUK,TCF3,EP300,BTK,EGR1,CAMK2A,RELA,VAV2,PLCG2,ABL1,PDPK1,PTK2B	PTEN,GSK3A,INPP5K
Erythropoietin Signaling	2.95E-10	AKT3,PRKD1	JUN,PRKCE,NFKB1,SRC,JAK2,AKT1,PIK3R1,RELA,PLCG2,FOS,PRKCD,PDPK1,PRKCA,PRKCB,PLCG1,PRKCH,PRKCZ,PRKCG,MAPK3	
PPARÎ±/RXRÎ± Activation	3.39E-10	PLCH2	JUN,MAP2K7,MAPK14,PRKAA1,IRS1,PRKCB,CREBBP,MAPK8,MAPK3,TRAF6,MAP3K7,NFKB1,CHUK,JAK2,SMAD2,ABCA1,EP300,INS,RELA,PLCG2,PRKCA,AP2A2,PRKAA2,NR2F1,PLCG1,CAND1,PRKACA,PRKAB2	PRKAR1B,ACVR1B,GNAS,PDIA3,MED23,PRKAG1

PI3K Signaling in B Lymphocytes	3.80E-10	NFATC3,AKT3,PLCH2	JUN,CAMK2B,AKT1,CALM1 (includes others),IRS1,PIK3R1,FOS,SYK,FYN,PRKCB,PRKCZ,MAPK3,NFKB1,IRS2,CHUK,BTK,CAMK2A,RELA,VAV2,PLCG2,ABL1,PDPK1,IL4R,PLCG1	PTEN,PDIA3
CREB Signaling in Neurons	3.80E-10	AKT3,GRIN2A,PLCH2,PRKD1,GRM3	CAMK2B,AKT1,CALM1 (includes others),GNG10,GNB1,PIK3R1,GRIN1,PRKCB,CREBBP,PRKCH,PRKCZ,MAPK3,PRKCE,GNB2L1,EP300,CAMK2A,PLCG2,PRKCD,PRKCA,GNB5,PLCG1,PRKACA,PRKCG	GRIA2,PRKAR1B,POLR2I,GNAS,PDIA3,PRKAG1
GÎ±q Signaling	5.62E-10	NFATC3,AKT3,PRKD1	GSK3B,AKT1,CALM1 (includes others),GNG10,GNB1,PIK3R1,PRKCB,PRKCH,PRKCZ,MAPK3,PRKCE,NFKB1,CHUK,GNB2L1,BTK,RHOJ,RELA,PLCG2,PRKCD,PRKCA,PTK2B,GNB5,PLCG1,PRKCG	PLD3,HRH1,RGS7,GNAS
Pancreatic Adenocarcinoma Signaling	5.62E-10	AKT3	CDC42,AKT1,JAK3,PIK3R1,TP53,STAT3,SUV39H1,CCNE1,CDKN1A,SIN3A,MAPK8,MAPK9,MAPK3,NFKB1,EGFR,JAK2,SMAD2,JAK1,RELA,ABL1,CCND1,BCL2,PTGS2,MDM2	PLD3
Ephrin Receptor Signaling	5.89E-10	AKT3,GRIN2A,PAK6	CDC42,AKT1,GNG10,GNB1,RGS3,ARPC1B,GRIN1,GRIN3B,STAT3,FYN,CREBBP,SDCBP,GRIN3A,MAPK3,DOK1,EFNB1,SRC,JAK2,EPHB6,GNB2L1,EP300,ABL1,GNB5,NCK2,BCAR1,RASA1,PAK4	PDGFB,EPHA2,ARPC3,GNAS
eNOS Signaling	8.51E-10	CHRNA5,AKT3,CHRNA3,PRKD1	AKT1,CALM1 (includes others),PRKAA1,PIK3R1,AQP1,PRKCB,PRKCH,PRKCZ,ESR1,PRKCE,PLCG2,HSPA4,PRKCD,PDPK1,CHRNB4,PRKCA,DNM2,PRKAA2,PLCG1,PRKACA,PRKAB2,PRKCG	PRKAR1B,GNAS,CASP3,PRKAG1
AMPK Signaling	1.12E-09	CHRNA5,AKT3,CHRNA3	MAPK14,PPM1A,SRC,MTOR,IRS2,AKT1,PRKAA1,IRS1,SMARCA4,PIK3R1,INS,PDPK1,CHRNB4,TSC2,PRKAA2,CFTR,PRKACA,EIF4EBP1,PRKAB2,PPP2CA	CAB39,PPP2R1A,PRKAR1B,GNAS,PRKAG1,ACACA
Mouse Embryonic Stem Cell Pluripotency	1.48E-09	AKT3,TCF4	GSK3B,MAP3K7,MAPK14,TCF3,JAK2,ID1,AKT1,ID2,CTNNB1,JAK3,JAK1,ID3,PIK3R1,TP53,APC,SMAD9,STAT3,ID4,SMAD1,CREBBP,MAPK3	FZD3
Neuregulin Signaling	1.66E-09	AKT3,PRKD1	PRKCE,EGFR,SRC,MTOR,AKT1,PICK1,PIK3R1,CDK5,PLCG2,PRKCD,PDPK1,DLG4,PRKCA,PRKCB,PLCG1,PRKCH,PRKCZ,PRKCG,MAPK3,PSEN1	PTEN
Breast Cancer Regulation by Stathmin1	1.78E-09	PRKD1	CAMK2B,PPP1CC,PPP1R14A,CDC42,CALM1 (includes others),GNG10,ARHGEF12,GNB1,PIK3R1,TP53,CCNE1,CDKN1A,PRKCB,PRKCH,PRKCZ,PPP2CA,MAPK3,PRKCE,GNB2L1,CAMK1D,CAMK2A,PRKCD,PRKCA,GNB5,ARHGEF1,TUBA1B,PRKACA,CDK1,PRKCG	TUBA1C,PPP2R1A,PRKAR1B,GNAS,PRKAG1
Glioblastoma Multiforme Signaling	1.91E-09	AKT3,PLCH2	GSK3B,CDC42,AKT1,PIK3R1,TP53,APC,CCNE1,TSC2,CDKN1A,CDK6,MAPK3,EGFR,MTOR,SRC,TCF3,CTNNB1,WNT7B,RHOJ,PLCG2,IGF1R,PRKCD,CCND1,PLCG1,MDM2	PTEN,PDGFB,FZD3,PDIA3

HGF Signaling	2.24E-09	AKT3,PRKD1	JUN,MAP2K7,CDC42,AKT1,PIK3R1,FOS,STAT3,CDKN1A,PRKCB,MAPK8,PRKCH,PRKCZ,MAPK9,MAPK3,PRKCE,MAP3K7,PLCG2,PRKCD,PRKCA,CCND1,PTGS2,PLCG1,PRKCG	
EGF Signaling	3.55E-09	AKT3	JUN,MAP2K7,MAPK14,EGFR,SRC,MTOR,AKT1,CSNK2B,JAK1,PIK3R1,FOS,STAT3,PRKCA,PLCG1,MAPK8,RASA1,MAPK3	
Colorectal Cancer Metastasis Signaling	3.72E-09	AKT3,TCF4	JUN,GSK3B,AKT1,GNG10,JAK3,GNB1,PIK3R1,TP53,APC,FOS,STAT3,MAPK8,MAPK9,MAPK3,NFKB1,EGFR,SRC,TCF3,JAK2,GNB2L1,CTNNB1,WNT7B,SMAD2,JAK1,RHOJ,RELA,GNB5,CCND1,PTGS2,PRKACA,PTGER3	PRKAR1B,LRP1,FZD3,GNAS,CASP3,PRKAG1
Melatonin Signaling	3.98E-09	PLCH2,PRKD1	PRKCE,CAMK2B,MAP2K7,CALM1 (includes others),CAMK2A,PLCG2,PRKCD,PRKCA,PRKCB,PLCG1,PRKCH,PRKACA,PRKCZ,PRKCG,MAPK3	PDIA3,PRKAR1B,PRKAG1
Cyclins and Cell Cycle Regulation	4.90E-09	NONE	GSK3B,HDAC5,HDAC2,CDK7,CDKN2C,CDC25A,TP53,HDAC7,ABL1,SUV39H1,CCND1,CCNE1,CDKN1A,SIN3A,HDAC4,CDK6,HDAC1,CDK1,PPP2CA	CCND2,PPP2R1A
Cell Cycle: G1/S Checkpoint Regulation	5.01E-09	NONE	GSK3B,HDAC5,HDAC2,CDKN2C,CDC25A,MAX,TP53,HDAC7,ABL1,SUV39H1,CCND1,CCNE1,CDKN1A,SIN3A,MDM2,HDAC4,CDK6,HDAC1	CCND2
Thrombin Signaling	6.17E-09	AKT3,PLCH2,PRKD1	CAMK2B,MAPK14,AKT1,GNG10,ARHGEF12,GNB1,PIK3R1,PRKCB,PRKCH,PRKCZ,MAPK3,PRKCE,NFKB1,EGFR,SRC,GNB2L1,CAMK1D,RHOJ,CAMK2A,RELA,PLCG2,PRKCD,PDPK1,PRKCA,GNB5,ARHGEF1,PLCG1,PRKCG	MYL9,GNAS,PDIA3
Hypoxia Signaling in the Cardiovascular System	6.61E-09	NONE	JUN,UBE2M,AKT1,EP300,UBE2E3,UBE2E1,TP53,SUMO1,UBE2D3,UBE2E2,UBE2S,VHL,COPS5,UBE2D2,CDC34,CREBBP,MDM2,UBE2D1	PTEN
LPS-stimulated MAPK Signaling	8.32E-09	PRKD1	JUN,PRKCE,MAP3K7,NFKB1,MAPK14,CDC42,CHUK,PIK3R1,RELA,FOS,PRKCD,PRKCA,PRKCB,MAPK8,PRKCH,PRKCZ,MAPK9,PRKCG,MAPK3	
ILK Signaling	1.10E-08	AKT3	JUN,GSK3B,CDC42,AKT1,IRS1,PIK3R1,MUC1,FOS,CREBBP,MAPK8,MAPK9,PPP2CA,MAPK3,NFKB1,MTOR,IRS2,FLNA,CTNNB1,EP300,ACTN1,RHOJ,RELA,PDPK1,CCND1,NCK2,PTGS2	PTEN,GSK3A,SNAI2,PPP2R1A,MYL9,CASP3
nNOS Signaling in Neurons	1.10E-08	GRIN2A,PRKD1	PRKCE,CALM1 (includes others),GRIN1,CAMK2A,GRIN3B,PRKCD,DLG4,PRKCA,PRKCB,PRKCH,PRKCZ,GRIN3A,DLG2,PRKCG	
Ovarian Cancer Signaling	1.12E-08	AKT3,TCF4	GSK3B,BRCA1,EGFR,SRC,TCF3,MTOR,AKT1,CTNNB1,WNT7B,PIK3R1,TP53,APC,ABL1,SUV39H1,CCND1,BCL2,PTGS2,SIN3A,PRKACA,PTGS1,MAPK3	PTEN,PRKAR1B,FZD3,PRKAG1
Wnt/Î²-catenin Signaling	1.45E-08	AKT3,TCF4	JUN,GSK3B,CSNK1A1,AKT1,UBD,TLE1,TP53,APC,CREBBP,PIN1,HDAC1,TLE4,UBC,PPP2CA,MAP3K7,SRC,TCF3,CSNK2B,CTNNB1,WNT7B,EP300,PPARD,CCND1,MDM2	GSK3A,PPP2R1A,LRP1,FZD3,ACVR1B

Growth Hormone Signaling	1.78E-08	PRKD1	PRKCE,JAK2,IRS1,PIK3R1,PLCG2,FOS,IGF1R,STAT3,PRKCD,PDPK1,PRKCA,PRKCB,PLCG1,PRKCH,PRKCZ,PRKCG,MAPK3	RPS6KA3
Insulin Receptor Signaling	1.78E-08	AKT3	GRB10,GSK3B,PPP1CC,PPP1R14A,MTOR,IRS2,JAK2,AKT1,JAK1,IRS1,PIK3R1,INS,FYN,PDPK1,TSC2,MAPK8,PRKACA,PRKCZ,EIF4EBP1,MAPK3	PTEN,GSK3A,INPP5K,ASIC2,PRKAR1B,PRKAG1
Estrogen-Dependent Breast Cancer Signaling	1.78E-08	AKT3	JUN,NFKB1,EGFR,SRC,AKT1,EP300,PIK3R1,SP1,TERT,RELA,FOS,IGF1R,CCND1,CREBBP,ESR1,MAPK3	HSD17B12
Factors Promoting Cardiogenesis in Vertebrates	1.78E-08	TCF4,PRKD1	PRKCE,GSK3B,MAP3K7,MAPK14,TCF3,CTNNB1,SMAD2,APC,SMAD9,PRKCD,PRKCA,CCNE1,SMAD1,PRKCB,PRKCH,PRKCZ,PRKCG	ACVR1B,LRP1,FZD3
Thrombopoietin Signaling	1.78E-08	PRKD1	JUN,PRKCE,IRS2,JAK2,PIK3R1,PLCG2,FOS,STAT3,PRKCD,PRKCA,PRKCB,PLCG1,PRKCH,PRKCZ,PRKCG,MAPK3	
VDR/RXR Activation	2.51E-08	PRKD1	PRKCE,EP300,SEMA3B,SP1,PPARD,PRKCD,PRKCA,CDKN1A,PRKCB,PRKCH,SPP1,PRKCZ,PRKCG	CSF2,CASR,HOXA10,YY1,VDR,SULT2A1
Small Cell Lung Cancer Signaling	2.82E-08	AKT3	NFKB1,CHUK,AKT1,PIK3R1,MAX,TP53,RELA,ABL1,SUV39H1,CCND1,BCL2,CCNE1,PTGS2,SIN3A,CDK6,CKS1B,TRAF6	PTEN
IL-17A Signaling in Airway Cells	2.95E-08	AKT3	GSK3B,MAP3K7,NFKB1,MAPK14,CHUK,JAK2,AKT1,JAK3,JAK1,PIK3R1,RELA,STAT3,MAPK8,MAPK9,TRAF6,MAPK3	PTEN
Xenobiotic Metabolism Signaling	4.79E-08	PRKD1,CUL3	CAMK2B,MAP2K7,MAPK14,PIK3R1,SUMO1,PRKCB,CREBBP,GRIP1,MAPK8,PRKCH,HDAC4,PRKCZ,MAPK9,PPP2CA,MAPK3,PRKCE,MAP3K7,HDAC5,NFKB1,EP300,CAMK1D,CAMK2A,RELA,PRKCD,PRKCA,PTGES3,FTL,KEAP1,RBX1,PRKCG	PPP2R1A,ALDH1B1,CHST12,SRA1,UGT2B4,ALDH6A1,SULT2A1,HS3ST1
Virus Entry via Endocytic Pathways	4.90E-08	PRKD1	PRKCE,CDC42,SRC,FOLR1,FLNA,AP2B1,PIK3R1,PLCG2,ABL1,PRKCD,FYN,PRKCA,DNM2,AP2A2,PRKCB,PLCG1,PRKCH,PRKCZ,PRKCG	CXADR
ATM Signaling	5.01E-08	NONE	JUN,BRCA1,MAPK14,CBX5,EP300,CDC25A,TP53,ABL1,TRIM28,CDKN1A,CREBBP,MAPK8,MDM2,MAPK9,CDK1	SMC1B,BLM
Hereditary Breast Cancer Signaling	5.62E-08	AKT3	HDAC5,BRCA1,HDAC2,AKT1,EP300,UBD,BARD1,SMARCA4,PIK3R1,TP53,HDAC7,RFC5,CCND1,CDKN1A,CREBBP,HDAC4,CDK6,HDAC1,CDK1,UBC	PTEN,BLM,POLR2I
NRF2-mediated Oxidative Stress Response	5.62E-08	PRKD1,CUL3	JUN,GSK3B,MAP2K7,MAPK14,AKT1,UBE2E3,PIK3R1,ENC1,FOS,FKBP5,CDC34,PRKCB,CREBBP,MAPK8,PRKCH,PRKCZ,MAPK9,MAPK3,PRKCE,MAP3K7,EP300,DNAJC8,PRKCD,PRKCA,FTL,KEAP1,RBX1,PRKCG	DNAJC10
p53 Signaling	5.62E-08	STAG1,AKT3	JUN,GSK3B,BRCA1,MAPK14,AKT1,CTNNB1,EP300,PIK3R1,PIAS1,TP53,TRIM29,CCND1,BCL2,CDKN1A,MAPK8,MDM2,HDAC1	PTEN,CCND2,SNAI2
CXCR4 Signaling	6.31E-08	AKT3,PAK6,PRKD1	JUN,AKT1,GNG10,GNB1,PIK3R1,FOS,PRKCB,MAPK8,PRKCH,PRKCZ,MAPK9,MAPK3,PRKCE,SRC,GNB2L1,EGR1,RHOJ,PRKCD,PRKCA,GNB5,BCAR1,PAK4,PRKCG	MYL9,GNAS

Gap Junction Signaling	9.77E-08	AKT3,PLCH2,PRKD1	PRKCE,CSNK1A1,EGFR,SRC,AKT1,CTNNB1,PIK3R1,SP1,PLCG2,PRKCD,PRKCA,TUBA1B,PRKCB,PLCG1,PRKCH,PRKACA,PRKCZ,PRKCG,MAPK3	TUBA1C,PRKAR1B,GNAS,SP3,PDIA3,PRKAG1
PDGF Signaling	1.05E-07	NONE	JUN,SRC,JAK2,CSNK2B,JAK3,JAK1,PIK3R1,PLCG2,ABL1,FOS,STAT3,PRKCA,PRKCB,PLCG1,MAPK8,RASA1,MAPK3	PDGFB,INPP5K
Chronic Myeloid Leukemia Signaling	1.05E-07	AKT3	CTBP1,HDAC5,HDAC2,NFKB1,CHUK,AKT1,PIK3R1,TP53,HDAC7,RELA,ABL1,SUV39H1,CCND1,CDKN1A,SIN3A,MDM2,HDAC4,CDK6,HDAC1,MAPK3	
Corticotropin Releasing Hormone Signaling	1.23E-07	PRKD1	JUN,PRKCE,MAPK14,CALM1 (includes others),NR4A1,EP300,PLCG2,FOS,PRKCD,PRKCA,PTGS2,PRKCB,CREBBP,PLCG1,PRKCH,PRKACA,PRKCZ,PRKCG,MAPK3	GNAS,PRKAR1B,PRKAG1
Cell Cycle: G2/M DNA Damage Checkpoint Regulation	1.35E-07	NONE	BRCA1,YWHAQ,YWHAG,CDK7,AURKA,EP300,TP53,ABL1,CDKN1A,MDM2,CKS1B,YWHAZ,PRKCZ,YWHAB,CDK1	
IL-3 Signaling	1.48E-07	AKT3,PRKD1	JUN,PRKCE,JAK2,AKT1,CSF2RB,JAK1,PIK3R1,FOS,STAT3,PRKCD,PRKCA,PRKCB,PRKCH,PRKCZ,PRKCG,MAPK3	
Calcium Signaling	1.48E-07	NFATC3,CHRNA5,GRIN2A,CHRNA3,ATP2A2	CAMK2B,CALM1 (includes others),GRIN1,GRIN3B,CREBBP,TPM1,HDAC4,HDAC1,GRIN3A,MAPK3,HDAC5,HDAC2,EP300,CAMK1D,CAMK2A,HDAC7,TPM3,CHRNB4,PRKACA,RYR2	GRIA2,PRKAR1B,MYL9,ASPH,PRKAG1
Î±-Adrenergic Signaling	1.51E-07	PRKD1	PRKCE,CALM1 (includes others),GNB2L1,GNG10,GNB1,PLCG2,PRKCD,PRKCA,GNB5,PRKCB,PLCG1,PRKCH,PRKACA,PRKCZ,PRKCG,MAPK3	GNAS,PRKAR1B,PRKAG1
Aryl Hydrocarbon Receptor Signaling	1.58E-07	NONE	JUN,NFKB1,SRC,MCM7,EP300,SMARCA4,SP1,TP53,RELA,FOS,HSPB1,HSPB7,CCND1,PTGES3,CCNE1,CDKN1A,NR2F1,MAPK8,MDM2,CDK6,NEDD8,ESR1,MAPK3	ALDH1B1,CCND2,ALDH6A1
Calcium-induced T Lymphocyte Apoptosis	1.62E-07	PRKD1,ATP2A2	PRKCE,HDAC2,CALM1 (includes others),NR4A1,EP300,PRKCD,PRKCA,HLA-DMB,PRKCB,PLCG1,PRKCH,PRKCZ,HDAC1,PRKCG	ZAP70
CD40 Signaling	2.09E-07	NONE	JUN,MAP2K7,MAP3K7,NFKB1,MAPK14,CHUK,JAK3,PIK3R1,RELA,FOS,STAT3,PTGS2,MAPK8,PTGS1,MAPK9,TRAF6,MAPK3	
Amyloid Processing	2.29E-07	AKT3	PRKCE,GSK3B,CSNK1A1,MAPK14,AKT1,CSNK2B,APP,CDK5,PRKACA,MAPK3,PSEN1	APH1B,PRKAR1B,PRKAG1
Nitric Oxide Signaling in the Cardiovascular System	2.88E-07	AKT3,CACNA1C,PRKD1,ATP2A2	PRKCE,CALM1 (includes others),AKT1,PRKAA1,PIK3R1,PLN,PRKCD,PRKCA,PRKCB,PRKCH,PRKACA,PRKCZ,RYR2,PRKCG,MAPK3	PRKAR1B,PRKAG1
Telomerase Signaling	2.88E-07	AKT3	POT1,HDAC5,HDAC2,EGFR,AKT1,PIK3R1,SP1,TP53,TERT,HDAC7,ABL1,PDPK1,TERF1,PTGES3,CDKN1A,HDAC4,HDAC1,PPP2CA,MAPK3	PPP2R1A

fMLP Signaling in Neutrophils	3.02E-07	NFATC3,PRKD1	PRKCE,NFKB1,CDC42,CALM1 (includes others),GNB2L1,GNG10,GNB1,PIK3R1,ARPC1B,RELA,PRKCD,PRKCA,GNB5,PRKCB,PRKCH,PRKCZ,PRKCG,MAPK3	ARPC3,GNAS
Adipogenesis pathway	3.47E-07	NONE	CTBP1,HDAC5,HDAC2,CDK7,AKT1,RUNX1T1,RBBP7,CDK5,TP53,NR2F2,HDAC7,SMAD9,SIN3B,EZH2,SMAD1,SIN3A,EGR2,RBBP4,HDAC4,HDAC1,ERCC3	GTF2H2,FZD3,SAP30L
ErbB4 Signaling	3.55E-07	PRKD1	PRKCE,AKT1,PIK3R1,PLCG2,PRKCD,PDPK1,PRKCA,PRKCB,PLCG1,PRKCH,PRKCZ,PRKCG,MAPK3,PSEN1	APH1B
Cholecystokinin/Gastrin-mediated Signaling	3.98E-07	PRKD1	JUN,PRKCE,MAPK14,EGFR,SRC,RHOJ,FOS,PRKCD,PRKCA,PTK2B,PTGS2,BCAR1,PRKCB,MAPK8,PRKCH,PRKCZ,MAPK9,PRKCG,MAPK3	CREM
Natural Killer Cell Signaling	4.07E-07	AKT3,PAK6,PRKD1	PRKCE,AKT1,PIK3R1,VAV2,PLCG2,PRKCD,SYK,FYN,PRKCA,PRKCB,PLCG1,PRKCH,PAK4,PRKCZ,PRKCG,MAPK3	LCP2,INPP5K,ZAP70
VEGF Family Ligand-Receptor Interactions	4.07E-07	AKT3,PRKD1	PRKCE,AKT1,PIK3R1,PLCG2,FOS,PRKCD,PRKCA,PRKCB,PLCG1,PRKCH,PRKCZ,PRKCG,MAPK3	PLA2G10,PLA2G6,NRP1
Role of Osteoblasts Osteoclasts and Chondrocytes in Rheumatoid Arthritis	4.27E-07	NFATC3,AKT3,TCF4	JUN,GSK3B,CSNK1A1,MAP2K7,MAPK14,AKT1,CALM1 (includes others),PIK3R1,APC,FOS,MAPK8,SPP1,MAPK9,MAPK3,TRAF6,MAP3K7,NFKB1,CHUK,SRC,TCF3,CTNNB1,WNT7B,RELA,SMAD9,PTK2B,BCL2,SMAD1	CSF2,LRP1,FZD3
Sertoli Cell-Sertoli Cell Junction Signaling	4.57E-07	AKT3,MPP6	JUN,GSK3B,MAP2K7,MAP3K7,MAPK14,CDC42,SRC,DLG1,AKT1,CTNNB1,JUP,CLDN19,ACTN1,TUBA1B,BCAR1,MAPK8,PRKACA,KEAP1,MAPK9,MAPK3,SPTAN1	PTEN,GSK3A,TUBA1C,PRKAR1B,CLDN18,PRKAG1
Cardiac Hypertrophy Signaling	6.46E-07	CACNA1C,PLCH2	JUN,GSK3B,MAP2K7,MAPK14,AKT1,CALM1 (includes others),GNG10,GNB1,IRS1,PIK3R1,CREBBP,MAPK8,MAPK9,MAPK3,MAP3K7,MTOR,GNB2L1,EP300,HAND2,RHOJ,PLCG2,IGF1R,HSPB1,GNB5,PLCG1,PRKACA	PRKAR1B,MYL9,GNAS,PDIA3,PRKAG1
Renal Cell Carcinoma Signaling	7.41E-07	AKT3,PAK6	JUN,CDC42,AKT1,EP300,UBD,PIK3R1,FOS,VHL,TCEB1,CREBBP,PAK4,RBX1,UBC,MAPK3	PDGFB
RANK Signaling in Osteoclasts	8.13E-07	AKT3	JUN,MAP2K7,MAP3K7,NFKB1,MAPK14,SRC,CHUK,CALM1 (includes others),AKT1,PIK3R1,RELA,FOS,PTK2B,MAPK8,MAPK9,TRAF6,MAPK3	MITF
NGF Signaling	1.02E-06	AKT3	MAP3K7,NFKB1,CDC42,CHUK,AKT1,EP300,PIK3R1,TP53,RELA,PLCG2,PRKCD,PDPK1,CREBBP,PLCG1,MAPK8,PRKCZ,MAPK9,TRAF6,MAPK3	RPS6KA3
IL-12 Signaling and Production in Macrophages	1.02E-06	AKT3,PRKD1	JUN,PRKCE,NFKB1,MAPK14,CHUK,AKT1,EP300,PIK3R1,RELA,FOS,PRKCD,PRKCA,PRKCB,MAPK8,PRKCH,PRKCZ,MAPK9,PRKCG,TRAF6,MAPK3	PCYOX1,APOL1
PTEN Signaling	1.35E-06	AKT3	GSK3B,NFKB1,CDC42,EGFR,CHUK,AKT1,CSNK2B,PIK3R1,RELA,IGF1R,PDPK1,CCND1,BCL2,CDKN1A,BCAR1,PRKCZ,MAPK3	PTEN,GSK3A,INPP5K,CASP3
IL-1 Signaling	1.35E-06	NONE	JUN,MAP2K7,MAP3K7,NFKB1,MAPK14,CHUK,GNB2L1,GNG10,GNB1,RELA,FOS,GNB5,MAPK8,PRKACA,MAPK9,TRAF6	GNAS,PRKAR1B,PRKAG1

CD28 Signaling in T Helper Cells	1.35E-06	NFATC3,AKT3	JUN,NFKB1,CDC42,CHUK,CALM1 (includes others),AKT1,PIK3R1,ARPC1B,RELA,FOS,SYK,FYN,PDPK1,HLA-DMB,PLCG1,MAPK8,MAPK9	ARPC3,LCP2,ZAP70
mTOR Signaling	1.38E-06	AKT3,PRKD1	EIF3G,AKT1,PRKAA1,IRS1,PIK3R1,RPS20,TSC2,PRKCB,PRKCH,PRKCZ,EIF4EBP1,PPP2CA,MAPK3,PRKCE,MTOR,INS,RHOJ,PRKCD,PDPK1,PRKCA,PRKAA2,PRKAB2,PRKCG	PLD3,PPP2R1A,RPS6KA3,PRKAG1
GPCR-Mediated Nutrient Sensing in Enteroendocrine Cells	1.78E-06	PLCH2,PRKD1	PRKCE,GNG10,PLCG2,PRKCD,PRKCA,PRKCB,PLCG1,PRKCH,PRKACA,PRKCZ,PRKCG	CASR,GNAS,PDIA3,PRKAR1B,PRKAG1
Role of NFAT in Regulation of the Immune Response	2.09E-06	NFATC3,AKT3	JUN,GSK3B,CSNK1A1,NFKB1,CHUK,CALM1 (includes others),AKT1,GNB2L1,GNG10,BTK,GNB1,PIK3R1,RELA,PLCG2,FOS,SYK,FYN,GNB5,HLA-DMB,PLCG1,MAPK3	GSK3A,LCP2,ZAP70,GNAS
Endothelin-1 Signaling	2.09E-06	PLCH2,PRKD1	JUN,PRKCE,MAPK14,SRC,PIK3R1,PLCG2,FOS,PRKCD,PRKCA,PTGS2,PRKCB,PLCG1,MAPK8,PRKCH,PRKCZ,PTGS1,MAPK9,PRKCG,MAPK3	PLD3,PLA2G6,PLA2G10,GNAS,PDIA3,CASP3
Regulation of the Epithelial-Mesenchymal Transition Pathway	2.82E-06	AKT3,TCF4	GSK3B,MAP2K7,AKT1,JAK3,PIK3R1,APC,STAT3,TWIST1,MAPK3,PSEN1,NFKB1,FGF12,EGFR,TCF3,JAK2,ID2,CTNNB1,WNT7B,SMAD2,JAK1,EGR1,RELA,TWIST2	SNAI2,FZD3,APH1B
TGF-Î² Signaling	3.02E-06	NONE	JUN,MAP3K7,MAPK14,CDC42,SMAD2,EP300,SMAD9,FOS,BCL2,SMAD1,CREBBP,MAPK8,HDAC1,MAPK9,TRAF6,MAPK3	ACVR1B,VDR
Regulation of IL-2 Expression in Activated and Anergic T Lymphocytes	3.31E-06	NFATC3	JUN,MAP2K7,NFKB1,CHUK,CALM1 (includes others),SMAD2,VAV2,RELA,PLCG2,FOS,FYN,PLCG1,MAPK8,MAPK9,MAPK3	ZAP70
Chemokine Signaling	3.55E-06	NONE	JUN,CAMK2B,MAPK14,SRC,CALM1 (includes others),CAMK1D,CAMK2A,PLCG2,FOS,PRKCA,PTK2B,PRKCB,PLCG1,MAPK8,MAPK3	CCL4
UVA-Induced MAPK Signaling	3.55E-06	PLCH2	JUN,MAPK14,EGFR,MTOR,PIK3R1,TP53,PLCG2,FOS,PARP1,PRKCA,PLCG1,MAPK8,MAPK9,MAPK3	RPS6KA3,CASP3,PDIA3
CCR3 Signaling in Eosinophils	4.27E-06	PAK6,PRKD1	PRKCE,MAPK14,CALM1 (includes others),GNB2L1,GNG10,GNB1,PIK3R1,PRKCD,PRKCA,GNB5,PRKCB,PRKCH,PAK4,PRKCZ,PRKCG,MAPK3	PLA2G10,PLA2G6,GNAS
IL-17 Signaling	4.27E-06	AKT3	JUN,GSK3B,MAP3K7,NFKB1,MAPK14,JAK2,AKT1,JAK1,PIK3R1,RELA,PTGS2,MAPK8,MAPK9,TRAF6,MAPK3	
JAK/Stat Signaling	4.27E-06	AKT3	JUN,NFKB1,MTOR,JAK2,AKT1,JAK3,JAK1,PIK3R1,PIAS1,RELA,FOS,STAT3,CDKN1A,MAPK3	PIAS2
G-Protein Coupled Receptor Signaling	4.68E-06	AKT3,GRM3	CAMK2B,TDP2,AKT1,PIK3R1,GLP1R,STAT3,FYN,PDE7B,PRKCB,CREBBP,MAPK3,PRKCE,NFKB1,CHUK,SRC,EP300,CAMK2A,RELA,PDPK1,PRKCA,PTK2B,RASA1,PRKACA,APEX1,PTGER3,PRKCG	HRH1,PRKAR1B,RGS7,HCAR3,GNAS,PRKAG1
Leptin Signaling in Obesity	6.03E-06	AKT3,PLCH2	STAT3,AKT1,JAK2,PLCG1,PIK3R1,INS,PRKACA,MAPK3,PLCG2	GHRL,GNAS,PDIA3,PRKAR1B,PRKAG1
Role of Tissue Factor in Cancer	6.03E-06	AKT3	MAPK14,CDC42,EGFR,SRC,MTOR,JAK2,AKT1,EGR1,PIK3R1,TP53,FYN,PRKCA,PTK2B,PDIA2,MAPK3	PTEN,CSF2,RPS6KA3,CASP3
Sperm Motility	6.31E-06	PLCH2,PRKD1	PRKCE,CALM1 (includes others),PLCG2,PRKCD,PRKCA,PTK2B,PRKCB,PLCG1,PRKCH,PRKACA,PRKCZ,PRKCG	PLA2G10,PLA2G6,CACNA1H,GNAS,PDIA3,PRKAR1B,PRKAG1

Myc Mediated Apoptosis Signaling	6.46E-06	AKT3	YWHAQ,YWHAG,AKT1,PIK3R1,TP53,IGF1R,BCL2,MAPK8,YWHAZ,PRKCZ,YWHAB,MAPK9	CASP3
IL-15 Signaling	6.46E-06	AKT3	NFKB1,MAPK14,JAK2,AKT1,JAK3,JAK1,PIK3R1,RELA,STAT3,SYK,BCL2,PLCG1,MAPK3	CSF2
NF-Î°B Signaling	7.76E-06	AKT3	GSK3B,MAP2K7,TDP2,AKT1,PIK3R1,PELI1,PRKCB,CREBBP,MAPK8,PRKCZ,HDAC1,TRAF6,MAP3K7,NFKB1,EGFR,HDAC2,CHUK,CSNK2B,EP300,INS,RELA,PLCG2,IGF1R,PRKACA	ZAP70
iNOS Signaling	8.71E-06	NONE	FOS,JUN,MAPK14,NFKB1,CHUK,CALM1 (includes others),JAK2,JAK3,JAK1,CREBBP,TRAF6,RELA	
Macropinocytosis Signaling	9.33E-06	PRKD1	PRKCE,CDC42,SRC,PIK3R1,INS,PLCG2,PRKCD,PRKCA,PRKCB,PLCG1,PRKCH,PRKCZ,PRKCG	PDGFB
April Mediated Signaling	1.17E-05	NFATC3	FOS,JUN,MAP2K7,MAPK14,NFKB1,CHUK,MAPK8,MAPK9,TRAF6,RELA	
IL-17A Signaling in Gastric Cells	1.20E-05	NONE	FOS,JUN,MAPK14,NFKB1,EGFR,MAPK8,MAPK9,RELA,MAPK3	
Semaphorin Signaling in Neurons	1.20E-05	PAK6,FES	PLXNA1,FYN,ARHGEF12,PAK4,CDK5,RHOJ,DPYSL5,MAPK3	RND1,NRP1,SEMA7A
IL-6 Signaling	1.29E-05	AKT3	JUN,MAP2K7,MAP3K7,NFKB1,MAPK14,CHUK,JAK2,AKT1,CSNK2B,PIK3R1,RELA,FOS,HSPB1,STAT3,HSPB7,MAPK8,MAPK9,TRAF6,MAPK3	
GÎ±12/13 Signaling	1.48E-05	AKT3	JUN,MAP2K7,NFKB1,CDC42,SRC,CHUK,AKT1,CTNNB1,BTK,PIK3R1,VAV2,RELA,PTK2B,ARHGEF1,MAPK8,RASA1,MAPK9,MAPK3	MYL9
DNA Methylation and Transcriptional Repression Signaling	1.62E-05	NONE	MTA2,HDAC2,DNMT1,SIN3A,RBBP7,RBBP4,MBD3,HDAC1	
Apoptosis Signaling	1.62E-05	NONE	PRKCE,MAP2K7,NFKB1,CHUK,TP53,ACIN1,RELA,PLCG2,PARP1,PRKCA,BCL2,PLCG1,MAPK8,CDK1,MAPK3,SPTAN1	CASP3
B Cell Activating Factor Signaling	1.95E-05	NFATC3	FOS,JUN,MAP2K7,MAPK14,NFKB1,CHUK,MAPK8,MAPK9,TRAF6,RELA	
G Protein Signaling Mediated by Tubby	1.95E-05	NONE	ABL1,JAK2,GNB2L1,GNB5,GNG10,GNB1,PLCG1,INS,PLCG2	GNAS
Pyridoxal 5'-phosphate Salvage Pathway	2.04E-05	NONE	PRKCE,CSNK1A1,NEK2,CDK7,PRKAA1,CDK5,PRKCD,PRKAA2,MAPK8,PRKCH,CDK6,MAPK9,CDK1,MAPK3	
Wnt/Ca+ pathway	2.19E-05	NFATC3,PLCH2	GSK3B,NFKB1,PRKCA,EP300,CREBBP,PLCG1,CAMK2A,RELA,PLCG2	PDIA3,FZD3
NF-Î°B Activation by Viruses	2.19E-05	AKT3,PRKD1	PRKCE,NFKB1,CHUK,AKT1,PIK3R1,RELA,PRKCD,PRKCA,PRKCB,PRKCH,PRKCZ,PRKCG,MAPK3	
Synaptic Long Term Depression	2.24E-05	PLCH2,PRKD1,GRM3	PRKCE,PLCG2,IGF1R,PRKCD,PRKCA,PRKCB,PLCG1,PRKCH,PRKCZ,RYR2,PPP2CA,PRKCG,MAPK3	PLA2G10,PLA2G6,GNAS,GRIA2,PPP2R1A,PDIA3
MIF Regulation of Innate Immunity	2.40E-05	NONE	FOS,JUN,NFKB1,PTGS2,MAPK8,TP53,MAPK9,RELA,MAPK3	PLA2G10,PLA2G6
Non-Small Cell Lung Cancer Signaling	2.40E-05	AKT3	EGFR,AKT1,PIK3R1,TP53,ABL1,PDPK1,PRKCA,SUV39H1,CCND1,SIN3A,PLCG1,CDK6,MAPK3	

HIF1Î± Signaling	2.57E-05	AKT3	JUN,MAPK14,AKT1,EP300,PIK3R1,TP53,VHL,COPS5,EGLN2,TCEB1,CREBBP,MAPK8,MDM2,APEX1,RBX1,MAPK9,MAPK3	
IL-17A Signaling in Fibroblasts	3.31E-05	NONE	FOS,JUN,GSK3B,MAP3K7,MAPK14,NFKB1,CHUK,TRAF6,RELA,MAPK3	
Epithelial Adherens Junction Signaling	3.80E-05	AKT3,TCF4	CDC42,EGFR,SRC,TCF3,AKT1,CTNNB1,JUP,ACTN1,ARPC1B,APC,VAV2,BAIAP2,TUBA1B,KEAP1	PTEN,ARPC3,MYL9,TUBA1C,ACVR1B,SNAI2
HIPPO signaling	3.98E-05	NONE	PPP1CC,PPP1R14A,YWHAQ,DLG1,YWHAG,SMAD2,DLG3,INADL,DLG4,SMAD1,YWHAZ,PRKCZ,YWHAB,DLG2,PPP2CA	PPP2R1A
Acute Myeloid Leukemia Signaling	4.07E-05	AKT3,TCF4	MAP2K7,NFKB1,TCF3,MTOR,AKT1,CSF2RB,JUP,PIK3R1,RELA,STAT3,CCND1,EIF4EBP1,MAPK3	
T Cell Receptor Signaling	4.79E-05	NFATC3	JUN,NFKB1,CHUK,CALM1 (includes others),BTK,PIK3R1,VAV2,RELA,FOS,FYN,PLCG1,MAPK8,RASA1,MAPK3	LCP2,ZAP70
Antioxidant Action of Vitamin C	4.79E-05	PLCH2	MAPK14,NFKB1,CHUK,JAK2,CSF2RB,PLCG1,MAPK8,MAPK9,RELA,MAPK3,PLCG2	CSF2,PLA2G10,PLD3,PLA2G6,PDIA3
PKCÎ¸ Signaling in T Lymphocytes	5.50E-05	NFATC3	JUN,CAMK2B,MAP3K7,NFKB1,CHUK,PIK3R1,CAMK2A,VAV2,RELA,PLCG2,FOS,FYN,HLA-DMB,PLCG1,MAPK8,MAPK3	LCP2,ZAP70
IL-2 Signaling	5.89E-05	AKT3	FOS,JUN,SYK,AKT1,PTK2B,CSNK2B,JAK3,JAK1,MAPK8,PIK3R1,MAPK3	
Hepatic Cholestasis	6.31E-05	PRKD1	JUN,PRKCE,MAP3K7,NFKB1,CHUK,INS,RELA,PRKCD,PRKCA,PRKCB,MAPK8,PRKCH,PRKACA,PRKCZ,ESR1,MAPK9,PRKCG,TRAF6	CSF2,GNAS,PRKAR1B,PRKAG1
GM-CSF Signaling	6.31E-05	AKT3	CAMK2B,STAT3,AKT1,JAK2,GNB2L1,CSF2RB,CCND1,PRKCB,PIK3R1,CAMK2A,MAPK3	CSF2
Protein Ubiquitination Pathway	6.31E-05	NONE	UBE2M,THOP1,UBE2E3,UBD,PSMA3,UBE2E2,UBE2S,HSPB7,CDC34,TCEB1,PSMA1,USP4,UBC,TRAF6,BRCA1,PARK2,UBE2E1,UBE2D3,HSPB1,DNAJC8,VHL,HSPA4,NEDD4,USP15,UBE2D2,MDM2,UBE2D1,RBX1,NEDD4L	UBE4B,DNAJC10
IL-22 Signaling	6.76E-05	AKT3	MAPK14,STAT3,AKT1,JAK1,MAPK8,MAPK9,MAPK3	
Lymphotoxin Î² Receptor Signaling	6.76E-05	AKT3	NFKB1,PDPK1,CHUK,AKT1,EP300,CREBBP,PIK3R1,TRAF6,RELA,MAPK3	CASP3
Aldosterone Signaling in Epithelial Cells	6.76E-05	PLCH2,PRKD1	PRKCE,PIK3R1,PLCG2,HSPB1,DNAJC8,NEDD4,HSPA4,PRKCD,PDPK1,PRKCA,HSPB7,PRKCB,PLCG1,PRKCH,PRKCZ,PRKCG,MAPK3	DNAJC10,ASIC2,PDIA3
HMGB1 Signaling	6.76E-05	AKT3	JUN,MAP2K7,NFKB1,MAPK14,CDC42,AKT1,RBBP7,PIK3R1,RHOJ,SP1,RELA,FOS,KAT6A,HAT1,MAPK8,MAPK9,MAPK3	CSF2
Leukocyte Extravasation Signaling	7.24E-05	PRKD1	MAPK14,CDC42,CLDN19,PIK3R1,PRKCB,MAPK8,PRKCH,PRKCZ,MAPK9,PRKCE,SRC,CTNNB1,BTK,ACTN1,VAV2,PLCG2,ABL1,PRKCD,PRKCA,PTK2B,BCAR1,PLCG1,ARHGAP12,PRKCG	CLDN18

Signaling by Rho Family GTPases	7.76E-05	PAK6	JUN,MAP2K7,CDC42,GNG10,ARHGEF12,GNB1,PIK3R1,ARPC1B,FOS,MAPK8,PRKCZ,MAPK9,MAPK3,NFKB1,CDC42EP4,GNB2L1,RHOJ,RELA,BAIAP2,NEDD4,PTK2B,GNB5,ARHGEF1,PAK4	SEPT3,ARPC3,MYL9,GNAS
Cardiac Î²-adrenergic Signaling	8.71E-05	CACNA1C,ATP2A2	PPP1CC,PPP1R14A,AKAP13,TDP2,GNB2L1,GNG10,GNB1,PLN,GNB5,PDE7B,PRKACA,APEX1,RYR2,PPP2CA	GNAS,PPP2R1A,PRKAR1B,PRKAG1
Integrin Signaling	0.0001	AKT3,PAK6	GSK3B,CDC42,SRC,AKT1,NEDD9,ACTN1,PIK3R1,RHOJ,ARPC1B,PLCG2,ABL1,FYN,NCK2,BCAR1,PLCG1,MAPK8,PAK4,TTN,MAPK3	PTEN,PDGFB,ARF5,ARPC3,MYL9
GADD45 Signaling	0.0001	NONE	BRCA1,CCNE1,CCND1,CDKN1A,TP53,CDK1	CCND2
Relaxin Signaling	0.0001047	AKT3	JUN,NFKB1,TDP2,AKT1,GNB2L1,GNG10,GNB1,PIK3R1,RELA,FOS,GNB5,PDE7B,PRKACA,PRKCZ,APEX1,MAPK3	GNAS,PRKAR1B,PRKAG1
PPAR Signaling	0.0001096	NONE	JUN,MAP3K7,NFKB1,CHUK,EP300,INS,RELA,PPARD,FOS,PTGS2,NR2F1,CREBBP,TRAF6,MAPK3	PDGFB,SRA1
ErbB2-ErbB3 Signaling	0.0001148	NONE	JUN,GSK3B,STAT3,PDPK1,AKT1,CCND1,JAK3,PIK3R1,SP1,MAPK3	PTEN,GSK3A
Glutamate Receptor Signaling	0.0001148	GRIN2A,GRM3	GRIN3B,HOMER3,DLG4,CALM1 (includes others),PICK1,GNB1,GRIP1,GRIN1,GRIN3A	GRIA2
Acute Phase Response Signaling	0.0001148	AKT3,TCF4	JUN,MAP2K7,MAP3K7,NFKB1,MAPK14,TCF3,MTOR,CHUK,JAK2,AKT1,PIK3R1,RELA,FOS,STAT3,PDPK1,FTL,MAPK8,MAPK9,TRAF6,MAPK3	CRABP1
Salvage Pathways of Pyrimidine Ribonucleotides	0.000123	NONE	NME7,PRKCE,CSNK1A1,NEK2,CDK7,PRKAA1,CDK5,PRKCD,PRKAA2,MAPK8,PRKCH,CDK6,MAPK9,CDK1,MAPK3	APOBEC1
Mechanisms of Viral Exit from Host Cells	0.0001288	PRKD1	PRKCE,NEDD4,PRKCD,PRKCA,PRKCB,VPS28,PRKCH,PRKCZ,PRKCG	
BMP signaling pathway	0.0001288	NONE	JUN,MAP3K7,MAPK14,NFKB1,SMAD1,CREBBP,MAPK8,PRKACA,MAPK9,RELA,SMAD9,MAPK3	PRKAR1B,PRKAG1
Estrogen Receptor Signaling	0.0001349	NONE	CTBP1,SRC,CDK7,EP300,HNRNPD,SMARCA4,HIST2H3C,RBFOX2,TAF7L,HIST3H3,CREBBP,GTF2E1,ESR1,ERCC3,MAPK3	GTF2H2,MED23,POLR2I,SRA1
Inhibition of Angiogenesis by TSP1	0.0001479	AKT3	JUN,MAPK14,FYN,AKT1,MAPK8,TP53,MAPK9	CASP3
Melanoma Signaling	0.0001549	AKT3	AKT1,CCND1,CDKN1A,MDM2,PIK3R1,TP53,MAPK3	PTEN,MITF
Role of MAPK Signaling in the Pathogenesis of Influenza	0.0001549	AKT3	MAP2K7,MAPK14,AKT1,PRKCA,PTGS2,BCL2,MAPK8,MAPK9,MAPK3	PLA2G10,PLA2G6,CASP3
IL-10 Signaling	0.0001549	NONE	JUN,MAP3K7,NFKB1,MAPK14,CHUK,JAK1,SP1,RELA,FOS,STAT3,IL4R,MAPK8,TRAF6	
GDNF Family Ligand-Receptor Interactions	0.0001549	NONE	JUN,DOK1,CDC42,IRS2,IRS1,PIK3R1,PLCG2,FOS,PLCG1,MAPK8,RASA1,MAPK9,MAPK3	
Reelin Signaling in Neurons	0.000195	NONE	GSK3B,MAP2K7,SRC,AKT1,ARHGEF12,MAPK8IP2,APP,PIK3R1,CDK5,FYN,MAPK8IP1,ARHGEF1,MAPK8,MAPK9	
Role of NANOG in Mammalian Embryonic Stem Cell Pluripotency	0.0002344	AKT3	GSK3B,JAK2,AKT1,CTNNB1,WNT7B,JAK3,JAK1,PIK3R1,TP53,APC,SMAD9,STAT3,TCL1A,SMAD1,MAPK3	FZD3
ERK5 Signaling	0.0002951	NONE	FOS,YWHAQ,EGFR,SRC,AKT1,YWHAG,EP300,CREBBP,YWHAZ,PRKCZ,YWHAB	RPS6KA3

Role of JAK1 and JAK3 in Î³c Cytokine Signaling	0.0002951	FES	SYK,STAT3,IRS2,JAK2,PTK2B,JAK3,IL4R,JAK1,IRS1,PIK3R1,MAPK3	
Ephrin B Signaling	0.0003162	NONE	CDC42,EFNB1,EPHB6,GNB2L1,GNB5,CTNNB1,NCK2,GNG10,GNB1,RGS3,VAV2,MAPK3	GNAS
Rac Signaling	0.0003467	PAK6	JUN,MAP2K7,NFKB1,CDC42,PIK3R1,ARPC1B,RELA,BAIAP2,PTK2B,MAPK8,PAK4,PRKCZ,MAPK3	ARPC3,ABI2
Germ Cell-Sertoli Cell Junction Signaling	0.0003631	PAK6	MAP2K7,MAP3K7,MAPK14,CDC42,SRC,AKT1,CTNNB1,JUP,ACTN1,PIK3R1,RHOJ,PDPK1,TUBA1B,BCAR1,MAPK8,PAK4,KEAP1,MAPK9,MAPK3	TUBA1C
Melanocyte Development and Pigmentation Signaling	0.0003715	NONE	SRC,BCL2,EP300,CREBBP,PLCG1,PIK3R1,PRKACA,MAPK3,PLCG2	RPS6KA3,MITF,GNAS,PRKAR1B,PRKAG1
Role of Pattern Recognition Receptors in Recognition of Bacteria and Viruses	0.0003981	PRKD1	PRKCE,NFKB1,PIK3R1,RELA,PLCG2,PRKCD,SYK,PRKCA,PRKCB,MAPK8,PRKCH,PRKCZ,MAPK9,PRKCG,TRAF6,MAPK3	CSF2
Clathrin-mediated Endocytosis Signaling	0.0004074	SNAP91	CDC42,FGF12,SRC,CSNK2B,SNX9,UBD,AP2B1,HGS,PIK3R1,INS,ARPC1B,DNM2,AP2A2,MDM2,EPS15,UBC	ARPC3,PCYOX1,PDGFB,RAB5C,STAM,APOL1
4-1BB Signaling in T Lymphocytes	0.0004266	NONE	JUN,MAPK14,NFKB1,CHUK,MAPK8,MAPK9,RELA,MAPK3	
Docosahexaenoic Acid (DHA) Signaling	0.0004365	AKT3	GSK3B,PDPK1,AKT1,BCL2,APP,PIK3R1	CASP3,GSK3A
Role of PKR in Interferon Induction and Antiviral Response	0.0005248	NONE	MAP3K7,MAPK14,NFKB1,CHUK,AKT1,TP53,TRAF6,RELA	CASP3
Neurotrophin/TRK Signaling	0.0005248	NONE	FOS,JUN,MAP2K7,CDC42,PDPK1,AKT1,EP300,CREBBP,PLCG1,MAPK8,PIK3R1,MAPK3	
iCOS-iCOSL Signaling in T Helper Cells	0.0005248	NFATC3,AKT3	CAMK2B,NFKB1,CHUK,CALM1 (includes others),AKT1,PIK3R1,CAMK2A,RELA,PDPK1,HLA-DMB,PLCG1	PTEN,LCP2,ZAP70
FAK Signaling	0.0005248	AKT3,PAK6	EGFR,SRC,AKT1,PIK3R1,PLCG2,FYN,PDPK1,BCAR1,PLCG1,PAK4,MAPK3	PTEN
TNFR1 Signaling	0.0005495	PAK6	FOS,JUN,CDC42,NFKB1,CHUK,MAPK8,PAK4,RELA	CASP3
CTLA4 Signaling in Cytotoxic T Lymphocytes	0.0005754	AKT3	SYK,FYN,AKT1,JAK2,AP2A2,AP2B1,PLCG1,PIK3R1,AP1M1,PPP2CA	LCP2,PPP2R1A,ZAP70
CDK5 Signaling	0.0006026	NONE	ABL1,PPP1CC,MAPK14,PPP1R14A,EGR1,MAPK8,CDK5,PRKACA,MAPK9,PPP2CA,MAPK3	GNAS,PPP2R1A,PRKAR1B,PRKAG1
Role of JAK family kinases in IL-6-type Cytokine Signaling	0.0006026	NONE	MAPK14,STAT3,JAK2,JAK1,MAPK8,MAPK9,MAPK3	
Type I Diabetes Mellitus Signaling	0.0006166	NONE	MAP2K7,MAP3K7,NFKB1,MAPK14,CHUK,JAK2,JAK1,PIAS1,INS,RELA,BCL2,HLA-DMB,MAPK8,MAPK9,TRAF6	CASP3
Tight Junction Signaling	0.0006166	AKT3	JUN,NFKB1,CDC42,CASK,AKT1,CTNNB1,CLDN19,RELA,FOS,INADL,PRKACA,PRKCZ,PPP2CA,SPTAN1	PTEN,CLDN18,MYL9,PPP2R1A,PRKAR1B,PRKAG1
FcÎ³RIIB Signaling in B Lymphocytes	0.0006166	NONE	DOK1,SYK,PDPK1,AKT1,BTK,MAPK8,PIK3R1,MAPK9,PLCG2	
Ceramide Signaling	0.0007413	AKT3	FOS,JUN,NFKB1,AKT1,BCL2,MAPK8,PIK3R1,PRKCZ,RELA,PPP2CA,MAPK3	PPP2R1A
DNA damage-induced 14-3-3Ïƒ Signaling	0.0007943	AKT3	BRCA1,AKT1,CCNE1,TP53,CDK1	
IL-9 Signaling	0.0007943	NONE	STAT3,NFKB1,IRS2,JAK3,JAK1,IRS1,PIK3R1,RELA	

CD27 Signaling in Lymphocytes	0.000871	NONE	FOS,JUN,MAP2K7,MAP3K7,NFKB1,CHUK,MAPK8,MAPK9,RELA	CASP3
Endometrial Cancer Signaling	0.000871	AKT3	GSK3B,PDPK1,AKT1,CTNNB1,CCND1,PIK3R1,TP53,MAPK3	PTEN
RhoGDI Signaling	0.000955	PAK6	CDC42,SRC,GNB2L1,GNG10,ARHGEF12,EP300,GNB1,RHOJ,ARPC1B,PRKCA,GNB5,ARHGEF1,CREBBP,GRIP1,PAK4,ARHGAP12,ESR1	ARPC3,MYL9,GNAS
IL-15 Production	0.000955	NONE	NFKB1,JAK2,PTK2B,JAK3,JAK1,PRKCZ,RELA	
Role of IL-17F in Allergic Inflammatory Airway Diseases	0.0010233	NONE	NFKB1,EP300,CREBBP,TRAF6,RELA,MAPK3	CSF2,RPS6KA3,CCL4
STAT3 Pathway	0.0010715	NONE	IGF1R,MAPK14,STAT3,EGFR,SRC,JAK2,BCL2,CDKN1A,CDC25A,MAPK8,MAPK9,MAPK3	
FLT3 Signaling in Hematopoietic Progenitor Cells	0.0012023	AKT3	MAPK14,STAT3,MTOR,PDPK1,AKT1,EP300,CREBBP,PIK3R1,EIF4EBP1,MAPK3	RPS6KA3
Dendritic Cell Maturation	0.0014791	AKT3,PLCH2	NFKB1,MAPK14,CHUK,JAK2,AKT1,EP300,PIK3R1,RELA,PLCG2,HLA-DMB,CREBBP,PLCG1,MAPK8,MAPK9,TRAF6,MAPK3	CSF2,PDIA3,FSCN1
IL-4 Signaling	0.0015488	NFATC3,AKT3	MTOR,AKT1,JAK2,HLA-DMB,JAK3,IL4R,JAK1,IRS1,PIK3R1	INPP5K
Amyotrophic Lateral Sclerosis Signaling	0.0016218	AKT3,GRIN2A,CACNA1C	GRIN3B,BCL2,PIK3R1,TP53,GRIN1,NEFL,GRIN3A	SSR4,RAB5C,GRIA2,CASP3
GÎ±s Signaling	0.0016218	NONE	SRC,GNB2L1,GNB5,GNG10,EP300,GNB1,CREBBP,PRKACA,GLP1R,RYR2,MAPK3	HCAR3,GNAS,PRKAR1B,PRKAG1
Prostanoid Biosynthesis	0.0017378	PTGIS	PTGS2,PTGES3,PTGS1	
Nur77 Signaling in T Lymphocytes	0.0017783	NONE	SIN3B,HDAC2,CALM1 (includes others),NR4A1,BCL2,HLA-DMB,EP300,SIN3A,HDAC1	CASP3
Remodeling of Epithelial Adherens Junctions	0.0019953	NONE	SRC,CTNNB1,DNM2,TUBA1B,HGS,ACTN1,ARPC1B,APC	RAB5C,ARPC3,TUBA1C
VEGF Signaling	0.0023988	AKT3	SRC,AKT1,ACTN1,PIK3R1,PLCG2,EIF1AY,PRKCA,PTK2B,BCL2,PRKCB,PLCG1,MAPK3	
PEDF Signaling	0.002884	AKT3,TCF4	MAPK14,NFKB1,CHUK,AKT1,BCL2,PIK3R1,TP53,RELA,MAPK3	
Estrogen-mediated S-phase Entry	0.002884	NONE	CCNE1,CCND1,CDKN1A,CDC25A,ESR1,CDK1	
p38 MAPK Signaling	0.0032359	NONE	HSPB1,MAP3K7,HIST3H3,MAPK14,MAPKAPK5,HSPB7,EP300,CREBBP,HIST2H3C,MAX,TP53,TRAF6	PLA2G10,RPS6KA3,PLA2G6
SAPK/JNK Signaling	0.0032359	NFATC3	JUN,MAP2K7,MAP3K7,CDC42,GNB1,MAPK8IP2,IRS1,PIK3R1,TP53,MAPK8IP1,MAPK8,MAPK9	
Cdc42 Signaling	0.0035481	NONE	JUN,GSK3B,MAPK14,CDC42,SRC,ARPC1B,APC,VAV2,FOS,BAIAP2,HLA-DMB,MAPK8,RASA1,PAK4,PRKCZ,MAPK9	ARPC3,CDC42SE1,MYL9
FGF Signaling	0.0038905	AKT3	MAPK14,FGF12,STAT3,AKT1,PRKCA,EP300,CREBBP,PLCG1,MAPK8,PIK3R1,MAPK3	
Toll-like Receptor Signaling	0.0038905	NONE	FOS,JUN,MAP3K7,MAPK14,NFKB1,CHUK,UBD,MAPK8,UBC,TRAF6,RELA	
Oncostatin M Signaling	0.0038905	NONE	MT2A,STAT3,JAK2,JAK3,JAK1,EPAS1,MAPK3	

Human Embryonic Stem Cell Pluripotency	0.0046774	AKT3,TCF4	GSK3B,PDPK1,TCF3,AKT1,CTNNB1,SMAD2,WNT7B,SMAD1,PIK3R1,APC	GSK3A,PDGFB,GNAS,FZD3
Cell Cycle Control of Chromosomal Replication	0.0052481	NONE	CDK7,MCM7,CDK5,CDK6,ORC4	RPA3
Spliceosomal Cycle	0.0054954	NONE	LOC102724594/U2AF1,U2AF2	
PAK Signaling	0.0057544	PAK6	CDC42,PTK2B,NCK2,MAPK8,PAK4,PIK3R1,MAPK9,MAPK3	CASP3,MYL9,PDGFB
Death Receptor Signaling	0.0074131	NONE	HSPB1,MAP2K7,NFKB1,PARP1,CHUK,HSPB7,BCL2,MAPK8,ACIN1,RELA,SPTAN1	CASP3
cAMP-mediated signaling	0.0074131	GRM3	CAMK2B,AKAP13,TDP2,SRC,CALM1 (includes others),EP300,CAMK1D,GLP1R,CAMK2A,STAT3,PDE7B,CREBBP,PRKACA,APEX1,PTGER3,MAPK3	RGS7,HCAR3,CREM,GNAS,PRKAR1B
TNFR2 Signaling	0.0075858	NONE	FOS,JUN,NFKB1,CHUK,MAPK8,RELA	
Role of p14/p19ARF in Tumor Suppression	0.0089125	NONE	NPM2,UBTF,MDM2,PIK3R1,TP53,TTF1	
Induction of Apoptosis by HIV1	0.0089125	NONE	MAP2K7,NFKB1,CHUK,BCL2,MAPK8,TP53,MAPK9,RELA	CASP3

Historic Gene Interactome Pathways

Ingenuity Canonical Pathways	B-H P-value	Molecules
PI3K/AKT Signaling	1.58E-36	GSK3B,YWHAQ,PTEN,AKT1,PPP2R4,CDC37,GSK3A,INPPL1,PIK3R1,ILK,NFKBIA,YWHAE,MAPK8IP1,MAP3K8,INPP5K,TSC2,CDKN1A,GRB2,PPP2R1A,NOS3,TSC1,PRKCZ,HSP90AA1,EIF4EBP1,FOXO1,RPS6KB1,PPP2CA,MAP3K5,MTOR,CHUK,JAK2,YWHAG,CTNNB1,RAF1,IKBKB,AKT2,BAD,THEM4,PDPK1,GAB2,CDKN1B,BCL2,RHEB,MDM2,BCL2L1,HSP90AB1,YWHAZ,FOXO3
Molecular Mechanisms of Cancer	1.00E-28	E2F6,CDC42,GNA11,ADCY6,PRKCQ,SMAD4,NFKBIA,GNAI3,GNAQ,SMAD3,XIAP,LRP1,TGFBR1,GNAZ,SRC,JAK2,CTNNB1,RAF1,SMAD2,BAD,BRAF,PAK6,CDKN1B,CASP3,BCL2L1,GSK3B,GNAI1,MAPK14,AKT1,BCL2L11,GNAO1,IRS1,GSK3A,PIK3R1,BMPR1B,ADCY10,CCND3,DIRAS3,GNAI2,FYN,SMAD7,CDKN1A,GRB2,CREBBP,PRKCZ,PRKDC,FOXO1,PSEN1,PLCB1,BRCA1,MAP3K5,NOTCH1,PAK1,RBPJ,EP300,CASP9,ATP,ZBTB17,AKT2,GAB2,MAP2K4,PSEN2,BCL2,MDM2,RAC1,CHEK1
PTEN Signaling	6.31E-26	GSK3B,FGFR1,CDC42,PTEN,AKT1,BCL2L11,FOXO4,GSK3A,INPPL1,PIK3R1,ILK,BMPR1B,INPP5K,CDKN1A,GRB2,NTRK3,TGFBR1,NTRK2,PRKCZ,FOXO1,RPS6KB1,EGFR,CHUK,CSNK2B,RAF1,CASP9,IKBKB,CSNK2A1,AKT2,BAD,FOXG1,PDPK1,CDKN1B,BCL2,CASP3,BCL2L1,FOXO3,RAC1
14-3-3-mediated Signaling	1.58E-21	GSK3B,TRAF2,YWHAQ,AKT1,TNFRSF1A,PRKCQ,YAP1,GSK3A,PIK3R1,YWHAE,TUBB2A,VIM,SNCA,TNF,TSC2,GRB2,MAPT,TSC1,PRKCZ,FOXO1,PLCB1,AKT1S1,MAP3K5,SRC,YWHAG,TUBB,RAF1,AKT2,BAD,MAP2K4,CDKN1B,TP73,PLCG1,YWHAZ
Neuregulin Signaling	2.51E-18	PTEN,AKT1,ERBB4,PICK1,PRKCQ,PIK3R1,ADAM17,ERBB2,GRB2,DCN,PRKCZ,HSP90AA1,RPS6KB1,PSEN1,EGFR,MTOR,SRC,RAF1,NRG1,AKT2,BAD,EGF,PDPK1,DLG4,CDKN1B,PLCG1,HSP90AB1,ERBB3
B Cell Receptor Signaling	3.16E-18	GSK3B,MAPK14,CDC42,PTEN,AKT1,CREB1,CALM1 (includes others),PRKCQ,GSK3A,INPPL1,PIK3R1,NFKBIA,PPP3CC,BCL10,MAP3K8,INPP5K,GRB2,CREBBP,CD79A,FOXO1,RPS6KB1,MAP3K5,MTOR,CHUK,MAP3K11,RAF1,EP300,IKBKB,AKT2,BAD,PDPK1,ATF4,GAB2,MAP2K4,BCL2L1,RAC1,LYN
p70S6K Signaling	7.94E-18	GNAI1,YWHAQ,AKT1,PRKCQ,PPP2R4,IRS1,PIK3R1,YWHAE,EEF2,GNAI3,GNAQ,GNAI2,GRB2,PPP2R1A,MAPT,PRKCZ,CD79A,RPS6KB1,PPP2CA,PLCB1,EGFR,MTOR,SRC,YWHAG,RAF1,AKT2,BAD,PDPK1,PLCG1,YWHAZ,LYN
HER-2 Signaling in Breast Cancer	1.26E-17	GSK3B,CDC42,AKT1,PRKCQ,GSK3A,PIK3R1,TSC2,ERBB2,CDKN1A,GRB2,ITGB3,TSC1,PRKCZ,FOXO1,EGFR,MAP3K5,CASP9,NRG1,AKT2,BAD,EGF,CDKN1B,PLCG1,MDM2,ERBB3
Insulin Receptor Signaling	2.51E-17	GSK3B,GRB10,PTEN,AKT1,PDE3B,FOXO4,IRS1,GSK3A,INPPL1,PIK3R1,FYN,INPP5K,TSC2,GRB2,SH2B2,TSC1,PRKCZ,NCK1,EIF4EBP1,FOXO1,RPS6KB1,PPP1R11,MTOR,JAK2,RAF1,PPP1CA,ATP,AKT2,BAD,PTPN1,PDPK1,FOXO3
Prostate Cancer Signaling	2.51E-17	GSK3B,PTEN,AKT1,CREB1,PIK3R1,NFKBIA,CDKN1A,GRB2,CREBBP,HSP90AA1,FOXO1,MTOR,CHUK,CTNNB1,RAF1,EP300,CASP9,AKT2,BAD,AR,PDPK1,ATF4,CDKN1B,BCL2,MDM2,HSP90AB1
GNRH Signaling	6.31E-17	GNAI1,MAPK14,CDC42,GNA11,CREB1,ADCY6,PRKCQ,GNAI3,ADCY10,GNAQ,GNAI2,MAP3K8,GRB2,CREBBP,ITPR1,PRKCZ,PLCB1,EGFR,MAP3K5,SRC,MAP3K11,RAF1,PAK1,EP300,ATP,EGF,ATF4,ITPR3,MAP2K4,PAK6,RAC1
Pancreatic Adenocarcinoma Signaling	7.94E-17	E2F6,CDC42,AKT1,PIK3R1,SMAD4,STAT1,SMAD3,ERBB2,CDKN1A,GRB2,TGFBR1,EGFR,JAK2,NOTCH1,VEGFA,RAF1,SMAD2,CASP9,HMOX1,AKT2,BAD,EGF,MAP2K4,CDKN1B,BCL2,MDM2,BCL2L1,RAC1

Glucocorticoid Receptor Signaling	1.58E-16	KAT2B,POU2F1,NCOR2,TRAF2,MAPK14,AKT1,CREB1,IFNG,PIK3R1,SMAD4,NFKBIA,SMARCC1,PPP3CC,SMARCB1,A2M,STAT1,TNF,SMAD3,CDKN1A,CDKN1C,GRB2,CREBBP,TGFBR1,HSP90AA1,ESR1,CD3E,CHUK,JAK2,CD3G,RAF1,SMAD2,EP300,IKBKB,SMARCE1,AKT2,AR,MAP2K4,BCL2,BCL2L1,HSP90AB1,FOXO3,RAC1,PRKAB2
NF-Î°B Signaling	2.00E-16	GSK3B,FGFR1,TRAF2,TDP2,AKT1,TNFRSF1A,PRKCQ,PIK3R1,NFKBIA,BMPR1B,BCL10,TNF,MAP3K8,NTRK3,CREBBP,TGFBR1,NTRK2,PRKCZ,RIPK1,IRAK1,EGFR,CHUK,TNFSF11,CSNK2B,TNFRSF1B,RAF1,EP300,IKBKB,TRADD,CSNK2A1,AKT2,EGF,BRAF,TNFAIP3
Production of Nitric Oxide and Reactive Oxygen Species in Macrophages	1.58E-15	MAPK14,AKT1,IFNG,TNFRSF1A,PRKCQ,PPP2R4,PIK3R1,APOC1,NFKBIA,DIRAS3,STAT1,TNF,MAP3K8,PPP2R1A,CREBBP,APOC4,PRKCZ,PPP2CA,PPP1R11,ALB,MAP3K5,CHUK,JAK2,MAP3K11,TNFRSF1B,PPP1CA,IKBKB,AKT2,APOC2,MAP2K4,CLU,PLCG1,APOE,RAC1
Role of NFAT in Regulation of the Immune Response	2.51E-15	GSK3B,GNAI1,GNA11,AKT1,CALM1 (includes others),GNAO1,PRKCQ,GNB1,GSK3A,PIK3R1,NFKBIA,PPP3CC,GNAI3,MS4A2,GNAQ,GNAI2,FYN,GRB2,ITPR1,CD79A,PLCB1,CD3E,GNAZ,CHUK,GNB2L1,CD3G,RAF1,IKBKB,AKT2,CABIN1,ITPR3,PLCG1,LYN
ErbB Signaling	2.51E-15	GSK3B,MAPK14,CDC42,EGFR,MTOR,AKT1,ERBB4,RAF1,PRKCQ,PAK1,NRG1,PIK3R1,EGF,PDPK1,MAP2K4,PAK6,ERBB2,GRB2,PLCG1,ERBB3,PRKCZ,NCK1,FOXO1,RPS6KB1
G-Protein Coupled Receptor Signaling	2.51E-15	GNAI1,TDP2,ADORA2A,GNA11,AKT1,CREB1,ADCY6,GNAO1,PDE3B,GABBR1,PIK3R1,PDE4B,NFKBIA,GNAI3,ADCY10,GRM3,GNAQ,DRD2,GNAI2,FYN,MAP3K8,RGS2,GRB2,CREBBP,DRD3,HTR2A,S1PR1,PLCB1,CHUK,SRC,RAF1,EP300,IKBKB,ATP,AKT2,PDPK1,BRAF,ATF4,DRD4,RGS4
ILK Signaling	1.26E-14	GSK3B,CDC42,PTEN,AKT1,CREB1,TNFRSF1A,PPP2R4,RICTOR,IRS1,GSK3A,PIK3R1,ILK,VIM,DIRAS3,ACTG1,TNF,ACTN2,PPP2R1A,ITGB3,CREBBP,SH2B2,PPP2CA,MTOR,VEGFA,FLNA,CTNNB1,EP300,AKT2,PDPK1,ATF4,MAP2K4,TGFB1I1,CASP3
Role of Tissue Factor in Cancer	1.58E-14	HCK,MAPK14,CDC42,GNA11,PTEN,AKT1,LIMK1,PIK3R1,GNAQ,FYN,ITGB3,RPS6KB1,PLCB1,EGFR,MTOR,SRC,JAK2,VEGFA,RPS6KA3,PAK1,AKT2,CSF1,CASP3,BCL2L1,RAC1,LYN
Ephrin Receptor Signaling	1.58E-14	GNAI1,CDC42,GNA11,AKT1,CREB1,GNAO1,LIMK1,GNB1,GNAI3,GNAQ,GNAI2,FYN,GRB2,CREBBP,SDCBP,NCK1,KALRN,GNAZ,SRC,ITSN1,JAK2,GNB2L1,VEGFA,RAF1,PAK1,SDC2,EP300,AKT2,EGF,ATF4,PAK6,RAC1
Protein Kinase A Signaling	1.58E-14	GSK3B,GNAI1,YWHAQ,TDP2,PTEN,CREB1,CALM1 (includes others),ADCY6,PDE3B,PRKCQ,GNB1,GSK3A,SMAD4,PDE4B,NFKBIA,YWHAE,PPP3CC,GNAI3,ADCY10,GNAQ,GNAI2,SMAD3,CREBBP,ITPR1,TGFBR1,NOS3,PRKCZ,AKAP6,PPP1R11,CDC27,PLCB1,CHUK,YWHAG,GNB2L1,FLNA,CTNNB1,RAF1,EP300,PPP1CA,ATP,BAD,PTPN1,BRAF,ATF4,ITPR3,PLCG1,YWHAZ,AKAP9
Cardiac Hypertrophy Signaling	3.16E-14	GSK3B,GNAI1,MAPK14,GNA11,AKT1,CREB1,CALM1 (includes others),ADCY6,GNAO1,GNB1,IRS1,PIK3R1,PPP3CC,MAPKAPK2,GNAI3,ADCY10,DIRAS3,GNAQ,GNAI2,MAP3K8,GRB2,CREBBP,TGFBR1,RPS6KB1,PLCB1,GNAZ,MAP3K5,MTOR,MAP3K11,GNB2L1,RAF1,EP300,ATP,HSPB1,MAP2K4,PLCG1
G Beta Gamma Signaling	3.98E-14	GNAI1,GNAZ,CDC42,EGFR,SRC,GNA11,AKT1,GNB2L1,GNAO1,RAF1,PRKCQ,PAK1,GNB1,ATP,AKT2,GNAI3,KCNJ6,KCNJ9,GNAQ,GNAI2,PDPK1,GRB2,PLCG1,PRKCZ
RAR Activation	3.98E-14	KAT2B,SORBS3,NCOR2,MAPK14,PTEN,AKT1,RARA,ADCY6,PRKCQ,PIK3R1,SMAD4,CRABP2,SMARCC1,MAPKAPK2,ADCY10,SMARCB1,SMAD7,SMAD3,CREBBP,PRKCZ,MAP3K5,SRC,JAK2,VEGFA,CSNK2B,SMAD2,EP300,SMARCE1,CSNK2A1,AKT2,PDPK1,MAP2K4,RAC1

Thrombin Signaling	3.98E-14	GNAI1,GATA2,MAPK14,GNA11,AKT1,CREB1,ADCY6,GNAO1,PRKCQ,GNB1,PIK3R1,GNAI3,ADCY10,DIRAS3,GNAQ,GNAI2,GRB2,ITPR1,PRKCZ,RPS6KB1,PLCB1,GNAZ,EGFR,SRC,GNB2L1,RAF1,IKBKB,GATA1,AKT2,EGF,PDPK1,ITPR3,PLCG1
Huntington's Disease Signaling	5.01E-14	NCOR2,GNA11,AKT1,CREB1,PRKCQ,GNB1,PIK3R1,DCTN1,PSME3,GNAQ,SNCA,HTT,GRB2,CREBBP,ITPR1,PRKCZ,BDNF,PLCB1,EGFR,MTOR,DNAJB1,GNB2L1,EP300,CASP9,CASP1,NSF,AKT2,EGF,PDPK1,ARFIP2,DLG4,ATF4,MAP2K4,CASP3,BCL2L1,STX1A
ERK5 Signaling	5.01E-14	YWHAQ,EGFR,SRC,YWHAG,AKT1,CREB1,RPS6KA3,EP300,WNK1,YWHAE,BAD,EGF,GNAQ,ATF4,MAP3K8,CREBBP,YWHAZ,PRKCZ,FOXO3,RPS6KB1
Dopamine-DARPP32 Feedback in cAMP Signaling	5.01E-14	GNAI1,CREB1,CALM1 (includes others),ADCY6,PRKCQ,PPP2R4,PPP3CC,GNAI3,KCNJ6,ADCY10,KCNJ9,GNAQ,DRD2,GNAI2,PPP2R1A,CREBBP,ITPR1,PRKCZ,PPP2CA,DRD3,PPP1R11,PLCB1,EP300,PPP1CA,ATP,CAMKK1,PAWR,ATF4,ITPR3,DRD4,PLCG1
Colorectal Cancer Metastasis Signaling	7.94E-14	GSK3B,AKT1,IFNG,TNFRSF1A,ADCY6,GNB1,PIK3R1,SMAD4,ADCY10,DIRAS3,STAT1,TNF,SMAD3,GRB2,LRP1,TGFBR1,EGFR,SRC,JAK2,GNB2L1,VEGFA,CTNNB1,SMAD2,CASP9,ATP,MMP17,AKT2,BAD,EGF,APPL1,BRAF,MAP2K4,CASP3,BCL2L1,RAC1,MMP23B
PI3K Signaling in B Lymphocytes	1.00E-13	PTEN,AKT1,CREB1,CALM1 (includes others),IRS1,PIK3R1,NFKBIA,PPP3CC,FYN,BCL10,ITPR1,ATF5,SH2B2,PRKCZ,CD79A,PLCB1,CHUK,RAF1,IKBKB,AKT2,PDPK1,ATF4,ITPR3,PLCG1,FOXO3,RAC1,LYN
Axonal Guidance Signaling	1.26E-13	CDC42,GNA11,SRGAP3,PRKCQ,LIMK1,GNB1,GNAI3,GNAQ,ERBB2,SDCBP,NCK1,BDNF,ADAM9,GNAZ,VEGFA,RAF1,ADAM19,DPYSL2,EGF,PLXNA1,PAK6,GSK3B,GNAI1,AKT1,GNAO1,PIK3R1,ACE,PPP3CC,TUBB2A,ADAM17,GNAI2,FYN,GRB2,NTRK3,NTRK2,PRKCZ,KALRN,PLCB1,ITSN1,GNB2L1,TUBB,PAK1,SDC2,NTF3,RTN4R,AKT2,PLCG1,SRGAP2,RAC1
Role of Macrophages, Fibroblasts and Endothelial Cells in Rheumatoid Arthritis	1.58E-13	GSK3B,TRAF2,MAPK14,AKT1,CREB1,CALM1 (includes others),TNFRSF1A,GNAO1,PRKCQ,PIK3R1,NFKBIA,PPP3CC,MAPKAPK2,GNAQ,TNF,CREBBP,LRP1,PRKCZ,RIPK1,PLCB1,IRAK1,CEBPD,CHUK,SRC,JAK2,TNFSF11,VEGFA,CTNNB1,TNFRSF1B,RAF1,EP300,IKBKB,TRADD,AKT2,ATF4,MAP2K4,CSF1,PLCG1,RAC1,DKK4
Breast Cancer Regulation by Stathmin1	2.00E-13	E2F6,GNAI1,CDC42,CALM1 (includes others),ADCY6,PRKCQ,PPP2R4,LIMK1,GNB1,PIK3R1,GNAI3,ADCY10,TUBB2A,GNAQ,GNAI2,CDKN1A,GRB2,PPP2R1A,ITPR1,PRKCZ,PPP2CA,PPP1R11,PLCB1,GNB2L1,TUBB,RAF1,PAK1,PPP1CA,ATP,ITPR3,CDKN1B,RAC1
Sertoli Cell-Sertoli Cell Junction Signaling	2.51E-13	GSK3B,F11R,MAPK14,CDC42,PTEN,EPN2,AKT1,TNFRSF1A,GSK3A,ILK,ADCY10,SPTBN1,TUBB2A,A2M,EPB41,ACTG1,TNF,MAP3K8,ACTN2,NOS3,MAP3K5,SRC,MAP3K11,TUBB,CTNNB1,RAF1,ATP,AKT2,MAP2K4,RAC1,SPTAN1
Glioblastoma Multiforme Signaling	3.16E-13	E2F6,GSK3B,CDC42,PTEN,AKT1,PIK3R1,DIRAS3,TSC2,CDKN1A,GRB2,ITPR1,TSC1,FOXO1,RPS6KB1,PLCB1,EGFR,MTOR,SRC,NF2,CTNNB1,RAF1,AKT2,EGF,ITPR3,CDKN1B,PLCG1,MDM2,RAC1
p53 Signaling	3.16E-13	KAT2B,GSK3B,BRCA1,MAPK14,PTEN,AKT1,CTNNB1,EP300,ST13,PIK3R1,PIAS1,AKT2,TOPBP1,TP53BP2,BCL2,CDKN1A,TP73,MDM2,BCL2L1,PRKDC,SIRT1,CHEK1,MDM4
IL-8 Signaling	3.16E-13	GNAI1,AKT1,PRKCQ,LIMK1,GNB1,PIK3R1,GNAI3,CCND3,DIRAS3,GNAI2,ITGB3,PRKCZ,EIF4EBP1,RPS6KB1,IRAK1,EGFR,MTOR,CHUK,SRC,GNB2L1,VEGFA,RAF1,IKBKB,HMOX1,AKT2,EGF,BRAF,MAP2K4,BCL2,BCL2L1,RAC1
Corticotropin Releasing Hormone Signaling	5.01E-13	GNAI1,MAPK14,CREB1,CALM1 (includes others),ADCY6,GNAO1,PRKCQ,GNAI3,ADCY10,GNAQ,GNAI2,CREBBP,ITPR1,NOS3,PRKCZ,BDNF,VEGFA,RAF1,NR4A1,EP300,ATP,BRAF,ATF4,ITPR3,PLCG1

CREB Signaling in Neurons	5.01E-13	GNAI1,GNA11,AKT1,CREB1,CALM1 (includes others),ADCY6,GNAO1,PRKCQ,GNB1,PIK3R1,GNAI3,ADCY10,GRM3,GNAQ,GNAI2,GRIA2,GRB2,CREBBP,ITPR1,PRKCZ,PLCB1,GNAZ,GNB2L1,RAF1,EP300,ATP,AKT2,ATF4,ITPR3,PLCG1
CXCR4 Signaling	1.26E-12	GNAI1,GNA11,AKT1,ADCY6,GNAO1,PRKCQ,GNB1,PIK3R1,GNAI3,ADCY10,DIRAS3,GNAQ,GNAI2,ITPR1,PRKCZ,PLCB1,GNAZ,SRC,GNB2L1,RAF1,PAK1,ATP,AKT2,ITPR3,MAP2K4,PAK6,RAC1,LYN
EGF Signaling	2.00E-12	MAPK14,EGFR,SRC,MTOR,AKT1,CSNK2B,RAF1,PIK3R1,AKT2,CSNK2A1,EGF,ITPR3,MAP2K4,STAT1,GRB2,PLCG1,ITPR1,RPS6KB1
HIPPO signaling	2.00E-12	STK3,YWHAQ,YWHAG,NF2,SMAD2,PPP2R4,PPP1CA,YAP1,SMAD4,YWHAE,CD44,DLG4,SMAD3,TP53BP2,SKP2,PPP2R1A,YWHAZ,PRKCZ,STK4,PPP2CA,PPP1R11
IGF-1 Signaling	3.16E-12	GRB10,YWHAQ,YWHAG,JAK2,AKT1,CSNK2B,RAF1,CASP9,IRS1,PIK3R1,YWHAE,AKT2,CSNK2A1,BAD,NEDD4,PDPK1,GRB2,YWHAZ,PRKCZ,FOXO1,FOXO3,RPS6KB1
Gap Junction Signaling	3.98E-12	GNAI1,AKT1,ADCY6,PRKCQ,PIK3R1,PPP3CC,GNAI3,ADCY10,TUBB2A,GNAQ,DRD2,GNAI2,ACTG1,GRB2,ITPR1,PRKCZ,HTR2A,PLCB1,EGFR,SRC,TUBB,CTNNB1,RAF1,ATP,AKT2,EGF,ITPR3,PLCG1
Type I Diabetes Mellitus Signaling	3.98E-12	CD3E,IRAK1,TRAF2,MAPK14,MAP3K5,CHUK,IFNG,JAK2,TNFRSF1A,CD3G,TNFRSF1B,CASP9,IKBKB,PIAS1,TRADD,NFKBIA,MAP2K4,STAT1,TNF,BCL2,CPE,CASP3,RIPK1
Acute Phase Response Signaling	6.31E-12	TRAF2,MAPK14,AKT1,TNFRSF1A,APOH,PIK3R1,CRABP2,NFKBIA,A2M,TNF,GRB2,ECSIT,RIPK1,ALB,IRAK1,MAP3K5,MTOR,CHUK,JAK2,TNFRSF1B,RAF1,IKBKB,TRADD,HMOX1,AKT2,OSMR,PDPK1,MAP2K4
NGF Signaling	6.31E-12	CDC42,MAP3K5,CHUK,AKT1,CREB1,MAP3K11,RAF1,RPS6KA3,EP300,IKBKB,PIK3R1,AKT2,PDPK1,ATF4,MAP2K4,MAP3K8,TRIO,GRB2,CREBBP,PLCG1,PRKCZ,RAC1,RPS6KB1
Role of Osteoblasts, Osteoclasts and Chondrocytes in Rheumatoid Arthritis	6.31E-12	GSK3B,TRAF2,MAPK14,AKT1,IFNG,CALM1 (includes others),TNFRSF1A,PIK3R1,SMAD4,NFKBIA,PPP3CC,ADAM17,TNF,ITGB3,XIAP,LRP1,FOXO1,MAP3K5,CHUK,SRC,TNFSF11,CTNNB1,TNFRSF1B,CASP9,IKBKB,TRADD,AKT2,BAD,MAP2K4,BCL2,CSF1,DKK4
Androgen Signaling	6.31E-12	GNAI1,KAT2B,GNAZ,SRC,GNA11,CALM1 (includes others),DNAJB1,GNB2L1,GNAO1,PRKCQ,EP300,GNB1,NCOA4,GNAI3,CALR,AR,GNAQ,GNAI2,TGFB1I1,SMAD3,CREBBP,PRKCZ,HSP90AA1
Role of NFAT in Cardiac Hypertrophy	7.94E-12	GSK3B,GNAI1,MAPK14,AKT1,CALM1 (includes others),ADCY6,PRKCQ,GNB1,PIK3R1,PPP3CC,GNAI3,ADCY10,GNAQ,GNAI2,GRB2,ITPR1,TGFBR1,PRKCZ,PLCB1,SRC,GNB2L1,RAF1,EP300,ATP,AKT2,CABIN1,ITPR3,MAP2K4,PLCG1
TNFR1 Signaling	7.94E-12	TRAF2,CDC42,CHUK,TNFRSF1A,PAK1,CASP9,IKBKB,TRADD,NFKBIA,TNFAIP3,MAP2K4,PAK6,TNF,CASP3,XIAP,RIPK1
PPARÎ±/RXRÎ± Activation	3.16E-11	NCOR2,MAPK14,GNA11,ADCY6,IRS1,SMAD4,NFKBIA,ADCY10,GNAQ,CYP2C18,SMAD3,GRB2,CREBBP,TGFBR1,HSP90AA1,PLCB1,CHUK,JAK2,RAF1,SMAD2,EP300,IKBKB,ATP,MAP2K4,CYP2C8,PLCG1,HSP90AB1,PRKAB2
Tec Kinase Signaling	5.01E-11	GNAI1,HCK,GNA11,GNAO1,PRKCQ,GNB1,PIK3R1,GNAI3,MS4A2,DIRAS3,GNAQ,GNAI2,FYN,ACTG1,STAT1,TNF,PRKCZ,GNAZ,SRC,JAK2,GNB2L1,PAK1,MAP2K4,PAK6,PLCG1,LYN
Endothelin-1 Signaling	5.01E-11	GNAI1,MAPK14,GNA11,ADCY6,GNAO1,PRKCQ,PIK3R1,GNAI3,ADCY10,GNAQ,GNAI2,GRB2,ITPR1,NOS3,PRKCZ,PLCB1,GNAZ,SRC,RAF1,CASP9,CASP1,ATP,HMOX1,BRAF,ITPR3,LCAT,CASP3,PLCG1

Death Receptor Signaling	5.01E-11	TRAF2,MAP3K5,CHUK,TNFRSF1A,TNFRSF1B,LIMK1,CASP9,IKBKB,TRADD,NFKBIA,TNKS,HSPB1,ACTG1,MAP2K4,TNF,BCL2,CASP3,XIAP,RIPK1,SPTAN1
Germ Cell-Sertoli Cell Junction Signaling	6.31E-11	MAPK14,CDC42,EPN2,AKT1,TNFRSF1A,LIMK1,PIK3R1,ILK,TUBB2A,A2M,DIRAS3,ACTG1,TNF,MAP3K8,ACTN2,TGFBR1,MAP3K5,SRC,MAP3K11,TUBB,CTNNB1,PAK1,PDPK1,MAP2K4,PAK6,RAC1
FLT3 Signaling in Hematopoietic Progenitor Cells	6.31E-11	MAPK14,MTOR,AKT1,CREB1,RAF1,RPS6KA3,EP300,PIK3R1,AKT2,BAD,PDPK1,ATF4,GAB2,STAT1,GRB2,CREBBP,EIF4EBP1,RPS6KB1
GÎ±q Signaling	1.12E-10	GSK3B,GNA11,AKT1,CALM1 (includes others),PRKCQ,GNB1,PIK3R1,NFKBIA,PPP3CC,DIRAS3,GNAQ,RGS2,ITPR1,PRKCZ,HTR2A,PLCB1,CHUK,GNB2L1,RAF1,IKBKB,HMOX1,AKT2,ITPR3,PLCG1,RGS4
cAMP-mediated signaling	1.20E-10	GNAI1,TDP2,ADORA2A,CREB1,CALM1 (includes others),ADCY6,GNAO1,PDE3B,GABBR1,PDE4B,PPP3CC,GNAI3,ADCY10,GRM3,DRD2,GNAI2,RGS2,CREBBP,AKAP6,DRD3,S1PR1,SRC,RAF1,EP300,ATP,BRAF,ATF4,DRD4,RGS4,AKAP9
Relaxin Signaling	1.58E-10	GNAI1,GNAZ,TDP2,GNA11,AKT1,CREB1,GNB2L1,ADCY6,VEGFA,GNAO1,PDE3B,GNB1,ATP,PIK3R1,PDE4B,NFKBIA,AKT2,GNAI3,ADCY10,GNAQ,GNAI2,BRAF,NOS3,PRKCZ
FcÎ³ Receptor-mediated Phagocytosis in Macrophages and Monocytes	1.78E-10	CDC42,HCK,RAB11A,SRC,PTEN,AKT1,PRKCQ,PAK1,PIK3R1,AKT2,HMOX1,FYN,GAB2,ACTG1,PLCG1,PRKCZ,RAC1,NCK1,RPS6KB1,LYN
Apoptosis Signaling	2.00E-10	MAP3K5,CHUK,BCL2L11,TNFRSF1A,TNFRSF1B,RAF1,PRKCQ,CASP9,IKBKB,NFKBIA,BAD,MAP2K4,TNF,BCL2,CASP3,PLCG1,XIAP,BCL2L1,SPTAN1
Induction of Apoptosis by HIV1	2.00E-10	TRAF2,MAP3K5,CHUK,TNFRSF1A,TNFRSF1B,CASP9,IKBKB,TRADD,NFKBIA,MAP2K4,TNF,BCL2,CASP3,XIAP,BCL2L1,RIPK1
Reelin Signaling in Neurons	2.00E-10	GSK3B,HCK,SRC,PAFAH1B1,AKT1,MAP3K11,VLDLR,APP,PIK3R1,LRP8,NDEL1,FYN,MAPK8IP1,MAP2K4,ITGB3,APOE,MAPT,LYN
CD28 Signaling in T Helper Cells	2.09E-10	CD3E,CDC42,CHUK,CALM1 (includes others),AKT1,CD3G,PRKCQ,PAK1,IKBKB,PIK3R1,NFKBIA,AKT2,PPP3CC,FYN,PDPK1,BCL10,ITPR3,MAP2K4,GRB2,PLCG1,ITPR1,RAC1
Regulation of IL-2 Expression in Activated and Anergic T Lymphocytes	3.02E-10	CD3E,CHUK,CALM1 (includes others),CD3G,SMAD2,RAF1,IKBKB,SMAD4,NFKBIA,PPP3CC,FYN,BCL10,MAP2K4,SMAD3,GRB2,PLCG1,TGFBR1,RAC1
Type II Diabetes Mellitus Signaling	3.24E-10	TRAF2,MAP3K5,MTOR,CHUK,AKT1,TNFRSF1A,TNFRSF1B,PRKCQ,IKBKB,IRS1,ATP,SLC27A4,PIK3R1,TRADD,NFKBIA,AKT2,PDPK1,MAP2K4,TNF,SLC27A3,SH2B2,PRKCZ,PRKAB2
ERK/MAPK Signaling	3.47E-10	YWHAQ,CREB1,PPP2R4,PIK3R1,FYN,STAT1,GRB2,PPP2R1A,CREBBP,EIF4EBP1,ESR1,PPP2CA,PPP1R11,SRC,YWHAG,RAF1,PAK1,EP300,PPP1CA,BAD,HSPB1,BRAF,ATF4,PAK6,PLCG1,YWHAZ,RAC1
Neurotrophin/TRK Signaling	3.63E-10	CDC42,MAP3K5,AKT1,CREB1,RAF1,EP300,NTF3,PIK3R1,PDPK1,ATF4,MAP2K4,GRB2,NTRK3,CREBBP,PLCG1,NTRK2,BDNF
RhoGDI Signaling	4.17E-10	GNAI1,CDC42,GNA11,GNAO1,LIMK1,GNB1,GNAI3,DIRAS3,GNAQ,GNAI2,ACTG1,DLC1,CREBBP,GRIP1,ESR2,ESR1,GNAZ,SRC,GNB2L1,PAK1,EP300,ARHGAP1,CD44,RDX,PAK6,RAC1
IL-6 Signaling	4.27E-10	TRAF2,MAPK14,CHUK,JAK2,AKT1,TNFRSF1A,VEGFA,CSNK2B,TNFRSF1B,RAF1,IKBKB,PIK3R1,NFKBIA,AKT2,CSNK2A1,MAPKAPK2,A2M,HSPB1,MAP2K4,TNF,GRB2
IL-1 Signaling	4.68E-10	GNAI1,GNAZ,IRAK1,MAPK14,GNA11,CHUK,GNB2L1,ADCY6,GNAO1,GNB1,IKBKB,ATP,NFKBIA,GNAI3,ADCY10,GNAQ,GNAI2,MAP2K4,ECSIT
P2Y Purigenic Receptor Signaling Pathway	5.01E-10	GNAI1,PLCB1,AKT1,CREB1,GNB2L1,ADCY6,RAF1,PRKCQ,EP300,GNB1,ATP,PIK3R1,AKT2,GNAI3,ADCY10,GNAQ,GNAI2,ATF4,CREBBP,PLCG1,ITGB3,PRKCZ

eNOS Signaling	5.50E-10	CALM1 (includes others),AKT1,ADCY6,VEGFA,PRKCQ,CASP9,ATP,PIK3R1,AKT2,ADCY10,GNAQ,PDPK1,ITPR3,CASP3,PLCG1,ITPR1,ESR2,NOS3,CCNA2,HSP90AB1,PRKCZ,HSP90AA1,ESR1,PRKAB2
Renin-Angiotensin Signaling	5.62E-10	MAPK14,JAK2,ADCY6,RAF1,PRKCQ,PAK1,ATP,PIK3R1,ACE,ADCY10,GNAQ,ITPR3,MAP2K4,PAK6,STAT1,TNF,GRB2,PLCG1,ITPR1,PRKCZ,RAC1
Cell Cycle: G2/M DNA Damage Checkpoint Regulation	1.05E-09	KAT2B,BRCA1,YWHAQ,YWHAG,EP300,YWHAE,SKP2,CDKN1A,MDM2,PRKDC,YWHAZ,PRKCZ,CHEK1,MDM4
Phospholipase C Signaling	1.20E-09	CREB1,CALM1 (includes others),ADCY6,PRKCQ,GNB1,PPP3CC,ADCY10,DIRAS3,GNAQ,FYN,GRB2,CREBBP,ITPR1,PRKCZ,CD79A,PLCB1,CD3E,SRC,GNB2L1,CD3G,RAF1,RPS6KA3,EP300,HMOX1,ATF4,ITPR3,PLCG1,RAC1,AHNAK,LYN
RANK Signaling in Osteoclasts	1.35E-09	TRAF2,MAPK14,SRC,MAP3K5,CHUK,TNFSF11,CALM1 (includes others),AKT1,MAP3K11,RAF1,IKBKB,PIK3R1,NFKBIA,AKT2,PPP3CC,MAP2K4,MAP3K8,XIAP
ATM Signaling	1.41E-09	BRCA1,MAPK14,CREB1,EP300,NFKBIA,SMC2,SMC3,ATF4,MAP2K4,CDKN1A,TP73,CREBBP,MDM2,CHEK1,MDM4
AMPK Signaling	1.48E-09	KAT2B,MAPK14,PPM1A,SRC,MTOR,AKT1,PFKFB2,PPP2R4,IRS1,SMARCE1,PIK3R1,SMARCC1,AKT2,PFKFB1,SMARCB1,PDPK1,TSC2,PPP2R1A,NOS3,TSC1,EIF4EBP1,RPS6KB1,PRKAB2,PPP2CA
iCOS-iCOSL Signaling in T Helper Cells	1.62E-09	CD3E,PTEN,CHUK,CALM1 (includes others),AKT1,CD3G,PRKCQ,IKBKB,PIK3R1,NFKBIA,AKT2,PPP3CC,BAD,PDPK1,GAB2,ITPR3,GRB2,PLCG1,ITPR1,RAC1
mTOR Signaling	2.19E-09	AKT1,PRKCQ,PPP2R4,RICTOR,IRS1,PIK3R1,DIRAS3,TSC2,PPP2R1A,TSC1,PRKCZ,EIF4EBP1,RPS6KB1,PPP2CA,AKT1S1,MTOR,VEGFA,RPS6KA3,HMOX1,AKT2,PDPK1,EIF3H,RHEB,RAC1,EIF3F,PRKAB2
Non-Small Cell Lung Cancer Signaling	2.34E-09	EGFR,AKT1,RAF1,CASP9,PIK3R1,AKT2,BAD,EGF,PDPK1,ITPR3,ERBB2,GRB2,PLCG1,ITPR1,FOXO3,STK4
p38 MAPK Signaling	3.02E-09	IRAK1,TRAF2,MAPK14,MAP3K5,CREB1,TNFRSF1A,TNFRSF1B,RPS6KA3,EP300,TRADD,MAPKAPK2,HSPB1,ATF4,MAP2K4,STAT1,TNF,CREBBP,TGFBR1,MAPT,RPS6KB1
Docosahexaenoic Acid (DHA) Signaling	3.63E-09	GSK3B,AKT1,CASP9,APP,GSK3A,PIK3R1,AKT2,BAD,PDPK1,BCL2,CASP3,BCL2L1,FOXO1
Endometrial Cancer Signaling	3.89E-09	GSK3B,PTEN,AKT1,CTNNB1,RAF1,CASP9,PIK3R1,ILK,AKT2,BAD,PDPK1,ERBB2,GRB2,FOXO3
Ephrin B Signaling	5.37E-09	GNAI1,GNAZ,CDC42,GNA11,ITSN1,GNB2L1,GNAO1,CTNNB1,PAK1,LIMK1,GNB1,GNAI3,GNAQ,GNAI2,RAC1,KALRN
VEGF Signaling	6.31E-09	SRC,AKT1,VEGFA,RAF1,PIK3R1,YWHAE,AKT2,BAD,ACTG1,BCL2,GRB2,ACTN2,PLCG1,NOS3,ELAVL1,BCL2L1,FOXO1,FOXO3
ErbB2-ErbB3 Signaling	8.13E-09	GSK3B,PTEN,AKT1,RAF1,NRG1,GSK3A,PIK3R1,BAD,PDPK1,CDKN1B,ERBB2,GRB2,ERBB3,FOXO1
Synaptic Long Term Depression	9.12E-09	GNAI1,PLCB1,GNAZ,GNA11,GNAO1,RAF1,PRKCQ,PPP2R4,GNAI3,GRM3,GNAQ,GNAI2,ITPR3,GRIA2,PPP2R1A,PLCG1,ITPR1,LCAT,NOS3,PRKCZ,PPP2CA,LYN
Role of PKR in Interferon Induction and Antiviral Response	9.77E-09	TRAF2,MAPK14,CHUK,IFNG,AKT1,TNFRSF1A,STAT1,TNF,CASP9,IKBKB,CASP3,NFKBIA
Myc Mediated Apoptosis Signaling	1.02E-08	YWHAQ,YWHAG,AKT1,CASP9,PIK3R1,YWHAE,AKT2,BAD,MAP2K4,BCL2,GRB2,CASP3,YWHAZ,PRKCZ

IL-12 Signaling and Production in Macrophages	1.05E-08	MAPK14,CHUK,IFNG,AKT1,PRKCQ,EP300,IKBKB,MST1R,PIK3R1,APOC1,AKT2,APOC2,MAP2K4,STAT1,TNF,CLU,MAP3K8,APOE,APOC4,PRKCZ,ALB
Synaptic Long Term Potentiation	1.20E-08	PLCB1,GNA11,CALM1 (includes others),CREB1,RAF1,PRKCQ,EP300,PPP1CA,ATP,PPP3CC,GRM3,GNAQ,ATF4,ITPR3,GRIA2,CREBBP,PLCG1,ITPR1,PRKCZ,PPP1R11
Regulation of the Epithelial-Mesenchymal Transition Pathway	1.55E-08	GSK3B,FGFR1,EGFR,JAK2,AKT1,NOTCH1,CTNNB1,SMAD2,RAF1,RBPJ,NOTCH4,PIK3R1,SMAD4,AKT2,EGF,ADAM17,BRAF,MAP2K4,PSEN2,SMAD3,MAML1,GRB2,TGFBR1,PSEN1
Wnt/Î²-catenin Signaling	1.58E-08	GSK3B,SRC,AKT1,RARA,GNAO1,CSNK2B,CTNNB1,PPP2R4,EP300,GSK3A,ILK,AKT2,CSNK2A1,CD44,GNAQ,APPL1,PPP2R1A,CREBBP,LRP1,TGFBR1,MDM2,DKK4,PPP2CA
Chronic Myeloid Leukemia Signaling	1.78E-08	E2F6,CHUK,AKT1,RAF1,IKBKB,PIK3R1,SMAD4,AKT2,BAD,GAB2,CDKN1B,SMAD3,CDKN1A,GRB2,TGFBR1,MDM2,BCL2L1
IL-3 Signaling	1.82E-08	JAK2,AKT1,RAF1,PRKCQ,PAK1,PIK3R1,AKT2,PPP3CC,BAD,GAB2,STAT1,GRB2,PRKCZ,RAC1,FOXO1
ErbB4 Signaling	2.40E-08	AKT1,ERBB4,RAF1,PRKCQ,YAP1,NRG1,PIK3R1,ADAM17,PDPK1,PSEN2,GRB2,PLCG1,PRKCZ,PSEN1
Signaling by Rho Family GTPases	2.82E-08	GNAI1,CDC42,GNA11,GNAO1,LIMK1,GNB1,PIK3R1,SEPT5,GNAI3,VIM,DIRAS3,GNAQ,GNAI2,ACTG1,PRKCZ,GNAZ,MAP3K11,GNB2L1,RAF1,PAK1,RDX,NEDD4,ARFIP2,MAP2K4,PAK6,CIT,RAC1
STAT3 Pathway	3.24E-08	FGFR1,MAPK14,EGFR,SRC,JAK2,MAP3K11,RAF1,BMPR1B,MAP2K4,BCL2,CDKN1A,NTRK3,TGFBR1,NTRK2,RAC1
Melanocyte Development and Pigmentation Signaling	3.80E-08	SRC,CREB1,ADCY6,RAF1,RPS6KA3,EP300,ATP,PIK3R1,ADCY10,ATF4,BCL2,GRB2,CREBBP,PLCG1,SH2B2,RPS6KB1
Lymphotoxin Î² Receptor Signaling	5.75E-08	TRAF2,CHUK,AKT1,EP300,CASP9,IKBKB,PIK3R1,NFKBIA,AKT2,PDPK1,CASP3,CREBBP,BCL2L1
FGF Signaling	6.17E-08	FGFR1,MAPK14,MAP3K5,AKT1,CREB1,RAF1,EP300,PIK3R1,AKT2,MAPKAPK2,ATF4,GRB2,CREBBP,PLCG1,ITPR1,RAC1
FAK Signaling	7.24E-08	EGFR,SRC,PTEN,AKT1,RAF1,PAK1,PIK3R1,AKT2,EGF,FYN,PDPK1,ACTG1,PAK6,GRB2,PLCG1,RAC1
Fc Epsilon RI Signaling	7.41E-08	MAPK14,AKT1,RAF1,PRKCQ,INPPL1,PIK3R1,AKT2,MS4A2,FYN,PDPK1,MAP2K4,TNF,INPP5K,GRB2,PLCG1,PRKCZ,RAC1,LYN
Angiopoietin Signaling	7.94E-08	CHUK,AKT1,PAK1,CASP9,IKBKB,PIK3R1,NFKBIA,AKT2,BAD,PAK6,GRB2,NOS3,NCK1,FOXO1
Agrin Interactions at Neuromuscular Junction	1.17E-07	CDC42,EGFR,SRC,ERBB4,PAK1,NRG1,ACTG1,MAP2K4,PAK6,ERBB2,ITGB3,ERBB3,RAC1,UTRN
Aryl Hydrocarbon Receptor Signaling	1.20E-07	NCOR2,SRC,AHRR,RARA,NQO2,EP300,CCND3,ALDH3A2,HSPB1,CDKN1B,TNF,CDKN1A,TP73,ESR2,MDM2,CCNA2,HSP90AB1,HSP90AA1,ESR1,CHEK1
JAK/Stat Signaling	1.66E-07	MTOR,PIAS2,JAK2,AKT1,RAF1,PIK3R1,PIAS1,AKT2,PTPN1,GNAQ,STAT1,CDKN1A,GRB2,BCL2L1
Ceramide Signaling	1.78E-07	AKT1,TNFRSF1A,TNFRSF1B,RAF1,PPP2R4,PIK3R1,AKT2,BAD,MAP2K4,TNF,BCL2,PPP2R1A,PRKCZ,PPP2CA,S1PR1
CCR3 Signaling in Eosinophils	1.78E-07	GNAI1,PLCB1,MAPK14,CALM1 (includes others),GNB2L1,RAF1,PRKCQ,PAK1,LIMK1,GNB1,PIK3R1,GNAI3,GNAI2,ITPR3,PAK6,ITPR1,PRKCZ,RAC1
PKCÎ¸ Signaling in T Lymphocytes	1.78E-07	POU2F1,CD3E,MAP3K5,CHUK,MAP3K11,CD3G,PRKCQ,IKBKB,PIK3R1,NFKBIA,PPP3CC,FYN,BCL10,MAP2K4,MAP3K8,GRB2,PLCG1,RAC1

LPS-stimulated MAPK Signaling	1.95E-07	MAPK14,CDC42,MAP3K5,CHUK,CREB1,RAF1,PRKCQ,PAK1,IKBKB,PIK3R1,NFKBIA,MAP2K4,PRKCZ,RAC1
Î±-Adrenergic Signaling	2.00E-07	GNAI1,CALM1 (includes others),GNB2L1,ADCY6,RAF1,PRKCQ,GNB1,ATP,GNAI3,ADCY10,GNAQ,GNAI2,ITPR3,PLCG1,ITPR1,PRKCZ
GÎ±i Signaling	2.00E-07	GNAI1,SRC,GNB2L1,ADCY6,RAF1,GABBR1,GNB1,ATP,GNAI3,ADCY10,GRM3,DRD2,GNAI2,GRB2,DRD4,RGS4,S1PR1,DRD3
UVB-Induced MAPK Signaling	2.51E-07	MAPK14,EGFR,MTOR,AKT1,MAP2K4,PRKCQ,RPS6KA3,PIK3R1,PRKCZ,EIF4EBP1,RPS6KB1,BAD
Human Embryonic Stem Cell Pluripotency	2.57E-07	GSK3B,FGFR1,AKT1,CTNNB1,SMAD2,NTF3,GSK3A,PIK3R1,SMAD4,AKT2,SMAD7,PDPK1,SMAD3,NTRK3,TGFBR1,NTRK2,BDNF,FOXO1,S1PR1
Telomerase Signaling	2.63E-07	EGFR,AKT1,RAF1,PPP2R4,PIK3R1,AKT2,TERT,EGF,PDPK1,CDKN1A,GRB2,PPP2R1A,HSP90AB1,HSP90AA1,PPP2CA,TERF2IP
TGF-Î² Signaling	2.75E-07	MAPK14,CDC42,SMAD2,RAF1,EP300,SMAD4,BMPR1B,SKI,SMAD7,MAP2K4,SMAD3,BCL2,GRB2,CREBBP,TGFBR1
Glioma Signaling	2.95E-07	E2F6,EGFR,PTEN,MTOR,CALM1 (includes others),AKT1,RAF1,PRKCQ,PIK3R1,AKT2,EGF,CDKN1A,GRB2,PLCG1,MDM2,PRKCZ
Sphingosine-1-phosphate Signaling	2.95E-07	GNAI1,PLCB1,AKT1,ADCY6,CASP9,CASP1,PIK3R1,AKT2,GNAI3,ADCY10,DIRAS3,GNAQ,GNAI2,CASP3,PLCG1,RAC1,S1PR1
Integrin Signaling	3.09E-07	GSK3B,CDC42,SRC,PTEN,AKT1,MAP3K11,RAF1,PAK1,PIK3R1,ILK,AKT2,DIRAS3,FYN,BRAF,ACTG1,MAP2K4,PAK6,GRB2,ACTN2,PLCG1,ITGB3,RAC1,NCK1
fMLP Signaling in Neutrophils	3.80E-07	GNAI1,PLCB1,CDC42,CALM1 (includes others),GNB2L1,RAF1,PRKCQ,GNB1,PIK3R1,NFKBIA,PPP3CC,GNAI3,GNAI2,ITPR3,ITPR1,PRKCZ,RAC1
T Cell Receptor Signaling	3.80E-07	CD3E,CHUK,CALM1 (includes others),CD3G,RAF1,PRKCQ,IKBKB,PIK3R1,NFKBIA,PPP3CC,FYN,BCL10,MAP2K4,GRB2,PLCG1,RAC1
Clathrin-mediated Endocytosis Signaling	3.80E-07	CDC42,RAB11A,SRC,VEGFA,CSNK2B,AMPH,PIK3R1,APOC1,PPP3CC,CSNK2A1,EGF,APOC2,LDLR,ACTG1,CLU,GRB2,ITGB3,APOE,MDM2,APOC4,RAC1,ALB
Leukocyte Extravasation Signaling	3.89E-07	GNAI1,F11R,MAPK14,CDC42,SRC,CTNNB1,PRKCQ,ARHGAP1,PIK3R1,MMP17,CD44,RDX,GNAI3,GNAI2,ACTG1,MAP2K4,DLC1,ACTN2,PLCG1,ITGB3,PRKCZ,RAC1,MMP23B
Endoplasmic Reticulum Stress Pathway	4.47E-07	CALR,TRAF2,ATF4,MAP3K5,MBTPS2,CASP9,CASP3,MBTPS1
TNFR2 Signaling	5.50E-07	TRAF2,CHUK,TNFAIP3,MAP2K4,TNFRSF1B,TNF,IKBKB,XIAP,NFKBIA
Dopamine Receptor Signaling	5.50E-07	COMT,ADCY6,PPP2R4,PPP1CA,ATP,ADCY10,DRD2,SLC6A3,NCS1,PPP2R1A,SLC18A1,DRD4,PPP2CA,PPP1R11,DRD3
Notch Signaling	6.17E-07	ADAM17,NOTCH1,PSEN2,RBPJ,MAML1,NOTCH4,MAML3,MAML2,DLL4,PSEN1
Erythropoietin Signaling	7.59E-07	SRC,JAK2,AKT1,RAF1,PRKCQ,PIK3R1,NFKBIA,AKT2,PDPK1,GRB2,PLCG1,PRKCZ,RPS6KB1
Inhibition of Angiogenesis by TSP1	7.94E-07	MAPK14,FYN,AKT1,VEGFA,MAP2K4,SDC2,CASP3,TGFBR1,NOS3,AKT2
HGF Signaling	8.13E-07	CDC42,MAP3K5,AKT1,MAP3K11,RAF1,PRKCQ,PAK1,PIK3R1,AKT2,MAP2K4,MAP3K8,CDKN1A,GRB2,PLCG1,PRKCZ,RAC1
GDNF Family Ligand-Receptor Interactions	8.71E-07	CDC42,CREB1,RAF1,GFRA2,IRS1,PIK3R1,ITPR3,MAP2K4,GRB2,PLCG1,ITPR1,RAC1,NCK1
Tight Junction Signaling	9.33E-07	F11R,CDC42,PTEN,AKT1,TNFRSF1A,CTNNB1,TNFRSF1B,PPP2R4,MPDZ,NSF,AKT2,EPB41,ACTG1,TNF,PPP2R1A,TGFBR1,PRKCZ,RAC1,PPP2CA,SPTAN1

FXR/RXR Activation	1.00E-06	AKT1,RARA,VLDLR,APOH,SCARB1,APOC1,AKT2,APOC2,MAP2K4,TNF,CLU,CREBBP,LCAT,APOE,APOC4,FOXO1,PLTP,ALB
Renal Cell Carcinoma Signaling	1.20E-06	CDC42,AKT1,VEGFA,RAF1,PAK1,EP300,PIK3R1,AKT2,PAK6,TCEB1,GRB2,CREBBP,RAC1
CCR5 Signaling in Macrophages	1.41E-06	GNAI1,CD3E,MAPK14,CALM1 (includes others),GNB2L1,CD3G,PRKCQ,GNB1,GNAI3,GNAI2,MAP2K4,PLCG1,PRKCZ
Small Cell Lung Cancer Signaling	1.41E-06	TRAF2,PTEN,CHUK,AKT1,CASP9,IKBKB,PIK3R1,NFKBIA,AKT2,CDKN1B,SKP2,BCL2,BCL2L1
CD27 Signaling in Lymphocytes	1.55E-06	TRAF2,MAP3K5,CHUK,MAP2K4,MAP3K11,MAP3K8,CASP9,IKBKB,CASP3,BCL2L1,NFKBIA
GM-CSF Signaling	1.55E-06	HCK,AKT1,JAK2,GNB2L1,STAT1,RAF1,GRB2,BCL2L1,PIK3R1,AKT2,PPP3CC,LYN
Melatonin Signaling	1.86E-06	GNAI1,PLCB1,CALM1 (includes others),GNAO1,RAF1,PRKCQ,GNAI3,GNAQ,GNAI2,BRAF,MAP2K4,PLCG1,PRKCZ
Prolactin Signaling	1.86E-06	JAK2,RAF1,PRKCQ,EP300,IRS1,PIK3R1,FYN,PDPK1,STAT1,GRB2,CREBBP,PLCG1,PRKCZ
UVA-Induced MAPK Signaling	2.00E-06	PLCB1,MAPK14,EGFR,MTOR,RPS6KA3,CASP9,PIK3R1,TNKS,MAP2K4,STAT1,CASP3,PLCG1,BCL2L1,RPS6KB1
Hypoxia Signaling in the Cardiovascular System	2.14E-06	ATF4,PTEN,AKT1,CREB1,VEGFA,EP300,CREBBP,MDM2,NOS3,HSP90AB1,NFKBIA,HSP90AA1
TWEAK Signaling	2.19E-06	TRAF2,CHUK,CASP9,IKBKB,CASP3,XIAP,TRADD,NFKBIA,RIPK1
GPCR-Mediated Nutrient Sensing in Enteroendocrine Cells	2.57E-06	GNAI1,PLCB1,GNA11,ADCY6,PRKCQ,ATP,GNAI3,ADCY10,GNAQ,GNAI2,ITPR3,PLCG1,ITPR1,PRKCZ
Dendritic Cell Maturation	2.63E-06	PLCB1,MAPK14,CHUK,JAK2,AKT1,CREB1,TNFRSF1A,TNFRSF1B,EP300,IKBKB,PIK3R1,NFKBIA,AKT2,ATF4,MAP2K4,CD1D,STAT1,TNF,CREBBP,PLCG1
Ovarian Cancer Signaling	2.63E-06	GSK3B,BRCA1,EGFR,SRC,PTEN,MTOR,AKT1,VEGFA,CTNNB1,RAF1,PIK3R1,AKT2,CD44,EGF,BRAF,BCL2,RPS6KB1
Epithelial Adherens Junction Signaling	2.63E-06	FGFR1,CDC42,EGFR,SRC,PTEN,EPN2,AKT1,NOTCH1,TUBB,CTNNB1,NOTCH4,AKT2,TUBB2A,EGF,ACTG1,ACTN2,TGFBR1,RAC1
GPCR-Mediated Integration of Enteroendocrine Signaling Exemplified by an L Cell	2.82E-06	GNAI1,PLCB1,GNA11,ADCY6,ATP,GNAI3,ADCY10,GNAQ,GNAI2,ITPR3,PLCG1,ITPR1,SSTR5
Regulation of eIF4 and p70S6K Signaling	3.16E-06	MAPK14,MTOR,AKT1,RAF1,PPP2R4,IRS1,PIK3R1,AKT2,PDPK1,EIF3H,GRB2,PPP2R1A,AGO3,PRKCZ,EIF4EBP1,EIF3F,RPS6KB1,PPP2CA
Calcium-induced T Lymphocyte Apoptosis	3.39E-06	CD3E,CALM1 (includes others),ITPR3,CABIN1,CD3G,PRKCQ,NR4A1,EP300,PLCG1,ITPR1,PRKCZ,PPP3CC
Rac Signaling	3.47E-06	CDC42,MAP3K11,RAF1,PAK1,LIMK1,PIK3R1,SH3RF1,CD44,BRK1,ARFIP2,MAP2K4,PAK6,PRKCZ,RAC1,RPS6KB1
PPAR Signaling	3.72E-06	NCOR2,TRAF2,CHUK,TNFRSF1A,TNFRSF1B,RAF1,EP300,IKBKB,NFKBIA,TNF,GRB2,CREBBP,HSP90AB1,HSP90AA1
iNOS Signaling	4.37E-06	IRAK1,MAPK14,CHUK,IFNG,CALM1 (includes others),JAK2,STAT1,IKBKB,CREBBP,NFKBIA
Xenobiotic Metabolism Signaling	5.01E-06	NCOR2,MAPK14,PRKCQ,PPP2R4,NQO2,PIK3R1,ALDH3A2,TNF,MAP3K8,PPP2R1A,CREBBP,GRIP1,PRKCZ,HSP90AA1,PPP2CA,MAP3K5,AHRR,MAP3K11,RAF1,EP300,HMOX1,MAP2K4,CYP2C8,HSP90AB1,DNAJC7
PEDF Signaling	5.25E-06	MAPK14,CHUK,AKT1,RAF1,BCL2,IKBKB,BCL2L1,PIK3R1,NFKBIA,AKT2,BDNF,RAC1
LXR/RXR Activation	6.92E-06	NCOR2,TNFRSF1A,TNFRSF1B,APOH,APOC1,APOC2,LDLR,TNF,CLU,APOA5,CYP51A1,LCAT,APOE,APOC4,PLTP,ALB

Caveolar-mediated Endocytosis Signaling	7.08E-06	EGF,EGFR,SRC,FYN,ITSN1,ACTG1,FLNA,COPB2,COPB1,ITGB3,PTPN1,ALB
NF-Î°B Activation by Viruses	8.13E-06	TRAF2,CHUK,AKT1,RAF1,PRKCQ,IKBKB,ITGB3,PIK3R1,NFKBIA,PRKCZ,AKT2,RIPK1
Natural Killer Cell Signaling	8.32E-06	AKT1,RAF1,PRKCQ,PAK1,INPPL1,PIK3R1,AKT2,FYN,PAK6,INPP5K,GRB2,PLCG1,PRKCZ,RAC1,NCK1
Paxillin Signaling	8.51E-06	MAPK14,CDC42,SRC,PAK1,PIK3R1,ARFIP2,ACTG1,MAP2K4,PAK6,GRB2,ACTN2,ITGB3,RAC1,NCK1
NRF2-mediated Oxidative Stress Response	1.05E-05	GSK3B,MAPK14,MAP3K5,AKT1,DNAJB1,RAF1,PRKCQ,NQO2,EP300,SCARB1,PIK3R1,HMOX1,ATF4,ACTG1,MAP2K4,CREBBP,PRKCZ,DNAJC7,JUNB
CNTF Signaling	1.10E-05	MTOR,AKT1,JAK2,STAT1,RAF1,RPS6KA3,GRB2,CNTF,PIK3R1,RPS6KB1
Amyloid Processing	1.10E-05	GSK3B,MAPK14,AKT1,PSEN2,CSNK2B,APP,MAPT,AKT2,CSNK2A1,PSEN1
Cell Cycle: G1/S Checkpoint Regulation	1.15E-05	E2F6,CCND3,GSK3B,CDKN1B,SMAD3,SKP2,CDKN1A,NRG1,MDM2,SMAD4,FOXO1
HIF1Î± Signaling	1.15E-05	MAPK14,AKT1,VEGFA,EP300,PIK3R1,MMP17,AKT2,EGLN1,TCEB1,CREBBP,MDM2,NOS3,HSP90AA1,MMP23B
Neuropathic Pain Signaling In Dorsal Horn Neurons	1.15E-05	PLCB1,SRC,CREB1,PRKCQ,PIK3R1,GRM3,ITPR3,KCNN3,GRIA2,PLCG1,ITPR1,NTRK2,PRKCZ,BDNF
Acute Myeloid Leukemia Signaling	1.20E-05	BRAF,MTOR,AKT1,RARA,MAP2K4,RAF1,GRB2,PIK3R1,AKT2,EIF4EBP1,RPS6KB1,BAD
Semaphorin Signaling in Neurons	1.29E-05	PLXNA1,DIRAS3,FYN,PAK6,PAK1,DPYSL3,LIMK1,ARHGAP1,DPYSL2,RAC1
HMGB1 Signaling	1.32E-05	KAT2B,MAPK14,CDC42,IFNG,AKT1,TNFRSF1A,TNFRSF1B,PIK3R1,AKT2,KAT6A,DIRAS3,MAP2K4,TNF,CNTF,RAC1
Role of BRCA1 in DNA Damage Response	1.35E-05	E2F6,POU2F1,BRCA1,TOPBP1,IFNG,STAT1,CDKN1A,FANCA,SMARCE1,SMARCC1,SMARCB1,CHEK1
Leptin Signaling in Obesity	1.35E-05	PLCB1,AKT1,JAK2,ADCY6,PDE3B,GRB2,PLCG1,ATP,PIK3R1,AKT2,FOXO1,ADCY10
Cholecystokinin/Gastrin-mediated Signaling	1.41E-05	PLCB1,MAPK14,EGFR,SRC,RAF1,PRKCQ,DIRAS3,GNAQ,ITPR3,MAP2K4,TNF,GRB2,ITPR1,PRKCZ
Hepatic Fibrosis / Hepatic Stellate Cell Activation	1.45E-05	FGFR1,COL4A1,EGFR,IFNG,TNFSF11,TNFRSF1A,VEGFA,TNFRSF1B,SMAD2,SMAD4,IL10RA,A2M,EGF,SMAD7,STAT1,TNF,SMAD3,BCL2,CSF1,TGFBR1
Estrogen-Dependent Breast Cancer Signaling	1.48E-05	EGFR,SRC,ATF4,AKT1,CREB1,EP300,CREBBP,PIK3R1,AKT2,TERT,ESR1
IL-17A Signaling in Airway Cells	1.48E-05	GSK3B,MAPK14,CHUK,PTEN,AKT1,JAK2,MAP2K4,IKBKB,PIK3R1,NFKBIA,AKT2
Estrogen-mediated S-phase Entry	1.58E-05	E2F6,CDKN1B,SKP2,CDKN1A,ESR2,CCNA2,ESR1
IL-15 Signaling	1.70E-05	TRAF2,MAPK14,AKT1,JAK2,TNF,RAF1,BCL2,PLCG1,BCL2L1,PIK3R1,AKT2
Melanoma Signaling	1.82E-05	BRAF,PTEN,AKT1,RAF1,CDKN1A,MDM2,PIK3R1,AKT2,BAD
Macropinocytosis Signaling	1.95E-05	EGF,CDC42,SRC,PAK1,PRKCQ,CSF1,PLCG1,ITGB3,PIK3R1,PRKCZ,RAC1
SAPK/JNK Signaling	1.95E-05	TRAF2,CDC42,MAP3K5,MAP3K11,GNB1,IRS1,PIK3R1,TRADD,MAPK8IP1,MAP2K4,GRB2,RAC1,RIPK1
PDGF Signaling	2.45E-05	SRC,JAK2,MAP2K4,CSNK2B,STAT1,RAF1,INPP5K,GRB2,PLCG1,INPPL1,PIK3R1,CSNK2A1
Nur77 Signaling in T Lymphocytes	2.75E-05	CD3E,CALM1 (includes others),CABIN1,CD3G,NR4A1,BCL2,EP300,CASP9,CASP3,PPP3CC
Antiproliferative Role of TOB in T Cell Signaling	2.75E-05	CDKN1B,SMAD2,SMAD3,SKP2,CCNA2,TGFBR1,SMAD4
Hepatic Cholestasis	2.75E-05	IRAK1,TRAF2,CHUK,IFNG,RARA,TNFRSF1A,ADCY6,TNFRSF1B,PRKCQ,IKBKB,ATP,NFKBIA,ADCY10,MAP2K4,TNF,CNTF,PRKCZ,ESR1

GÎ±s Signaling	2.82E-05	HCK,ADORA2A,SRC,CREB1,GNB2L1,ADCY6,EP300,GNB1,ATP,ADCY10,BRAF,ATF4,RGS2,CREBBP
Role of MAPK Signaling in the Pathogenesis of Influenza	3.31E-05	MAPK14,MAP3K5,IFNG,AKT1,MAP2K4,TNF,RAF1,BCL2,LCAT,CASP3,AKT2
Growth Hormone Signaling	3.72E-05	A2M,PDPK1,JAK2,STAT1,PRKCQ,RPS6KA3,IRS1,PLCG1,PIK3R1,PRKCZ,RPS6KB1
CTLA4 Signaling in Cytotoxic T Lymphocytes	4.37E-05	CD3E,FYN,AKT1,JAK2,CD3G,PPP2R4,PPP2R1A,GRB2,PLCG1,PIK3R1,AKT2,PPP2CA
CDK5 Signaling	4.57E-05	MAPK14,ADCY6,RAF1,PPP2R4,PPP1CA,ATP,ADCY10,PPP2R1A,NTRK2,MAPT,BDNF,PPP2CA,PPP1R11
Chemokine Signaling	4.79E-05	GNAI1,PLCB1,GNAI2,MAPK14,GNAQ,SRC,CALM1 (includes others),RAF1,LIMK1,PLCG1,GNAI3
PAK Signaling	6.03E-05	CDC42,MAP2K4,PAK6,TNF,RAF1,PAK1,LIMK1,GRB2,CASP3,PIK3R1,RAC1,NCK1
TR/RXR Activation	6.76E-05	NCOR2,MTOR,LDLR,AKT1,PDE3B,APOA5,EP300,SCARB1,MDM2,PIK3R1,NCOA4,AKT2
Amyotrophic Lateral Sclerosis Signaling	6.76E-05	GLUL,VEGFA,PAK1,CASP9,CASP1,PIK3R1,GRIA2,BCL2,NEFM,CASP3,XIAP,BCL2L1,RAC1
Cyclins and Cell Cycle Regulation	6.92E-05	E2F6,CCND3,GSK3B,CDKN1B,RAF1,PPP2R4,SKP2,CDKN1A,PPP2R1A,CCNA2,PPP2CA
VDR/RXR Activation	7.76E-05	NCOR2,IFNG,TNFSF11,CDKN1B,PRKCQ,CDKN1A,MXD1,EP300,PRKCZ,FOXO1,CSNK2A1
Cardiac Î²-adrenergic Signaling	8.13E-05	TDP2,GNB2L1,ADCY6,PPP2R4,PDE3B,PPP1CA,GNB1,ATP,PDE4B,ADCY10,PPP2R1A,AKAP9,AKAP6,PPP2CA,PPP1R11
4-1BB Signaling in T Lymphocytes	8.91E-05	TRAF2,MAPK14,MAP3K5,CHUK,MAP2K4,IKBKB,NFKBIA
Virus Entry via Endocytic Pathways	9.12E-05	CDC42,SRC,FYN,ITSN1,ACTG1,FLNA,PRKCQ,PLCG1,ITGB3,PIK3R1,PRKCZ,RAC1
Mouse Embryonic Stem Cell Pluripotency	9.12E-05	GSK3B,MAPK14,AKT1,JAK2,CTNNB1,RAF1,GRB2,CREBBP,XIAP,PIK3R1,SMAD4,AKT2
UVC-Induced MAPK Signaling	9.12E-05	MAPK14,EGFR,BRAF,SRC,MAP2K4,RAF1,PRKCQ,PRKCZ
Unfolded protein response	9.12E-05	CALR,TRAF2,CEBPD,ATF4,MAP3K5,MBTPS2,BCL2,CANX,MBTPS1
Role of PI3K/AKT Signaling in the Pathogenesis of Influenza	1.05E-04	GNAI1,GSK3B,GNAI2,IFNG,AKT1,CASP9,PIK3R1,NFKBIA,AKT2,GNAI3
Nitric Oxide Signaling in the Cardiovascular System	1.15E-04	CALM1 (includes others),AKT1,VEGFA,PRKCQ,PDE3B,PIK3R1,AKT2,ITPR3,ITPR1,NOS3,HSP90AB1,PRKCZ,HSP90AA1
Interferon Signaling	1.62E-04	IFNG,JAK2,STAT1,IFIT3,BCL2,PIAS1,PSMB8
Neuroprotective Role of THOP1 in Alzheimer's Disease	1.78E-04	IFNG,CREB1,NFYA,APP,ATP,MAPT,ACE,YWHAE
LPS/IL-1 Mediated Inhibition of RXR Function	1.78E-04	IRAK1,TRAF2,RARA,TNFRSF1A,TNFRSF1B,SLC27A4,SCARB1,APOC1,ALDH3A2,APOC2,PPARGC1B,MAP2K4,TNF,CYP2C8,APOE,SLC27A3,APOC4,ECSIT,PLTP
Thrombopoietin Signaling	1.82E-04	GAB2,JAK2,STAT1,RAF1,PRKCQ,GRB2,PLCG1,PIK3R1,PRKCZ
MSP-RON Signaling Pathway	2.04E-04	IFNG,JAK2,ACTG1,TNF,CSF1,MST1R,PIK3R1,PRKCZ
Parkinson's Signaling	2.14E-04	MAPK14,SNCA,CASP9,CASP3,SEPT5
BMP signaling pathway	2.95E-04	MAPK14,SMAD7,CREB1,MAP2K4,RAF1,GRB2,CREBBP,XIAP,SMAD4,BMPR1B
IL-4 Signaling	3.31E-04	MTOR,AKT1,JAK2,INPP5K,GRB2,IRS1,INPPL1,PIK3R1,AKT2,RPS6KB1
Activation of IRF by Cytosolic Pattern Recognition Receptors	3.39E-04	CHUK,MAP2K4,STAT1,TNF,IKBKB,CREBBP,ZBP1,NFKBIA,RIPK1
Aldosterone Signaling in Epithelial Cells	3.72E-04	PLCB1,DNAJB1,RAF1,PRKCQ,PIK3R1,HSPB1,NEDD4,PDPK1,ITPR3,PLCG1,ITPR1,HSP90AB1,PRKCZ,HSP90AA1,DNAJC7

CD40 Signaling	3.80E-04	TRAF2,MAPK14,CHUK,TNFAIP3,MAP2K4,IKBKB,PIK3R1,NFKBIA,MAPKAPK2
VEGF Family Ligand-Receptor Interactions	4.90E-04	AKT1,VEGFA,RAF1,PRKCQ,GRB2,PLCG1,NOS3,PIK3R1,PRKCZ,AKT2
Hereditary Breast Cancer Signaling	4.90E-04	BRCA1,PTEN,AKT1,EP300,SMARCE1,PIK3R1,SMARCC1,AKT2,SMARCB1,CDKN1A,FANCA,CREBBP,CHEK1
Cdc42 Signaling	5.75E-04	GSK3B,CD3E,MAPK14,EXOC4,CDC42,SRC,MAP3K11,CD3G,RAF1,PAK1,LIMK1,EXOC1,MAP2K4,PRKCZ,EXOC7
GÎ±12/13 Signaling	6.76E-04	CDC42,SRC,MAP3K5,CHUK,AKT1,MAP2K4,CTNNB1,RAF1,IKBKB,PIK3R1,NFKBIA,AKT2
Sperm Motility	7.08E-04	PLCB1,CALM1 (includes others),MAP3K11,PRKCQ,MST1R,ATP,PDE4B,ADCY10,ITPR3,PLCG1,ITPR1,LCAT,PRKCZ
Antioxidant Action of Vitamin C	8.32E-04	PLCB1,MAPK14,CHUK,JAK2,MAP2K4,TNF,IKBKB,LCAT,PLCG1,NFKBIA,HMOX1
Role of RIG1-like Receptors in Antiviral Innate Immunity	9.33E-04	TRAF2,CHUK,EP300,IKBKB,CREBBP,NFKBIA,RIPK1
FcÎ³RIIB Signaling in B Lymphocytes	9.33E-04	PDPK1,AKT1,MAP2K4,GRB2,PIK3R1,CD79A,LYN
Pyridoxal 5'-phosphate Salvage Pathway	1.00E-03	IRAK1,BRAF,MAP2K4,PAK1,PRKCQ,MAP3K8,LIMK1,ATP,AKT2
Toll-like Receptor Signaling	1.00E-03	IRAK1,MAPK14,CHUK,TNFAIP3,MAP2K4,TNF,IKBKB,NFKBIA,ECSIT
Cellular Effects of Sildenafil (Viagra)	1.05E-03	PLCB1,CALM1 (includes others),ADCY6,PDE3B,ATP,PDE4B,ADCY10,ITPR3,ACTG1,KCNN3,PLCG1,ITPR1,NOS3
Protein Ubiquitination Pathway	1.05E-03	BRCA1,IFNG,DNAJB1,PSMC3,ATP,HSPB1,NEDD4,FBXW7,SKP2,TCEB1,SUGT1,XIAP,MDM2,HSP90AB1,HSP90AA1,USP48,USP4,DNAJC7,PSMB8
Factors Promoting Cardiogenesis in Vertebrates	1.20E-03	GSK3B,MAPK14,CTNNB1,SMAD2,PRKCQ,LRP1,TGFBR1,SMAD4,PRKCZ,BMPR1B
Adipogenesis pathway	1.35E-03	FGFR1,EZH2,FBXW7,CEBPD,AKT1,TNFRSF1A,TNF,SMAD3,FOXO1,RPS6KB1,SIRT1,SETDB1
IL-17A Signaling in Fibroblasts	1.38E-03	GSK3B,MAPK14,CEBPD,CHUK,IKBKB,NFKBIA
Role of NANOG in Mammalian Embryonic Stem Cell Pluripotency	1.41E-03	GSK3B,AKT1,JAK2,CTNNB1,TCL1A,RAF1,GRB2,PIK3R1,SMAD4,AKT2,BMPR1B
Calcium Signaling	1.51E-03	CALM1 (includes others),CREB1,EP300,CHRNA7,ATP,ATP2B1,CAMKK1,PPP3CC,CALR,ATF4,CABIN1,ITPR3,GRIA2,CREBBP,ITPR1
IL-22 Signaling	1.51E-03	MAPK14,AKT1,MAP2K4,STAT1,AKT2
G Protein Signaling Mediated by Tubby	1.58E-03	PLCB1,GNAQ,JAK2,GNB2L1,GNB1,PLCG1
Glutamate Receptor Signaling	1.62E-03	GLUL,DLG4,CALM1 (includes others),PICK1,GRIA2,GNB1,GRIP1,GRM3
Role of JAK family kinases in IL-6-type Cytokine Signaling	1.82E-03	MAPK14,JAK2,MAP2K4,STAT1,OSMR
D-myo-inositol (1,3,4)-trisphosphate Biosynthesis	1.82E-03	PTEN,INPP5K,ITPKC,ATP,INPPL1
Mitotic Roles of Polo-Like Kinase	1.95E-03	RAD21,PPP2R4,PPP2R1A,HSP90AB1,HSP90AA1,SMC3,PPP2CA,CDC27
April Mediated Signaling	2.09E-03	TRAF2,MAPK14,CHUK,MAP2K4,IKBKB,NFKBIA
IL-15 Production	2.63E-03	JAK2,MAP3K11,STAT1,MST1R,PRKCZ
B Cell Activating Factor Signaling	2.69E-03	TRAF2,MAPK14,CHUK,MAP2K4,IKBKB,NFKBIA
Inhibition of Matrix Metalloproteases	2.69E-03	A2M,ADAM17,SDC2,LRP1,MMP17,MMP23B
Role of CHK Proteins in Cell Cycle Checkpoint Control	2.95E-03	E2F6,BRCA1,PPP2R4,CDKN1A,PPP2R1A,PPP2CA,CHEK1

IL-2 Signaling	2.95E-03	AKT1,CSNK2B,RAF1,GRB2,PIK3R1,AKT2,CSNK2A1
Bladder Cancer Signaling	2.95E-03	EGF,EGFR,VEGFA,RAF1,CDKN1A,ERBB2,MDM2,MMP17,MMP23B
Actin Cytoskeleton Signaling	3.02E-03	CDC42,FLNA,RAF1,PAK1,LIMK1,PIK3R1,BRK1,RDX,EGF,ACTG1,PAK6,TRIO,GRB2,ACTN2,TIAM2,RAC1
Thyroid Cancer Signaling	3.02E-03	BRAF,CTNNB1,NTF3,NTRK3,NTRK2,BDNF
IL-10 Signaling	3.31E-03	MAPK14,CHUK,MAP2K4,TNF,IKBKB,NFKBIA,HMOX1,IL10RA
IL-17 Signaling	3.31E-03	GSK3B,MAPK14,AKT1,JAK2,MAP2K4,PIK3R1,AKT2,MAPKAPK2
Estrogen Receptor Signaling	3.89E-03	KAT2B,NCOR2,SRC,RAF1,EP300,GRB2,CREBBP,PHB2,ESR2,PRKDC,ESR1
Regulation of Cellular Mechanics by Calpain Protease	3.89E-03	EGF,EGFR,SRC,CDKN1B,GRB2,ACTN2,CCNA2
Salvage Pathways of Pyrimidine Ribonucleotides	4.27E-03	IRAK1,BRAF,NME3,MAP2K4,PAK1,PRKCQ,MAP3K8,LIMK1,ATP,AKT2
Role of IL-17F in Allergic Inflammatory Airway Diseases	4.27E-03	ATF4,CREB1,RAF1,RPS6KA3,EP300,CREBBP
Wnt/Ca+ pathway	4.27E-03	PLCB1,GSK3B,ATF4,CREB1,EP300,CREBBP,PLCG1
TREM1 Signaling	4.57E-03	IRAK1,AKT1,JAK2,TNF,GRB2,CASP1,PLCG1,AKT2
Cytotoxic T Lymphocyte-mediated Apoptosis of Target Cells	5.37E-03	CD3E,CD3G,BCL2,CASP9,CASP3
Superpathway of D-myo-inositol (1,4,5)-trisphosphate Metabolism	5.37E-03	PTEN,INPP5K,ITPKC,ATP,INPPL1
Oncostatin M Signaling	7.08E-03	JAK2,STAT1,RAF1,GRB2,OSMR
Role of JAK2 in Hormone-like Cytokine Signaling	7.94E-03	JAK2,STAT1,IRS1,SH2B2,PTPN1
Ephrin A Signaling	8.13E-03	CDC42,FYN,PAK1,LIMK1,PIK3R1,RAC1
Antiproliferative Role of Somatostatin Receptor 2	8.71E-03	BRAF,SRC,GNB2L1,CDKN1B,CDKN1A,GNB1,PIK3R1
RhoA Signaling	9.77E-03	PLXNA1,NEDD4,ACTG1,KTN1,DLC1,CIT,LIMK1,ARHGAP1,SEPT5,RDX

Overlap of Pathways between GWAS and Historic Interactomes

Pathway	P-value Nature	# of Genes Historical Gene Interactome	# of Genes Common	# of Genes GWAS Gene Interactome
Molecular Mechanisms of Cancer	7.94328E-25	44	22	48
RAR Activation	5.01187E-19	21	12	30
Role of NFAT in Cardiac Hypertrophy	1E-18	18	11	31
Glioma Signaling	1.25893E-17	7	9	21
Huntington's Disease Signaling	1E-16	24	12	33
UVB-Induced MAPK Signaling	3.16228E-16	5	7	15
Neuropathic Pain Signaling In Dorsal Horn Neurons	6.30957E-16	8	6	23
14-3-3-mediated Signaling	1E-15	23	11	20
G Beta Gamma Signaling	1.25893E-15	15	9	18
ERK/MAPK Signaling	1.25893E-15	13	14	25
p70S6K Signaling	1.58489E-15	17	14	17
Androgen Signaling	1.58489E-15	15	8	22
GNRH Signaling	2.51189E-15	23	8	24
ErbB Signaling	6.30957E-15	13	11	15
Role of Macrophages, Fibroblasts and Endothelial Cells in Rheumatoid Arthritis	6.30957E-15	26	14	35
Fc? Receptor-mediated Phagocytosis in Macrophages and Monocytes	6.30957E-15	0	0	27
Synaptic Long Term Potentiation	1.25893E-14	13	7	23
Glucocorticoid Receptor Signaling	1.58489E-14	31	12	33
Protein Kinase A Signaling	1.58489E-14	32	16	40
Axonal Guidance Signaling	1.99526E-14	37	12	48
PI3K/AKT Signaling	3.16228E-14	27	21	9
Fc Epsilon RI Signaling	5.01187E-14	10	8	20
Dopamine-DARPP32 Feedback in cAMP Signaling	6.30957E-14	24	7	27
Prolactin Signaling	7.94328E-14	4	9	14
IGF-1 Signaling	1.58489E-13	10	12	14
CCR5 Signaling in Macrophages	1.99526E-13	7	6	16
Production of Nitric Oxide and Reactive Oxygen Species in Macrophages	3.16228E-13	24	10	25
Renin-Angiotensin Signaling	3.98107E-13	15	6	21
P2Y Purigenic Receptor Signaling Pathway	6.30957E-13	14	8	20
B Cell Receptor Signaling	7.94328E-13	23	14	20
UVC-Induced MAPK Signaling	1.25893E-12	4	4	13
HER-2 Signaling in Breast Cancer	1.99526E-12	15	10	12
CREB Signaling in Neurons	1.99526E-12	19	11	22
IL-8 Signaling	2.51189E-12	21	10	24

Ephrin Receptor Signaling	3.16228E-12	21	11	22
PI3K Signaling in B Lymphocytes	3.98107E-12	17	10	18
Phospholipase C Signaling	5.01187E-12	20	10	29
Mouse Embryonic Stem Cell Pluripotency	5.01187E-12	5	7	17
Tec Kinase Signaling	6.30957E-12	17	9	22
Hypoxia Signaling in the Cardiovascular System	6.30957E-12	7	5	15
Prostate Cancer Signaling	1E-11	13	13	9
Erythropoietin Signaling	1E-11	6	7	13
Glioblastoma Multiforme Signaling	1.99526E-11	16	12	17
Melatonin Signaling	2.51189E-11	10	3	17
G?q Signaling	2.51189E-11	0	0	29
EGF Signaling	3.16228E-11	10	8	10
Ovarian Cancer Signaling	3.98107E-11	7	10	17
PPAR?/RXR? Activation	3.98107E-11	0	0	32
Neuregulin Signaling	5.01187E-11	17	11	11
Breast Cancer Regulation by Stathmin1	5.01187E-11	24	8	25
eNOS Signaling	5.01187E-11	15	9	19
HGF Signaling	5.01187E-11	11	5	19
Pancreatic Adenocarcinoma Signaling	6.30957E-11	20	8	16
Xenobiotic Metabolism Signaling	6.30957E-11	17	8	32
Type II Diabetes Mellitus Signaling	1E-10	15	8	17
Factors Promoting Cardiogenesis in Vertebrates	1.25893E-10	4	6	16
nNOS Signaling in Neurons	1.38038E-10	2	3	13
Growth Hormone Signaling	1.69824E-10	4	7	12
Wnt/?-catenin Signaling	1.7378E-10	0	0	30
NRF2-mediated Oxidative Stress Response	1.90546E-10	12	7	24
Thrombopoietin Signaling	2.0893E-10	5	4	13
Colorectal Cancer Metastasis Signaling	2.34423E-10	24	12	24
AMPK Signaling	3.89045E-10	12	12	14
ILK Signaling	4.46684E-10	18	15	16
Gap Junction Signaling	4.67735E-10	21	7	21
LPS-stimulated MAPK Signaling	4.89779E-10	9	5	14
Thrombin Signaling	8.70964E-10	23	10	21
Corticotropin Releasing Hormone Signaling	1.02329E-09	18	7	16
PDGF Signaling	1.31826E-09	6	6	13
CXCR4 Signaling	1.38038E-09	21	7	20
Estrogen-Dependent Breast Cancer Signaling	1.65959E-09	3	8	9
Cyclins and Cell Cycle Regulation	1.65959E-09	8	3	16
VDR/RXR Activation	1.65959E-09	9	2	17
Adipogenesis pathway	1.90546E-09	10	2	22

Insulin Receptor Signaling	1.94984E-09	18	14	11
IL-3 Signaling	2.18776E-09	11	4	14
Sertoli Cell-Sertoli Cell Junction Signaling	2.81838E-09	22	9	20
Calcium-induced T Lymphocyte Apoptosis	2.81838E-09	7	5	12
Cell Cycle: G1/S Checkpoint Regulation	2.81838E-09	9	2	15
IL-17A Signaling in Airway Cells	2.81838E-09	4	7	10
Virus Entry via Endocytic Pathways	2.81838E-09	6	6	14
p53 Signaling	2.88403E-09	12	11	10
Nitric Oxide Signaling in the Cardiovascular System	3.46737E-09	9	4	17
Natural Killer Cell Signaling	4.7863E-09	8	7	15
Cholecystokinin/Gastrin-mediated Signaling	5.12861E-09	10	4	17
ATM Signaling	6.0256E-09	10	5	11
Role of Osteoblasts, Osteoclasts and Chondrocytes in Rheumatoid Arthritis	6.91831E-09	22	10	22
VEGF Family Ligand-Receptor Interactions	7.24436E-09	6	4	14
Hereditary Breast Cancer Signaling	1.12202E-08	7	6	16
Calcium Signaling	1.14815E-08	11	4	24
?-Adrenergic Signaling	1.1749E-08	0	0	19
NGF Signaling	1.51356E-08	13	10	11
Renal Cell Carcinoma Signaling	1.58489E-08	6	7	10
Small Cell Lung Cancer Signaling	1.58489E-08	8	5	12
Chemokine Signaling	1.58489E-08	7	4	13
Endothelin-1 Signaling	1.94984E-08	22	6	21
Telomerase Signaling	1.99526E-08	9	7	13
Aryl Hydrocarbon Receptor Signaling	2.34423E-08	15	5	19
CD40 Signaling	2.69153E-08	6	3	13
Chronic Myeloid Leukemia Signaling	3.71535E-08	13	4	15
Cardiac Hypertrophy Signaling	3.89045E-08	23	13	18
Amyloid Processing	4.46684E-08	5	5	9
IL-12 Signaling and Production in Macrophages	5.12861E-08	15	6	17
ErbB4 Signaling	5.88844E-08	9	5	10
TGF-? Signaling	6.76083E-08	0	0	18
CCR3 Signaling in Eosinophils	7.76247E-08	11	7	14
RANK Signaling in Osteoclasts	8.31764E-08	12	6	12
UVA-Induced MAPK Signaling	8.31764E-08	7	7	11
CD28 Signaling in T Helper Cells	8.91251E-08	14	8	13
fMLP Signaling in Neutrophils	9.12011E-08	11	6	14
Sperm Motility	1.20226E-07	10	3	18
Role of Tissue Factor in Cancer	1.25893E-07	14	12	8
Cell Cycle: G2/M DNA Damage Checkpoint Regulation	2.13796E-07	7	7	6
Epithelial Adherens Junction Signaling	2.23872E-07	12	6	17

Myc Mediated Apoptosis Signaling	2.63027E-07	6	8	6
G-Protein Coupled Receptor Signaling	2.81838E-07	30	10	22
Role of NFAT in Regulation of the Immune Response	2.95121E-07	23	10	15
Macropinocytosis Signaling	3.46737E-07	6	5	10
PTEN Signaling	4.16869E-07	24	14	6
Leukocyte Extravasation Signaling	4.2658E-07	16	7	20
Regulation of IL-2 Expression in Activated and Anergic T Lymphocytes	4.89779E-07	13	5	11
Synaptic Long Term Depression	4.89779E-07	16	6	16
mTOR Signaling	5.24807E-07	15	11	15
Semaphorin Signaling in Neurons	5.7544E-07	7	3	10
IL-1 Signaling	7.4131E-07	15	4	13
IL-17 Signaling	7.58578E-07	3	5	10
HIF1? Signaling	8.31764E-07	0	0	18
Leptin Signaling in Obesity	1.09648E-06	8	4	11
Non-Small Cell Lung Cancer Signaling	1.1749E-06	11	5	9
Regulation of the Epithelial-Mesenchymal Transition Pathway	1.20226E-06	17	7	18
NF-?B Signaling	1.34896E-06	0	0	24
IL-6 Signaling	1.38038E-06	14	7	12
IL-15 Signaling	1.41254E-06	5	6	8
DNA Methylation and Transcriptional Repression Signaling	1.47911E-06	0	1	7
G?12/13 Signaling	1.54882E-06	0	0	19
HIPPO signaling	1.62181E-06	13	8	8
Aldosterone Signaling in Epithelial Cells	1.8197E-06	9	6	16
Protein Ubiquitination Pathway	2.39883E-06	13	6	24
Apoptosis Signaling	2.63027E-06	14	5	11
iNOS Signaling	3.46737E-06	5	5	6
GM-CSF Signaling	3.89045E-06	8	4	9
IL-2 Signaling	3.89045E-06	4	3	9
JAK/Stat Signaling	4.2658E-06	8	6	8
NF-?B Activation by Viruses	5.01187E-06	0	0	14
Estrogen Receptor Signaling	5.49541E-06	7	4	15
Pyridoxal 5'-phosphate Salvage Pathway	5.62341E-06	9	0	13
April Mediated Signaling	5.88844E-06	4	2	8
Integrin Signaling	6.60693E-06	14	9	16
Wnt/Ca+ pathway	7.07946E-06	3	4	8
PKC? Signaling in T Lymphocytes	7.24436E-06	0	0	18
IL-22 Signaling	7.4131E-06	3	2	6
T Cell Receptor Signaling	8.31764E-06	11	5	11
Antioxidant Action of Vitamin C	8.31764E-06	7	4	12
ErbB2-ErbB3 Signaling	8.70964E-06	8	6	6

Glutamate Receptor Signaling	8.70964E-06	1	7	5
B Cell Activating Factor Signaling	9.77237E-06	4	2	8
Acute Phase Response Signaling	1.04713E-05	21	7	15
IL-17A Signaling in Gastric Cells	1.04713E-05	2	2	6
Cardiac ?-adrenergic Signaling	1.09648E-05	0	0	19
GDNF Family Ligand-Receptor Interactions	1.12202E-05	9	4	9
Role of MAPK Signaling in the Pathogenesis of Influenza	1.12202E-05	7	4	9
Role of NANOG in Mammalian Embryonic Stem Cell Pluripotency	1.23027E-05	5	6	11
Mechanisms of Viral Exit from Host Cells	1.23027E-05	2	2	8
MIF Regulation of Innate Immunity	1.23027E-05	2	0	10
Germ Cell-Sertoli Cell Junction Signaling	1.44544E-05	18	8	13
Inhibition of Angiogenesis by TSP1	1.62181E-05	6	4	5
Hepatic Cholestasis	1.77828E-05	15	3	18
Rac Signaling	2.0893E-05	11	4	12
IL-17A Signaling in Fibroblasts	2.0893E-05	3	3	6
ERK5 Signaling	2.51189E-05	10	10	2
Role of JAK1 and JAK3 in ?c Cytokine Signaling	2.51189E-05	0	0	12
Melanocyte Development and Pigmentation Signaling	2.69153E-05	9	7	7
Tight Junction Signaling	2.81838E-05	12	8	13
Lymphotoxin ? Receptor Signaling	2.81838E-05	0	0	11
Signaling by Rho Family GTPases	3.01995E-05	20	7	19
HMGB1 Signaling	3.46737E-05	10	5	12
BMP signaling pathway	3.89045E-05	8	2	11
FAK Signaling	4.0738E-05	7	9	5
Clathrin-mediated Endocytosis Signaling	4.36516E-05	17	5	17
Acute Myeloid Leukemia Signaling	4.46684E-05	9	3	10
CDK5 Signaling	4.46684E-05	10	3	12
CTLA4 Signaling in Cytotoxic T Lymphocytes	4.57088E-05	5	7	7
Relaxin Signaling	4.67735E-05	18	6	12
Neurotrophin/TRK Signaling	4.7863E-05	10	7	5
Docosahexaenoic Acid (DHA) Signaling	5.37032E-05	5	8	1
Reelin Signaling in Neurons	5.88844E-05	11	7	6
IL-10 Signaling	6.45654E-05	6	2	10
Role of Pattern Recognition Receptors in Recognition of Bacteria and Viruses	7.24436E-05	7	2	15
Fc?RIIB Signaling in B Lymphocytes	8.12831E-05	0	0	9
PPAR Signaling	9.54993E-05	11	3	11
G Protein Signaling Mediated by Tubby	9.54993E-05	2	4	4
Role of JAK family kinases in IL-6-type Cytokine Signaling	9.77237E-05	3	2	5
Melanoma Signaling	0.0001	5	4	5
Endometrial Cancer Signaling	0.000109648	8	6	4

Salvage Pathways of Pyrimidine Ribonucleotides	0.000109648	10	0	14
Ephrin B Signaling	0.000114815	12	4	8
iCOS-iCOSL Signaling in T Helper Cells	0.000123027	13	7	8
GADD45 Signaling	0.000151356	2	1	5
DNA damage-induced 14-3-3? Signaling	0.000151356	0	0	6
IL-4 Signaling	0.000169824	4	6	6
Dendritic Cell Maturation	0.000223872	12	8	12
Nur77 Signaling in T Lymphocytes	0.000239883	5	5	5
Cdc42 Signaling	0.000251189	10	5	14
Remodeling of Epithelial Adherens Junctions	0.000251189	4	2	9
VEGF Signaling	0.00025704	13	5	8
Ceramide Signaling	0.000281838	9	6	6
p38 MAPK Signaling	0.000301995	15	5	10
TNFR1 Signaling	0.000346737	12	4	5
SAPK/JNK Signaling	0.000363078	8	5	8
PEDF Signaling	0.000371535	7	5	6
RhoGDI Signaling	0.000398107	17	9	10
Role of PKR in Interferon Induction and Antiviral Response	0.000398107	8	4	4
4-1BB Signaling in T Lymphocytes	0.00042658	5	2	5
Human Embryonic Stem Cell Pluripotency	0.000436516	12	7	9
G?s Signaling	0.000467735	0	0	14
Prostanoid Biosynthesis	0.000467735	0	0	4
STAT3 Pathway	0.00047863	10	5	6
FGF Signaling	0.000489779	10	6	6
Type I Diabetes Mellitus Signaling	0.000512861	17	6	8
FLT3 Signaling in Hematopoietic Progenitor Cells	0.000537032	10	8	3
Amyotrophic Lateral Sclerosis Signaling	0.000537032	9	4	9
CD27 Signaling in Lymphocytes	0.000549541	9	2	7
PAK Signaling	0.000758578	8	4	8
Oncostatin M Signaling	0.000776247	4	1	6
IL-9 Signaling	0.000776247	2	2	5
Role of IL-17F in Allergic Inflammatory Airway Diseases	0.000794328	3	3	5
cAMP-mediated signaling	0.001202264	24	6	15
IL-15 Production	0.001202264	3	2	4
RhoA Signaling	0.00144544	8	2	12
Sphingosine-1-phosphate Signaling	0.001479108	13	4	9
Toll-like Receptor Signaling	0.001995262	7	2	8
Spliceosomal Cycle	0.002089296	0	0	2
Role of p14/p19ARF in Tumor Suppression	0.002137962	1	2	4
PCP pathway	0.002238721	4	1	8

CNTF Signaling	0.002398833	5	5	3
Cellular Effects of Sildenafil (Viagra)	0.002454709	11	2	12
Paxillin Signaling	0.002511886	9	5	7
Unfolded protein response	0.003090295	8	1	7
DNA Double-Strand Break Repair by Non-Homologous End Joining	0.003090295	1	0	4
Death Receptor Signaling	0.003311311	15	5	6
Circadian Rhythm Signaling	0.003548134	2	2	4
Telomere Extension by Telomerase	0.004073803	2	0	4
Agrin Interactions at Neuromuscular Junction	0.004168694	10	4	5
Estrogen-mediated S-phase Entry	0.004168694	6	1	4
Regulation of Cellular Mechanics by Calpain Protease	0.004365158	5	2	6
Phospholipases	0.004365158	3	1	7
Parkinson's Signaling	0.005128614	3	2	2
Actin Cytoskeleton Signaling	0.005495409	13	3	16
Induction of Apoptosis by HIV1	0.005888437	13	3	5
Ephrin A Signaling	0.006025596	3	3	4
Systemic Lupus Erythematosus Signaling	0.006309573	8	4	15
TREM1 Signaling	0.00724436	5	3	6
Regulation of eIF4 and p70S6K Signaling	0.007413102	9	9	5
Netrin Signaling	0.008317638	3	0	6
Dopamine Receptor Signaling	0.009332543	13	2	7
Thyroid Cancer Signaling	0.009332543	5	1	5
G?i Signaling	0.009332543	0	0	12
TNFR2 Signaling	0.009549926	8	1	4
