# Supplementary figures and images for: Schizophrenia interactome with 504 novel protein–protein interactions
Source: NPJ Schizophr. 2016 Apr 27;2:16012–. doi: 10.1038/npjschz.2016.12 (PMC4898894; doi:10.1038/npjschz.2016.12)

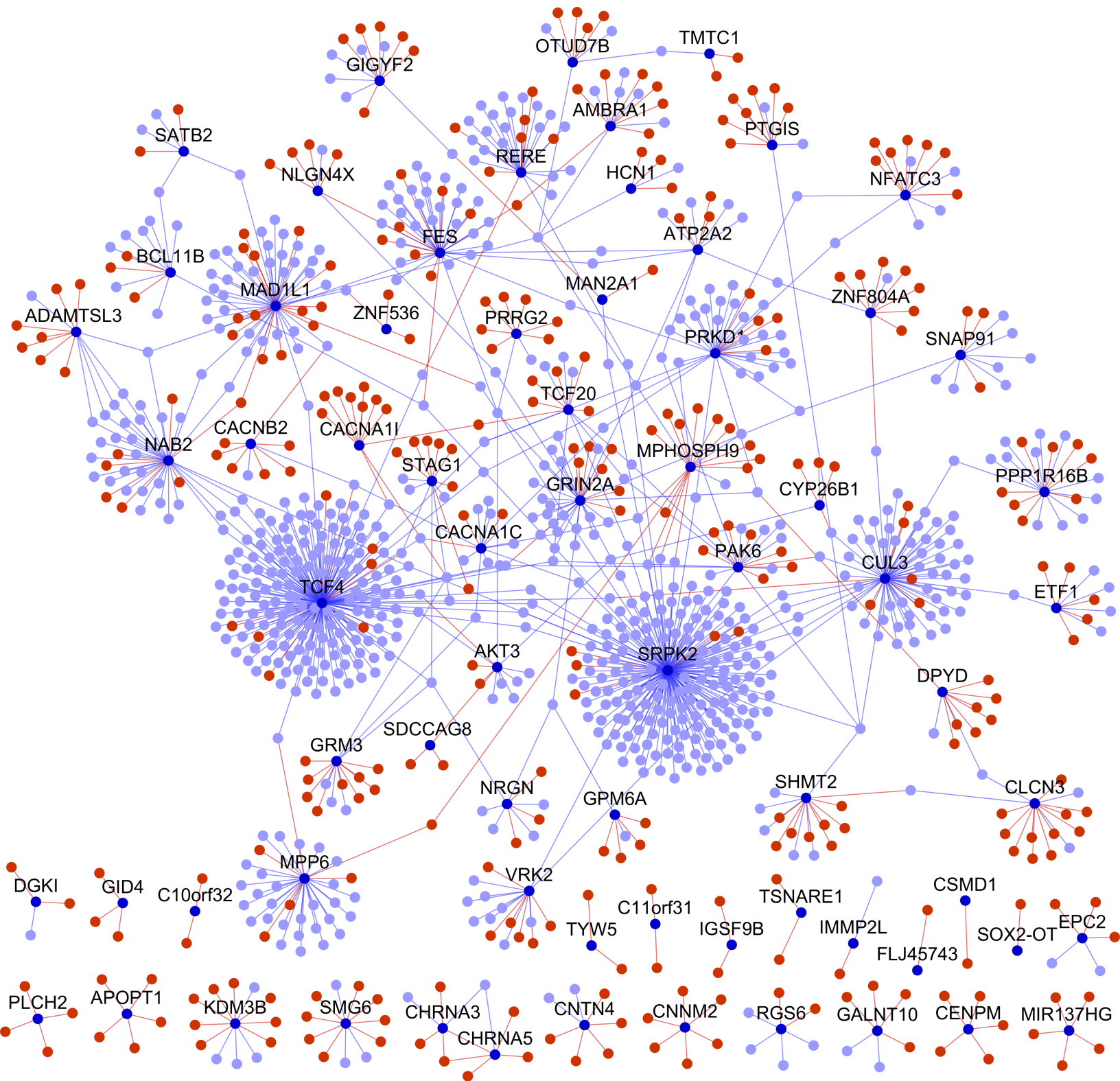

Supplement: Supplementary File 5 [file npjschz201612-s5.pdf]

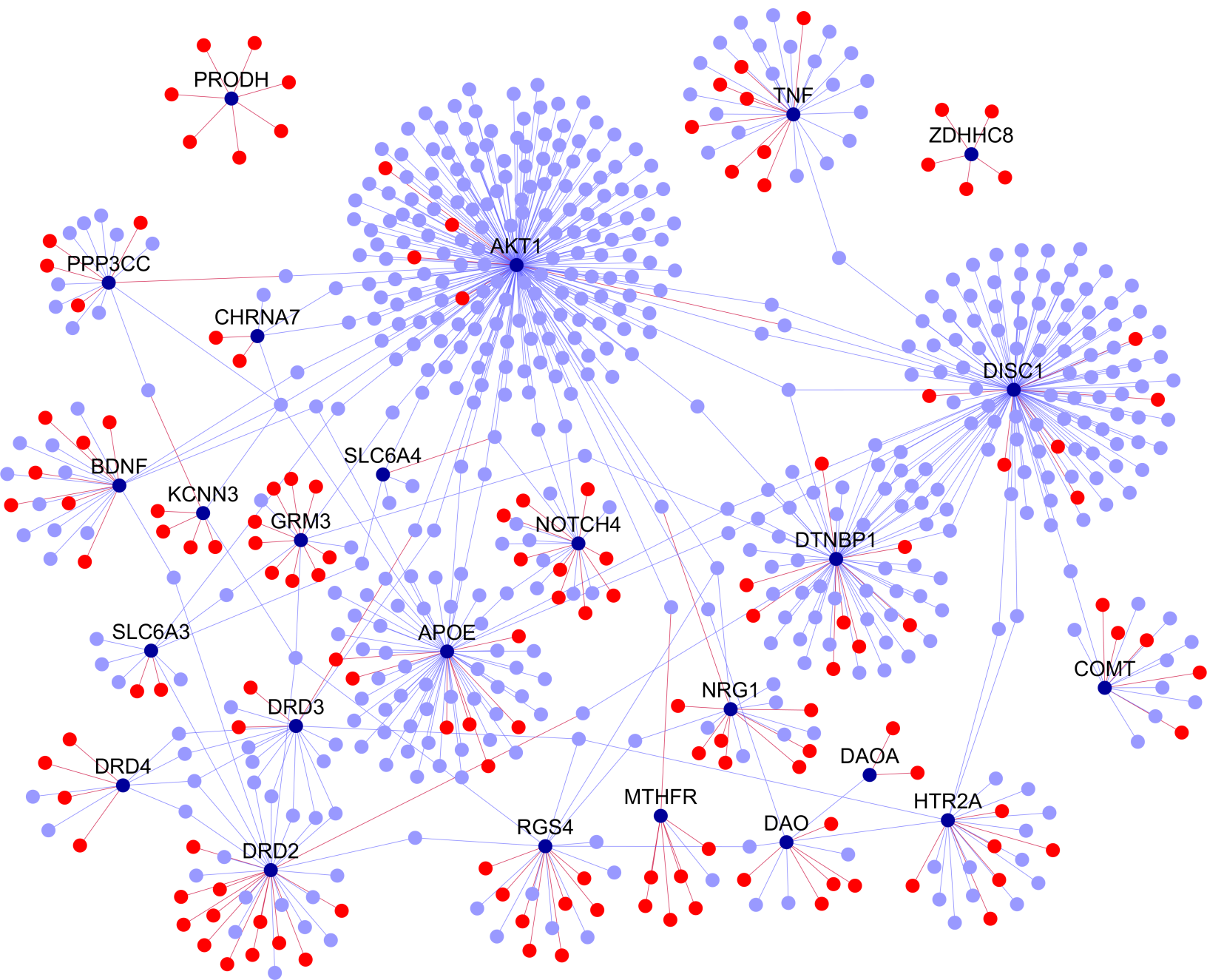

Supplement: Supplementary File 6 [file npjschz201612-s6.pdf]

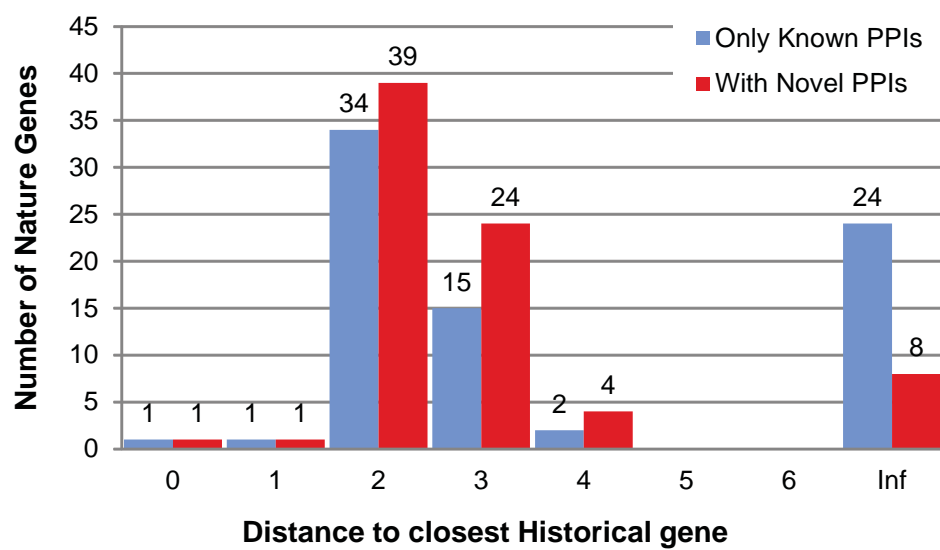

Supplement: Supplementary File 7 [file npjschz201612-s7.pdf]

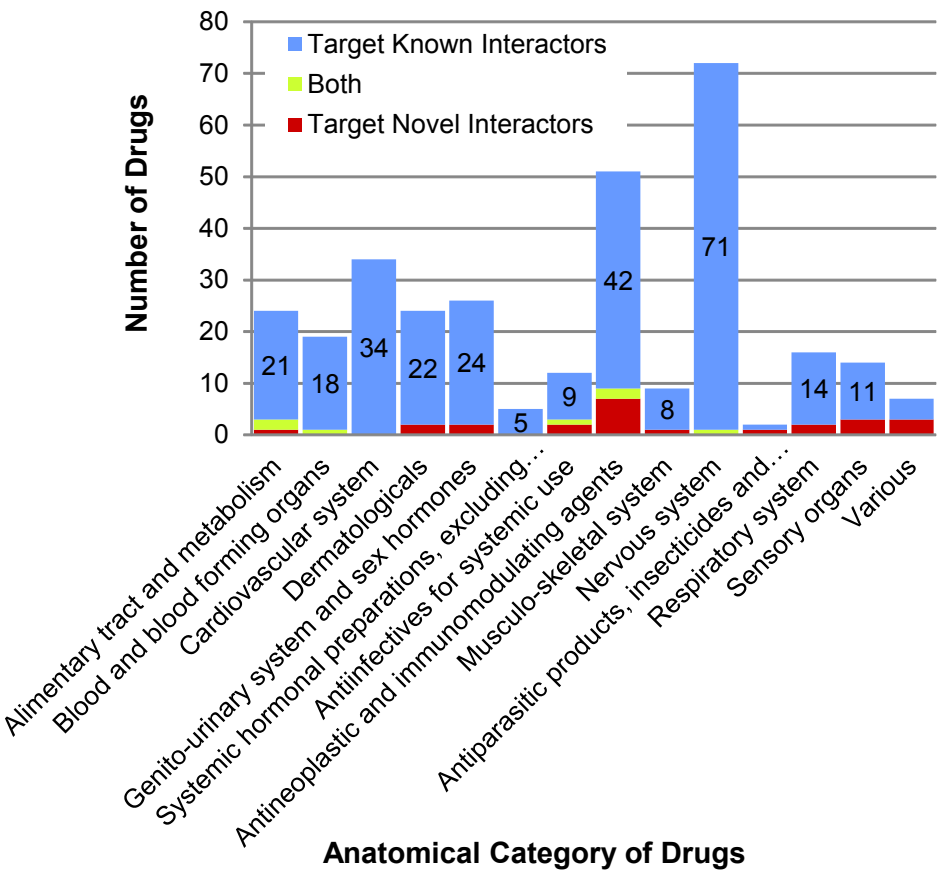

Supplement: Supplementary File 9 [file npjschz201612-s9.pdf]

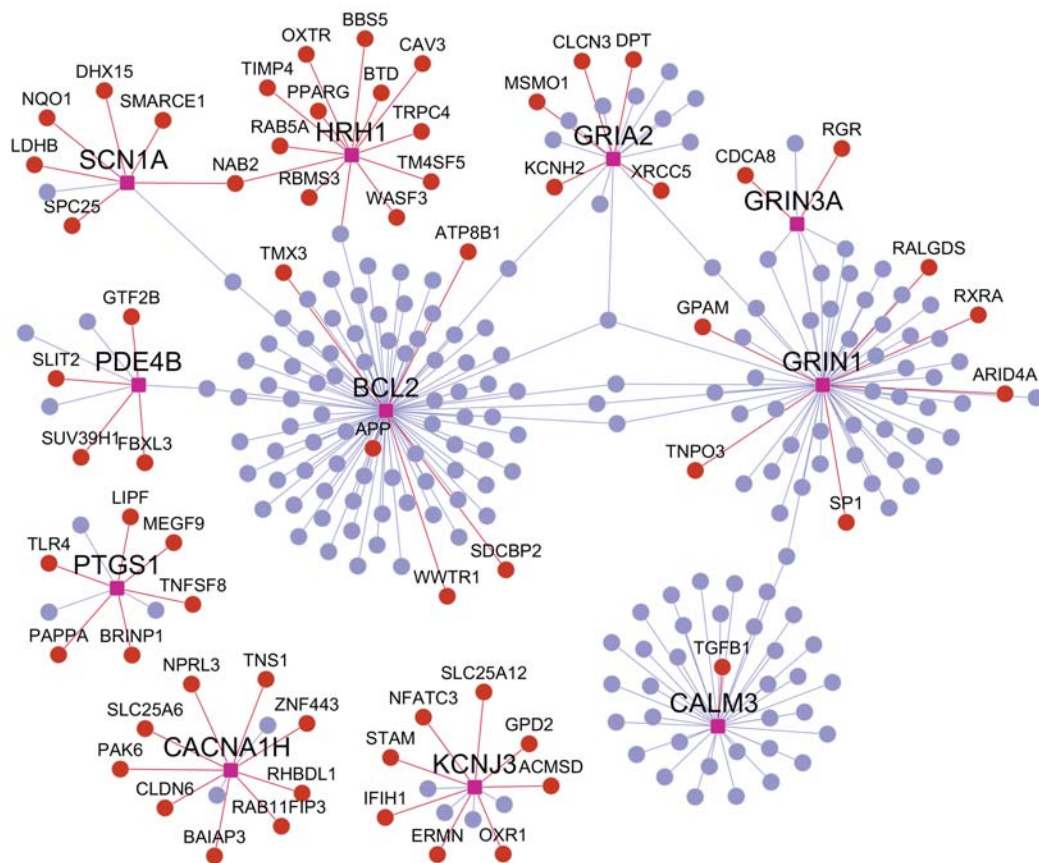

Supplement: Supplementary File 11 [file npjschz201612-s11.pdf]
