# Supplementary material for: Schizophrenia interactome with 504 novel protein–protein interactions
Source: NPJ Schizophr. 2016 Apr 27;2:16012–. doi: 10.1038/npjschz.2016.12 (PMC4898894; doi:10.1038/npjschz.2016.12)
Supplement: Supplementary File 8 [file npjschz201612-s8.pdf]

Genes from the interactome that are found to overlap with proteomics studies[1]

GWAS/Historical Genes: DISC1

Known Interactors: ACTG1, ATP6V1B2, CALM1, CALM2, CAMK2B, DPYSL2, GLUL, GNB1, IL16, MAPK3, NEFL, NEFM, PGK1, PRDX2, TPM1, TUBB2A

Novel Interactors: None

Genes from the interactome that are found to be differentially expressed in grey and white matter of Schizophrenia postmortem brains[2]

GWAS/Historical Genes in Grey Matter: None

Known interactors present in Grey matter: ATP6V1B2, CALM1, CALM2, CALM3, CTBP1, DCTN2, DPYSL2, DPYSL3, EIF3F, FKBP4, GLUL, GNAI1, GNAI3, GNB1, IMMT, MAX, NCAM1, NDRG1, NEFL, NSF, PPIA, PPP1CA, PPP2R1A, PRDX2, PRNP, SNCA, SPTAN1, SRI, TCP1, TUBA1B, TUBB, YWHAE, YWHAQ, YWHAZ

Novel interactors present in Grey matter: ANXA5, BYSL, CD59, IDH3A, PDIA3, TUBA1C, TUFM

GWAS/Historical Genes in White Matter: None

Known interactors present in White matter: ACTG1, ALB, ATP6V1B2, DPYSL2, DPYSL5, IMPDH2, NEFL, NEFM, PCMT1, PRDX2, SPTAN1, STX1A, TUBB, TUBB2A, VIM, YWHAE, YWHAG, YWHAZ

Novel interactors present in White matter: ACTR1A, ANXA5, FSCN1

References

1 Nascimento, J. M. & Martins-de-Souza, D. The proteome of schizophrenia. *npj Schizophrenia* **1** (2015).

2 English, J. A., Pennington, K., Dunn, M. J. & Cotter, D. R. The neuroproteomics of schizophrenia. *Biological psychiatry* **69**, 163-172, doi:10.1016/j.biopsych.2010.06.031 (2011).
