# Supplementary material for: Schizophrenia interactome with 504 novel protein–protein interactions
Source: NPJ Schizophr. 2016 Apr 27;2:16012–. doi: 10.1038/npjschz.2016.12 (PMC4898894; doi:10.1038/npjschz.2016.12)
Supplement: Supplementary File 10 [file npjschz201612-s10.pdf]

Drugs of GWAS Gene Interactome

Gene Type	EntrezID	Gene Symbol	DrugBank ID	Drug Name	Category Anatomic	Category Therapeutic	Category Chemical
Candidate Gene	775	CACNA1C	DB01373	Calcium	A	2	AC01
Candidate Gene	775	CACNA1C	DB01373	Calcium	A	2	AC02
Candidate Gene	775	CACNA1C	DB06751	Drotaverine	A	3	AD02
Candidate Gene	775	CACNA1C	DB00653	Magnesium Sulfate	A	6	AD04
Candidate Gene	775	CACNA1C	DB01373	Calcium	A	6	AC08
Candidate Gene	775	CACNA1C	DB01373	Calcium	A	7	XA03
Candidate Gene	775	CACNA1C	DB01373	Calcium	A	11	HA31
Candidate Gene	775	CACNA1C	DB00653	Magnesium Sulfate	A	12	CC02
Candidate Gene	775	CACNA1C	DB01373	Calcium	A	12	AA01
Candidate Gene	775	CACNA1C	DB01373	Calcium	A	12	AA02
Candidate Gene	775	CACNA1C	DB01373	Calcium	A	12	AA03
Candidate Gene	775	CACNA1C	DB01373	Calcium	A	12	AA04
Candidate Gene	775	CACNA1C	DB01373	Calcium	A	12	AA05
Candidate Gene	775	CACNA1C	DB01373	Calcium	A	12	AA06
Candidate Gene	775	CACNA1C	DB01373	Calcium	A	12	AA08
Candidate Gene	775	CACNA1C	DB01373	Calcium	A	12	AA09
Candidate Gene	775	CACNA1C	DB01373	Calcium	A	12	AA10
Candidate Gene	775	CACNA1C	DB01373	Calcium	A	12	AA11
Candidate Gene	775	CACNA1C	DB01373	Calcium	A	12	AA20
Candidate Gene	775	CACNA1C	DB01373	Calcium	A	12	AA30
Candidate Gene	783	CACNB2	DB00653	Magnesium Sulfate	A	6	AD04
Candidate Gene	783	CACNB2	DB00653	Magnesium Sulfate	A	12	CC02
Candidate Gene	2903	GRIN2A	DB00142	L-Glutamic Acid	A	9	AB01
Candidate Gene	2903	GRIN2A	DB06593	Dehydroepiandrosterone	A	14	AA07
Candidate Gene	6472	SHMT2	DB00114	Pyridoxal Phosphate	A	11	HA02
Candidate Gene	6472	SHMT2	DB00114	Pyridoxal Phosphate	A	11	HA06
Candidate Gene	775	CACNA1C	DB01373	Calcium	B	1	AC08
Candidate Gene	775	CACNA1C	DB01373	Calcium	B	2	BC08
Candidate Gene	775	CACNA1C	DB00653	Magnesium Sulfate	B	5	XA05
Candidate Gene	783	CACNB2	DB00653	Magnesium Sulfate	B	5	XA05

Candidate Gene	2903	GRIN2A	DB00145	Glycine	B	3	AA01
Candidate Gene	2903	GRIN2A	DB00145	Glycine	B	5	CX03
Candidate Gene	5740	PTGIS	DB01240	Epoprostenol	B	1	AC09
Candidate Gene	6472	SHMT2	DB00145	Glycine	B	3	AA01
Candidate Gene	6472	SHMT2	DB00145	Glycine	B	5	CX03
Candidate Gene	775	CACNA1C	DB00308	Ibutilide	C	1	BD05
Candidate Gene	775	CACNA1C	DB04855	Dronedarone	C	1	BD07
Candidate Gene	775	CACNA1C	DB00421	Spironolactone	C	3	DA01
Candidate Gene	775	CACNA1C	DB01373	Calcium	C	5	BX01
Candidate Gene	775	CACNA1C	DB00270	Isradipine	C	8	CA03
Candidate Gene	775	CACNA1C	DB00381	Amlodipine	C	8	CA01
Candidate Gene	775	CACNA1C	DB00393	Nimodipine	C	8	CA06
Candidate Gene	775	CACNA1C	DB00401	Nisoldipine	C	8	CA07
Candidate Gene	775	CACNA1C	DB00622	Nicardipine	C	8	CA04
Candidate Gene	775	CACNA1C	DB00661	Verapamil	C	8	DA01
Candidate Gene	775	CACNA1C	DB01023	Felodipine	C	8	CA02
Candidate Gene	775	CACNA1C	DB01054	Nitrendipine	C	8	CA08
Candidate Gene	775	CACNA1C	DB01115	Nifedipine	C	8	CA05
Candidate Gene	775	CACNA1C	DB01388	Mibefradil	C	8	CX01
Candidate Gene	775	CACNA1C	DB06712	Nilvadipine	C	8	CA10
Candidate Gene	8911	CACNA1I	DB00421	Spironolactone	C	3	DA01
Candidate Gene	8911	CACNA1I	DB00661	Verapamil	C	8	DA01
Candidate Gene	8911	CACNA1I	DB01388	Mibefradil	C	8	CX01
Candidate Gene	783	CACNB2	DB04855	Dronedarone	C	1	BD07
Candidate Gene	783	CACNB2	DB00421	Spironolactone	C	3	DA01
Candidate Gene	783	CACNB2	DB00270	Isradipine	C	8	CA03
Candidate Gene	783	CACNB2	DB00381	Amlodipine	C	8	CA01
Candidate Gene	783	CACNB2	DB00393	Nimodipine	C	8	CA06
Candidate Gene	783	CACNB2	DB00401	Nisoldipine	C	8	CA07
Candidate Gene	783	CACNB2	DB00622	Nicardipine	C	8	CA04
Candidate Gene	783	CACNB2	DB00661	Verapamil	C	8	DA01
Candidate Gene	783	CACNB2	DB01023	Felodipine	C	8	CA02
Candidate Gene	783	CACNB2	DB01054	Nitrendipine	C	8	CA08
Candidate Gene	783	CACNB2	DB01115	Nifedipine	C	8	CA05

Candidate Gene	783	CACNB2	DB01388	Mibefradil	C	8	CX01
Candidate Gene	783	CACNB2	DB06712	Nilvadipine	C	8	CA10
Candidate Gene	775	CACNA1C	DB01373	Calcium	D	3	AX04
Candidate Gene	775	CACNA1C	DB00653	Magnesium Sulfate	D	11	AX05
Candidate Gene	775	CACNA1C	DB01373	Calcium	D	11	AX03
Candidate Gene	783	CACNB2	DB00653	Magnesium Sulfate	D	11	AX05
Candidate Gene	775	CACNA1C	DB01373	Calcium	J	4	AA03
Candidate Gene	5740	PTGIS	DB08343	Phenylbutazone	M	1	AA01
Candidate Gene	5740	PTGIS	DB08343	Phenylbutazone	M	2	AA01
Candidate Gene	775	CACNA1C	DB01373	Calcium	N	2	BA15
Candidate Gene	775	CACNA1C	DB00568	Cinnarizine	N	7	CA02
Candidate Gene	775	CACNA1C	DB01373	Calcium	N	7	BB02
Candidate Gene	8911	CACNA1I	DB00617	Paramethadione	N	3	AC01
Candidate Gene	8911	CACNA1I	DB00909	Zonisamide	N	3	AX15
Candidate Gene	8911	CACNA1I	DB00568	Cinnarizine	N	7	CA02
Candidate Gene	8911	CACNA1I	DB04841	Flunarizine	N	7	CA03
Candidate Gene	2903	GRIN2A	DB02330	Halothane	N	1	AB01
Candidate Gene	2903	GRIN2A	DB00454	Pethidine	N	2	AB02
Candidate Gene	2903	GRIN2A	DB06738	Ketobemidone	N	2	AB01
Candidate Gene	2903	GRIN2A	DB00949	Felbamate	N	3	AX10
Candidate Gene	2903	GRIN2A	DB00996	Gabapentin	N	3	AX12
Candidate Gene	2903	GRIN2A	DB01174	Phenobarbital	N	3	AA02
Candidate Gene	2903	GRIN2A	DB00312	Pentobarbital	N	5	CA01
Candidate Gene	2903	GRIN2A	DB00418	Secobarbital	N	5	CA06
Candidate Gene	2903	GRIN2A	DB00289	Atomoxetine	N	6	BA09
Candidate Gene	2903	GRIN2A	DB01043	Memantine	N	6	DX01
Candidate Gene	2903	GRIN2A	DB04896	Milnacipran	N	6	AX17
Candidate Gene	2903	GRIN2A	DB00659	Acamprosate	N	7	BB03
Candidate Gene	775	CACNA1C	DB01373	Calcium	R	1	AX01
Candidate Gene	775	CACNA1C	DB01373	Calcium	V	3	AE01
Candidate Gene	775	CACNA1C	DB01373	Calcium	V	3	AF04
Candidate Gene	775	CACNA1C	DB00653	Magnesium Sulfate	V	4	CC02
Candidate Gene	775	CACNA1C	DB01373	Calcium	V	8	AC10
Candidate Gene	783	CACNB2	DB00653	Magnesium Sulfate	V	4	CC02

Known Interactor	240	ALOX5	DB01017	Minocycline	A	1	AB23
Known Interactor	1956	EGFR	DB05291	Lidocaine	A	1	AD11
Known Interactor	4790	NFKB1	DB00945	Acetylsalicylic acid	A	1	AD05
Known Interactor	5562	PRKAA1	DB00945	Acetylsalicylic acid	A	1	AD05
Known Interactor	5565	PRKAB2	DB00945	Acetylsalicylic acid	A	1	AD05
Known Interactor	5742	PTGS1	DB00945	Acetylsalicylic acid	A	1	AD05
Known Interactor	5743	PTGS2	DB00945	Acetylsalicylic acid	A	1	AD05
Known Interactor	7157	TP53	DB00945	Acetylsalicylic acid	A	1	AD05
Known Interactor	801	CALM1	DB01373	Calcium	A	2	AC01
Known Interactor	801	CALM1	DB01373	Calcium	A	2	AC02
Known Interactor	5733	PTGER3	DB00929	Misoprostol	A	2	BB01
Known Interactor	801	CALM1	DB01373	Calcium	A	6	AC08
Known Interactor	240	ALOX5	DB00244	Mesalazine	A	7	EC02
Known Interactor	240	ALOX5	DB08518	Sulfasalazine	A	7	EC01
Known Interactor	240	ALOX5	DB01014	Balsalazide	A	7	EC04
Known Interactor	801	CALM1	DB00836	Loperamide	A	7	DA03
Known Interactor	801	CALM1	DB00836	Loperamide	A	7	DA05
Known Interactor	801	CALM1	DB01373	Calcium	A	7	XA03
Known Interactor	5742	PTGS1	DB00244	Mesalazine	A	7	EC02
Known Interactor	5742	PTGS1	DB08518	Sulfasalazine	A	7	EC01
Known Interactor	5742	PTGS1	DB01014	Balsalazide	A	7	EC04
Known Interactor	5743	PTGS2	DB00244	Mesalazine	A	7	EC02
Known Interactor	5743	PTGS2	DB08518	Sulfasalazine	A	7	EC01
Known Interactor	5743	PTGS2	DB01014	Balsalazide	A	7	EC04
Known Interactor	2902	GRIN1	DB00142	L-Glutamic Acid	A	9	AB01
Known Interactor	116443	GRIN3A	DB00142	L-Glutamic Acid	A	9	AB01
Known Interactor	116444	GRIN3B	DB00142	L-Glutamic Acid	A	9	AB01
Known Interactor	19	ABCA1	DB01016	Glyburide	A	10	BB01
Known Interactor	1080	CFTR	DB01016	Glyburide	A	10	BB01
Known Interactor	2740	GLP1R	DB01276	Exenatide	A	10	BX04
Known Interactor	2740	GLP1R	DB06655	Liraglutide	A	10	BX07
Known Interactor	3480	IGF1R	DB01383	Insulin Regular	A	10	AB01
Known Interactor	3480	IGF1R	DB01383	Insulin Regular	A	10	AB03
Known Interactor	3480	IGF1R	DB01383	Insulin Regular	A	10	AB04

Known Interactor	3480	IGF1R	DB01383	Insulin Regular	A	10	AB05
Known Interactor	3480	IGF1R	DB01383	Insulin Regular	A	10	AC01
Known Interactor	3480	IGF1R	DB01383	Insulin Regular	A	10	AC03
Known Interactor	3480	IGF1R	DB01383	Insulin Regular	A	10	AD05
Known Interactor	3480	IGF1R	DB01383	Insulin Regular	A	10	AE04
Known Interactor	3480	IGF1R	DB01383	Insulin Regular	A	10	AE05
Known Interactor	3480	IGF1R	DB01310	Insulin Lispro	A	10	AC04
Known Interactor	3480	IGF1R	DB01310	Insulin Lispro	A	10	AD04
Known Interactor	5562	PRKAA1	DB00914	Phenformin	A	10	BA01
Known Interactor	221120	ALKBH3	DB00126	Vitamin C	A	11	GA01
Known Interactor	240	ALOX5	DB00163	Vitamin E	A	11	HA03
Known Interactor	801	CALM1	DB01373	Calcium	A	11	HA31
Known Interactor	112398	EGLN2	DB00126	Vitamin C	A	11	GA01
Known Interactor	5515	PPP2CA	DB00163	Vitamin E	A	11	HA03
Known Interactor	5578	PRKCA	DB00163	Vitamin E	A	11	HA03
Known Interactor	5579	PRKCB	DB00163	Vitamin E	A	11	HA03
Known Interactor	284904	SEC14L4	DB00163	Vitamin E	A	11	HA03
Known Interactor	801	CALM1	DB01373	Calcium	A	12	AA01
Known Interactor	801	CALM1	DB01373	Calcium	A	12	AA02
Known Interactor	801	CALM1	DB01373	Calcium	A	12	AA03
Known Interactor	801	CALM1	DB01373	Calcium	A	12	AA04
Known Interactor	801	CALM1	DB01373	Calcium	A	12	AA05
Known Interactor	801	CALM1	DB01373	Calcium	A	12	AA06
Known Interactor	801	CALM1	DB01373	Calcium	A	12	AA08
Known Interactor	801	CALM1	DB01373	Calcium	A	12	AA09
Known Interactor	801	CALM1	DB01373	Calcium	A	12	AA10
Known Interactor	801	CALM1	DB01373	Calcium	A	12	AA11
Known Interactor	801	CALM1	DB01373	Calcium	A	12	AA20
Known Interactor	801	CALM1	DB01373	Calcium	A	12	AA30
Known Interactor	367	AR	DB00621	Oxandrolone	A	14	AA08
Known Interactor	367	AR	DB00984	Nandrolone phenpropionate	A	14	AB01
Known Interactor	367	AR	DB02901	Dihydrotestosterone	A	14	AA01
Known Interactor	2099	ESR1	DB06593	Dehydroepiandrosterone	A	14	AA07
Known Interactor	2902	GRIN1	DB06593	Dehydroepiandrosterone	A	14	AA07

Known Interactor	116443	GRIN3A	DB06593	Dehydroepiandrosterone	A	14	AA07
Known Interactor	116444	GRIN3B	DB06593	Dehydroepiandrosterone	A	14	AA07
Known Interactor	801	CALM1	DB01373	Calcium	B	1	AC08
Known Interactor	2353	FOS	DB08813	Nadroparin	B	1	AB06
Known Interactor	4790	NFKB1	DB00945	Acetylsalicylic acid	B	1	AC06
Known Interactor	4790	NFKB1	DB08814	Triflusal	B	1	AC18
Known Interactor	5467	PPARD	DB00374	Treprostinil	B	1	AC21
Known Interactor	5562	PRKAA1	DB00945	Acetylsalicylic acid	B	1	AC06
Known Interactor	5565	PRKAB2	DB00945	Acetylsalicylic acid	B	1	AC06
Known Interactor	5742	PTGS1	DB00945	Acetylsalicylic acid	B	1	AC06
Known Interactor	5742	PTGS1	DB08814	Triflusal	B	1	AC18
Known Interactor	5743	PTGS2	DB00945	Acetylsalicylic acid	B	1	AC06
Known Interactor	7157	TP53	DB00945	Acetylsalicylic acid	B	1	AC06
Known Interactor	801	CALM1	DB01373	Calcium	B	2	BC08
Known Interactor	116444	GRIN3B	DB00145	Glycine	B	3	AA01
Known Interactor	116444	GRIN3B	DB00145	Glycine	B	5	CX03
Known Interactor	596	BCL2	DB01050	Ibuprofen	C	1	EB16
Known Interactor	784	CACNB3	DB04855	Dronedarone	C	1	BD07
Known Interactor	801	CALM1	DB01429	Aprindine	C	1	BB04
Known Interactor	801	CALM1	DB04825	Prenylamine	C	1	DX02
Known Interactor	1080	CFTR	DB01050	Ibuprofen	C	1	EB16
Known Interactor	1956	EGFR	DB05291	Lidocaine	C	1	BB01
Known Interactor	116443	GRIN3A	DB00721	Procaine	C	1	BA02
Known Interactor	5295	PIK3R1	DB01064	Isoprenaline	C	1	CA02
Known Interactor	5742	PTGS1	DB00328	Indomethacin	C	1	EB03
Known Interactor	5742	PTGS1	DB01050	Ibuprofen	C	1	EB16
Known Interactor	5743	PTGS2	DB00328	Indomethacin	C	1	EB03
Known Interactor	5743	PTGS2	DB01050	Ibuprofen	C	1	EB16
Known Interactor	5742	PTGS1	DB08225	Minoxidil	C	2	DC01
Known Interactor	367	AR	DB00421	Spironolactone	C	3	DA01
Known Interactor	784	CACNB3	DB00421	Spironolactone	C	3	DA01
Known Interactor	1080	CFTR	DB00887	Bumetanide	C	3	CA02
Known Interactor	801	CALM1	DB00925	Phenoxybenzamine	C	4	AX02
Known Interactor	801	CALM1	DB00527	Cinchocaine	C	5	AD04

Known Interactor	801	CALM1	DB01373	Calcium	C	5	BX01
Known Interactor	1956	EGFR	DB05291	Lidocaine	C	5	AD01
Known Interactor	116443	GRIN3A	DB00721	Procaine	C	5	AD05
Known Interactor	784	CACNB3	DB00393	Nimodipine	C	8	CA06
Known Interactor	784	CACNB3	DB00661	Verapamil	C	8	DA01
Known Interactor	784	CACNB3	DB01388	Mibefradil	C	8	CX01
Known Interactor	801	CALM1	DB00622	Nicardipine	C	8	CA04
Known Interactor	801	CALM1	DB01023	Felodipine	C	8	CA02
Known Interactor	801	CALM1	DB01115	Nifedipine	C	8	CA05
Known Interactor	801	CALM1	DB01244	Bepridil	C	8	EA02
Known Interactor	3725	JUN	DB01029	Irbesartan	C	9	CA04
Known Interactor	19	ABCA1	DB01599	Probucol	C	10	AX02
Known Interactor	3066	HDAC2	DB00227	Lovastatin	C	10	AA02
Known Interactor	5467	PPARD	DB00159	Icosapent	C	10	AX06
Known Interactor	5467	PPARD	DB08380	Bezafibrate	C	10	AB02
Known Interactor	5742	PTGS1	DB00159	Icosapent	C	10	AX06
Known Interactor	5743	PTGS2	DB00159	Icosapent	C	10	AX06
Known Interactor	367	AR	DB01026	Ketoconazole	D	1	AC08
Known Interactor	1786	DNMT1	DB01099	Flucytosine	D	1	AE21
Known Interactor	116443	GRIN3A	DB00898	Ethanol	D	1	AE06
Known Interactor	5742	PTGS1	DB01398	Salicylate-sodium	D	1	AE12
Known Interactor	5743	PTGS2	DB01398	Salicylate-sodium	D	1	AE12
Known Interactor	801	CALM1	DB01373	Calcium	D	3	AX04
Known Interactor	801	CALM1	DB00527	Cinchocaine	D	4	AB02
Known Interactor	801	CALM1	DB01069	Promethazine	D	4	AA10
Known Interactor	1956	EGFR	DB05291	Lidocaine	D	4	AB01
Known Interactor	116443	GRIN3A	DB00721	Procaine	D	4	AB03
Known Interactor	5578	PRKCA	DB05013	Ingenol Mebutate	D	6	BX02
Known Interactor	5580	PRKCD	DB05013	Ingenol Mebutate	D	6	BX02
Known Interactor	116443	GRIN3A	DB00898	Ethanol	D	8	AX08
Known Interactor	5295	PIK3R1	DB01064	Isoprenaline	D	8	AX05
Known Interactor	240	ALOX5	DB00586	Diclofenac	D	11	AX18
Known Interactor	801	CALM1	DB01373	Calcium	D	11	AX03
Known Interactor	2932	GSK3B	DB01356	Lithium	D	11	AX04

Known Interactor	2475	MTOR	DB00337	Pimecrolimus	D	11	AH02
Known Interactor	5742	PTGS1	DB08225	Minoxidil	D	11	AX01
Known Interactor	5742	PTGS1	DB00586	Diclofenac	D	11	AX18
Known Interactor	5742	PTGS1	DB00936	Salicylic acid	D	11	AC30
Known Interactor	5743	PTGS2	DB00586	Diclofenac	D	11	AX18
Known Interactor	5743	PTGS2	DB00936	Salicylic acid	D	11	AC30
Known Interactor	221120	ALKBH3	DB00126	Vitamin C	G	1	AD03
Known Interactor	367	AR	DB01026	Ketoconazole	G	1	AF11
Known Interactor	112398	EGLN2	DB00126	Vitamin C	G	1	AD03
Known Interactor	596	BCL2	DB01050	Ibuprofen	G	2	CC01
Known Interactor	1080	CFTR	DB01050	Ibuprofen	G	2	CC01
Known Interactor	5733	PTGER3	DB00917	Dinoprostone	G	2	AD02
Known Interactor	5742	PTGS1	DB00788	Naproxen	G	2	CC02
Known Interactor	5742	PTGS1	DB01050	Ibuprofen	G	2	CC01
Known Interactor	5743	PTGS2	DB00788	Naproxen	G	2	CC02
Known Interactor	5743	PTGS2	DB01050	Ibuprofen	G	2	CC01
Known Interactor	367	AR	DB00506	Levonorgestrel	G	3	AC03
Known Interactor	367	AR	DB00506	Levonorgestrel	G	3	AD01
Known Interactor	367	AR	DB00624	Testosterone	G	3	BA02
Known Interactor	367	AR	DB00624	Testosterone	G	3	BA03
Known Interactor	367	AR	DB00624	Testosterone	G	3	EK01
Known Interactor	367	AR	DB01185	Fluoxymesterone	G	3	BA01
Known Interactor	367	AR	DB01406	Danazol	G	3	XA01
Known Interactor	367	AR	DB02901	Dihydrotestosterone	G	3	BB02
Known Interactor	367	AR	DB04839	Cyproterone acetate	G	3	HA01
Known Interactor	2099	ESR1	DB00255	Diethylstilbestrol	G	3	CB02
Known Interactor	2099	ESR1	DB00255	Diethylstilbestrol	G	3	CC05
Known Interactor	2099	ESR1	DB00269	Chlorotrianisene	G	3	CA06
Known Interactor	2099	ESR1	DB00286	Conjugated Estrogens	G	3	CA57
Known Interactor	2099	ESR1	DB00294	Etonogestrel	G	3	AC08
Known Interactor	2099	ESR1	DB00304	Desogestrel	G	3	AC09
Known Interactor	2099	ESR1	DB00506	Levonorgestrel	G	3	AC03
Known Interactor	2099	ESR1	DB00506	Levonorgestrel	G	3	AD01
Known Interactor	2099	ESR1	DB00396	Progesterone	G	3	AC06

Known Interactor	2099	ESR1	DB00396	Progesterone	G	3	DA02
Known Interactor	2099	ESR1	DB00396	Progesterone	G	3	DA03
Known Interactor	2099	ESR1	DB00396	Progesterone	G	3	DA04
Known Interactor	2099	ESR1	DB00481	Raloxifene	G	3	XC01
Known Interactor	2099	ESR1	DB00655	Estrone	G	3	CA07
Known Interactor	2099	ESR1	DB00655	Estrone	G	3	CC04
Known Interactor	2099	ESR1	DB00783	Estradiol	G	3	CA01
Known Interactor	2099	ESR1	DB00783	Estradiol	G	3	CA03
Known Interactor	2099	ESR1	DB00882	Clomifene	G	3	GB02
Known Interactor	2099	ESR1	DB00890	Dienestrol	G	3	CB01
Known Interactor	2099	ESR1	DB00890	Dienestrol	G	3	CC02
Known Interactor	2099	ESR1	DB00957	Norgestimate	G	3	AA11
Known Interactor	2099	ESR1	DB01185	Fluoxymesterone	G	3	BA01
Known Interactor	2099	ESR1	DB01406	Danazol	G	3	XA01
Known Interactor	2099	ESR1	DB01431	Allylestrenol	G	3	DC01
Known Interactor	2099	ESR1	DB05375	Estriol	G	3	CA04
Known Interactor	2099	ESR1	DB05375	Estriol	G	3	CC06
Known Interactor	3480	IGF1R	DB01277	Mecasermin	H	1	AC03
Known Interactor	367	AR	DB02478	Fludrocortisone	H	2	AA02
Known Interactor	2099	ESR1	DB01108	Trilostane	H	2	CA01
Known Interactor	2740	GLP1R	DB00040	Glucagon recombinant	H	4	AA01
Known Interactor	240	ALOX5	DB01017	Minocycline	J	1	AA08
Known Interactor	116443	GRIN3A	DB00721	Procaine	J	1	CE09
Known Interactor	367	AR	DB01026	Ketoconazole	J	2	AB02
Known Interactor	1786	DNMT1	DB01099	Flucytosine	J	2	AX01
Known Interactor	240	ALOX5	DB00233	Aminosalicylic Acid	J	4	AA01
Known Interactor	801	CALM1	DB01373	Calcium	J	4	AA03
Known Interactor	5743	PTGS2	DB00233	Aminosalicylic Acid	J	4	AA01
Known Interactor	7015	TERT	DB00495	Zidovudine	J	5	AF01
Known Interactor	25	ABL1	DB03261	Imatinib	L	1	XE01
Known Interactor	25	ABL1	DB01254	Dasatinib	L	1	XE06
Known Interactor	25	ABL1	DB04868	Nilotinib	L	1	XE08
Known Interactor	25	ABL1	DB06616	Bosutinib	L	1	XE14
Known Interactor	25	ABL1	DB08896	Regorafenib	L	1	XE21

Known Interactor	207	AKT1	DB01169	Arsenic trioxide	L	1	XX27
Known Interactor	240	ALOX5	DB00179	Masoprocol	L	1	XX10
Known Interactor	596	BCL2	DB05927	Paclitaxel	L	1	CD01
Known Interactor	596	BCL2	DB01248	Docetaxel	L	1	CD02
Known Interactor	595	CCND1	DB01169	Arsenic trioxide	L	1	XX27
Known Interactor	1956	EGFR	DB00002	Cetuximab	L	1	XC06
Known Interactor	1956	EGFR	DB00072	Trastuzumab	L	1	XC03
Known Interactor	1956	EGFR	DB07998	Gefitinib	L	1	XE02
Known Interactor	1956	EGFR	DB00530	Erlotinib	L	1	XE03
Known Interactor	1956	EGFR	DB02584	Lapatinib	L	1	XE07
Known Interactor	1956	EGFR	DB01269	Panitumumab	L	1	XC08
Known Interactor	1956	EGFR	DB05294	Vandetanib	L	1	XE12
Known Interactor	1956	EGFR	DB08916	Afatinib	L	1	XE13
Known Interactor	2099	ESR1	DB01196	Estramustine	L	1	XX11
Known Interactor	2534	FYN	DB01254	Dasatinib	L	1	XE06
Known Interactor	3065	HDAC1	DB02546	Vorinostat	L	1	XX38
Known Interactor	3066	HDAC2	DB02546	Vorinostat	L	1	XX38
Known Interactor	3717	JAK2	DB08877	Ruxolitinib	L	1	XE18
Known Interactor	3725	JUN	DB00570	Vinblastine	L	1	CA01
Known Interactor	3725	JUN	DB01169	Arsenic trioxide	L	1	XX27
Known Interactor	5595	MAPK3	DB01169	Arsenic trioxide	L	1	XX27
Known Interactor	2475	MTOR	DB01590	Everolimus	L	1	XE10
Known Interactor	2475	MTOR	DB06287	Temsirolimus	L	1	XE09
Known Interactor	5170	PDPK1	DB00482	Celecoxib	L	1	XX33
Known Interactor	5423	POLB	DB00987	Cytarabine	L	1	BC01
Known Interactor	5743	PTGS2	DB00482	Celecoxib	L	1	XX33
Known Interactor	367	AR	DB00499	Flutamide	L	2	BB01
Known Interactor	367	AR	DB00665	Nilutamide	L	2	BB02
Known Interactor	367	AR	DB06284	Bicalutamide	L	2	BB03
Known Interactor	2099	ESR1	DB00255	Diethylstilbestrol	L	2	AA01
Known Interactor	2099	ESR1	DB00396	Progesterone	L	2	AB02
Known Interactor	2099	ESR1	DB00539	Toremifene	L	2	BA02
Known Interactor	2099	ESR1	DB00675	Tamoxifen	L	2	BA01
Known Interactor	2099	ESR1	DB00783	Estradiol	L	2	AA02

Known Interactor	2099	ESR1	DB00783	Estradiol	L	2	AA03
Known Interactor	2099	ESR1	DB00947	Fulvestrant	L	2	BA03
Known Interactor	5578	PRKCA	DB00675	Tamoxifen	L	2	BA01
Known Interactor	5579	PRKCB	DB00675	Tamoxifen	L	2	BA01
Known Interactor	5580	PRKCD	DB00675	Tamoxifen	L	2	BA01
Known Interactor	5581	PRKCE	DB00675	Tamoxifen	L	2	BA01
Known Interactor	1439	CSF2RB	DB00020	Sargramostim	L	3	AA09
Known Interactor	596	BCL2	DB05297	DHA-paclitaxel	L	4	AA26
Known Interactor	3717	JAK2	DB08895	Tofacitinib	L	4	AA29
Known Interactor	3718	JAK3	DB08895	Tofacitinib	L	4	AA29
Known Interactor	2475	MTOR	DB02439	Sirolimus	L	4	AA10
Known Interactor	2475	MTOR	DB01590	Everolimus	L	4	AA18
Known Interactor	4790	NFKB1	DB01041	Thalidomide	L	4	AX02
Known Interactor	5478	PPIA	DB00091	Cyclosporine	L	4	AD01
Known Interactor	5743	PTGS2	DB00480	Lenalidomide	L	4	AX04
Known Interactor	5743	PTGS2	DB01041	Thalidomide	L	4	AX02
Known Interactor	2185	PTK2B	DB01097	Leflunomide	L	4	AA13
Known Interactor	240	ALOX5	DB00586	Diclofenac	M	1	AB05
Known Interactor	240	ALOX5	DB00939	Meclofenamic acid	M	1	AG04
Known Interactor	367	AR	DB02266	Flufenamic Acid	M	1	AG03
Known Interactor	596	BCL2	DB01050	Ibuprofen	M	1	AE01
Known Interactor	596	BCL2	DB01050	Ibuprofen	M	1	AE14
Known Interactor	1080	CFTR	DB01050	Ibuprofen	M	1	AE01
Known Interactor	1080	CFTR	DB01050	Ibuprofen	M	1	AE14
Known Interactor	5595	MAPK3	DB00605	Sulindac	M	1	AB02
Known Interactor	5170	PDPK1	DB00482	Celecoxib	M	1	AH01
Known Interactor	5467	PPARD	DB00605	Sulindac	M	1	AB02
Known Interactor	5742	PTGS1	DB00328	Indomethacin	M	1	AB01
Known Interactor	5742	PTGS1	DB00461	Nabumetone	M	1	AX01
Known Interactor	5742	PTGS1	DB00465	Ketorolac	M	1	AB15
Known Interactor	5742	PTGS1	DB00469	Tenoxicam	M	1	AC02
Known Interactor	5742	PTGS1	DB00500	Tolmetin	M	1	AB03
Known Interactor	5742	PTGS1	DB00554	Piroxicam	M	1	AC01
Known Interactor	5742	PTGS1	DB00573	Fenoprofen	M	1	AE04

Known Interactor	5742	PTGS1	DB00586	Diclofenac	M	1	AB05
Known Interactor	5742	PTGS1	DB00605	Sulindac	M	1	AB02
Known Interactor	5742	PTGS1	DB00712	Flurbiprofen	M	1	AE09
Known Interactor	5742	PTGS1	DB00749	Etodolac	M	1	AB08
Known Interactor	5742	PTGS1	DB00784	Mefenamic acid	M	1	AG01
Known Interactor	5742	PTGS1	DB00788	Naproxen	M	1	AE02
Known Interactor	5742	PTGS1	DB08343	Phenylbutazone	M	1	AA01
Known Interactor	5742	PTGS1	DB00814	Meloxicam	M	1	AC06
Known Interactor	5742	PTGS1	DB00870	Suprofen	M	1	AE07
Known Interactor	5742	PTGS1	DB00939	Meclofenamic acid	M	1	AG04
Known Interactor	5742	PTGS1	DB00991	Oxaprozin	M	1	AE12
Known Interactor	5742	PTGS1	DB05823	Ketoprofen	M	1	AE03
Known Interactor	5742	PTGS1	DB05823	Ketoprofen	M	1	AE17
Known Interactor	5742	PTGS1	DB01050	Ibuprofen	M	1	AE01
Known Interactor	5742	PTGS1	DB01050	Ibuprofen	M	1	AE14
Known Interactor	5742	PTGS1	DB01283	Lumiracoxib	M	1	AH06
Known Interactor	5742	PTGS1	DB01600	Tiaprofenic acid	M	1	AE11
Known Interactor	5742	PTGS1	DB02266	Flufenamic Acid	M	1	AG03
Known Interactor	5742	PTGS1	DB04552	Niflumic Acid	M	1	AX02
Known Interactor	5742	PTGS1	DB06725	Lornoxicam	M	1	AC05
Known Interactor	5743	PTGS2	DB00328	Indomethacin	M	1	AB01
Known Interactor	5743	PTGS2	DB00461	Nabumetone	M	1	AX01
Known Interactor	5743	PTGS2	DB00465	Ketorolac	M	1	AB15
Known Interactor	5743	PTGS2	DB00469	Tenoxicam	M	1	AC02
Known Interactor	5743	PTGS2	DB00482	Celecoxib	M	1	AH01
Known Interactor	5743	PTGS2	DB00500	Tolmetin	M	1	AB03
Known Interactor	5743	PTGS2	DB00533	Rofecoxib	M	1	AH02
Known Interactor	5743	PTGS2	DB00554	Piroxicam	M	1	AC01
Known Interactor	5743	PTGS2	DB00573	Fenoprofen	M	1	AE04
Known Interactor	5743	PTGS2	DB07576	Valdecoxib	M	1	AH03
Known Interactor	5743	PTGS2	DB00586	Diclofenac	M	1	AB05
Known Interactor	5743	PTGS2	DB00605	Sulindac	M	1	AB02
Known Interactor	5743	PTGS2	DB00712	Flurbiprofen	M	1	AE09
Known Interactor	5743	PTGS2	DB00749	Etodolac	M	1	AB08

Known Interactor	5743	PTGS2	DB00784	Mefenamic acid	M	1	AG01
Known Interactor	5743	PTGS2	DB00788	Naproxen	M	1	AE02
Known Interactor	5743	PTGS2	DB08343	Phenylbutazone	M	1	AA01
Known Interactor	5743	PTGS2	DB00814	Meloxicam	M	1	AC06
Known Interactor	5743	PTGS2	DB00870	Suprofen	M	1	AE07
Known Interactor	5743	PTGS2	DB00939	Meclofenamic acid	M	1	AG04
Known Interactor	5743	PTGS2	DB00991	Oxaprozin	M	1	AE12
Known Interactor	5743	PTGS2	DB05823	Ketoprofen	M	1	AE03
Known Interactor	5743	PTGS2	DB05823	Ketoprofen	M	1	AE17
Known Interactor	5743	PTGS2	DB01050	Ibuprofen	M	1	AE01
Known Interactor	5743	PTGS2	DB01050	Ibuprofen	M	1	AE14
Known Interactor	5743	PTGS2	DB01283	Lumiracoxib	M	1	AH06
Known Interactor	5743	PTGS2	DB01600	Tiaprofenic acid	M	1	AE11
Known Interactor	5743	PTGS2	DB07166	Etoricoxib	M	1	AH05
Known Interactor	5743	PTGS2	DB02266	Flufenamic Acid	M	1	AG03
Known Interactor	5743	PTGS2	DB04552	Niflumic Acid	M	1	AX02
Known Interactor	5743	PTGS2	DB04743	Nimesulide	M	1	AX17
Known Interactor	5743	PTGS2	DB06725	Lornoxicam	M	1	AC05
Known Interactor	240	ALOX5	DB00586	Diclofenac	M	2	AA15
Known Interactor	240	ALOX5	DB00939	Meclofenamic acid	M	2	AA18
Known Interactor	596	BCL2	DB01050	Ibuprofen	M	2	AA13
Known Interactor	1080	CFTR	DB01050	Ibuprofen	M	2	AA13
Known Interactor	5742	PTGS1	DB00500	Tolmetin	M	2	AA21
Known Interactor	5742	PTGS1	DB00554	Piroxicam	M	2	AA07
Known Interactor	5742	PTGS1	DB00586	Diclofenac	M	2	AA15
Known Interactor	5742	PTGS1	DB00712	Flurbiprofen	M	2	AA19
Known Interactor	5742	PTGS1	DB00788	Naproxen	M	2	AA12
Known Interactor	5742	PTGS1	DB08343	Phenylbutazone	M	2	AA01
Known Interactor	5742	PTGS1	DB00939	Meclofenamic acid	M	2	AA18
Known Interactor	5742	PTGS1	DB05823	Ketoprofen	M	2	AA10
Known Interactor	5742	PTGS1	DB01050	Ibuprofen	M	2	AA13
Known Interactor	5742	PTGS1	DB04552	Niflumic Acid	M	2	AA17
Known Interactor	5743	PTGS2	DB00500	Tolmetin	M	2	AA21
Known Interactor	5743	PTGS2	DB00554	Piroxicam	M	2	AA07

Known Interactor	5743	PTGS2	DB00586	Diclofenac	M	2	AA15
Known Interactor	5743	PTGS2	DB00712	Flurbiprofen	M	2	AA19
Known Interactor	5743	PTGS2	DB00788	Naproxen	M	2	AA12
Known Interactor	5743	PTGS2	DB08343	Phenylbutazone	M	2	AA01
Known Interactor	5743	PTGS2	DB00939	Meclofenamic acid	M	2	AA18
Known Interactor	5743	PTGS2	DB05823	Ketoprofen	M	2	AA10
Known Interactor	5743	PTGS2	DB01050	Ibuprofen	M	2	AA13
Known Interactor	5743	PTGS2	DB04552	Niflumic Acid	M	2	AA17
Known Interactor	5743	PTGS2	DB04743	Nimesulide	M	2	AA26
Known Interactor	2902	GRIN1	DB01173	Orphenadrine	M	3	BC01
Known Interactor	116443	GRIN3A	DB01173	Orphenadrine	M	3	BC01
Known Interactor	116444	GRIN3B	DB01173	Orphenadrine	M	3	BC01
Known Interactor	5775	PTPN4	DB00630	Alendronate	M	5	BA04
Known Interactor	801	CALM1	DB00527	Cinchocaine	N	1	BB06
Known Interactor	801	CALM1	DB00753	Isoflurane	N	1	AB06
Known Interactor	1956	EGFR	DB05291	Lidocaine	N	1	BB02
Known Interactor	116443	GRIN3A	DB00721	Procaine	N	1	BA02
Known Interactor	116443	GRIN3A	DB00721	Procaine	N	1	BA04
Known Interactor	116443	GRIN3A	DB02330	Halothane	N	1	AB01
Known Interactor	116443	GRIN3A	DB01221	Ketamine	N	1	AX03
Known Interactor	116443	GRIN3A	DB01221	Ketamine	N	1	AX14
Known Interactor	116444	GRIN3B	DB02330	Halothane	N	1	AB01
Known Interactor	801	CALM1	DB01373	Calcium	N	2	BA15
Known Interactor	2902	GRIN1	DB00454	Pethidine	N	2	AB02
Known Interactor	2902	GRIN1	DB06738	Ketobemidone	N	2	AB01
Known Interactor	116443	GRIN3A	DB00193	Tramadol	N	2	AX02
Known Interactor	116443	GRIN3A	DB00333	Methadone	N	2	AC52
Known Interactor	116443	GRIN3A	DB06738	Ketobemidone	N	2	AB01
Known Interactor	116444	GRIN3B	DB06738	Ketobemidone	N	2	AB01
Known Interactor	4790	NFKB1	DB00945	Acetylsalicylic acid	N	2	BA01
Known Interactor	27115	PDE7B	DB00201	Caffeine	N	2	BE01
Known Interactor	5562	PRKAA1	DB00945	Acetylsalicylic acid	N	2	BA01
Known Interactor	5565	PRKAB2	DB00945	Acetylsalicylic acid	N	2	BA01
Known Interactor	5742	PTGS1	DB06895	Diflunisal	N	2	BA11

Known Interactor	5742	PTGS1	DB00936	Salicylic acid	N	2	BA04
Known Interactor	5742	PTGS1	DB00945	Acetylsalicylic acid	N	2	BA01
Known Interactor	5742	PTGS1	DB01399	Salsalate	N	2	BA06
Known Interactor	5742	PTGS1	DB01435	Antipyrine	N	2	BB01
Known Interactor	5742	PTGS1	DB08243	Phenacetin	N	2	BE03
Known Interactor	5743	PTGS2	DB06895	Diflunisal	N	2	BA11
Known Interactor	5743	PTGS2	DB00936	Salicylic acid	N	2	BA04
Known Interactor	5743	PTGS2	DB00945	Acetylsalicylic acid	N	2	BA01
Known Interactor	5743	PTGS2	DB01399	Salsalate	N	2	BA06
Known Interactor	5743	PTGS2	DB01435	Antipyrine	N	2	BB01
Known Interactor	7157	TP53	DB00945	Acetylsalicylic acid	N	2	BA01
Known Interactor	2902	GRIN1	DB00996	Gabapentin	N	3	AX12
Known Interactor	2902	GRIN1	DB01174	Phenobarbital	N	3	AA02
Known Interactor	116443	GRIN3A	DB00949	Felbamate	N	3	AX10
Known Interactor	116443	GRIN3A	DB00996	Gabapentin	N	3	AX12
Known Interactor	116443	GRIN3A	DB01174	Phenobarbital	N	3	AA02
Known Interactor	116444	GRIN3B	DB00996	Gabapentin	N	3	AX12
Known Interactor	116444	GRIN3B	DB01174	Phenobarbital	N	3	AA02
Known Interactor	3066	HDAC2	DB00510	Valproic Acid	N	3	AG01
Known Interactor	596	BCL2	DB01367	Rasagiline	N	4	BD02
Known Interactor	2902	GRIN1	DB01173	Orphenadrine	N	4	AB02
Known Interactor	116443	GRIN3A	DB00392	Ethopropazine	N	4	AA05
Known Interactor	116443	GRIN3A	DB00915	Amantadine	N	4	BB01
Known Interactor	116443	GRIN3A	DB01173	Orphenadrine	N	4	AB02
Known Interactor	116444	GRIN3B	DB01173	Orphenadrine	N	4	AB02
Known Interactor	801	CALM1	DB00623	Fluphenazine	N	5	AB02
Known Interactor	801	CALM1	DB08616	Trifluoperazine	N	5	AB06
Known Interactor	801	CALM1	DB00850	Perphenazine	N	5	AB03
Known Interactor	801	CALM1	DB08189	Melatonin	N	5	CH01
Known Interactor	801	CALM1	DB01100	Pimozide	N	5	AG02
Known Interactor	2099	ESR1	DB08189	Melatonin	N	5	CH01
Known Interactor	2902	GRIN1	DB00312	Pentobarbital	N	5	CA01
Known Interactor	2902	GRIN1	DB00418	Secobarbital	N	5	CA06
Known Interactor	116443	GRIN3A	DB00312	Pentobarbital	N	5	CA01

Known Interactor	116443	GRIN3A	DB00418	Secobarbital	N	5	CA06
Known Interactor	116444	GRIN3B	DB00312	Pentobarbital	N	5	CA01
Known Interactor	116444	GRIN3B	DB00418	Secobarbital	N	5	CA06
Known Interactor	2932	GSK3B	DB01356	Lithium	N	5	AN01
Known Interactor	2902	GRIN1	DB00289	Atomoxetine	N	6	BA09
Known Interactor	2902	GRIN1	DB01043	Memantine	N	6	DX01
Known Interactor	2902	GRIN1	DB04896	Milnacipran	N	6	AX17
Known Interactor	116443	GRIN3A	DB00289	Atomoxetine	N	6	BA09
Known Interactor	116443	GRIN3A	DB01043	Memantine	N	6	DX01
Known Interactor	116443	GRIN3A	DB04896	Milnacipran	N	6	AX17
Known Interactor	116444	GRIN3B	DB00289	Atomoxetine	N	6	BA09
Known Interactor	116444	GRIN3B	DB04896	Milnacipran	N	6	AX17
Known Interactor	27115	PDE7B	DB00201	Caffeine	N	6	BC01
Known Interactor	801	CALM1	DB01373	Calcium	N	7	BB02
Known Interactor	801	CALM1	DB04841	Flunarizine	N	7	CA03
Known Interactor	2902	GRIN1	DB00659	Acamprosate	N	7	BB03
Known Interactor	116443	GRIN3A	DB00333	Methadone	N	7	BC02
Known Interactor	116443	GRIN3A	DB00659	Acamprosate	N	7	BB03
Known Interactor	116444	GRIN3B	DB00659	Acamprosate	N	7	BB03
Known Interactor	240	ALOX5	DB00711	Diethylcarbamazine	P	2	CB02
Known Interactor	5742	PTGS1	DB00711	Diethylcarbamazine	P	2	CB02
Known Interactor	801	CALM1	DB01373	Calcium	R	1	AX01
Known Interactor	2353	FOS	DB00852	Pseudoephedrine	R	1	BA02
Known Interactor	3725	JUN	DB00852	Pseudoephedrine	R	1	BA02
Known Interactor	6271	S100A1	DB00768	Olopatadine	R	1	AC08
Known Interactor	1956	EGFR	DB05291	Lidocaine	R	2	AD02
Known Interactor	240	ALOX5	DB00471	Montelukast	R	3	DC03
Known Interactor	3066	HDAC2	DB00277	Theophylline	R	3	DA04
Known Interactor	3066	HDAC2	DB00277	Theophylline	R	3	DA05
Known Interactor	4790	NFKB1	DB01411	Pranlukast	R	3	DC02
Known Interactor	27115	PDE7B	DB00651	Dyphylline	R	3	DA01
Known Interactor	116443	GRIN3A	DB00333	Methadone	R	5	DA06
Known Interactor	116443	GRIN3A	DB00514	Dextromethorphan	R	5	DA09
Known Interactor	801	CALM1	DB01069	Promethazine	R	6	AD02

Known Interactor	801	CALM1	DB01069	Promethazine	R	6	AD05
Known Interactor	27115	PDE7B	DB00920	Ketotifen	R	6	AX17
Known Interactor	1080	CFTR	DB08820	Ivacaftor	R	7	AX02
Known Interactor	221120	ALKBH3	DB00126	Vitamin C	S	1	XA15
Known Interactor	240	ALOX5	DB00586	Diclofenac	S	1	BC03
Known Interactor	358	AQP1	DB00819	Acetazolamide	S	1	EC01
Known Interactor	367	AR	DB00984	Nandrolone phenpropionate	S	1	XA11
Known Interactor	801	CALM1	DB00527	Cinchocaine	S	1	HA06
Known Interactor	1956	EGFR	DB05291	Lidocaine	S	1	HA07
Known Interactor	112398	EGLN2	DB00126	Vitamin C	S	1	XA15
Known Interactor	116443	GRIN3A	DB00721	Procaine	S	1	HA02
Known Interactor	116443	GRIN3A	DB00721	Procaine	S	1	HA05
Known Interactor	27115	PDE7B	DB00920	Ketotifen	S	1	GX08
Known Interactor	5478	PPIA	DB00091	Cyclosporine	S	1	XA18
Known Interactor	5733	PTGER3	DB06863	Bimatoprost	S	1	EE03
Known Interactor	5742	PTGS1	DB00465	Ketorolac	S	1	BC05
Known Interactor	5742	PTGS1	DB00554	Piroxicam	S	1	BC06
Known Interactor	5742	PTGS1	DB00586	Diclofenac	S	1	BC03
Known Interactor	5742	PTGS1	DB00712	Flurbiprofen	S	1	BC04
Known Interactor	5742	PTGS1	DB01398	Salicylate-sodium	S	1	BC08
Known Interactor	5742	PTGS1	DB06802	Nepafenac	S	1	BC10
Known Interactor	5743	PTGS2	DB00465	Ketorolac	S	1	BC05
Known Interactor	5743	PTGS2	DB00554	Piroxicam	S	1	BC06
Known Interactor	5743	PTGS2	DB00586	Diclofenac	S	1	BC03
Known Interactor	5743	PTGS2	DB00712	Flurbiprofen	S	1	BC04
Known Interactor	5743	PTGS2	DB01398	Salicylate-sodium	S	1	BC08
Known Interactor	5743	PTGS2	DB06802	Nepafenac	S	1	BC10
Known Interactor	6271	S100A1	DB00768	Olopatadine	S	1	GX09
Known Interactor	801	CALM1	DB00527	Cinchocaine	S	2	DA04
Known Interactor	1956	EGFR	DB05291	Lidocaine	S	2	DA01
Known Interactor	5742	PTGS1	DB01435	Antipyrine	S	2	DA03
Known Interactor	5743	PTGS2	DB01435	Antipyrine	S	2	DA03
Known Interactor	801	CALM1	DB01373	Calcium	V	3	AE01
Known Interactor	801	CALM1	DB01373	Calcium	V	3	AF04

Known Interactor	2099	ESR1	DB01183	Naloxone	V	3	AB15
Known Interactor	116443	GRIN3A	DB00898	Ethanol	V	3	AB16
Known Interactor	116443	GRIN3A	DB00898	Ethanol	V	3	AZ01
Known Interactor	801	CALM1	DB01373	Calcium	V	8	AC10
Novel Interactor	31	ACACA	DB00121	Biotin	A	11	HA05
Novel Interactor	836	CASP3	DB01017	Minocycline	A	1	AB23
Novel Interactor	2891	GRIA2	DB00142	L-Glutamic Acid	A	9	AB01
Novel Interactor	5959	RDH5	DB00162	Vitamin A	A	11	CA01
Novel Interactor	6286	S100P	DB01003	Cromoglicic acid	A	7	EB01
Novel Interactor	6822	SULT2A1	DB06593	Dehydroepiandrosterone	A	14	AA07
Novel Interactor	7421	VDR	DB00136	Calcitriol	A	11	CC04
Novel Interactor	7421	VDR	DB00146	Calcidiol	A	11	CC06
Novel Interactor	7421	VDR	DB00153	Ergocalciferol	A	11	CC01
Novel Interactor	7421	VDR	DB00169	Cholecalciferol	A	11	CC05
Novel Interactor	7421	VDR	DB01070	Dihydrotachysterol	A	11	CC02
Novel Interactor	7421	VDR	DB01436	Alfacalcidol	A	11	CC03
Novel Interactor	444	ASPH	DB00128	L-Aspartic Acid	B	3	AA09
Novel Interactor	4035	LRP1	DB00025	Antihemophilic Factor	B	2	BD02
Novel Interactor	4035	LRP1	DB00031	Tenecteplase	B	1	AD11
Novel Interactor	4035	LRP1	DB00100	Coagulation Factor IX	B	2	BD04
Novel Interactor	40	ASIC2	DB00594	Amiloride	C	3	DB01
Novel Interactor	8912	CACNA1H	DB00270	Isradipine	C	8	CA03
Novel Interactor	8912	CACNA1H	DB00421	Spironolactone	C	3	DA01
Novel Interactor	8912	CACNA1H	DB01023	Felodipine	C	8	CA02
Novel Interactor	8912	CACNA1H	DB01054	Nitrendipine	C	8	CA08
Novel Interactor	8912	CACNA1H	DB01115	Nifedipine	C	8	CA05
Novel Interactor	8912	CACNA1H	DB01118	Amiodarone	C	1	BD01
Novel Interactor	8912	CACNA1H	DB01244	Bepridil	C	8	EA02
Novel Interactor	8912	CACNA1H	DB01388	Mibefradil	C	8	CX01
Novel Interactor	3269	HRH1	DB00455	Loratadine	C	9	AA03
Novel Interactor	3269	HRH1	DB00797	Tolazoline	C	4	AB02
Novel Interactor	6323	SCN1A	DB04855	Dronedarone	C	1	BD07
Novel Interactor	1595	CYP51A1	DB01007	Tioconazole	D	1	AC07
Novel Interactor	2891	GRIA2	DB00794	Primidone	D	8	AC04

Novel Interactor	3269	HRH1	DB00283	Clemastine	D	4	AA14
Novel Interactor	3269	HRH1	DB00792	Tripelennamine	D	4	AA04
Novel Interactor	3269	HRH1	DB01069	Promethazine	D	4	AA10
Novel Interactor	3269	HRH1	DB06975	Diphenhydramine	D	4	AA32
Novel Interactor	3269	HRH1	DB06975	Diphenhydramine	D	4	AA33
Novel Interactor	3269	HRH1	DB06691	Mepyramine	D	4	AA02
Novel Interactor	3269	HRH1	DB07523	Chloropyramine	D	4	AA09
Novel Interactor	3269	HRH1	DB08801	Dimetindene	D	4	AA13
Novel Interactor	3269	HRH1	DB08802	Isothipendyl	D	4	AA22
Novel Interactor	5959	RDH5	DB00162	Vitamin A	D	10	AD02
Novel Interactor	6286	S100P	DB01003	Cromoglicic acid	D	11	AH03
Novel Interactor	7421	VDR	DB00136	Calcitriol	D	5	AX03
Novel Interactor	7421	VDR	DB02300	Calcipotriol	D	5	AX02
Novel Interactor	1595	CYP51A1	DB01007	Tioconazole	G	1	AF08
Novel Interactor	6323	SCN1A	DB01438	Phenazopyridine	G	4	BX06
Novel Interactor	846	CASR	DB01012	Cinacalcet	H	5	BX01
Novel Interactor	7421	VDR	DB00910	Paricalcitol	H	5	BX02
Novel Interactor	836	CASP3	DB01017	Minocycline	J	1	AA08
Novel Interactor	1595	CYP51A1	DB01167	Itraconazole	J	2	AC02
Novel Interactor	1969	EPHA2	DB01254	Dasatinib	L	1	XE06
Novel Interactor	1969	EPHA2	DB08896	Regorafenib	L	1	XE21
Novel Interactor	116447	TOP1MT	DB00762	Irinotecan	L	1	XX19
Novel Interactor	116447	TOP1MT	DB01030	Topotecan	L	1	XX17
Novel Interactor	3269	HRH1	DB00797	Tolazoline	M	2	AX02
Novel Interactor	3269	HRH1	DB01173	Orphenadrine	M	3	BC01
Novel Interactor	8912	CACNA1H	DB00568	Cinnarizine	N	7	CA02
Novel Interactor	8912	CACNA1H	DB00909	Zonisamide	N	3	AX15
Novel Interactor	8912	CACNA1H	DB04841	Flunarizine	N	7	CA03
Novel Interactor	2891	GRIA2	DB00306	Talbutal	N	5	CA07
Novel Interactor	2891	GRIA2	DB00312	Pentobarbital	N	5	CA01
Novel Interactor	2891	GRIA2	DB00418	Secobarbital	N	5	CA06
Novel Interactor	2891	GRIA2	DB00463	Metharbital	N	3	AA30
Novel Interactor	2891	GRIA2	DB00599	Thiopental	N	1	AF03
Novel Interactor	2891	GRIA2	DB00599	Thiopental	N	5	CA19

Novel Interactor	2891	GRIA2	DB00794	Primidone	N	3	AA03
Novel Interactor	2891	GRIA2	DB00849	Methylphenobarbital	N	3	AA01
Novel Interactor	2891	GRIA2	DB01174	Phenobarbital	N	3	AA02
Novel Interactor	2891	GRIA2	DB01351	Amobarbital	N	5	CA02
Novel Interactor	2891	GRIA2	DB01352	Aprobarbital	N	5	CA05
Novel Interactor	2891	GRIA2	DB01354	Heptabarbital	N	5	CA11
Novel Interactor	2891	GRIA2	DB01355	Hexobarbital	N	1	AF02
Novel Interactor	2891	GRIA2	DB01355	Hexobarbital	N	5	CA16
Novel Interactor	2891	GRIA2	DB04599	Aniracetam	N	6	BX11
Novel Interactor	3269	HRH1	DB00215	Citalopram	N	6	AB04
Novel Interactor	3269	HRH1	DB00215	Citalopram	N	6	AB10
Novel Interactor	3269	HRH1	DB00245	Benzatropine	N	4	AC01
Novel Interactor	3269	HRH1	DB00246	Ziprasidone	N	5	AE04
Novel Interactor	3269	HRH1	DB00321	Amitriptyline	N	6	AA09
Novel Interactor	3269	HRH1	DB00334	Olanzapine	N	5	AH03
Novel Interactor	3269	HRH1	DB00363	Clozapine	N	5	AH02
Novel Interactor	3269	HRH1	DB00370	Mirtazapine	N	6	AX11
Novel Interactor	3269	HRH1	DB00408	Loxapine	N	5	AH01
Novel Interactor	3269	HRH1	DB00420	Promazine	N	5	AA01
Novel Interactor	3269	HRH1	DB00420	Promazine	N	5	AA02
Novel Interactor	3269	HRH1	DB00420	Promazine	N	5	AA03
Novel Interactor	3269	HRH1	DB00420	Promazine	N	5	AA04
Novel Interactor	3269	HRH1	DB00420	Promazine	N	5	AA05
Novel Interactor	3269	HRH1	DB08002	Imipramine	N	6	AA02
Novel Interactor	3269	HRH1	DB08002	Imipramine	N	6	AA03
Novel Interactor	3269	HRH1	DB08002	Imipramine	N	6	AA06
Novel Interactor	3269	HRH1	DB00540	Nortriptyline	N	6	AA10
Novel Interactor	3269	HRH1	DB00543	Amoxapine	N	6	AA17
Novel Interactor	3269	HRH1	DB00557	Hydroxyzine	N	5	BB01
Novel Interactor	3269	HRH1	DB00568	Cinnarizine	N	7	CA02
Novel Interactor	3269	HRH1	DB00656	Trazodone	N	6	AX05
Novel Interactor	3269	HRH1	DB00734	Risperidone	N	5	AX08
Novel Interactor	3269	HRH1	DB00777	Propiomazine	N	5	CM06
Novel Interactor	3269	HRH1	DB00934	Maprotiline	N	6	AA21

Novel Interactor	3269	HRH1	DB01142	Doxepin	N	6	AA12
Novel Interactor	3269	HRH1	DB07682	Desipramine	N	6	AA01
Novel Interactor	3269	HRH1	DB01173	Orphenadrine	N	4	AB02
Novel Interactor	3269	HRH1	DB01224	Quetiapine	N	5	AH04
Novel Interactor	3269	HRH1	DB01238	Aripiprazole	N	5	AX12
Novel Interactor	3269	HRH1	DB01239	Chlorprothixene	N	5	AF03
Novel Interactor	3269	HRH1	DB01267	Paliperidone	N	5	AX13
Novel Interactor	3269	HRH1	DB08920	Zuclopenthixol	N	5	AF05
Novel Interactor	3269	HRH1	DB04841	Flunarizine	N	7	CA03
Novel Interactor	3269	HRH1	DB04946	Iloperidone	N	5	AX14
Novel Interactor	3269	HRH1	DB06148	Mianserin	N	6	AX03
Novel Interactor	3269	HRH1	DB06216	Asenapine	N	5	AH05
Novel Interactor	3269	HRH1	DB06698	Betahistine	N	7	CA01
Novel Interactor	3760	KCNJ3	DB02330	Halothane	N	1	AB01
Novel Interactor	6323	SCN1A	DB00252	Phenytoin	N	3	AB02
Novel Interactor	6323	SCN1A	DB00252	Phenytoin	N	3	AB04
Novel Interactor	6323	SCN1A	DB00252	Phenytoin	N	3	AB05
Novel Interactor	6323	SCN1A	DB00273	Topiramate	N	3	AX11
Novel Interactor	6323	SCN1A	DB00510	Valproic Acid	N	3	AG01
Novel Interactor	6323	SCN1A	DB00909	Zonisamide	N	3	AX15
Novel Interactor	6323	SCN1A	DB01121	Phenacemide	N	3	AX07
Novel Interactor	6323	SCN1A	DB01595	Nitrazepam	N	5	CD02
Novel Interactor	6323	SCN1A	DB04930	Permethrin	P	3	AC04
Novel Interactor	2891	GRIA2	DB00794	Primidone	R	1	AX07
Novel Interactor	2891	GRIA2	DB00794	Primidone	R	2	AA18
Novel Interactor	3269	HRH1	DB00283	Clemastine	R	6	AA04
Novel Interactor	3269	HRH1	DB00341	Cetirizine	R	6	AE07
Novel Interactor	3269	HRH1	DB00341	Cetirizine	R	6	AE09
Novel Interactor	3269	HRH1	DB00342	Terfenadine	R	6	AX12
Novel Interactor	3269	HRH1	DB00354	Buclizine	R	6	AE01
Novel Interactor	3269	HRH1	DB00366	Doxylamine	R	6	AA09
Novel Interactor	3269	HRH1	DB00405	Dexbrompheniramine	R	6	AB06
Novel Interactor	3269	HRH1	DB00427	Triprolidine	R	6	AX07
Novel Interactor	3269	HRH1	DB00434	Cyproheptadine	R	6	AX02

Novel Interactor	3269	HRH1	DB00455	Loratadine	R	6	AX13
Novel Interactor	3269	HRH1	DB00455	Loratadine	R	6	AX27
Novel Interactor	3269	HRH1	DB00637	Astemizole	R	6	AX11
Novel Interactor	3269	HRH1	DB00719	Azatadine	R	6	AX09
Novel Interactor	3269	HRH1	DB00737	Meclizine	R	6	AE05
Novel Interactor	3269	HRH1	DB00748	Carbinoxamine	R	6	AA08
Novel Interactor	3269	HRH1	DB00751	Epinastine	R	6	AX24
Novel Interactor	3269	HRH1	DB00768	Olopatadine	R	1	AC08
Novel Interactor	3269	HRH1	DB00792	Tripelennamine	R	6	AC04
Novel Interactor	3269	HRH1	DB008	Brompheniramine	R	6	AB01
Novel Interactor	3269	HRH1	DB00902	Methdilazine	R	6	AD04
Novel Interactor	3269	HRH1	DB00920	Ketotifen	R	6	AX17
Novel Interactor	3269	HRH1	DB00950	Fexofenadine	R	6	AX26
Novel Interactor	3269	HRH1	DB00972	Azelastine	R	1	AC03
Novel Interactor	3269	HRH1	DB00972	Azelastine	R	6	AX19
Novel Interactor	3269	HRH1	DB00985	Dimenhydrinate	R	6	AA02
Novel Interactor	3269	HRH1	DB01069	Promethazine	R	6	AD02
Novel Interactor	3269	HRH1	DB01069	Promethazine	R	6	AD05
Novel Interactor	3269	HRH1	DB01071	Mequitazine	R	6	AD07
Novel Interactor	3269	HRH1	DB01106	Levocabastine	R	1	AC02
Novel Interactor	3269	HRH1	DB01114	Chlorphenamine	R	6	AB02
Novel Interactor	3269	HRH1	DB01114	Chlorphenamine	R	6	AB04
Novel Interactor	3269	HRH1	DB01146	Diphenylpyraline	R	6	AA07
Novel Interactor	3269	HRH1	DB01176	Cyclizine	R	6	AE03
Novel Interactor	3269	HRH1	DB01176	Cyclizine	R	6	AE04
Novel Interactor	3269	HRH1	DB01246	Alimemazine	R	6	AD01
Novel Interactor	3269	HRH1	DB01619	Phenindamine	R	6	AX04
Novel Interactor	3269	HRH1	DB01620	Pheniramine	R	6	AB05
Novel Interactor	3269	HRH1	DB06691	Mepyramine	R	6	AC01
Novel Interactor	3269	HRH1	DB08799	Antazoline	R	1	AC04
Novel Interactor	3269	HRH1	DB07523	Chloropyramine	R	6	AC03
Novel Interactor	3269	HRH1	DB08801	Dimetindene	R	6	AB03
Novel Interactor	3269	HRH1	DB08802	Isothipendyl	R	6	AD09
Novel Interactor	5959	RDH5	DB00162	Vitamin A	R	1	AX02

Novel Interactor	6286	S100P	DB01003	Cromoglicic acid	R	1	AC01
Novel Interactor	6286	S100P	DB01003	Cromoglicic acid	R	3	BC01
Novel Interactor	2891	GRIA2	DB00794	Primidone	S	1	AX08
Novel Interactor	2891	GRIA2	DB00794	Primidone	S	3	AA05
Novel Interactor	3269	HRH1	DB00751	Epinastine	S	1	GX10
Novel Interactor	3269	HRH1	DB00768	Olopatadine	S	1	GX09
Novel Interactor	3269	HRH1	DB00920	Ketotifen	S	1	GX08
Novel Interactor	3269	HRH1	DB00972	Azelastine	S	1	GX07
Novel Interactor	3269	HRH1	DB01084	Emedastine	S	1	GX06
Novel Interactor	3269	HRH1	DB01106	Levocabastine	S	1	GX02
Novel Interactor	8829	NRP1	DB04895	Pegaptanib	S	1	LA03
Novel Interactor	5959	RDH5	DB00162	Vitamin A	S	1	XA02
Novel Interactor	6286	S100P	DB01003	Cromoglicic acid	S	1	GX01
Novel Interactor	3269	HRH1	DB00667	Histamine Phosphate	V	4	CG03
Novel Interactor	3269	HRH1	DB01146	Diphenylpyraline	V	1	AA02
Novel Interactor	3269	HRH1	DB01146	Diphenylpyraline	V	1	AA20
Novel Interactor	8829	NRP1	DB00039	Palifermin	V	3	AF08

Drugs of Historical Gene Interactome

Gene Role	Gene Entrez ID	Gene Symbol	Drugbank ID	Drug Name	Category Anatomic	Category Therapeutic	Category Chemical
Candidate Gene	207	AKT1	DB01169	Arsenic trioxide	L	1	XX27
Candidate Gene	1139	CHRNA7	DB00184	Nicotine	A	11	HA01
Candidate Gene	1139	CHRNA7	DB00184	Nicotine	C	4	AC01
Candidate Gene	1139	CHRNA7	DB00184	Nicotine	C	10	AD02
Candidate Gene	1139	CHRNA7	DB00184	Nicotine	N	7	BA01
Candidate Gene	1139	CHRNA7	DB00306	Talbutal	N	5	CA07
Candidate Gene	1139	CHRNA7	DB00312	Pentobarbital	N	5	CA01
Candidate Gene	1139	CHRNA7	DB00418	Secobarbital	N	5	CA06
Candidate Gene	1139	CHRNA7	DB00463	Metharbital	N	3	AA30
Candidate Gene	1139	CHRNA7	DB00514	Dextromethorphan	R	5	DA09
Candidate Gene	1139	CHRNA7	DB00599	Thiopental	N	1	AF03
Candidate Gene	1139	CHRNA7	DB00599	Thiopental	N	5	CA19
Candidate Gene	1139	CHRNA7	DB00674	Galantamine	N	6	DA04
Candidate Gene	1139	CHRNA7	DB00794	Primidone	D	8	AC04
Candidate Gene	1139	CHRNA7	DB00794	Primidone	N	3	AA03
Candidate Gene	1139	CHRNA7	DB00794	Primidone	R	1	AX07
Candidate Gene	1139	CHRNA7	DB00794	Primidone	R	2	AA18
Candidate Gene	1139	CHRNA7	DB00794	Primidone	S	1	AX08
Candidate Gene	1139	CHRNA7	DB00794	Primidone	S	3	AA05
Candidate Gene	1139	CHRNA7	DB00849	Methylphenobarbital	N	3	AA01
Candidate Gene	1139	CHRNA7	DB01174	Phenobarbital	N	3	AA02
Candidate Gene	1139	CHRNA7	DB01273	Varenicline	N	7	BA03
Candidate Gene	1139	CHRNA7	DB01351	Amobarbital	N	5	CA02
Candidate Gene	1139	CHRNA7	DB01352	Aprobarbital	N	5	CA05
Candidate Gene	1139	CHRNA7	DB01354	Heptabarbital	N	5	CA11
Candidate Gene	1139	CHRNA7	DB01355	Hexobarbital	N	1	AF02
Candidate Gene	1139	CHRNA7	DB01355	Hexobarbital	N	5	CA16
Candidate Gene	1312	COMT	DB00323	Tolcapone	N	4	BX01
Candidate Gene	1312	COMT	DB00494	Entacapone	N	4	BX02
Candidate Gene	1312	COMT	DB04820	Nialamide	N	6	AF02

Candidate Gene	1813	DRD2	DB00182	Amphetamine	N	6	BA01
Candidate Gene	1813	DRD2	DB00246	Ziprasidone	N	5	AE04
Candidate Gene	1813	DRD2	DB00248	Cabergoline	G	2	CB03
Candidate Gene	1813	DRD2	DB00248	Cabergoline	N	4	BC06
Candidate Gene	1813	DRD2	DB00268	Ropinirole	N	4	BC04
Candidate Gene	1813	DRD2	DB00334	Olanzapine	N	5	AH03
Candidate Gene	1813	DRD2	DB00363	Clozapine	N	5	AH02
Candidate Gene	1813	DRD2	DB00370	Mirtazapine	N	6	AX11
Candidate Gene	1813	DRD2	DB00372	Thiethylperazine	R	6	AD03
Candidate Gene	1813	DRD2	DB00391	Sulpiride	N	5	AL01
Candidate Gene	1813	DRD2	DB00391	Sulpiride	N	5	AL07
Candidate Gene	1813	DRD2	DB00408	Loxapine	N	5	AH01
Candidate Gene	1813	DRD2	DB00409	Remoxipride	N	5	AL04
Candidate Gene	1813	DRD2	DB00413	Pramipexole	N	4	BC05
Candidate Gene	1813	DRD2	DB00420	Promazine	N	5	AA01
Candidate Gene	1813	DRD2	DB00420	Promazine	N	5	AA02
Candidate Gene	1813	DRD2	DB00420	Promazine	N	5	AA03
Candidate Gene	1813	DRD2	DB00420	Promazine	N	5	AA04
Candidate Gene	1813	DRD2	DB00420	Promazine	N	5	AA05
Candidate Gene	1813	DRD2	DB00433	Prochlorperazine	N	5	AB04
Candidate Gene	1813	DRD2	DB00450	Droperidol	N	5	AD08
Candidate Gene	1813	DRD2	DB08002	Imipramine	N	6	AA02
Candidate Gene	1813	DRD2	DB08002	Imipramine	N	6	AA03
Candidate Gene	1813	DRD2	DB08002	Imipramine	N	6	AA06
Candidate Gene	1813	DRD2	DB00490	Buspirone	N	5	BE01
Candidate Gene	1813	DRD2	DB00502	Haloperidol	N	5	AD01
Candidate Gene	1813	DRD2	DB00540	Nortriptyline	N	6	AA10
Candidate Gene	1813	DRD2	DB00543	Amoxapine	N	6	AA17
Candidate Gene	1813	DRD2	DB00568	Cinnarizine	N	7	CA02
Candidate Gene	1813	DRD2	DB00589	Lisuride	G	2	CB02
Candidate Gene	1813	DRD2	DB00589	Lisuride	N	2	CA07
Candidate Gene	1813	DRD2	DB00623	Fluphenazine	N	5	AB02
Candidate Gene	1813	DRD2	DB00679	Thioridazine	N	5	AC02
Candidate Gene	1813	DRD2	DB00696	Ergotamine	N	2	CA02

Candidate Gene	1813	DRD2	DB00734	Risperidone	N	5	AX08
Candidate Gene	1813	DRD2	DB00777	Propiomazine	N	5	CM06
Candidate Gene	1813	DRD2	DB00805	Minaprine	N	6	AX07
Candidate Gene	1813	DRD2	DB08616	Trifluoperazine	N	5	AB06
Candidate Gene	1813	DRD2	DB00850	Perphenazine	N	5	AB03
Candidate Gene	1813	DRD2	DB00915	Amantadine	N	4	BB01
Candidate Gene	1813	DRD2	DB00933	Mesoridazine	N	5	AC03
Candidate Gene	1813	DRD2	DB00934	Maprotiline	N	6	AA21
Candidate Gene	1813	DRD2	DB00988	Dopamine	C	1	CA04
Candidate Gene	1813	DRD2	DB01043	Memantine	N	6	DX01
Candidate Gene	1813	DRD2	DB01287	Ergoloid mesylate	C	4	AE01
Candidate Gene	1813	DRD2	DB01063	Acetophenazine	N	5	AB07
Candidate Gene	1813	DRD2	DB01069	Promethazine	D	4	AA10
Candidate Gene	1813	DRD2	DB01069	Promethazine	R	6	AD02
Candidate Gene	1813	DRD2	DB01069	Promethazine	R	6	AD05
Candidate Gene	1813	DRD2	DB01100	Pimozide	N	5	AG02
Candidate Gene	1813	DRD2	DB01142	Doxepin	N	6	AA12
Candidate Gene	1813	DRD2	DB07682	Desipramine	N	6	AA01
Candidate Gene	1813	DRD2	DB01184	Domperidone	A	3	FA03
Candidate Gene	1813	DRD2	DB01186	Pergolide	N	4	BC02
Candidate Gene	1813	DRD2	DB01200	Bromocriptine	G	2	CB01
Candidate Gene	1813	DRD2	DB01200	Bromocriptine	N	4	BC01
Candidate Gene	1813	DRD2	DB01221	Ketamine	N	1	AX03
Candidate Gene	1813	DRD2	DB01221	Ketamine	N	1	AX14
Candidate Gene	1813	DRD2	DB01224	Quetiapine	N	5	AH04
Candidate Gene	1813	DRD2	DB01233	Metoclopramide	A	3	FA01
Candidate Gene	1813	DRD2	DB01235	L-DOPA	N	4	BA01
Candidate Gene	1813	DRD2	DB01235	L-DOPA	N	4	BA04
Candidate Gene	1813	DRD2	DB01238	Aripiprazole	N	5	AX12
Candidate Gene	1813	DRD2	DB01239	Chlorprothixene	N	5	AF03
Candidate Gene	1813	DRD2	DB01267	Paliperidone	N	5	AX13
Candidate Gene	1813	DRD2	DB01392	Yohimbine	G	4	BE04
Candidate Gene	1813	DRD2	DB01425	Alizapride	A	3	FA05
Candidate Gene	1813	DRD2	DB01618	Molindone	N	5	AE02

Candidate Gene	1813	DRD2	DB01621	Pipotiazine	N	5	AC04
Candidate Gene	1813	DRD2	DB01622	Thioproperazine	N	5	AB08
Candidate Gene	1813	DRD2	DB01623	Thiothixene	N	5	AF04
Candidate Gene	1813	DRD2	DB08920	Zuclopenthixol	N	5	AF05
Candidate Gene	1813	DRD2	DB04599	Aniracetam	N	6	BX11
Candidate Gene	1813	DRD2	DB04842	Fluspirilene	N	5	AG01
Candidate Gene	1813	DRD2	DB04844	Tetrabenazine	N	7	XX06
Candidate Gene	1813	DRD2	DB04946	Iloperidone	N	5	AX14
Candidate Gene	1813	DRD2	DB05271	Rotigotine	N	4	BC09
Candidate Gene	1813	DRD2	DB06144	Sertindole	N	5	AE03
Candidate Gene	1813	DRD2	DB06148	Mianserin	N	6	AX03
Candidate Gene	1813	DRD2	DB06216	Asenapine	N	5	AH05
Candidate Gene	1813	DRD2	DB06288	Amisulpride	N	5	AL05
Candidate Gene	1813	DRD2	DB08815	Lurasidone	N	5	AE05
Candidate Gene	1814	DRD3	DB00246	Ziprasidone	N	5	AE04
Candidate Gene	1814	DRD3	DB00248	Cabergoline	G	2	CB03
Candidate Gene	1814	DRD3	DB00248	Cabergoline	N	4	BC06
Candidate Gene	1814	DRD3	DB00268	Ropinirole	N	4	BC04
Candidate Gene	1814	DRD3	DB00334	Olanzapine	N	5	AH03
Candidate Gene	1814	DRD3	DB00363	Clozapine	N	5	AH02
Candidate Gene	1814	DRD3	DB00370	Mirtazapine	N	6	AX11
Candidate Gene	1814	DRD3	DB00391	Sulpiride	N	5	AL01
Candidate Gene	1814	DRD3	DB00391	Sulpiride	N	5	AL07
Candidate Gene	1814	DRD3	DB00408	Loxapine	N	5	AH01
Candidate Gene	1814	DRD3	DB00409	Remoxipride	N	5	AL04
Candidate Gene	1814	DRD3	DB00413	Pramipexole	N	4	BC05
Candidate Gene	1814	DRD3	DB00502	Haloperidol	N	5	AD01
Candidate Gene	1814	DRD3	DB00543	Amoxapine	N	6	AA17
Candidate Gene	1814	DRD3	DB00589	Lisuride	G	2	CB02
Candidate Gene	1814	DRD3	DB00589	Lisuride	N	2	CA07
Candidate Gene	1814	DRD3	DB00734	Risperidone	N	5	AX08
Candidate Gene	1814	DRD3	DB00988	Dopamine	C	1	CA04
Candidate Gene	1814	DRD3	DB01100	Pimozide	N	5	AG02
Candidate Gene	1814	DRD3	DB01184	Domperidone	A	3	FA03

Candidate Gene	1814	DRD3	DB01186	Pergolide	N	4	BC02
Candidate Gene	1814	DRD3	DB01200	Bromocriptine	G	2	CB01
Candidate Gene	1814	DRD3	DB01200	Bromocriptine	N	4	BC01
Candidate Gene	1814	DRD3	DB01224	Quetiapine	N	5	AH04
Candidate Gene	1814	DRD3	DB01235	L-DOPA	N	4	BA01
Candidate Gene	1814	DRD3	DB01235	L-DOPA	N	4	BA04
Candidate Gene	1814	DRD3	DB01238	Aripiprazole	N	5	AX12
Candidate Gene	1814	DRD3	DB01239	Chlorprothixene	N	5	AF03
Candidate Gene	1814	DRD3	DB01267	Paliperidone	N	5	AX13
Candidate Gene	1814	DRD3	DB01392	Yohimbine	G	4	BE04
Candidate Gene	1814	DRD3	DB04946	Iloperidone	N	5	AX14
Candidate Gene	1814	DRD3	DB05271	Rotigotine	N	4	BC09
Candidate Gene	1814	DRD3	DB06148	Mianserin	N	6	AX03
Candidate Gene	1814	DRD3	DB06216	Asenapine	N	5	AH05
Candidate Gene	1814	DRD3	DB06288	Amisulpride	N	5	AL05
Candidate Gene	1815	DRD4	DB00246	Ziprasidone	N	5	AE04
Candidate Gene	1815	DRD4	DB00248	Cabergoline	G	2	CB03
Candidate Gene	1815	DRD4	DB00248	Cabergoline	N	4	BC06
Candidate Gene	1815	DRD4	DB00268	Ropinirole	N	4	BC04
Candidate Gene	1815	DRD4	DB00334	Olanzapine	N	5	AH03
Candidate Gene	1815	DRD4	DB00363	Clozapine	N	5	AH02
Candidate Gene	1815	DRD4	DB00372	Thiethylperazine	R	6	AD03
Candidate Gene	1815	DRD4	DB00408	Loxapine	N	5	AH01
Candidate Gene	1815	DRD4	DB00409	Remoxipride	N	5	AL04
Candidate Gene	1815	DRD4	DB00413	Pramipexole	N	4	BC05
Candidate Gene	1815	DRD4	DB00420	Promazine	N	5	AA01
Candidate Gene	1815	DRD4	DB00420	Promazine	N	5	AA02
Candidate Gene	1815	DRD4	DB00420	Promazine	N	5	AA03
Candidate Gene	1815	DRD4	DB00420	Promazine	N	5	AA04
Candidate Gene	1815	DRD4	DB00420	Promazine	N	5	AA05
Candidate Gene	1815	DRD4	DB00543	Amoxapine	N	6	AA17
Candidate Gene	1815	DRD4	DB00589	Lisuride	G	2	CB02
Candidate Gene	1815	DRD4	DB00589	Lisuride	N	2	CA07
Candidate Gene	1815	DRD4	DB00734	Risperidone	N	5	AX08

Candidate Gene	1815	DRD4	DB00777	Propiomazine	N	5	CM06
Candidate Gene	1815	DRD4	DB00988	Dopamine	C	1	CA04
Candidate Gene	1815	DRD4	DB01186	Pergolide	N	4	BC02
Candidate Gene	1815	DRD4	DB01200	Bromocriptine	G	2	CB01
Candidate Gene	1815	DRD4	DB01200	Bromocriptine	N	4	BC01
Candidate Gene	1815	DRD4	DB01224	Quetiapine	N	5	AH04
Candidate Gene	1815	DRD4	DB01235	L-DOPA	N	4	BA01
Candidate Gene	1815	DRD4	DB01235	L-DOPA	N	4	BA04
Candidate Gene	1815	DRD4	DB01238	Aripiprazole	N	5	AX12
Candidate Gene	1815	DRD4	DB01267	Paliperidone	N	5	AX13
Candidate Gene	1815	DRD4	DB04946	Iloperidone	N	5	AX14
Candidate Gene	1815	DRD4	DB05271	Rotigotine	N	4	BC09
Candidate Gene	1815	DRD4	DB06216	Asenapine	N	5	AH05
Candidate Gene	3356	HTR2A	DB00246	Ziprasidone	N	5	AE04
Candidate Gene	3356	HTR2A	DB00247	Methysergide	N	2	CA04
Candidate Gene	3356	HTR2A	DB00248	Cabergoline	G	2	CB03
Candidate Gene	3356	HTR2A	DB00248	Cabergoline	N	4	BC06
Candidate Gene	3356	HTR2A	DB00268	Ropinirole	N	4	BC04
Candidate Gene	3356	HTR2A	DB00321	Amitriptyline	N	6	AA09
Candidate Gene	3356	HTR2A	DB00334	Olanzapine	N	5	AH03
Candidate Gene	3356	HTR2A	DB00363	Clozapine	N	5	AH02
Candidate Gene	3356	HTR2A	DB00370	Mirtazapine	N	6	AX11
Candidate Gene	3356	HTR2A	DB00408	Loxapine	N	5	AH01
Candidate Gene	3356	HTR2A	DB00409	Remoxipride	N	5	AL04
Candidate Gene	3356	HTR2A	DB00413	Pramipexole	N	4	BC05
Candidate Gene	3356	HTR2A	DB00420	Promazine	N	5	AA01
Candidate Gene	3356	HTR2A	DB00420	Promazine	N	5	AA02
Candidate Gene	3356	HTR2A	DB00420	Promazine	N	5	AA03
Candidate Gene	3356	HTR2A	DB00420	Promazine	N	5	AA04
Candidate Gene	3356	HTR2A	DB00420	Promazine	N	5	AA05
Candidate Gene	3356	HTR2A	DB00434	Cyproheptadine	R	6	AX02
Candidate Gene	3356	HTR2A	DB08002	Imipramine	N	6	AA02
Candidate Gene	3356	HTR2A	DB08002	Imipramine	N	6	AA03
Candidate Gene	3356	HTR2A	DB08002	Imipramine	N	6	AA06

Candidate Gene	3356	HTR2A	DB00502	Haloperidol	N	5	AD01
Candidate Gene	3356	HTR2A	DB00540	Nortriptyline	N	6	AA10
Candidate Gene	3356	HTR2A	DB00543	Amoxapine	N	6	AA17
Candidate Gene	3356	HTR2A	DB00589	Lisuride	G	2	CB02
Candidate Gene	3356	HTR2A	DB00589	Lisuride	N	2	CA07
Candidate Gene	3356	HTR2A	DB00604	Cisapride	A	3	FA02
Candidate Gene	3356	HTR2A	DB00656	Trazodone	N	6	AX05
Candidate Gene	3356	HTR2A	DB00679	Thioridazine	N	5	AC02
Candidate Gene	3356	HTR2A	DB00696	Ergotamine	N	2	CA02
Candidate Gene	3356	HTR2A	DB00715	Paroxetine	N	6	AB05
Candidate Gene	3356	HTR2A	DB00734	Risperidone	N	5	AX08
Candidate Gene	3356	HTR2A	DB00751	Epinastine	R	6	AX24
Candidate Gene	3356	HTR2A	DB00751	Epinastine	S	1	GX10
Candidate Gene	3356	HTR2A	DB00777	Propiomazine	N	5	CM06
Candidate Gene	3356	HTR2A	DB00805	Minaprine	N	6	AX07
Candidate Gene	3356	HTR2A	DB00843	Donepezil	N	6	DA02
Candidate Gene	3356	HTR2A	DB00924	Cyclobenzaprine	M	3	BX08
Candidate Gene	3356	HTR2A	DB00933	Mesoridazine	N	5	AC03
Candidate Gene	3356	HTR2A	DB00934	Maprotiline	N	6	AA21
Candidate Gene	3356	HTR2A	DB01287	Ergoloid mesylate	C	4	AE01
Candidate Gene	3356	HTR2A	DB01069	Promethazine	D	4	AA10
Candidate Gene	3356	HTR2A	DB01069	Promethazine	R	6	AD02
Candidate Gene	3356	HTR2A	DB01069	Promethazine	R	6	AD05
Candidate Gene	3356	HTR2A	DB01079	Tegaserod	A	6	AX06
Candidate Gene	3356	HTR2A	DB01142	Doxepin	N	6	AA12
Candidate Gene	3356	HTR2A	DB01149	Nefazodone	N	6	AX06
Candidate Gene	3356	HTR2A	DB07682	Desipramine	N	6	AA01
Candidate Gene	3356	HTR2A	DB01186	Pergolide	N	4	BC02
Candidate Gene	3356	HTR2A	DB01200	Bromocriptine	G	2	CB01
Candidate Gene	3356	HTR2A	DB01200	Bromocriptine	N	4	BC01
Candidate Gene	3356	HTR2A	DB01221	Ketamine	N	1	AX03
Candidate Gene	3356	HTR2A	DB01221	Ketamine	N	1	AX14
Candidate Gene	3356	HTR2A	DB01224	Quetiapine	N	5	AH04
Candidate Gene	3356	HTR2A	DB01238	Aripiprazole	N	5	AX12

Candidate Gene	3356	HTR2A	DB01239	Chlorprothixene	N	5	AF03
Candidate Gene	3356	HTR2A	DB07600	Clomipramine	N	6	AA04
Candidate Gene	3356	HTR2A	DB01267	Paliperidone	N	5	AX13
Candidate Gene	3356	HTR2A	DB01392	Yohimbine	G	4	BE04
Candidate Gene	3356	HTR2A	DB01618	Molindone	N	5	AE02
Candidate Gene	3356	HTR2A	DB01621	Pipotiazine	N	5	AC04
Candidate Gene	3356	HTR2A	DB01622	Thioproperazine	N	5	AB08
Candidate Gene	3356	HTR2A	DB01623	Thiothixene	N	5	AF04
Candidate Gene	3356	HTR2A	DB08920	Zuclopenthixol	N	5	AF05
Candidate Gene	3356	HTR2A	DB04599	Aniracetam	N	6	BX11
Candidate Gene	3356	HTR2A	DB04946	Iloperidone	N	5	AX14
Candidate Gene	3356	HTR2A	DB06144	Sertindole	N	5	AE03
Candidate Gene	3356	HTR2A	DB06148	Mianserin	N	6	AX03
Candidate Gene	3356	HTR2A	DB06216	Asenapine	N	5	AH05
Candidate Gene	3356	HTR2A	DB06288	Amisulpride	N	5	AL05
Candidate Gene	3356	HTR2A	DB08815	Lurasidone	N	5	AE05
Candidate Gene	4524	MTHFR	DB00115	Cyanocobalamin	B	3	BA01
Candidate Gene	4524	MTHFR	DB00115	Cyanocobalamin	B	3	BB01
Candidate Gene	6531	SLC6A3	DB00182	Amphetamine	N	6	BA01
Candidate Gene	6531	SLC6A3	DB00191	Phentermine	A	8	AA01
Candidate Gene	6531	SLC6A3	DB00245	Benzatropine	N	4	AC01
Candidate Gene	6531	SLC6A3	DB00285	Venlafaxine	N	6	AX16
Candidate Gene	6531	SLC6A3	DB00285	Venlafaxine	N	6	AX23
Candidate Gene	6531	SLC6A3	DB00289	Atomoxetine	N	6	BA09
Candidate Gene	6531	SLC6A3	DB00370	Mirtazapine	N	6	AX11
Candidate Gene	6531	SLC6A3	DB00408	Loxapine	N	5	AH01
Candidate Gene	6531	SLC6A3	DB00422	Methylphenidate	N	6	BA04
Candidate Gene	6531	SLC6A3	DB00454	Pethidine	N	2	AB02
Candidate Gene	6531	SLC6A3	DB08002	Imipramine	N	6	AA02
Candidate Gene	6531	SLC6A3	DB08002	Imipramine	N	6	AA03
Candidate Gene	6531	SLC6A3	DB08002	Imipramine	N	6	AA06
Candidate Gene	6531	SLC6A3	DB00476	Duloxetine	N	6	AX21
Candidate Gene	6531	SLC6A3	DB00543	Amoxapine	N	6	AA17
Candidate Gene	6531	SLC6A3	DB00579	Mazindol	A	8	AA05

Candidate Gene	6531	SLC6A3	DB00721	Procaine	C	1	BA02
Candidate Gene	6531	SLC6A3	DB00721	Procaine	C	5	AD05
Candidate Gene	6531	SLC6A3	DB00721	Procaine	D	4	AB03
Candidate Gene	6531	SLC6A3	DB00721	Procaine	J	1	CE09
Candidate Gene	6531	SLC6A3	DB00721	Procaine	N	1	BA02
Candidate Gene	6531	SLC6A3	DB00721	Procaine	N	1	BA04
Candidate Gene	6531	SLC6A3	DB00721	Procaine	S	1	HA02
Candidate Gene	6531	SLC6A3	DB00721	Procaine	S	1	HA05
Candidate Gene	6531	SLC6A3	DB00745	Modafinil	N	6	BA07
Candidate Gene	6531	SLC6A3	DB00852	Pseudoephedrine	R	1	BA02
Candidate Gene	6531	SLC6A3	DB00907	Cocaine	N	1	BC01
Candidate Gene	6531	SLC6A3	DB00907	Cocaine	R	2	AD03
Candidate Gene	6531	SLC6A3	DB00907	Cocaine	S	1	HA01
Candidate Gene	6531	SLC6A3	DB00907	Cocaine	S	2	DA02
Candidate Gene	6531	SLC6A3	DB00937	Diethylpropion	A	8	AA03
Candidate Gene	6531	SLC6A3	DB00988	Dopamine	C	1	CA04
Candidate Gene	6531	SLC6A3	DB01104	Sertraline	N	6	AB06
Candidate Gene	6531	SLC6A3	DB01105	Sibutramine	A	8	AA10
Candidate Gene	6531	SLC6A3	DB01114	Chlorphenamine	R	6	AB02
Candidate Gene	6531	SLC6A3	DB01114	Chlorphenamine	R	6	AB04
Candidate Gene	6531	SLC6A3	DB01146	Diphenylpyraline	R	6	AA07
Candidate Gene	6531	SLC6A3	DB01146	Diphenylpyraline	V	1	AA02
Candidate Gene	6531	SLC6A3	DB01146	Diphenylpyraline	V	1	AA20
Candidate Gene	6531	SLC6A3	DB01149	Nefazodone	N	6	AX06
Candidate Gene	6531	SLC6A3	DB01156	Bupropion	N	6	AX12
Candidate Gene	6531	SLC6A3	DB01576	Dextroamphetamine	N	6	BA02
Candidate Gene	6531	SLC6A3	DB01577	Methamphetamine	N	6	BA03
Candidate Gene	6531	SLC6A3	DB04821	Nomifensine	N	6	AX04
Candidate Gene	6531	SLC6A3	DB06148	Mianserin	N	6	AX03
Candidate Gene	6531	SLC6A3	DB06701	Dexmethylphenidate	N	6	BA11
Candidate Gene	6532	SLC6A4	DB00176	Fluvoxamine	N	6	AB08
Candidate Gene	6532	SLC6A4	DB00182	Amphetamine	N	6	BA01
Candidate Gene	6532	SLC6A4	DB00191	Phentermine	A	8	AA01
Candidate Gene	6532	SLC6A4	DB00193	Tramadol	N	2	AX02

Candidate Gene	6532	SLC6A4	DB00215	Citalopram	N	6	AB04
Candidate Gene	6532	SLC6A4	DB00215	Citalopram	N	6	AB10
Candidate Gene	6532	SLC6A4	DB00285	Venlafaxine	N	6	AX16
Candidate Gene	6532	SLC6A4	DB00285	Venlafaxine	N	6	AX23
Candidate Gene	6532	SLC6A4	DB00289	Atomoxetine	N	6	BA09
Candidate Gene	6532	SLC6A4	DB00321	Amitriptyline	N	6	AA09
Candidate Gene	6532	SLC6A4	DB00344	Protriptyline	N	6	AA11
Candidate Gene	6532	SLC6A4	DB00370	Mirtazapine	N	6	AX11
Candidate Gene	6532	SLC6A4	DB00408	Loxapine	N	5	AH01
Candidate Gene	6532	SLC6A4	DB00422	Methylphenidate	N	6	BA04
Candidate Gene	6532	SLC6A4	DB00454	Pethidine	N	2	AB02
Candidate Gene	6532	SLC6A4	DB08002	Imipramine	N	6	AA02
Candidate Gene	6532	SLC6A4	DB08002	Imipramine	N	6	AA03
Candidate Gene	6532	SLC6A4	DB08002	Imipramine	N	6	AA06
Candidate Gene	6532	SLC6A4	DB00472	Fluoxetine	N	6	AB03
Candidate Gene	6532	SLC6A4	DB00476	Duloxetine	N	6	AX21
Candidate Gene	6532	SLC6A4	DB00514	Dextromethorphan	R	5	DA09
Candidate Gene	6532	SLC6A4	DB00540	Nortriptyline	N	6	AA10
Candidate Gene	6532	SLC6A4	DB00543	Amoxapine	N	6	AA17
Candidate Gene	6532	SLC6A4	DB00574	Fenfluramine	A	8	AA02
Candidate Gene	6532	SLC6A4	DB00574	Fenfluramine	A	8	AA04
Candidate Gene	6532	SLC6A4	DB00579	Mazindol	A	8	AA05
Candidate Gene	6532	SLC6A4	DB00656	Trazodone	N	6	AX05
Candidate Gene	6532	SLC6A4	DB00661	Verapamil	C	8	DA01
Candidate Gene	6532	SLC6A4	DB00715	Paroxetine	N	6	AB05
Candidate Gene	6532	SLC6A4	DB00805	Minaprine	N	6	AX07
Candidate Gene	6532	SLC6A4	DB00852	Pseudoephedrine	R	1	BA02
Candidate Gene	6532	SLC6A4	DB00907	Cocaine	N	1	BC01
Candidate Gene	6532	SLC6A4	DB00907	Cocaine	R	2	AD03
Candidate Gene	6532	SLC6A4	DB00907	Cocaine	S	1	HA01
Candidate Gene	6532	SLC6A4	DB00907	Cocaine	S	2	DA02
Candidate Gene	6532	SLC6A4	DB00988	Dopamine	C	1	CA04
Candidate Gene	6532	SLC6A4	DB01104	Sertraline	N	6	AB06
Candidate Gene	6532	SLC6A4	DB01105	Sibutramine	A	8	AA10

Candidate Gene	6532	SLC6A4	DB01114	Chlorphenamine	R	6	AB02
Candidate Gene	6532	SLC6A4	DB01114	Chlorphenamine	R	6	AB04
Candidate Gene	6532	SLC6A4	DB01142	Doxepin	N	6	AA12
Candidate Gene	6532	SLC6A4	DB01149	Nefazodone	N	6	AX06
Candidate Gene	6532	SLC6A4	DB07682	Desipramine	N	6	AA01
Candidate Gene	6532	SLC6A4	DB07600	Clomipramine	N	6	AA04
Candidate Gene	6532	SLC6A4	DB01577	Methamphetamine	N	6	BA03
Candidate Gene	6532	SLC6A4	DB04832	Zimelidine	N	6	AB02
Candidate Gene	6532	SLC6A4	DB04836	Amineptine	N	6	AA19
Candidate Gene	6532	SLC6A4	DB04896	Milnacipran	N	6	AX17
Candidate Gene	6532	SLC6A4	DB06148	Mianserin	N	6	AX03
Candidate Gene	6532	SLC6A4	DB06204	Tapentadol	N	2	AX06
Candidate Gene	6532	SLC6A4	DB06701	Dexmethylphenidate	N	6	BA11
Candidate Gene	7124	TNF	DB00005	Etanercept	L	4	AB01
Candidate Gene	7124	TNF	DB00051	Adalimumab	L	4	AB04
Candidate Gene	7124	TNF	DB00065	Infliximab	L	4	AB02
Candidate Gene	7124	TNF	DB00608	Chloroquine	P	1	BA01
Candidate Gene	7124	TNF	DB00608	Chloroquine	P	1	BA02
Candidate Gene	7124	TNF	DB00668	Epinephrine	A	1	AD01
Candidate Gene	7124	TNF	DB00668	Epinephrine	B	2	BC09
Candidate Gene	7124	TNF	DB00668	Epinephrine	C	1	CA24
Candidate Gene	7124	TNF	DB00668	Epinephrine	R	1	AA14
Candidate Gene	7124	TNF	DB00668	Epinephrine	R	3	AA01
Candidate Gene	7124	TNF	DB00668	Epinephrine	S	1	EA01
Candidate Gene	7124	TNF	DB00852	Pseudoephedrine	R	1	BA02
Candidate Gene	7124	TNF	DB01041	Thalidomide	L	4	AX02
Candidate Gene	7124	TNF	DB01407	Clenbuterol	R	3	AC14
Candidate Gene	7124	TNF	DB01407	Clenbuterol	R	3	CC13
Candidate Gene	7124	TNF	DB01411	Pranlukast	R	3	DC02
Candidate Gene	7124	TNF	DB01427	Amrinone	C	1	CE01
Candidate Gene	7124	TNF	DB04956	Afelimomab	L	4	AB03
Candidate Gene	7124	TNF	DB06674	golimumab	L	4	AB06
Candidate Gene	7124	TNF	DB08904	Certolizumab pegol	L	4	AB05
Known Interactor	2	A2M	DB00102	Becaplermin	A	1	AD08

Known Interactor	2	A2M	DB00102	Becaplermin	D	3	AX06
Known Interactor	2	A2M	DB00626	Bacitracin	D	6	AX05
Known Interactor	2	A2M	DB00626	Bacitracin	J	1	XX10
Known Interactor	2	A2M	DB00626	Bacitracin	R	2	AB04
Known Interactor	1636	ACE	DB00178	Ramipril	C	9	AA05
Known Interactor	1636	ACE	DB00492	Fosinopril	C	9	AA09
Known Interactor	1636	ACE	DB00519	Trandolapril	C	9	AA10
Known Interactor	1636	ACE	DB00542	Benazepril	C	9	AA07
Known Interactor	1636	ACE	DB00584	Enalapril	C	9	AA02
Known Interactor	1636	ACE	DB00691	Moexipril	C	9	AA13
Known Interactor	1636	ACE	DB00790	Perindopril	C	9	AA04
Known Interactor	1636	ACE	DB00881	Quinapril	C	9	AA06
Known Interactor	1636	ACE	DB01180	Rescinnamine	C	2	AA01
Known Interactor	1636	ACE	DB01197	Captopril	C	9	AA01
Known Interactor	1636	ACE	DB01340	Cilazapril	C	9	AA08
Known Interactor	1636	ACE	DB01348	Spirapril	C	9	AA11
Known Interactor	1636	ACE	DB08836	Temocapril	C	9	AA14
Known Interactor	135	ADORA2A	DB00201	Caffeine	N	2	BE01
Known Interactor	135	ADORA2A	DB00201	Caffeine	N	6	BC01
Known Interactor	135	ADORA2A	DB00277	Theophylline	R	3	DA04
Known Interactor	135	ADORA2A	DB00277	Theophylline	R	3	DA05
Known Interactor	135	ADORA2A	DB00358	Mefloquine	P	1	BC02
Known Interactor	135	ADORA2A	DB00640	Adenosine	C	1	EB10
Known Interactor	135	ADORA2A	DB00651	Dyphylline	R	3	DA01
Known Interactor	135	ADORA2A	DB00806	Pentoxifylline	C	4	AD03
Known Interactor	135	ADORA2A	DB01412	Theobromine	C	3	BD01
Known Interactor	135	ADORA2A	DB01412	Theobromine	R	3	DA07
Known Interactor	135	ADORA2A	DB04932	Defibrotide	B	1	AX01
Known Interactor	135	ADORA2A	DB06213	Regadenoson	C	1	EB21
Known Interactor	26	AOC1	DB00594	Amiloride	C	3	DB01
Known Interactor	367	AR	DB00506	Levonorgestrel	G	3	AC03
Known Interactor	367	AR	DB00506	Levonorgestrel	G	3	AD01
Known Interactor	367	AR	DB00421	Spironolactone	C	3	DA01
Known Interactor	367	AR	DB00499	Flutamide	L	2	BB01

Known Interactor	367	AR	DB00621	Oxandrolone	A	14	AA08
Known Interactor	367	AR	DB00624	Testosterone	G	3	BA02
Known Interactor	367	AR	DB00624	Testosterone	G	3	BA03
Known Interactor	367	AR	DB00624	Testosterone	G	3	EK01
Known Interactor	367	AR	DB00665	Nilutamide	L	2	BB02
Known Interactor	367	AR	DB02478	Fludrocortisone	H	2	AA02
Known Interactor	367	AR	DB00984	Nandrolone phenpropionate	A	14	AB01
Known Interactor	367	AR	DB00984	Nandrolone phenpropionate	S	1	XA11
Known Interactor	367	AR	DB01026	Ketoconazole	D	1	AC08
Known Interactor	367	AR	DB01026	Ketoconazole	G	1	AF11
Known Interactor	367	AR	DB01026	Ketoconazole	J	2	AB02
Known Interactor	367	AR	DB06284	Bicalutamide	L	2	BB03
Known Interactor	367	AR	DB01185	Fluoxymesterone	G	3	BA01
Known Interactor	367	AR	DB01406	Danazol	G	3	XA01
Known Interactor	367	AR	DB02266	Flufenamic Acid	M	1	AG03
Known Interactor	367	AR	DB02901	Dihydrotestosterone	A	14	AA01
Known Interactor	367	AR	DB02901	Dihydrotestosterone	G	3	BB02
Known Interactor	367	AR	DB04839	Cyproterone acetate	G	3	HA01
Known Interactor	596	BCL2	DB01050	Ibuprofen	C	1	EB16
Known Interactor	596	BCL2	DB01050	Ibuprofen	G	2	CC01
Known Interactor	596	BCL2	DB01050	Ibuprofen	M	1	AE01
Known Interactor	596	BCL2	DB01050	Ibuprofen	M	1	AE14
Known Interactor	596	BCL2	DB01050	Ibuprofen	M	2	AA13
Known Interactor	596	BCL2	DB05927	Paclitaxel	L	1	CD01
Known Interactor	596	BCL2	DB01248	Docetaxel	L	1	CD02
Known Interactor	596	BCL2	DB01367	Rasagiline	N	4	BD02
Known Interactor	596	BCL2	DB05297	DHA-paclitaxel	L	4	AA26
Known Interactor	673	BRAF	DB07438	Sorafenib	L	1	XE05
Known Interactor	673	BRAF	DB08881	Vemurafenib	L	1	XE15
Known Interactor	673	BRAF	DB08896	Regorafenib	L	1	XE21
Known Interactor	801	CALM1	DB00527	Cinchocaine	C	5	AD04
Known Interactor	801	CALM1	DB00527	Cinchocaine	D	4	AB02
Known Interactor	801	CALM1	DB00527	Cinchocaine	N	1	BB06
Known Interactor	801	CALM1	DB00527	Cinchocaine	S	1	HA06

Known Interactor	801	CALM1	DB00527	Cinchocaine	S	2	DA04
Known Interactor	801	CALM1	DB00622	Nicardipine	C	8	CA04
Known Interactor	801	CALM1	DB00623	Fluphenazine	N	5	AB02
Known Interactor	801	CALM1	DB00753	Isoflurane	N	1	AB06
Known Interactor	801	CALM1	DB08616	Trifluoperazine	N	5	AB06
Known Interactor	801	CALM1	DB00836	Loperamide	A	7	DA03
Known Interactor	801	CALM1	DB00836	Loperamide	A	7	DA05
Known Interactor	801	CALM1	DB00850	Perphenazine	N	5	AB03
Known Interactor	801	CALM1	DB00925	Phenoxybenzamine	C	4	AX02
Known Interactor	801	CALM1	DB01023	Felodipine	C	8	CA02
Known Interactor	801	CALM1	DB08189	Melatonin	N	5	CH01
Known Interactor	801	CALM1	DB01069	Promethazine	D	4	AA10
Known Interactor	801	CALM1	DB01069	Promethazine	R	6	AD02
Known Interactor	801	CALM1	DB01069	Promethazine	R	6	AD05
Known Interactor	801	CALM1	DB01100	Pimozide	N	5	AG02
Known Interactor	801	CALM1	DB01115	Nifedipine	C	8	CA05
Known Interactor	801	CALM1	DB01244	Bepridil	C	8	EA02
Known Interactor	801	CALM1	DB01373	Calcium	A	2	AC01
Known Interactor	801	CALM1	DB01373	Calcium	A	2	AC02
Known Interactor	801	CALM1	DB01373	Calcium	A	6	AC08
Known Interactor	801	CALM1	DB01373	Calcium	A	7	XA03
Known Interactor	801	CALM1	DB01373	Calcium	A	11	HA31
Known Interactor	801	CALM1	DB01373	Calcium	A	12	AA01
Known Interactor	801	CALM1	DB01373	Calcium	A	12	AA02
Known Interactor	801	CALM1	DB01373	Calcium	A	12	AA03
Known Interactor	801	CALM1	DB01373	Calcium	A	12	AA04
Known Interactor	801	CALM1	DB01373	Calcium	A	12	AA05
Known Interactor	801	CALM1	DB01373	Calcium	A	12	AA06
Known Interactor	801	CALM1	DB01373	Calcium	A	12	AA08
Known Interactor	801	CALM1	DB01373	Calcium	A	12	AA09
Known Interactor	801	CALM1	DB01373	Calcium	A	12	AA10
Known Interactor	801	CALM1	DB01373	Calcium	A	12	AA11
Known Interactor	801	CALM1	DB01373	Calcium	A	12	AA20
Known Interactor	801	CALM1	DB01373	Calcium	A	12	AA30

Known Interactor	801	CALM1	DB01373	Calcium	B	1	AC08
Known Interactor	801	CALM1	DB01373	Calcium	B	2	BC08
Known Interactor	801	CALM1	DB01373	Calcium	C	5	BX01
Known Interactor	801	CALM1	DB01373	Calcium	D	3	AX04
Known Interactor	801	CALM1	DB01373	Calcium	D	11	AX03
Known Interactor	801	CALM1	DB01373	Calcium	J	4	AA03
Known Interactor	801	CALM1	DB01373	Calcium	N	2	BA15
Known Interactor	801	CALM1	DB01373	Calcium	N	7	BB02
Known Interactor	801	CALM1	DB01373	Calcium	R	1	AX01
Known Interactor	801	CALM1	DB01373	Calcium	V	3	AE01
Known Interactor	801	CALM1	DB01373	Calcium	V	3	AF04
Known Interactor	801	CALM1	DB01373	Calcium	V	8	AC10
Known Interactor	801	CALM1	DB01429	Aprindine	C	1	BB04
Known Interactor	801	CALM1	DB04825	Prenylamine	C	1	DX02
Known Interactor	801	CALM1	DB04841	Flunarizine	N	7	CA03
Known Interactor	811	CALR	DB00025	Antihemophilic Factor	B	2	BD02
Known Interactor	811	CALR	DB00031	Tenecteplase	B	1	AD11
Known Interactor	811	CALR	DB08189	Melatonin	N	5	CH01
Known Interactor	821	CANX	DB00025	Antihemophilic Factor	B	2	BD02
Known Interactor	821	CANX	DB00031	Tenecteplase	B	1	AD11
Known Interactor	836	CASP3	DB01017	Minocycline	A	1	AB23
Known Interactor	836	CASP3	DB01017	Minocycline	J	1	AA08
Known Interactor	1363	CPE	DB01383	Insulin Regular	A	10	AB01
Known Interactor	1363	CPE	DB01383	Insulin Regular	A	10	AB03
Known Interactor	1363	CPE	DB01383	Insulin Regular	A	10	AB04
Known Interactor	1363	CPE	DB01383	Insulin Regular	A	10	AB05
Known Interactor	1363	CPE	DB01383	Insulin Regular	A	10	AC01
Known Interactor	1363	CPE	DB01383	Insulin Regular	A	10	AC03
Known Interactor	1363	CPE	DB01383	Insulin Regular	A	10	AD05
Known Interactor	1363	CPE	DB01383	Insulin Regular	A	10	AE04
Known Interactor	1363	CPE	DB01383	Insulin Regular	A	10	AE05
Known Interactor	1385	CREB1	DB01183	Naloxone	V	3	AB15
Known Interactor	1786	DNMT1	DB01099	Flucytosine	D	1	AE21
Known Interactor	1786	DNMT1	DB01099	Flucytosine	J	2	AX01

Known Interactor	1950	EGF	DB00364	Sucralfate	A	2	BX02
Known Interactor	1956	EGFR	DB00002	Cetuximab	L	1	XC06
Known Interactor	1956	EGFR	DB00072	Trastuzumab	L	1	XC03
Known Interactor	1956	EGFR	DB05291	Lidocaine	A	1	AD11
Known Interactor	1956	EGFR	DB05291	Lidocaine	C	1	BB01
Known Interactor	1956	EGFR	DB05291	Lidocaine	C	5	AD01
Known Interactor	1956	EGFR	DB05291	Lidocaine	D	4	AB01
Known Interactor	1956	EGFR	DB05291	Lidocaine	N	1	BB02
Known Interactor	1956	EGFR	DB05291	Lidocaine	R	2	AD02
Known Interactor	1956	EGFR	DB05291	Lidocaine	S	1	HA07
Known Interactor	1956	EGFR	DB05291	Lidocaine	S	2	DA01
Known Interactor	1956	EGFR	DB07998	Gefitinib	L	1	XE02
Known Interactor	1956	EGFR	DB00530	Erlotinib	L	1	XE03
Known Interactor	1956	EGFR	DB02584	Lapatinib	L	1	XE07
Known Interactor	1956	EGFR	DB01269	Panitumumab	L	1	XC08
Known Interactor	1956	EGFR	DB05294	Vandetanib	L	1	XE12
Known Interactor	1956	EGFR	DB08916	Afatinib	L	1	XE13
Known Interactor	2064	ERBB2	DB00072	Trastuzumab	L	1	XC03
Known Interactor	2064	ERBB2	DB02584	Lapatinib	L	1	XE07
Known Interactor	2064	ERBB2	DB06366	Pertuzumab	L	1	XC13
Known Interactor	2064	ERBB2	DB08916	Afatinib	L	1	XE13
Known Interactor	2099	ESR1	DB00255	Diethylstilbestrol	G	3	CB02
Known Interactor	2099	ESR1	DB00255	Diethylstilbestrol	G	3	CC05
Known Interactor	2099	ESR1	DB00255	Diethylstilbestrol	L	2	AA01
Known Interactor	2099	ESR1	DB00269	Chlorotrianisene	G	3	CA06
Known Interactor	2099	ESR1	DB00286	Conjugated Estrogens	G	3	CA57
Known Interactor	2099	ESR1	DB00294	Etonogestrel	G	3	AC08
Known Interactor	2099	ESR1	DB00304	Desogestrel	G	3	AC09
Known Interactor	2099	ESR1	DB00506	Levonorgestrel	G	3	AC03
Known Interactor	2099	ESR1	DB00506	Levonorgestrel	G	3	AD01
Known Interactor	2099	ESR1	DB00396	Progesterone	G	3	AC06
Known Interactor	2099	ESR1	DB00396	Progesterone	G	3	DA02
Known Interactor	2099	ESR1	DB00396	Progesterone	G	3	DA03
Known Interactor	2099	ESR1	DB00396	Progesterone	G	3	DA04

Known Interactor	2099	ESR1	DB00396	Progesterone	L	2	AB02
Known Interactor	2099	ESR1	DB00481	Raloxifene	G	3	XC01
Known Interactor	2099	ESR1	DB00539	Toremifene	L	2	BA02
Known Interactor	2099	ESR1	DB00655	Estrone	G	3	CA07
Known Interactor	2099	ESR1	DB00655	Estrone	G	3	CC04
Known Interactor	2099	ESR1	DB00675	Tamoxifen	L	2	BA01
Known Interactor	2099	ESR1	DB00783	Estradiol	G	3	CA01
Known Interactor	2099	ESR1	DB00783	Estradiol	G	3	CA03
Known Interactor	2099	ESR1	DB00783	Estradiol	L	2	AA02
Known Interactor	2099	ESR1	DB00783	Estradiol	L	2	AA03
Known Interactor	2099	ESR1	DB00882	Clomifene	G	3	GB02
Known Interactor	2099	ESR1	DB00890	Dienestrol	G	3	CB01
Known Interactor	2099	ESR1	DB00890	Dienestrol	G	3	CC02
Known Interactor	2099	ESR1	DB00947	Fulvestrant	L	2	BA03
Known Interactor	2099	ESR1	DB00957	Norgestimate	G	3	AA11
Known Interactor	2099	ESR1	DB08189	Melatonin	N	5	CH01
Known Interactor	2099	ESR1	DB01108	Trilostane	H	2	CA01
Known Interactor	2099	ESR1	DB01183	Naloxone	V	3	AB15
Known Interactor	2099	ESR1	DB01185	Fluoxymesterone	G	3	BA01
Known Interactor	2099	ESR1	DB01196	Estramustine	L	1	XX11
Known Interactor	2099	ESR1	DB01406	Danazol	G	3	XA01
Known Interactor	2099	ESR1	DB01431	Allylestrenol	G	3	DC01
Known Interactor	2099	ESR1	DB06593	Dehydroepiandrosterone	A	14	AA07
Known Interactor	2099	ESR1	DB05375	Estriol	G	3	CA04
Known Interactor	2099	ESR1	DB05375	Estriol	G	3	CC06
Known Interactor	2100	ESR2	DB00255	Diethylstilbestrol	G	3	CB02
Known Interactor	2100	ESR2	DB00255	Diethylstilbestrol	G	3	CC05
Known Interactor	2100	ESR2	DB00255	Diethylstilbestrol	L	2	AA01
Known Interactor	2100	ESR2	DB00481	Raloxifene	G	3	XC01
Known Interactor	2100	ESR2	DB00675	Tamoxifen	L	2	BA01
Known Interactor	2100	ESR2	DB00783	Estradiol	G	3	CA01
Known Interactor	2100	ESR2	DB00783	Estradiol	G	3	CA03
Known Interactor	2100	ESR2	DB00783	Estradiol	L	2	AA02
Known Interactor	2100	ESR2	DB00783	Estradiol	L	2	AA03

Known Interactor	2100	ESR2	DB01108	Trilostane	H	2	CA01
Known Interactor	2100	ESR2	DB01196	Estramustine	L	1	XX11
Known Interactor	2100	ESR2	DB06593	Dehydroepiandrosterone	A	14	AA07
Known Interactor	2100	ESR2	DB05375	Estriol	G	3	CA04
Known Interactor	2100	ESR2	DB05375	Estriol	G	3	CC06
Known Interactor	2534	FYN	DB01254	Dasatinib	L	1	XE06
Known Interactor	2550	GABBR1	DB00181	Baclofen	M	3	BX01
Known Interactor	2550	GABBR1	DB00837	Progabide	N	3	AG05
Known Interactor	2550	GABBR1	DB01080	Vigabatrin	N	3	AG04
Known Interactor	2554	GABRA1	DB00186	Lorazepam	N	5	BA06
Known Interactor	2554	GABRA1	DB00189	Ethchlorvynol	N	5	CM08
Known Interactor	2554	GABRA1	DB00228	Enflurane	N	1	AB04
Known Interactor	2554	GABRA1	DB00231	Temazepam	N	5	CD07
Known Interactor	2554	GABRA1	DB00273	Topiramate	N	3	AX11
Known Interactor	2554	GABRA1	DB00292	Etomidate	N	1	AX07
Known Interactor	2554	GABRA1	DB00306	Talbutal	N	5	CA07
Known Interactor	2554	GABRA1	DB00312	Pentobarbital	N	5	CA01
Known Interactor	2554	GABRA1	DB00334	Olanzapine	N	5	AH03
Known Interactor	2554	GABRA1	DB00349	Clobazam	N	5	BA09
Known Interactor	2554	GABRA1	DB00371	Meprobamate	N	5	BC01
Known Interactor	2554	GABRA1	DB00402	Eszopiclone	N	5	CF04
Known Interactor	2554	GABRA1	DB00404	Alprazolam	N	5	BA12
Known Interactor	2554	GABRA1	DB00418	Secobarbital	N	5	CA06
Known Interactor	2554	GABRA1	DB00425	Zolpidem	N	5	CF02
Known Interactor	2554	GABRA1	DB00463	Metharbital	N	3	AA30
Known Interactor	2554	GABRA1	DB00474	Methohexital	N	1	AF01
Known Interactor	2554	GABRA1	DB00474	Methohexital	N	5	CA15
Known Interactor	2554	GABRA1	DB00475	Chlordiazepoxide	N	5	BA02
Known Interactor	2554	GABRA1	DB00543	Amoxapine	N	6	AA17
Known Interactor	2554	GABRA1	DB00546	Adinazolam	N	5	BA07
Known Interactor	2554	GABRA1	DB00599	Thiopental	N	1	AF03
Known Interactor	2554	GABRA1	DB00599	Thiopental	N	5	CA19
Known Interactor	2554	GABRA1	DB00628	Clorazepate	N	5	BA05
Known Interactor	2554	GABRA1	DB00659	Acamprosate	N	7	BB03

Known Interactor	2554	GABRA1	DB00683	Midazolam	N	5	CD08
Known Interactor	2554	GABRA1	DB00690	Flurazepam	N	5	CD01
Known Interactor	2554	GABRA1	DB00753	Isoflurane	N	1	AB06
Known Interactor	2554	GABRA1	DB00794	Primidone	D	8	AC04
Known Interactor	2554	GABRA1	DB00794	Primidone	N	3	AA03
Known Interactor	2554	GABRA1	DB00794	Primidone	R	1	AX07
Known Interactor	2554	GABRA1	DB00794	Primidone	R	2	AA18
Known Interactor	2554	GABRA1	DB00794	Primidone	S	1	AX08
Known Interactor	2554	GABRA1	DB00794	Primidone	S	3	AA05
Known Interactor	2554	GABRA1	DB00801	Halazepam	N	5	BA13
Known Interactor	2554	GABRA1	DB05893	Propofol	N	1	AX10
Known Interactor	2554	GABRA1	DB07699	Diazepam	N	5	BA01
Known Interactor	2554	GABRA1	DB07699	Diazepam	N	5	BA17
Known Interactor	2554	GABRA1	DB00837	Progabide	N	3	AG05
Known Interactor	2554	GABRA1	DB00842	Oxazepam	N	5	BA04
Known Interactor	2554	GABRA1	DB00849	Methylphenobarbital	N	3	AA01
Known Interactor	2554	GABRA1	DB00897	Triazolam	N	5	CD05
Known Interactor	2554	GABRA1	DB00898	Ethanol	D	1	AE06
Known Interactor	2554	GABRA1	DB00898	Ethanol	D	8	AX08
Known Interactor	2554	GABRA1	DB00898	Ethanol	V	3	AB16
Known Interactor	2554	GABRA1	DB00898	Ethanol	V	3	AZ01
Known Interactor	2554	GABRA1	DB00962	Zaleplon	N	5	CF03
Known Interactor	2554	GABRA1	DB01028	Methoxyflurane	N	2	BG09
Known Interactor	2554	GABRA1	DB01287	Ergoloid mesylate	C	4	AE01
Known Interactor	2554	GABRA1	DB01068	Clonazepam	N	3	AE01
Known Interactor	2554	GABRA1	DB01107	Methyprylon	N	5	CE02
Known Interactor	2554	GABRA1	DB02330	Halothane	N	1	AB01
Known Interactor	2554	GABRA1	DB01174	Phenobarbital	N	3	AA02
Known Interactor	2554	GABRA1	DB01189	Desflurane	N	1	AB07
Known Interactor	2554	GABRA1	DB01198	Zopiclone	N	5	CF01
Known Interactor	2554	GABRA1	DB01205	Flumazenil	V	3	AB25
Known Interactor	2554	GABRA1	DB01215	Estazolam	N	5	CD04
Known Interactor	2554	GABRA1	DB01236	Sevoflurane	N	1	AB08
Known Interactor	2554	GABRA1	DB01351	Amobarbital	N	5	CA02

Known Interactor	2554	GABRA1	DB01352	Aprobarbital	N	5	CA05
Known Interactor	2554	GABRA1	DB01354	Heptabarbital	N	5	CA11
Known Interactor	2554	GABRA1	DB01355	Hexobarbital	N	1	AF02
Known Interactor	2554	GABRA1	DB01355	Hexobarbital	N	5	CA16
Known Interactor	2554	GABRA1	DB01381	Ginkgo biloba	N	6	DX02
Known Interactor	2554	GABRA1	DB01437	Glutethimide	N	5	CE01
Known Interactor	2554	GABRA1	DB01558	Bromazepam	N	5	BA08
Known Interactor	2554	GABRA1	DB01587	Ketazolam	N	5	BA10
Known Interactor	2554	GABRA1	DB01588	Prazepam	N	5	BA11
Known Interactor	2554	GABRA1	DB01589	Quazepam	N	5	CD10
Known Interactor	2554	GABRA1	DB01594	Cinolazepam	N	5	CD13
Known Interactor	2554	GABRA1	DB01595	Nitrazepam	N	5	CD02
Known Interactor	2554	GABRA1	DB06593	Dehydroepiandrosterone	A	14	AA07
Known Interactor	2752	GLUL	DB00130	L-Glutamine	A	16	AA03
Known Interactor	2752	GLUL	DB00142	L-Glutamic Acid	A	9	AB01
Known Interactor	2891	GRIA2	DB00142	L-Glutamic Acid	A	9	AB01
Known Interactor	2891	GRIA2	DB00306	Talbutal	N	5	CA07
Known Interactor	2891	GRIA2	DB00312	Pentobarbital	N	5	CA01
Known Interactor	2891	GRIA2	DB00418	Secobarbital	N	5	CA06
Known Interactor	2891	GRIA2	DB00463	Metharbital	N	3	AA30
Known Interactor	2891	GRIA2	DB00599	Thiopental	N	1	AF03
Known Interactor	2891	GRIA2	DB00599	Thiopental	N	5	CA19
Known Interactor	2891	GRIA2	DB00794	Primidone	D	8	AC04
Known Interactor	2891	GRIA2	DB00794	Primidone	N	3	AA03
Known Interactor	2891	GRIA2	DB00794	Primidone	R	1	AX07
Known Interactor	2891	GRIA2	DB00794	Primidone	R	2	AA18
Known Interactor	2891	GRIA2	DB00794	Primidone	S	1	AX08
Known Interactor	2891	GRIA2	DB00794	Primidone	S	3	AA05
Known Interactor	2891	GRIA2	DB00849	Methylphenobarbital	N	3	AA01
Known Interactor	2891	GRIA2	DB01174	Phenobarbital	N	3	AA02
Known Interactor	2891	GRIA2	DB01351	Amobarbital	N	5	CA02
Known Interactor	2891	GRIA2	DB01352	Aprobarbital	N	5	CA05
Known Interactor	2891	GRIA2	DB01354	Heptabarbital	N	5	CA11
Known Interactor	2891	GRIA2	DB01355	Hexobarbital	N	1	AF02

Known Interactor	2891	GRIA2	DB01355	Hexobarbital	N	5	CA16
Known Interactor	2891	GRIA2	DB04599	Aniracetam	N	6	BX11
Known Interactor	2932	GSK3B	DB01356	Lithium	D	11	AX04
Known Interactor	2932	GSK3B	DB01356	Lithium	N	5	AN01
Known Interactor	3320	HSP90AA1	DB00615	Rifabutin	J	4	AB04
Known Interactor	3320	HSP90AA1	DB00716	Nedocromil	R	1	AC07
Known Interactor	3320	HSP90AA1	DB00716	Nedocromil	R	3	BC03
Known Interactor	3320	HSP90AA1	DB00716	Nedocromil	S	1	GX04
Known Interactor	3458	IFNG	DB01250	Olsalazine	A	7	EC03
Known Interactor	3551	IKBKB	DB00244	Mesalazine	A	7	EC02
Known Interactor	3551	IKBKB	DB08518	Sulfasalazine	A	7	EC01
Known Interactor	3551	IKBKB	DB00945	Acetylsalicylic acid	A	1	AD05
Known Interactor	3551	IKBKB	DB00945	Acetylsalicylic acid	B	1	AC06
Known Interactor	3551	IKBKB	DB00945	Acetylsalicylic acid	N	2	BA01
Known Interactor	3551	IKBKB	DB00995	Auranofin	M	1	CB03
Known Interactor	3551	IKBKB	DB01169	Arsenic trioxide	L	1	XX27
Known Interactor	3615	IMPDH2	DB00688	Mycophenolate mofetil	L	4	AA06
Known Interactor	3615	IMPDH2	DB01033	Mercaptopurine	L	1	BB02
Known Interactor	3690	ITGB3	DB00054	Abciximab	B	1	AC13
Known Interactor	3690	ITGB3	DB00063	Eptifibatide	B	1	AC16
Known Interactor	3690	ITGB3	DB00775	Tirofiban	B	1	AC17
Known Interactor	3717	JAK2	DB08877	Ruxolitinib	L	1	XE18
Known Interactor	3717	JAK2	DB08895	Tofacitinib	L	4	AA29
Known Interactor	3763	KCNJ6	DB02330	Halothane	N	1	AB01
Known Interactor	56479	KCNQ5	DB04953	Ezogabine	N	3	AX21
Known Interactor	3949	LDLR	DB00707	Porfimer	L	1	XD01
Known Interactor	4035	LRP1	DB00025	Antihemophilic Factor	B	2	BD02
Known Interactor	4035	LRP1	DB00031	Tenecteplase	B	1	AD11
Known Interactor	4035	LRP1	DB00100	Coagulation Factor IX	B	2	BD04
Known Interactor	4036	LRP2	DB00013	Urokinase	B	1	AD04
Known Interactor	4036	LRP2	DB01383	Insulin Regular	A	10	AB01
Known Interactor	4036	LRP2	DB01383	Insulin Regular	A	10	AB03
Known Interactor	4036	LRP2	DB01383	Insulin Regular	A	10	AB04
Known Interactor	4036	LRP2	DB01383	Insulin Regular	A	10	AB05

Known Interactor	4036	LRP2	DB01383	Insulin Regular	A	10	AC01
Known Interactor	4036	LRP2	DB01383	Insulin Regular	A	10	AC03
Known Interactor	4036	LRP2	DB01383	Insulin Regular	A	10	AD05
Known Interactor	4036	LRP2	DB01383	Insulin Regular	A	10	AE04
Known Interactor	4036	LRP2	DB01383	Insulin Regular	A	10	AE05
Known Interactor	4036	LRP2	DB00798	Gentamicin	D	6	AX07
Known Interactor	4036	LRP2	DB00798	Gentamicin	J	1	GB03
Known Interactor	4036	LRP2	DB00798	Gentamicin	S	1	AA11
Known Interactor	4036	LRP2	DB00798	Gentamicin	S	2	AA14
Known Interactor	4036	LRP2	DB00798	Gentamicin	S	3	AA06
Known Interactor	4130	MAP1A	DB01196	Estramustine	L	1	XX11
Known Interactor	4133	MAP2	DB01196	Estramustine	L	1	XX11
Known Interactor	4133	MAP2	DB05927	Paclitaxel	L	1	CD01
Known Interactor	4133	MAP2	DB01248	Docetaxel	L	1	CD02
Known Interactor	4137	MAPT	DB05927	Paclitaxel	L	1	CD01
Known Interactor	4137	MAPT	DB01248	Docetaxel	L	1	CD02
Known Interactor	2206	MS4A2	DB00043	Omalizumab	R	3	DX05
Known Interactor	2475	MTOR	DB00337	Pimecrolimus	D	11	AH02
Known Interactor	2475	MTOR	DB02439	Sirolimus	L	4	AA10
Known Interactor	2475	MTOR	DB01590	Everolimus	L	1	XE10
Known Interactor	2475	MTOR	DB01590	Everolimus	L	4	AA18
Known Interactor	2475	MTOR	DB06287	Temsirolimus	L	1	XE09
Known Interactor	4792	NFKBIA	DB00945	Acetylsalicylic acid	A	1	AD05
Known Interactor	4792	NFKBIA	DB00945	Acetylsalicylic acid	B	1	AC06
Known Interactor	4792	NFKBIA	DB00945	Acetylsalicylic acid	N	2	BA01
Known Interactor	4846	NOS3	DB00125	L-Arginine	B	5	XB01
Known Interactor	4846	NOS3	DB00360	Tetrahydrobiopterin	A	16	AX07
Known Interactor	4846	NOS3	DB01110	Miconazole	A	1	AB09
Known Interactor	4846	NOS3	DB01110	Miconazole	A	7	AC01
Known Interactor	4846	NOS3	DB01110	Miconazole	D	1	AC02
Known Interactor	4846	NOS3	DB01110	Miconazole	G	1	AF04
Known Interactor	4846	NOS3	DB01110	Miconazole	J	2	AB01
Known Interactor	4846	NOS3	DB01110	Miconazole	S	2	AA13
Known Interactor	5140	PDE3B	DB00201	Caffeine	N	2	BE01

Known Interactor	5140	PDE3B	DB00201	Caffeine	N	6	BC01
Known Interactor	5142	PDE4B	DB00201	Caffeine	N	2	BE01
Known Interactor	5142	PDE4B	DB00201	Caffeine	N	6	BC01
Known Interactor	5142	PDE4B	DB00277	Theophylline	R	3	DA04
Known Interactor	5142	PDE4B	DB00277	Theophylline	R	3	DA05
Known Interactor	5142	PDE4B	DB00651	Dyphylline	R	3	DA01
Known Interactor	5142	PDE4B	DB00806	Pentoxifylline	C	4	AD03
Known Interactor	5142	PDE4B	DB00920	Ketotifen	R	6	AX17
Known Interactor	5142	PDE4B	DB00920	Ketotifen	S	1	GX08
Known Interactor	5142	PDE4B	DB01088	Iloprost	B	1	AC11
Known Interactor	5142	PDE4B	DB07725	Papaverine	A	3	AD01
Known Interactor	5142	PDE4B	DB07725	Papaverine	G	4	BE02
Known Interactor	5142	PDE4B	DB01412	Theobromine	C	3	BD01
Known Interactor	5142	PDE4B	DB01412	Theobromine	R	3	DA07
Known Interactor	5142	PDE4B	DB01427	Amrinone	C	1	CE01
Known Interactor	5142	PDE4B	DB01656	Roflumilast	R	3	DX07
Known Interactor	5142	PDE4B	DB05266	Ibudilast	R	3	DC04
Known Interactor	5170	PDPK1	DB00482	Celecoxib	L	1	XX33
Known Interactor	5170	PDPK1	DB00482	Celecoxib	M	1	AH01
Known Interactor	5295	PIK3R1	DB01064	Isoprenaline	C	1	CA02
Known Interactor	5295	PIK3R1	DB01064	Isoprenaline	D	8	AX05
Known Interactor	5515	PPP2CA	DB00163	Vitamin E	A	11	HA03
Known Interactor	5553	PRG2	DB00020	Sargramostim	L	3	AA09
Known Interactor	5565	PRKAB2	DB00945	Acetylsalicylic acid	A	1	AD05
Known Interactor	5565	PRKAB2	DB00945	Acetylsalicylic acid	B	1	AC06
Known Interactor	5565	PRKAB2	DB00945	Acetylsalicylic acid	N	2	BA01
Known Interactor	5588	PRKCQ	DB00675	Tamoxifen	L	2	BA01
Known Interactor	5591	PRKDC	DB00201	Caffeine	N	2	BE01
Known Interactor	5591	PRKDC	DB00201	Caffeine	N	6	BC01
Known Interactor	5621	PRNP	DB00759	Tetracycline	A	1	AB13
Known Interactor	5621	PRNP	DB00759	Tetracycline	A	1	AB21
Known Interactor	5621	PRNP	DB00759	Tetracycline	D	6	AA02
Known Interactor	5621	PRNP	DB00759	Tetracycline	D	6	AA04
Known Interactor	5621	PRNP	DB00759	Tetracycline	J	1	AA03

Known Interactor	5621	PRNP	DB00759	Tetracycline	J	1	AA07
Known Interactor	5621	PRNP	DB00759	Tetracycline	J	1	AA09
Known Interactor	5621	PRNP	DB00759	Tetracycline	S	1	AA02
Known Interactor	5621	PRNP	DB00759	Tetracycline	S	1	AA09
Known Interactor	5621	PRNP	DB00759	Tetracycline	S	2	AA08
Known Interactor	5621	PRNP	DB00759	Tetracycline	S	3	AA02
Known Interactor	5770	PTPN1	DB01133	Tiludronate	M	5	BA05
Known Interactor	5879	RAC1	DB00514	Dextromethorphan	R	5	DA09
Known Interactor	5894	RAF1	DB07438	Sorafenib	L	1	XE05
Known Interactor	5894	RAF1	DB08896	Regorafenib	L	1	XE21
Known Interactor	5914	RARA	DB00210	Adapalene	D	10	AD03
Known Interactor	5914	RARA	DB00459	Acitretin	D	5	BB02
Known Interactor	5914	RARA	DB00523	Alitretinoin	D	11	AH04
Known Interactor	5914	RARA	DB00523	Alitretinoin	L	1	XX22
Known Interactor	5914	RARA	DB00799	Tazarotene	D	5	AX05
Known Interactor	5914	RARA	DB00926	Etretinate	D	5	BB01
Known Interactor	6383	SDC2	DB00020	Sargramostim	L	3	AA09
Known Interactor	6755	SSTR5	DB00104	Octreotide	H	1	CB02
Known Interactor	6755	SSTR5	DB04894	Vapreotide	H	1	CB04
Known Interactor	6755	SSTR5	DB06663	Pasireotide	H	1	CB05
Known Interactor	7015	TERT	DB00495	Zidovudine	J	5	AF01
Known Interactor	7133	TNFRSF1B	DB00005	Etanercept	L	4	AB01
Known Interactor	8600	TNFSF11	DB00480	Lenalidomide	L	4	AX04
Known Interactor	8600	TNFSF11	DB06643	Denosumab	M	5	BX04
Known Interactor	203068	TUBB	DB00361	Vinorelbine	L	1	CA04
Known Interactor	203068	TUBB	DB00541	Vincristine	L	1	CA02
Known Interactor	203068	TUBB	DB00570	Vinblastine	L	1	CA01
Known Interactor	203068	TUBB	DB08417	Podofilox	D	6	BB04
Known Interactor	203068	TUBB	DB01394	Colchicine	M	4	AC01
Known Interactor	7422	VEGFA	DB00112	Bevacizumab	L	1	XC07
Known Interactor	7422	VEGFA	DB01017	Minocycline	A	1	AB23
Known Interactor	7422	VEGFA	DB01017	Minocycline	J	1	AA08
Known Interactor	7422	VEGFA	DB01120	Gliclazide	A	10	BB09
Known Interactor	7422	VEGFA	DB01136	Carvedilol	C	7	AG02

Known Interactor	7422	VEGFA	DB01270	Ranibizumab	S	1	LA04
Known Interactor	7422	VEGFA	DB05294	Vandetanib	L	1	XE12
Known Interactor	7422	VEGFA	DB06779	Dalteparin	B	1	AB04
Known Interactor	7422	VEGFA	DB08885	Aflibercept	S	1	LA05
Known Interactor	79001	VKORC1	DB00170	Menadione	B	2	BA02
Known Interactor	79001	VKORC1	DB00266	Dicoumarol	B	1	AA01
Known Interactor	79001	VKORC1	DB00498	Phenindione	B	1	AA02
Known Interactor	79001	VKORC1	DB00682	Warfarin	B	1	AA03
Known Interactor	79001	VKORC1	DB00946	Phenprocoumon	B	1	AA04
Known Interactor	79001	VKORC1	DB01418	Acenocoumarol	B	1	AA07
Novel Interactor	833	CARS	DB04443	L-Cysteine	R	5	CB01
Novel Interactor	833	CARS	DB04443	L-Cysteine	S	1	XA08
Novel Interactor	833	CARS	DB04443	L-Cysteine	V	3	AB23
Novel Interactor	834	CASP1	DB01017	Minocycline	A	1	AB23
Novel Interactor	834	CASP1	DB01017	Minocycline	J	1	AA08
Novel Interactor	916	CD3E	DB00075	Muromonab	L	4	AA02
Novel Interactor	917	CD3G	DB00075	Muromonab	L	4	AA02
Novel Interactor	960	CD44	DB08818	Hyaluronan	D	3	AX05
Novel Interactor	960	CD44	DB08818	Hyaluronan	M	9	AX01
Novel Interactor	960	CD44	DB08818	Hyaluronan	R	1	AX09
Novel Interactor	960	CD44	DB08818	Hyaluronan	S	1	KA01
Novel Interactor	634	CEACAM1	DB00113	Arcitumomab	V	9	IA06
Novel Interactor	1595	CYP51A1	DB01007	Tioconazole	D	1	AC07
Novel Interactor	1595	CYP51A1	DB01007	Tioconazole	G	1	AF08
Novel Interactor	1595	CYP51A1	DB01167	Itraconazole	J	2	AC02
Novel Interactor	54583	EGLN1	DB00126	Vitamin C	A	11	GA01
Novel Interactor	54583	EGLN1	DB00126	Vitamin C	G	1	AD03
Novel Interactor	54583	EGLN1	DB00126	Vitamin C	S	1	XA15
Novel Interactor	2260	FGFR1	DB00039	Palifermin	V	3	AF08
Novel Interactor	2260	FGFR1	DB07438	Sorafenib	L	1	XE05
Novel Interactor	2260	FGFR1	DB08896	Regorafenib	L	1	XE21
Novel Interactor	3055	HCK	DB06616	Bosutinib	L	1	XE14
Novel Interactor	4758	NEU1	DB00198	Oseltamivir	J	5	AH02
Novel Interactor	4835	NQO2	DB00170	Menadione	B	2	BA02

Novel Interactor	4835	NQO2	DB08189	Melatonin	N	5	CH01
Novel Interactor	4835	NQO2	DB01087	Primaquine	P	1	BA03
Novel Interactor	6240	RRM1	DB00242	Cladribine	L	1	BB04
Novel Interactor	6240	RRM1	DB00441	Gemcitabine	L	1	BC05
Novel Interactor	6240	RRM1	DB07554	Clofarabine	L	1	BB06
Novel Interactor	6240	RRM1	DB01005	Hydroxyurea	L	1	XX05
Novel Interactor	6240	RRM1	DB01073	Fludarabine	L	1	BB05
Novel Interactor	7172	TPMT	DB01250	Olsalazine	A	7	EC03

Significantly Connected Drugs

We ranked drugs that target the genes in the schizophrenia interactome using Fisher's exact test. We compared the number of schizophrenia interactome genes that a drug targets compared to the total number of genes that same drug targets. Fisher's test computes the significance of that overlap as a p-value, which was further filtered using Benjamini-Hochberg correction for multiple testing. We showed the significantly connected drugs in dark green color in the drug-gene interactome figure.

P-value	Drug
0.000024	Tamoxifen
0.000828	Acetylsalicylic acid
0.004422	Phenobarbital
0.008544	Atomoxetine
0.008544	Estramustine
0.008544	Resveratrol
0.010423	Arsenic trioxide
0.020037	Flunarizine
0.020037	Remoxipride
0.020037	Secobarbital
0.020037	Staurosporine
0.020037	Sulindac
0.02949	Milnacipran
0.031159	Dehydroepiandrosterone
0.033901	ABT-263
0.033901	Alsterpaullone
0.033901	Butriptyline
0.033901	Flavopiridol
0.033901	Mesalazine
0.033901	Pentobarbital
0.033901	Phenylbutazone
0.033901	Vitamin E
0.044906	Dopamine
0.044906	Gavestinel
0.044906	Ibuprofen
0.044906	Iloperidone
0.044906	"Inositol 1,3,4,5-Tetrakisphosphate"
0.044906	Melatonin
0.044906	Memantine
0.044906	Mibefradil
0.044906	Minocycline
0.044906	Orphenadrine
0.044906	Pseudoephedrine
